# Effects of aged garlic extract and FruArg on gene expression and signaling pathways in lipopolysaccharide-activated microglial cells

**DOI:** 10.1038/srep35323

**Published:** 2016-10-13

**Authors:** Hailong Song, Yuan Lu, Zhe Qu, Valeri V. Mossine, Matthew B. Martin, Jie Hou, Jiankun Cui, Brenda A. Peculis, Thomas P. Mawhinney, Jianlin Cheng, C. Michael Greenlief, Kevin Fritsche, Francis J. Schmidt, Ronald B. Walter, Dennis B. Lubahn, Grace Y. Sun, Zezong Gu

**Affiliations:** 1 Department of Pathology & Anatomical Sciences, University of Missouri School of Medicine, Columbia, MO 65212, USA; 2 Center for Translational Neuroscience, University of Missouri School of Medicine, Columbia, MO 65212, USA; 3 Center for Botanical Interaction Studies, University of Missouri, Columbia, MO 65211, USA; 4 Department of Biochemistry, University of Missouri, Columbia, MO 65211, USA; 5 Xiphophorus Genetic Stock Center, Texas State University, San Marcos, TX 78666, USA; 6 Department of Computer Science, Informatics Institute, University of Missouri, Columbia, MO 65211, USA; 7 Department of Chemistry, University of Missouri, Columbia, MO 65211, USA; 8 Divison of Animal Sciences, University of Missouri, Columbia, MO 65211, USA

## Abstract

Aged garlic extract (AGE) is widely used as a dietary supplement on account of its protective effects against oxidative stress and inflammation. But less is known about specific molecular targets of AGE and its bioactive components, including *N*-α-(1-deoxy-D-fructos-1-yl)-L-arginine (FruArg). Our recent study showed that both AGE and FruArg significantly attenuate lipopolysaccharide (LPS)-induced neuroinflammatory responses in BV-2 microglial cells. This study aims to unveil effects of AGE and FruArg on gene expression regulation in LPS stimulated BV-2 cells. Results showed that LPS treatment significantly altered mRNA levels from 2563 genes. AGE reversed 67% of the transcriptome alteration induced by LPS, whereas FruArg accounted for the protective effect by reversing expression levels of 55% of genes altered by LPS. Key pro-inflammatory canonical pathways induced by the LPS stimulation included toll-like receptor signaling, IL-6 signaling, and Nrf2-mediated oxidative stress pathway, along with elevated expression levels of genes, such as *Il6, Cd14, Casp3, Nfkb1, Hmox1,* and *Tnf*. These effects could be modulated by treatment with both AGE and FruArg. These findings suggests that AGE and FruArg are capable of alleviating oxidative stress and neuroinflammatory responses stimulated by LPS in BV-2 cells.

Garlic (*Allium sativum L.*) has been recognized to offer health benefits including cardiovascular protective effects and cancer preventive effects^1^,^2^,^3^. Aged garlic extract (AGE) is a nutritional supplement prepared by prolonged extraction (normally for 20 months) of fresh garlic with 15–20% aqueous ethanol at room temperature. This product is odorless and appears to be superior to normal garlic in its antioxidant properties. AGE can act as a superoxide radical scavenger^4^,^5^,^6^. AGE was further shown to promote a potent antioxidant protection in cells by enhancing activity of the cellular antioxidant enzymes superoxide dismutase, catalase and glutathione peroxidase, and by increasing glutathione^7^,^8^. As a dietary supplement, AGE was shown to reduce total serum cholesterol, low-density lipoprotein, and systolic pressure in hypercholesterolemic patients and to inhibit platelet aggregation in both animal and human subjects^9^,^10^,^11^.

*N*-α-(1-deoxy-D-fructos-1-yl)-L-arginine (FruArg) is a major carbohydrate derivative and a component of non-sulfur containing nutraceuticals from AGE^12^,^13^. Quantitative analysis revealed that AGE contained 2.1–2.4 mmol/L of FruArg, but none was detected in either raw or heated garlic juice^14^. FruArg belongs to a family of fructose-amino acids in AGE, and is an Amadori rearrangement product arising from the condensation reaction between free glucose and arginine during early stages of the Maillard reaction, which is responsible for color formation in foods and, to a large extent, antioxidant properties of AGE^14^,^15^,^16^. Studies indicated the anti-tumor effects of certain species of fructose-amino acids are due mainly to their ability to inhibit cancer cell proliferation and adhesion^17^,^18^. Meanwhile, FruArg was shown to exhibit antioxidant activity and hydrogen peroxide scavenging capacity that is comparable to the potent hydrogen peroxide scavenging compound ascorbic acid^14^,^19^. Our previous study demonstrated that AGE and FruArg could suppress NO production in a concentration-dependent manner without affecting the cell viability in lipopolysaccharide (LPS)-induced mouse BV-2 microglial cells, suggesting AGE and FruArg have beneficial effects in mitigating neuroinflammation^20^.

In the present study, RNA-Seq analysis was conducted to assess global gene expression affected by AGE and FruArg in LPS-induced BV-2 microglial cells. Our results show that AGE is capable of repressing levels of pro-inflammatory mRNAs in LPS-treated cells and that FruArg is an active functional component in AGE that accounts for the protective effects. Additionally, we have also annotated the canonical pathways and functional networks that may be responsible for the protective effects of these botanical compounds. The present study addresses the regulatory function of AGE and FruArg on LPS-stimulated cells with respect to global gene expression by providing quantitative information about the population of mRNA species. The data broaden our understanding of the effect of AGE and FruArg to the level of gene expression regulation and suggested their therapeutic potential for mitigating inflammatory abnormalities in the aging individuals.

## Results

### LPS induced transcriptome alteration in BV-2 microglial cells

**Figure 1:**
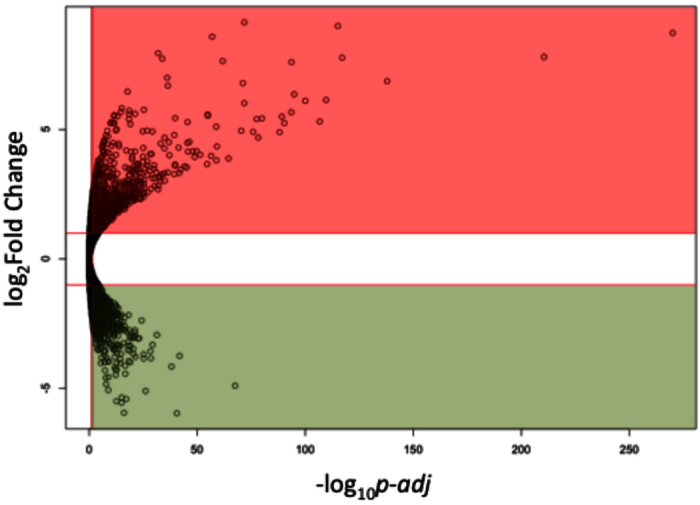
LPS induced gene changes in BV-2 microglial cells. LPS treatment induced differential expression of 2563 genes. For a gene to be considered as differentially expressed gene, the “log_2_Fold Change” has to ≥1 or ≤−1, with a FDR adjusted p-value ≤ 0.05. Red region highlighted up-regulated genes (1391 genes), green region highlighted down-regulated genes (1172 genes). Vertical redline represented *p-*adj = 0.05, two horizontal lines indicate log_2_Fold Change of +1 and −1.

Endotoxin LPS is one of the most potent stimuli for microglial cells. It is capable of activating multiple intracellular signaling pathways leading to cytokines secretion. The global gene expression profile alterations that were created by LPS stimulus was represented by the differentially expressed genes (DEGs) between BV-2 microglial cells treated with 100 ng/mL LPS and control cells. DEG analysis identified 2563 genes whose expression levels were altered significantly by LPS treatment (Fig. 1 and Supplementary Table S1). A total of 1172 genes were down-regulated and 1391 genes were up-regulated in response to the LPS. A number of genes associated with inflammation were significantly up-regulated, such as *Cd14, Il1a, Il1b, Nfkb1, Nfkb2, Tlr1, Tlr2, Tlr3, Tlr6,* and *Tnf*, which served as mRNA markers of LPS treatment in these cells. Their up-regulation proved the reliability of the LPS model used in the experiments^21^,^22^.

**Figure 2:**
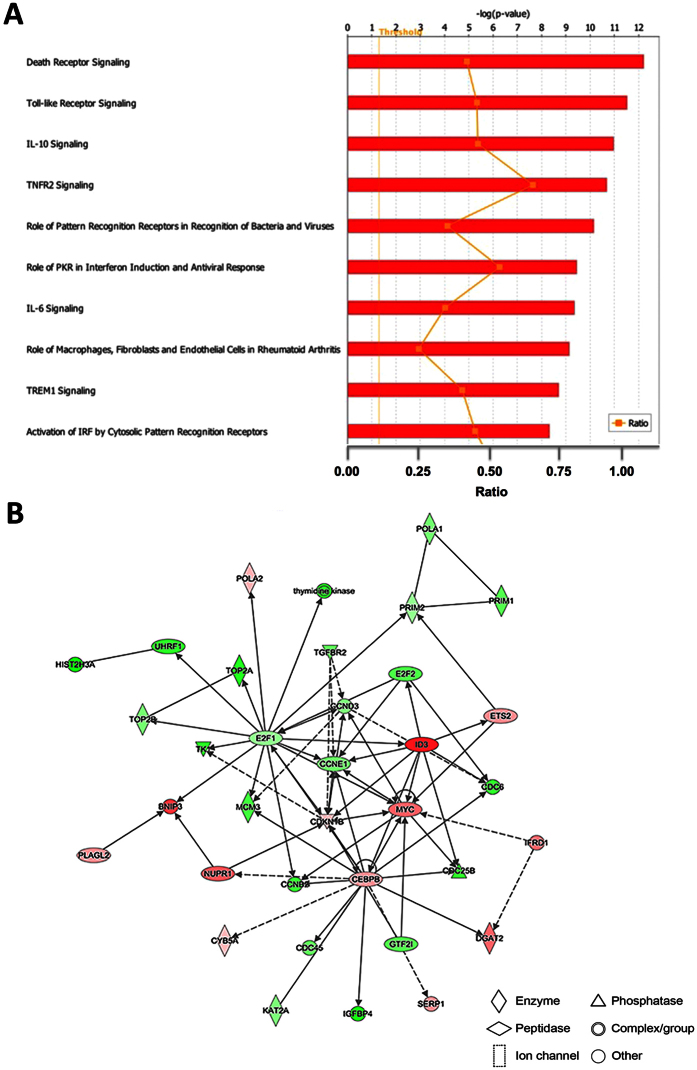
Effects of LPS in BV-2 microglial cells on top canonical pathways and disease/function networks. (**A**) Top 10 canonical pathways predicted by the differentially expressed genes corresponding to LPS stimulation of BV-2 cells. The canonical pathways were ranked according to the –log (p-value). A ratio indicates the numbers of genes that were differentially expressed in each pathway over the total numbers of genes in that specific pathway. (**B**) The top disease and function network is associated with cell cycle, cellular development, and cellular growth and proliferation corresponding to the LPS stimulation of BV-2 cells. The identified genes involved in the networks were displayed in red (up-regulation) and green (down-regulation) color. The color intensity indicates the degree of regulation. Solid lines in the network imply direct interactions between genes, and dashed lines indicate indirect interactions. Geometric shapes represent different general functional families of gene regulation (diamond for enzyme, oval for transcription regulator, trapezoid for transporter, inverted triangle for kinase, double circle for complex/group, and circle for others).

To evaluate which canonical signaling pathways and signaling networks could be activated upon LPS treatment, we performed a gene set enrichment analysis by Ingenuity Pathway Analysis (IPA). The most significant canonical pathways for the genes altered by LPS include death receptor signaling, toll-like receptor signaling, IL-10 signaling, TNFR2 signaling, role of pattern recognition receptors in recognition of bacteria and viruses, role of PKR in interferon induction and antiviral response, IL-6 signaling, role of macrophages, fibroblasts and endothelial cells in rheumatoid arthritis, TREM1 signaling, and activation of IRF by cytosolic pattern recognition receptors (Fig. 2A) (see Supplementary Table S4). Additionally, the disease and function networks were further analyzed. The top-ranked network is related to cell cycle, cellular development, and cellular growth and proliferation, with 34 focus genes, including *Serp1, Ifrd1,* and *Nupr1* (Fig. 2B). Among these focus genes, 21 genes were up-regulated and 13 genes were down-regulated.

### AGE represses LPS-induced transcriptome alteration and FruArg partially contributes to the AGE’s LPS-repression activity

**Figure 3:**
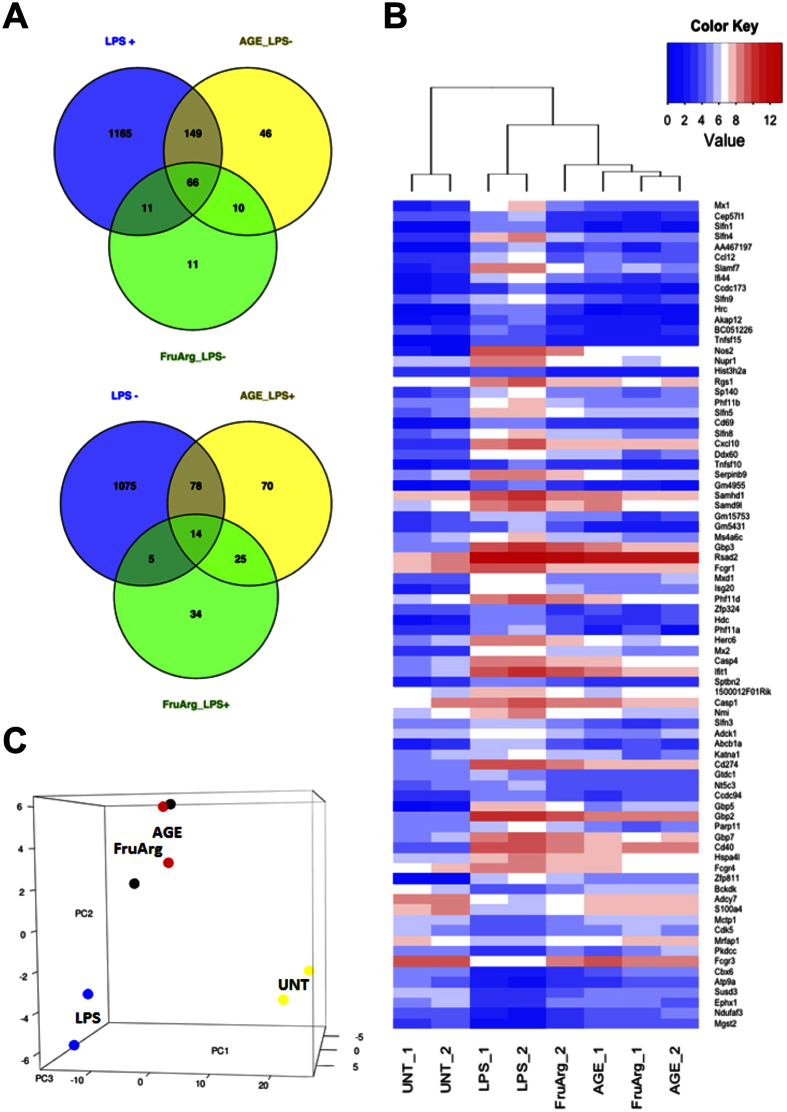
AGE and FruArg repress LPS-induced alteration of gene expression in BV-2 cells. (**A,B**) Venn graph and heatmap showing AGE co-treatment with LPS stimulation was capable of repressing LPS-altered genes (LPS+/−). A large portion of AGE co-treatment down-regulated genes (AGE_LPS-) were up-regulated by LPS; similarly, a large portion of AGE co-treatment up-regulated genes (AGE_LPS+) were down-regulated by LPS treatment alone. Similar effect as AGE was also observed after FruArg treatment (FruArg_LPS+/−). In addition, there are 80 genes that were found to have the same LPS repressing activity after AGE or FruArg treatment. (**C**) Principle component analysis showed that these 80 genes effectively separated untreated control (UNT) from LPS stimulus, as well as co-treatment of LPS with either AGE or FruArg.

We co-treated the BV-2 cells with LPS and AGE in order to test whether AGE is capable of reversing the LPS-induced transcriptome alteration. Co-administration of AGE with LPS antagonized LPS-induced changes in the levels of 458 mRNAs (see Supplementary Figure S1). The expression levels of 215 LPS-stimulated mRNAs were reduced by AGE co-treatment relative to the levels in cells treated with LPS alone. Conversely, the expression levels of 92 LPS-repressed mRNAs were elevated by AGE co-treatment relative to the levels in cells treated with LPS alone (Fig. 3A,B, and Supplementary Figure S3A and Supplementary Table S2). A total of 307 genes accounted for 67% of the mRNAs whose expression was affected by AGE were also affected by LPS, in an opposing manner.

Because FruArg has been reported to be a bioactive component of AGE^12^,^13^, we tested its effect on LPS-altered gene expression in BV-2 cells, hypothesizing that some mRNAs would be altered by treatment with this compound in similar gene expression patterns treated by AGE. RNA-Seq analysis showed that the LPS-induced transcriptome alterations were antagonized by FruArg treatment. When compared to LPS alone, the co-treatment with FruArg altered 175 DEGs (see Supplementary Figure S2). The LPS-induced expression pattern of 96 mRNAs was reversed by FruArg co-treatment. Of these 96, the expression level of 77 LPS-stimulated mRNAs was reduced by FruArg co-treatment; the expression level of 19 LPS-repressed mRNAs was stimulated by FruArg co-treatment. In total, co-treatment with FruArg antagonized the alterations of 55% of the LPS-altered mRNA. Additionally, the expression levels of 80 out of the 96 mRNAs whose expression was altered by co-treatment with FruArg (83%) were also altered in the same way by co-treatment with AGE (Fig. 3A,B, and Supplementary Figure S3B and Supplementary Table S3).

To further establish the relationship between AGE, FruArg and LPS, we also performed Principal Component Analysis (PCA) on the normalized expression values of these 80 genes. The results demonstrated the consistency of these effects (Fig. 3C and Table 1). Along the first component, which accounts for 81% of variance, LPS-treated samples were clearly separated from the untreated controls. AGE co-treatments and FruArg co-treatments, although not clearly separated, were isolated from LPS treatments, supporting the idea that FruArg and AGE have similar activity on the expression of microglial mRNAs.

### AGE and FruArg repressed LPS-altered canonical signaling pathways

**Figure 4:**
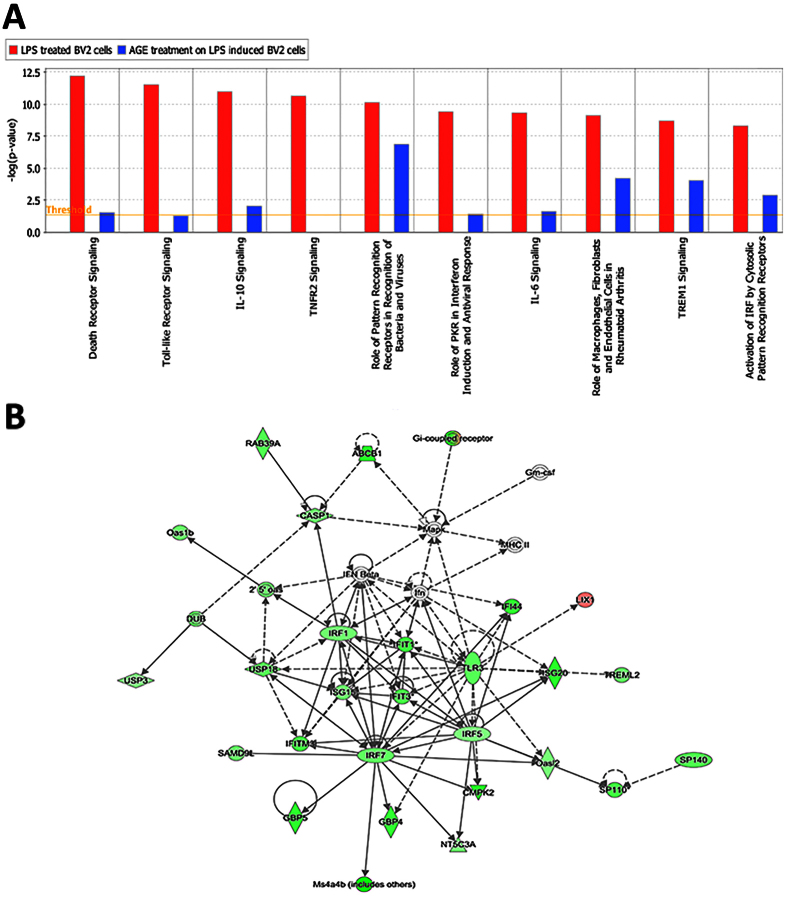
Effects of AGE on LPS-induced alterations of canonical pathways and disease/function networks in BV-2 microglial cells. (**A**) Top 10 canonical pathways affected by LPS stimulation were repressed by AGE treatment. (**B**) The top disease and function network is associated with antimicrobial response, inflammatory response, and infectious diseases corresponding to the effects of AGE on LPS stimulation of BV-2 cells.

**Figure 5:**
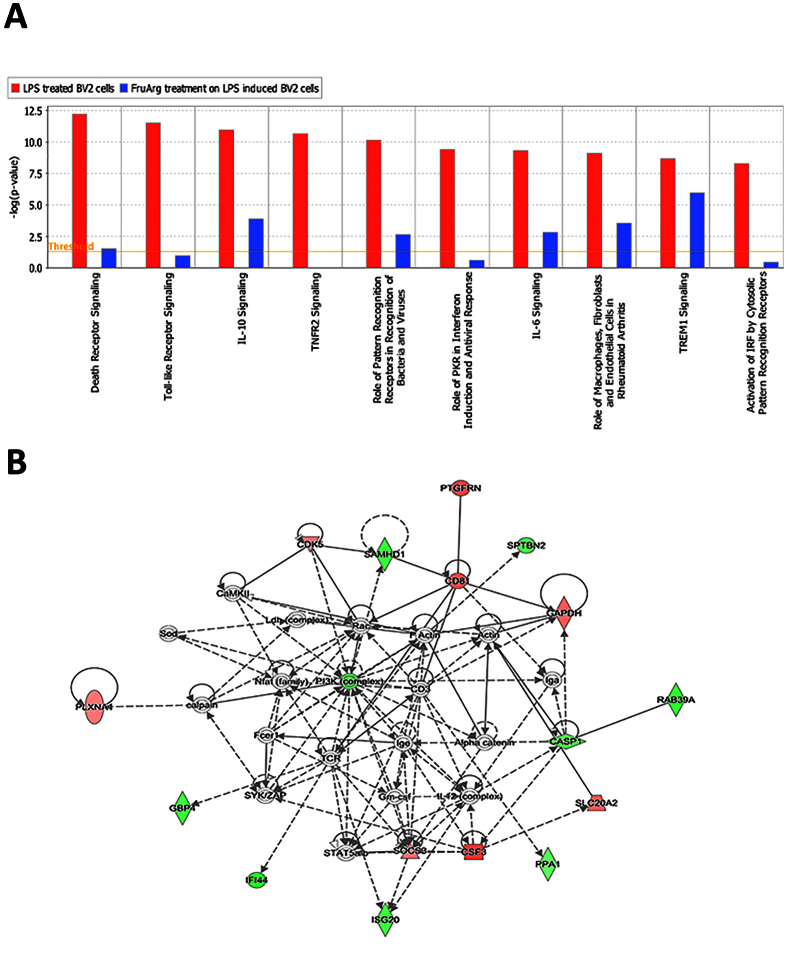
Effects of FruArg on LPS-induced alterations of canonical pathways and disease/function networks in BV-2 microglial cells. (**A**) Top 10 canonical pathways predicted by the differentially expressed genes corresponding to the effects of FruArg on LPS induction of BV-2 cells. (**B**) The top disease and function network is associated with cellular movement, cell death and survival, and cell morphology corresponding to the effects of FruArg on LPS stimulation of BV-2 cells.

To further understand the biological functions of AGE’s effects in LPS-stimulated BV-2 microglial cells, we mapped the mRNA identities to known signaling pathways and regulatory networks that were potentially affected by AGE treatment using IPA (Figs 4 and 5, and S4 and Supplementary Tables S5 and S6). The top 10 canonical pathways affected by the LPS stimulation and correlated genes, including *Il1a, Il1b, Cd14, Casp3, Casp7, Nfkb1, Nfkb2, Tnf, Traf1, Traf2, Hmox1, Il6,* and *Tank*, were repressed by AGE treatment (Fig. 4A) (see Supplementary Table S5). The most significant changes of canonical pathways modulated by AGE were TNFR2 signaling. Additionally, the disease/function networks that associated with AGE downregulated *Casp1, Tlr3, Serp1, Irf1, Irf5,* and *Irf7* genes were involved in the antimicrobial response, inflammatory response, and infectious diseases (Fig. 4B). This gene interaction network was designated by IPA with the highest number of focus molecules (n = 27).

We also analyzed the canonical pathways related to disease/function networks to determine the regulation of FruArg on LPS-induced BV-2 cells. Similar to AGE, the top 10 canonical pathways and related genes, including *Casp3, Casp7, Nfkb1, Nfkb2, Tnf, Traf2, Il1a, Il1b, Il6, Cd14, Il36g, Map2k3, Map2k6,* and *Hmox1,* affected by the LPS stimulation were all repressed by FruArg treatment (Fig. 5A, Supplementary Table S6). The most significant change of canonical pathways modulated by AGE was TNFR2 signaling.

Subsequently, we compared the canonical pathways regulated by both AGE and FruArg (see Supplementary Figure S4). AGE and FruArg share major canonical pathways such as role of pattern recognition receptor in recognition of bacteria and viruses, interferon signaling, dendritic cell maturation, activation of IRF by cystolic pattern recognition receptors, p53 signaling, and retinoic acid mediated apoptosis signaling, endothelin-1 signaling, death receptor signaling, role of NFAT in regulation of the immune response, Nrf2-mediated oxidative stress response, and acute phase response signaling. The data indicated FruArg and AGE targeted common pathways that were modulated by LPS. Moreover, the top network is associated with cellular movement, cell death and survival, and cell morphology by regulating genes, such as *Casp1, Cd81, Cdk5, Gapdh, Nupr1,* and *Serp1* (Fig. 5B). This gene interaction network was designated with the highest number of focus molecules (n = 16). Hence, the canonical pathways and cellular networks suggested that FruArg exhibits potent anti-inflammatory ability to reverse LPS-induced alterations.

## Discussion

Microglia serve as resident immune cells in the central nervous system (CNS). Chronic neuroinflammatory responses mediated by activated microglial cells in the central nervous system have critical roles in the pathogenesis of numerous neurodegenerative diseases, including Alzheimer’s disease and Parkinson’s disease^23^,^24^,^25^,^26^. Various inflammatory mediators and cytotoxic molecules, such as IL-1α, IL-6, TNF-α, ROS, and NO, released by chronic activated microglial cells in response to infection, injury, or endotoxins (e.g. LPS) may trigger neuronal damage and even cell death^27^,^28^,^29^,^30^. NO serves as a lipid-soluble free signaling molecule that transports freely across cell membranes^31^,^32^,^33^. Nitrosative stress can be induced by excessive production of NO and other reactive nitrogen species (RNS), which contributes to the pathogenesis of traumatic brain injury, Parkinson’s disease, and Alzheimer’s disease^34^,^35^,^36^,^37^,^38^,^39^. LPS stimulation can significantly increase NO concentration in microglia. Antioxidants such as AGE and FruArg could alleviate microglial activation and suppress the nitrosative stress by reducing NO production, and thus have a potential to counteract the undesirable effects of chronic activation of microglial cells. In the present study, we showed the altered expression of LPS responsive genes, including *Traf1, Nos2, Saa3, Lcn2, Il6, Irg1, Il1b,* and *Cxcl3*. The up-regulation of these genes confirmed the activation of BV-2 microglial cells by LPS. Especially, NOS2 and its gene product, inducible NOS (iNOS), is involved in neuroinflammation by generating NO and plays key roles in the pathogenesis of certain neurodegenerative disorders^40^,^41^,^42^. *Traf1* (tumor necrosis factor receptor-associated factor 1) gene is also reported to regulate the inflammatory response^43^,^44^. In addition, *Il6* gene is related to not only proinflammatory, but also neurodevelopment functions^45^,^46^. A total of 2563 genes were shown to respond to LPS stimulation. These genes are associated with death receptor signaling, toll-like receptor signaling, IL-10 signaling, TNFR2 signaling, role of pattern recognition receptors in recognition of bacteria and viruses canonical pathways (Fig. 2A). Top networks modulated by LPS exposure are related to cell cycle, cellular development, cellular growth and proliferation, protein synthesis, and cellular function and maintenance (Fig. 2B). Overall, our gene expression profiling in LPS-treated BV-2 microglial cells provided a useful set of pathway markers.

Both AGE and its bioactive component FruArg demonstrated a potential in neuronal protection against cellular oxidative stress and inflammatory responses by directly scavenging superoxide free radicals^4^,^5^,^6^,^18^,^20^. The neuroprotective effects of AGE and FruArg were realized through anti-oxidant/anti-inflammatory activities and targeting certain intracellular proteins and molecular pathways, according to data obtained by quantitative proteomic approach in our previous study^20^. We have demonstrated the significant suppression of NO production by either AGE or FruArg treatment and the multi-modal regulation of protein expression in LPS-induced BV-2 microglial cells. It also suggested that AGE and FruArg are similar to a number of botanicals by modulating the toll-like receptor and nuclear translocation of transcription factors for gene regulation^47^,^48^,^49^,^50^,^51^. Here in this study, we analyzed the gene expression profiles of both AGE and FruArg treatment to further investigate their effects on LPS-induced BV-2 microglial cells. 67% of AGE altered genes and 55% of FruArg altered genes showed reversed activity compared to LPS stimulation. It is clear that the major activity of AGE and FruArg is to repress LPS’s activity in gene expression. Furthermore, the shared modulation of genes by co-treatment of LPS between AGE and FruArg show that FruArg serves as a functional compound in AGE, in consistence with our previous proteomic study^20^, that accounts for its protective effects. We conclude that FruArg contributed to the anti-inflammatory effect of AGE by affecting mRNA levels associated with stress and inflammation.

FruArg is an Amadori product from AGE generated by the amino-carbonyl (Maillard) reaction, which is a nonenzymatic browning reaction of amino acids with sugars^16^. At the protein level, it was shown by both our proteomic analysis and other reports that the Maillard reaction products may decrease pro-oxidant activity in cells by affecting enzymatic activity of peroxiredoxin-1, mitochondrial superoxide dismutase 2, and glutamate-cysteine ligase in LPS-induced BV-2 cells^14^,^20^. At gene expression levels, FruArg also exhibited ability to suppress the production of certain inflammatory mediators by inhibiting the gene expression of *Il1a, Il1b, Nfkb1, Nfkb2, Traf1, Traf2, Casp1, Cd14, Il6,* and *Pi3kr5*. Additionally, by reducing the levels of NOS2 gene, FruArg could inhibit the excessive production of NO induced by LPS. The data demonstrate that FruArg can modulate and reverse stimulation of microglia by LPS at gene level. The ability to repress the pro-inflammatory response and oxidative stress both at protein and gene levels suggests that FruArg may have a medicinal value. On the other hand, from the perspective of canonical pathway analysis, both our previous proteomic and current RNA-Seq study show that FruArg can alter the Nrf2-mediated oxidative stress response pathway, which plays a major role for antioxidant protection^20^,^52^,^53^,^54^. Genes as *Gsta3, Gstm5, Gstp1, Hmox1,* and *Mgst2* related to Nrf2 mediated oxidative stress pathway were all up-regulated. The expression of these genes may enhance the subsequent activities of glutathione S-transferase and heme oxygenase 1 to catalyze the conjugation of reduced form of glutathione with excess oxidants, to detoxify and to catabolize free heme and produce carbon monoxide. Therefore, the neuroprotection mechanism of FruArg may involve both suppression of pro-inflammatory and promotion of antioxidant genes.

In conclusion, our results suggest that AGE and FruArg are capable of alleviating oxidative and neuroinflammatory responses stimulated by LPS in BV-2 cells by modulating gene expression. Inhibition of the expression of multiple immune- and inflammation-related genes suggested that AGE and FruArg could be drug candidates for the prevention of inflammation-mediated neurodegenerative diseases.

## Materials and Methods

### Cell culture and stimulation

The immortalized mouse microglial cells (BV-2) were originally obtained from Dr. R. Donato (University of Perugia, Italy) and were gifted from co-author Dr. Grace Y. Sun^55^,^56^. The cells, as previously described, were grown in Dulbecco’s modified Eagle’s medium (DMEM) (Gibco, Grand Island, NY, USA) supplemented with 5% (v/v) heat-inactivated fetal bovine serum (FBS) (Atlanta Biologicals, Inc., Lawrenceville, GA, USA), 25U/mL penicillin, and 25mg/mL streptomycin (Gibco, Grand Island, NY, USA) in a saturated humidity atmosphere containing 95% (v/v) air and 5% (v/v) CO_2_ at 37 °C^57^,^58^. Cells were cultured in DMEM without serum for 4 hours at 70–80% confluence and exposed to 100ng/mL LPS (rough strains from Escherichia coli F583 Rd mutant, Sigma-Aldrich, St. Louis, MO, USA) for 20 hours with or without aqueous AGE (0.5%, v/v) (Wakunaga of America, Mission Viejo, CA, USA) and FruArg (3 mM), which were added 1 hour prior to LPS exposure, respectively. FruArg was chemically synthesized and the purity and stability were measured as previously described^20^.

### Total RNA extraction and RNA-Seq

Total RNA was extracted using TRIzol (Life Technologies) according to the manufacture’s protocol. Briefly, 0.2 mL of chloroform was added to the homogenized sample and then the tubes were shaken for 15 seconds and incubated for 2–3 minutes at room temperature. Next, the mixture was centrifuged at 12,000 × g for 10 minutes at 4 °C, and the aqueous phase of the sample was extracted and placed into new tubes. Then, 0.5 mL of 100% isopropanol was added to the aqueous phase and incubated at room temperature for 10 minutes. The lysis mixture was centrifuged 12,000 × g for 10 minutes at 4 °C. The supernatant from the tube was removed and washed with 1 mL of 75% ethanol. The RNA pellet was vortexed briefly, centrifuged at 7,500 × g for 5 minutes at 4 °C, and dried at room temperature for 5–10 minutes. Finally, the RNA pellet was resuspended in RNase-free water or 0.5% SDS solution and incubated in a water bath or heat block set at 55–60 °C for 10–15 minutes. Total RNA concentration was determined using a spectrophotometer (NanoDrop Technologies, Wilmington, DE, USA). RNA quality was verified on an Agilent Bioanalyzer (Agilent Technologies, Santa Clara, CA) to confirm that RIN scores were above 7 prior to sequencing. RNA sequencing was performed upon libraries constructed using the Illumina TrueSeq library preparation system that employs a poly-A tail selection. RNA libraries were sequenced as 100nt single-end fragments using Illumina Hi-Seq 2000 system (Illumina, Inc., San Diego, CA, USA). Adaptor sequences were clipped off first. Subsequently, sequencing reads were trimmed and filtered based on quality scores by using fastx_toolbox (http://hannonlab.cshl.edu/fastx_toolkit/) that removed low-scoring sections of each read and preserved the longest remaining sequencing reads.

### Differential gene expression analysis

The trimmed and filtered reads were mapped to mouse genome (Mus_musculus.GRCm38) from Ensemble using Tophat2^59^. Mapped reads were quantified as raw counts by the Subreads package function “featureCounts”. Differentially expressed genes were analyzed using R/Bioconductor package DESeq2. For a gene to be a Differentially Expressed Genes (DEGs), it has to alter at least 2 fold with False Discovery Rate adjusted p-value (p-adj) less than 0.05 (Log2FC ≥1 or Log2FC ≤−1, FDR <0.05). For data visualization, the normalized gene expression value in the format of Count per Million reads (cpm) was log_2_-transformed and plotted by R package gplots^60^.

### Principal component analysis (PCA)

Raw gene counts were normalized to library sizes of each sample and further log_2_ transformed. PCA analysis was performed using R build-in package “prcomp”. The first three components, which accounted for 98% of the variance in dataset were plotted using R package “rgl”.

### Functional annotation and canonical pathway analysis

The data were analyzed using QIAGEN’s Ingenuity^®^ Pathway Analysis (IPA^®^, QIAGEN Redwood City, www.qiagen.com/ingenuity) for associated gene functions and pathways, as well as to predict gene interaction networks. The significance values for the canonical pathways and networks are calculated by right-tailed Fisher’s exact test. The cutoff p-value is 0.05. Taller bars equate to increased significance.

### Accessibility of raw sequencing data and accession number

The raw sequencing reads files and process data files were submitted to NCBI GEO. The accession number is GSE87531.

## Additional Information

**How to cite this article**: Song, H. *et al*. Effects of aged garlic extract and FruArg on gene expression and signaling pathways in lipopolysaccharide-activated microglial cells. *Sci. Rep.* **6**, 35323; doi: 10.1038/srep35323 (2016).

## Supplementary Material

Supplementary Information

## Figures and Tables

**Table 1:** Summary of PCA.

Importance of components	PC1	PC2	PC3	PC4	PC5	PC6	PC7	PC8
Standard deviation	15.32700	5.10028	4.56230	1.83078	1.30763	0.98858	0.70489	2.628E−15
Proportion of Variance	0.81490	0.09023	0.07220	0.01163	0.00593	0.00339	0.00172	0.000E + 00
Cumulative Proportion	0.81490	0.90513	0.97730	0.98895	0.99489	0.99828	1.00000	1.000E + 00

Principal components were ranked based on the proportion of variance they accounted in the dataset. The first 3 PCs accounted for most of the variance of the dataset.

## References

[b1] Stabler S. N., Tejani A. M., Huynh F. & Fowkes C. Garlic for the prevention of cardiovascular morbidity and mortality in hypertensive patients. Cochrane Database Syst Rev 8, CD007653, 10.1002/14651858.CD007653.pub2 (2012). PMC6885043 22895963

[b2] Rahman K. Effects of garlic on platelet biochemistry and physiology. Mol Nutr Food Res 51, 1335–1344, 10.1002/mnfr.200700058 (2007). 17966136

[b3] Amagase H., Schaffer E. M. & Milner J. A. Dietary components modify the ability of garlic to suppress 7, 12-dimethylbenz (a) anthracene-induced mammary DNA adducts. The Journal of nutrition 126, 817 (1996). 8613883 10.1093/jn/126.4.817

[b4] Moriguchi T., Saito H. & Nishiyama N. Anti‐ageing effect of aged garlic extract in the inbred brain atrophy mouse model. Clinical and experimental pharmacology and physiology 24, 235–242 (1997). 9131291 10.1111/j.1440-1681.1997.tb01813.x

[b5] Kyo E., Uda N., Kasuga S. & Itakura Y. Immunomodulatory effects of aged garlic extract. J Nutr 131, 1075S–1079S (2001). 11238820 10.1093/jn/131.3.1075S

[b6] Morihara N., Hayama M. & Fujii H. Aged garlic extract scavenges superoxide radicals. Plant Foods Hum Nutr 66, 17–21, 10.1007/s11130-011-0216-6 (2011). 21318303

[b7] Imai J. . Antioxidant and radical scavenging effects of aged garlic extract and its constituents. Planta medica 60, 417–420 (1994). 7997468 10.1055/s-2006-959522

[b8] Borek C. Antioxidant health effects of aged garlic extract. The Journal of nutrition 131, 1010S–1015S (2001). 11238807 10.1093/jn/131.3.1010S

[b9] Steiner M., Khan A. H., Holbert D. & Lin R. A double-blind crossover study in moderately hypercholesterolemic men that compared the effect of aged garlic extract and placebo administration on blood lipids. The American journal of clinical nutrition 64, 866–870 (1996). 8942410 10.1093/ajcn/64.6.866

[b10] Rahman K. & Billington D. Dietary supplementation with aged garlic extract inhibits ADP-induced platelet aggregation in humans. The Journal of nutrition 130, 2662–2665 (2000). 11053504 10.1093/jn/130.11.2662

[b11] Steiner M. & Li W. Aged garlic extract, a modulator of cardiovascular risk factors: a dose-finding study on the effects of AGE on platelet functions. The Journal of nutrition 131, 980S–984S (2001). 11238801 10.1093/jn/131.3.980S

[b12] Amagase H. Clarifying the real bioactive constituents of garlic. J Nutr 136, 716S–725S (2006). 16484550 10.1093/jn/136.3.716S

[b13] Mossine V. V. & Mawhinney T. P. 1-Amino-1-deoxy-D-fructose (“fructosamine”) and its derivatives. Adv Carbohydr Chem Biochem 64, 291–402, 10.1016/S0065-2318(10)64006-1 (2010). 20837201

[b14] Ryu K., Ide N., Matsuura H. & Itakura Y. Nα-(1-deoxy-D-fructos-1-yl)-L-arginine, an antioxidant compound identified in aged garlic extract. The Journal of nutrition 131, 972S–976S (2001). 11238799 10.1093/jn/131.3.972S

[b15] Ledl F. Chemical pathways of the Maillard reaction. The Maillard reaction in food processing, human nutrition and physiology 19–42 (1990).

[b16] Martins S. I., Jongen W. M. & Van Boekel M. A. A review of Maillard reaction in food and implications to kinetic modelling. Trends in Food Science & Technology 11, 364–373 (2000).

[b17] Mossine V. V., Glinsky V. V. & Mawhinney T. P. Antitumor effects of the early Maillard reaction products, in The Maillard Reaction: Interface between Aging, Nutrition and Metabolism, M.C. Thomas and J. Forbes, Editors. Royal Society of Chemistry 170–179 (2010).

[b18] Mossine V. V., Chopra P. & Mawhinney T. P. Interaction of tomato lycopene and ketosamine against rat prostate tumorigenesis. Cancer Research 68, 4384–4391 (2008). 18519700 10.1158/0008-5472.CAN-08-0108

[b19] Ide N., Lau B. H., Ryu K., Matsuura H. & Itakura Y. Antioxidant effects of fructosyl arginine, a Maillard reaction product in aged garlic extract. The Journal of nutritional biochemistry 10, 372–376 (1999). 15539313 10.1016/s0955-2863(99)00021-2

[b20] Zhou H. . Proteomic analysis of the effects of aged garlic extract and its FruArg component on lipopolysaccharide-induced neuroinflammatory response in microglial cells. PLoS One 9, e113531, 10.1371/journal.pone.0113531 (2014). 25420111 PMC4242640

[b21] Das A. . Dual RNA sequencing reveals the expression of unique transcriptomic signatures in lipopolysaccharide-induced BV-2 microglial cells. PloS one 10, e0121117 (2015). 25811458 10.1371/journal.pone.0121117 PMC4374676

[b22] Schneiderman A. I., Braver E. R. & Kang H. K. Understanding sequelae of injury mechanisms and mild traumatic brain injury incurred during the conflicts in Iraq and Afghanistan: persistent postconcussive symptoms and posttraumatic stress disorder. American journal of epidemiology 167, 1446–1452 (2008). 18424429 10.1093/aje/kwn068

[b23] Streit W. J. Microglial activation and neuroinflammation in Alzheimer’s disease: a critical examination of recent history. Frontiers in aging neuroscience 2 (2010). 10.3389/fnagi.2010.00022 PMC2890154 20577641

[b24] Rogers J., Mastroeni D., Leonard B., Joyce J. & Grover A. Neuroinflammation in Alzheimer’s disease and Parkinson’s disease: are microglia pathogenic in either disorder? International review of neurobiology 82, 235–246 (2007). 17678964 10.1016/S0074-7742(07)82012-5

[b25] Qian L. & Flood P. M. Microglial cells and Parkinson’s disease. Immunologic research 41, 155–164 (2008). 18512160 10.1007/s12026-008-8018-0

[b26] Town T., Nikolic V. & Tan J. The microglial. Journal of neuroinflammation 2, 24 (2005). 16259628 10.1186/1742-2094-2-24 PMC1298325

[b27] Wood P. Neuroinflammation: mechanisms and management. (Springer Science & Business Media, 2002).

[b28] Block M. L., Zecca L. & Hong J.-S. Microglia-mediated neurotoxicity: uncovering the molecular mechanisms. Nature Reviews Neuroscience 8, 57–69 (2007). 17180163 10.1038/nrn2038

[b29] Graeber M. B. & Streit W. J. Microglia: biology and pathology. Acta neuropathologica 119, 89–105 (2010). 20012873 10.1007/s00401-009-0622-0

[b30] Di Filippo M. . Persistent activation of microglia and NADPH drive hippocampal dysfunction in experimental multiple sclerosis. Scientific reports 6 (2016). 10.1038/srep20926 PMC4757867 26887636

[b31] Pacher P., Beckman J. S. & Liaudet L. Nitric oxide and peroxynitrite in health and disease. Physiological reviews 87, 315–424 (2007). 17237348 10.1152/physrev.00029.2006 PMC2248324

[b32] Bal-Price A. & Brown G. C. Inflammatory neurodegeneration mediated by nitric oxide from activated glia-inhibiting neuronal respiration, causing glutamate release and excitotoxicity. The Journal of Neuroscience 21, 6480–6491 (2001). 11517237 10.1523/JNEUROSCI.21-17-06480.2001 PMC6763071

[b33] Gibbons H. M. & Dragunow M. Microglia induce neural cell death via a proximity-dependent mechanism involving nitric oxide. Brain research 1084, 1–15 (2006). 16564033 10.1016/j.brainres.2006.02.032

[b34] Tsang A. H. & Chung K. K. Oxidative and nitrosative stress in Parkinson’s disease. Biochimica et Biophysica Acta (BBA)-Molecular Basis of Disease 1792, 643–650 (2009). 19162179 10.1016/j.bbadis.2008.12.006

[b35] Danielson S. R. & Andersen J. K. Oxidative and nitrative protein modifications in Parkinson’s disease. Free Radical Biology and Medicine 44, 1787–1794 (2008). 18395015 10.1016/j.freeradbiomed.2008.03.005 PMC2422863

[b36] Gu Z., Nakamura T., Yao D., Shi Z. & Lipton S. Nitrosative and oxidative stress links dysfunctional ubiquitination to Parkinson’s disease. Cell death and differentiation 12, 1202–1204 (2005). 16094397 10.1038/sj.cdd.4401705

[b37] Calabrese V. . Nitrosative stress, cellular stress response, and thiol homeostasis in patients with Alzheimer’s disease. Antioxidants & redox signaling 8, 1975–1986 (2006). 17034343 10.1089/ars.2006.8.1975

[b38] Di Pietro V. . Neuroglobin expression and oxidant/antioxidant balance after graded traumatic brain injury in the rat. Free Radical Biology and Medicine 69, 258–264 (2014). 24491879 10.1016/j.freeradbiomed.2014.01.032

[b39] Buchwalow I. . L-arginine-NO-cGMP signalling pathway in pancreatitis. Scientific reports 3, 1899 (2013). 23712581 10.1038/srep01899 PMC3664897

[b40] Colton C. A. . The effects of NOS2 gene deletion on mice expressing mutated human AβPP. Journal of Alzheimer’s disease: JAD 15, 571 (2008). 19096157 10.3233/jad-2008-15405 PMC2667339

[b41] Dawson V. L. & Dawson T. M. Nitric oxide in neurodegeneration. Progress in brain research 118, 215–229 (1998). 9932444 10.1016/s0079-6123(08)63210-0

[b42] Uttara B., Singh A. V., Zamboni P. & Mahajan R. Oxidative stress and neurodegenerative diseases: a review of upstream and downstream antioxidant therapeutic options. Current neuropharmacology 7, 65 (2009). 19721819 10.2174/157015909787602823 PMC2724665

[b43] Bryce P. J., Oyoshi M. K., Kawamoto S., Oettgen H. C. & Tsitsikov E. N. TRAF1 regulates Th2 differentiation, allergic inflammation and nuclear localization of the Th2 transcription factor, NIP45. International immunology 18, 101–111 (2006). 16352630 10.1093/intimm/dxh354

[b44] Cerhan J. R. . Genetic variation in 1253 immune and inflammation genes and risk of non-Hodgkin lymphoma. Blood 110, 4455–4463 (2007). 17827388 10.1182/blood-2007-05-088682 PMC2234796

[b45] Scheller J., Chalaris A., Schmidt-Arras D. & Rose-John S. The pro-and anti-inflammatory properties of the cytokine interleukin-6. Biochimica et Biophysica Acta (BBA)-Molecular Cell Research 1813, 878–888 (2011). 21296109 10.1016/j.bbamcr.2011.01.034

[b46] Baune B. T. . Interleukin-6 gene (IL-6): a possible role in brain morphology in the healthy adult brain. Journal of neuroinflammation 9, 125 (2012). 22695063 10.1186/1742-2094-9-125 PMC3464888

[b47] Yoon Y. D. . Toll-like receptor 4-dependent activation of macrophages by polysaccharide isolated from the radix of Platycodon grandiflorum. International immunopharmacology 3, 1873–1882 (2003). 14636836 10.1016/j.intimp.2003.09.005

[b48] Chino A. . Juzentaihoto, a Kampo medicine, enhances IL-12 production by modulating Toll-like receptor 4 signaling pathways in murine peritoneal exudate macrophages. International immunopharmacology 5, 871–882 (2005). 15778123 10.1016/j.intimp.2005.01.004

[b49] Patwardhan B. & Gautam M. Botanical immunodrugs: scope and opportunities. Drug discovery today 10, 495–502 (2005). 15809195 10.1016/S1359-6446(04)03357-4 PMC7128543

[b50] Pugh N. D. . Melanin: dietary mucosal immune modulator from Echinacea and other botanical supplements. International immunopharmacology 5, 637–647 (2005). 15710333 10.1016/j.intimp.2004.12.011

[b51] Zhao L., Lee J. Y. & Hwang D. H. Inhibition of pattern recognition receptor-mediated inflammation by bioactive phytochemicals. Nutrition reviews 69, 310–320 (2011). 21631512 10.1111/j.1753-4887.2011.00394.x PMC3881972

[b52] Zhang M. . Emerging roles of Nrf2 and phase II antioxidant enzymes in neuroprotection. Progress in neurobiology 100, 30–47 (2013). 23025925 10.1016/j.pneurobio.2012.09.003 PMC3623606

[b53] Sethy N. K., Singh M., Kumar R., Ilavazhagan G. & Bhargava K. Upregulation of transcription factor NRF2-mediated oxidative stress response pathway in rat brain under short-term chronic hypobaric hypoxia. Functional & integrative genomics 11, 119–137 (2011). 20922447 10.1007/s10142-010-0195-y

[b54] Jung J.-S. . Anti-inflammatory mechanism of exogenous C2 ceramide in lipopolysaccharide-stimulated microglia. Biochimica et Biophysica Acta (BBA)-Molecular and Cell Biology of Lipids 1831, 1016–1026 (2013). 23384839 10.1016/j.bbalip.2013.01.020

[b55] Blasi E., Barluzzi R., Bocchini V., Mazzolla R. & Bistoni F. Immortalization of murine microglial cells by a v-raf/v-myc carrying retrovirus. J Neuroimmunol 27, 229–237 (1990). 2110186 10.1016/0165-5728(90)90073-v

[b56] Bocchini V. . An immortalized cell line expresses properties of activated microglial cells. J Neurosci Res 31, 616–621, 10.1002/jnr.490310405 (1992). 1578513

[b57] Shen S. . Distinct signaling pathways for induction of type II NOS by IFNgamma and LPS in BV-2 microglial cells. Neurochem Int 47, 298–307, 10.1016/j.neuint.2005.03.007 (2005). 15955597

[b58] Jiang J. . Sutherlandia frutescens ethanol extracts inhibit oxidative stress and inflammatory responses in neurons and microglial cells. PLoS One 9, e89748, 10.1371/journal.pone.0089748 (2014). 24587007 PMC3934922

[b59] Li J. . From Gigabyte to Kilobyte: A Bioinformatics Protocol for Mining Large RNA-Seq Transcriptomics Data. PLoS One 10, e0125000, 10.1371/journal.pone.0125000 (2015). 25902288 PMC4406561

[b60] Lu Y., Li J., Cheng J. & Lubahn D. B. Genes targeted by the Hedgehog-signaling pathway can be regulated by Estrogen related receptor β. BMC molecular biology 16, 19 (2015). 26597826 10.1186/s12867-015-0047-3 PMC4657266

