# Supplementary material for: Effects of aged garlic extract and FruArg on gene expression and signaling pathways in lipopolysaccharide-activated microglial cells
Source: Sci Rep. 2016 Oct 13;6:35323. doi: 10.1038/srep35323 (PMC5062119; doi:10.1038/srep35323)
Supplement: Supplementary Information [file srep35323-s1.pdf]

Supplementary information for

Effects of aged garlic extract and FruArg on gene expression and signaling pathways in lipopolysaccharide-activated microglial cells

Hailong Song[1,2,3,+], Yuan Lu[4,8,+], Zhe Qu[1,2,3], Valeri V. Mossine[4], Matthew B. Martin[4], Jie Hou[3,5], Jiankun Cui[1,3], Brenda A. Peculis[4], Thomas P. Mawhinney[6], Jianlin Cheng[3,5], C. Michael Greenlief[3,6], Kevin Fritsche[3,7], Francis J. Schmidt[4], Ronald B. Walter[8], Dennis B. Lubahn[3,4], Grace Y. Sun[1,2,3,4], Zezong Gu[1,2,3,*]

[*]Corresponding author: guze@health.missouri.edu
[+]These authors contributed equally to this work.

Departments of [1]Pathology & Anatomical Sciences, and [4]Biochemistry, [2]Center for Translational Neuroscience, University of Missouri School of Medicine, Columbia, MO 652112, USA; [3]Center for Botanical Interaction Studies, Departments of [5]Computer Science, Informatics Institute, [6]Chemistry, and [7]Animal Sciences, University of Missouri, Columbia, MO 65211, USA; [8]Xiphophorus Genetic Stock Center, Texas State University, San Marcos, TX, 78666 USA

Corresponding author:
Dr. Zezong Gu, Department of Pathology & Anatomical Sciences, Center for Translational Neuroscience, University of Missouri-Columbia, School of Medicine, Columbia, MO 65212, USA
E-mail: guze@health.missouri.edu
Phone: 1-573-884-3880
Fax: 1-573-884-4612

Supplementary Figures and Figure Legends

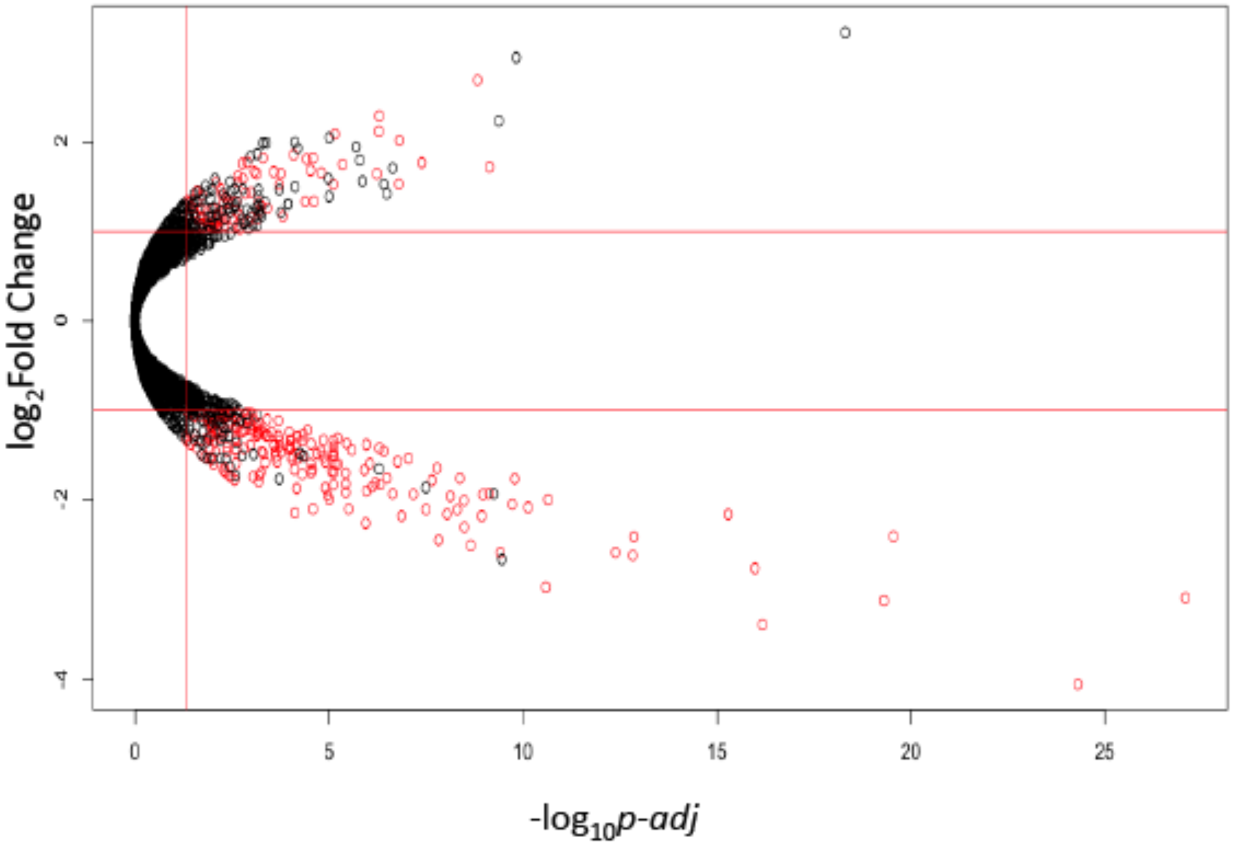

Supplementary Figure S1. Induced genes by co-treatment of AGE with LPS in BV-2 microglial cells. The co-treatment of AGE with LPS altered 458 DEGs in total compared to LPS stimulant alone group. For a gene to be considered as differentially expressed gene, the "$\log_2$Fold Change" has to ≥1 or ≤-1, with a FDR adjusted *p*-value ≤0.05. Red dots highlighted genes that were reversed by AGE co-treatment. Vertical redline represented *p*-adj = 0.05, two horizontal lines indicate $\log_2$Fold Change of +1 and -1.

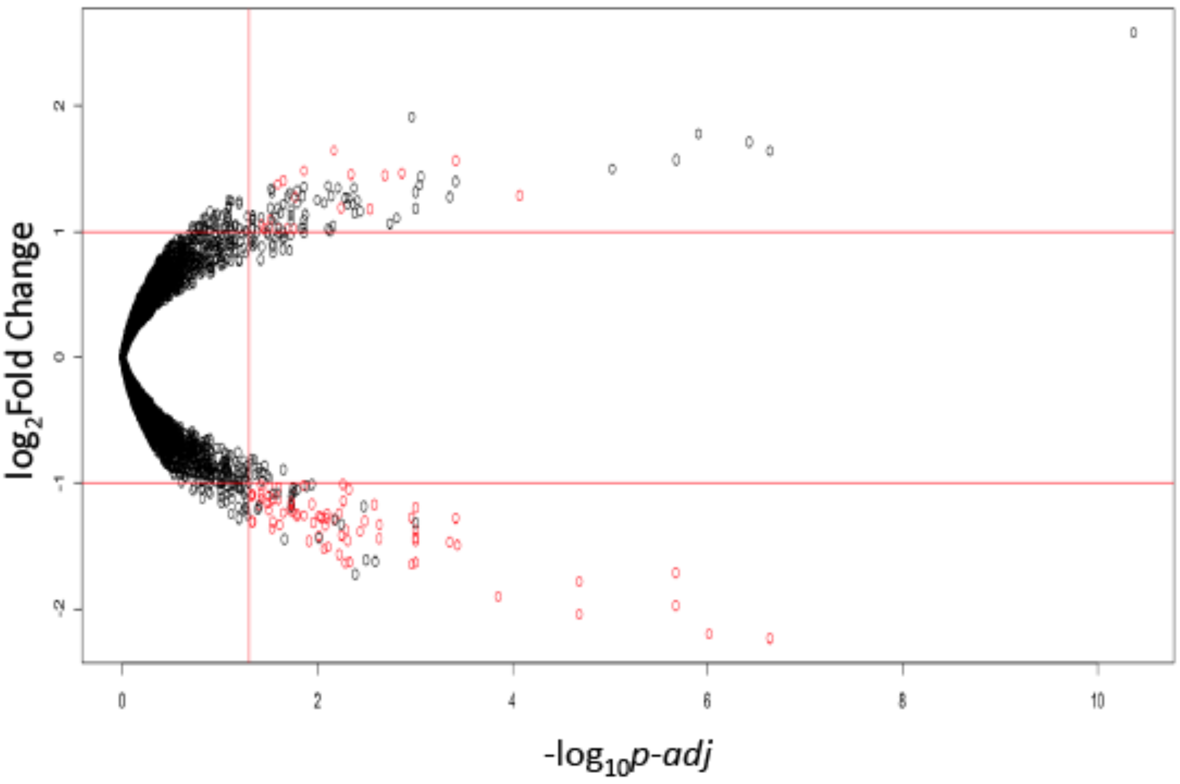

Supplementary Figure S2. Induced genes by co-treatment of FruArg with LPS in BV-2 microglial cells. The co-treatment of FruArg with LPS altered 175 DEGs in total compared to LPS stimulant alone group. For a gene to be considered as differentially expressed gene, the "$\log_2$Fold Change" has to ≥ 1 or ≤ -1, with a FDR adjusted *p*-value ≤ 0.05. Red dots highlighted genes that were reversed by FruArg co-treatment compared to the LPS induction. Vertical redline represented *p*-adj = 0.05, two horizontal lines indicate $\log_2$Fold Change of +1 and -1.

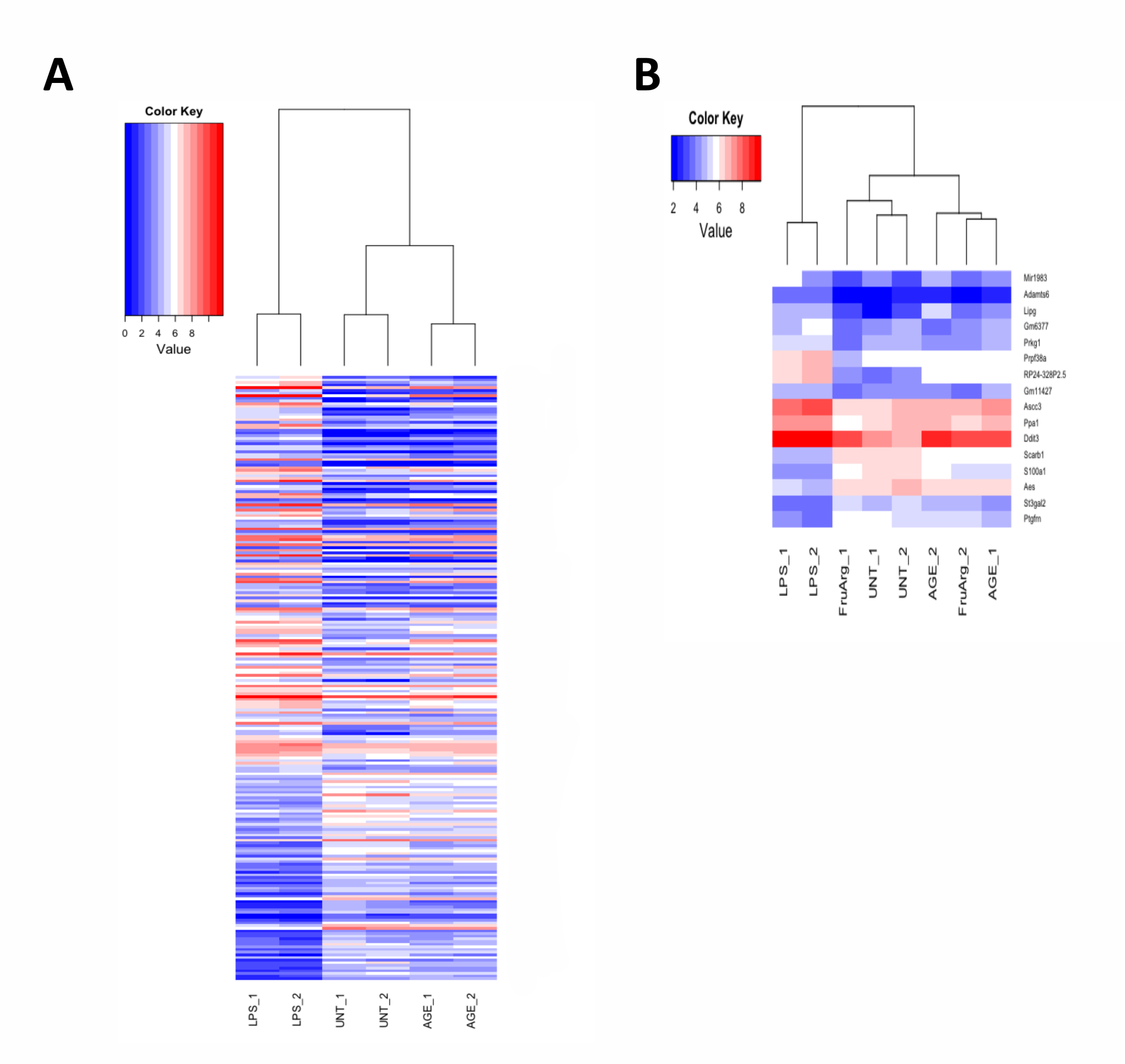

Supplementary Figure S3. LPS-altered genes that were repressed by AGE or FruArg independently. (A) Heatmap showing genes that were responsive to LPS, and were repressed by AGE co-treatment was shown. (B) Heatmap showing genes that were responsive to LPS, and were repressed by FruArg co-treatment was shown. These genes indicated AGE's and FruArg's LPS repression functions in LPS-induced BV-2 cells. Also, the repression effects of AGE come partly from FruArg.

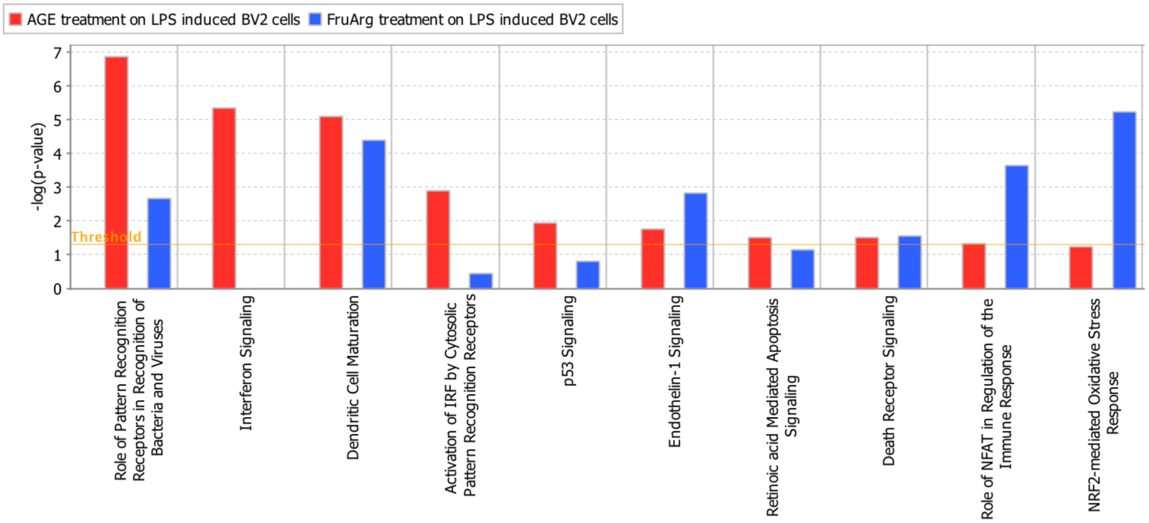

Supplementary Figure S4. Canonical pathways comparison between AGE and FruArg group. Top canonical pathways that were both affected by the AGE and FruArg treatment against LPS stimulation in BV-2 cells. The significances are based on the pathways' -log (p-value), which are calculated by Fisher's exact test right-tailed. Taller bars equate to increased significance.

Supplemental Tables

Supplementary Table S1. Differentially expressed genes of pair-wise comparison (LPS vs. UNT)

LPS vs. control						
ID	baseMean	log2FoldChange	lfcSE	stat	p-value	padj
ENSMUSG00000051212	254.7733825	-5.959649858	0.429648687	-13.87098353	9.50E-44	2.71E-41
ENSMUSG00000038204	61.71320853	-5.94325467	0.675307317	-8.800814863	1.36E-18	7.76E-17
ENSMUSG00000064065	56.92518293	-5.553704318	0.653175633	-8.502620185	1.85E-17	9.81E-16
ENSMUSG00000002104	46.67227177	-5.495951812	0.703363883	-7.813810097	5.55E-15	2.23E-13
ENSMUSG00000099517	69.97112706	-5.411049542	0.596235008	-9.075363691	1.13E-19	7.22E-18
ENSMUSG00000029570	60.1467743	-5.351016905	0.62801661	-8.520502203	1.59E-17	8.57E-16
ENSMUSG00000069310	134.5536035	-5.100564956	0.456262146	-11.17902285	5.17E-29	7.61E-27
ENSMUSG00000027670	25.77444781	-5.064873496	0.772661085	-6.555103648	5.56E-11	1.37E-09
ENSMUSG00000071042	972.026406	-4.896386309	0.274784933	-17.81897665	5.03E-71	3.00E-68
ENSMUSG00000035165	21.97215958	-4.837575504	0.78467663	-6.165056177	7.05E-10	1.47E-08
ENSMUSG00000061991	23.84652317	-4.587651788	0.750100323	-6.116050943	9.59E-10	1.96E-08
ENSMUSG00000058773	474.1275441	-4.562660258	0.693895969	-6.575424073	4.85E-11	1.21E-09
ENSMUSG00000022419	54.01660943	-4.479641341	0.582285721	-7.693201434	1.43E-14	5.51E-13
ENSMUSG00000101355	109.6738908	-4.434706513	0.494536	-8.967408876	3.04E-19	1.87E-17
ENSMUSG00000026955	24.86483171	-4.304783951	0.722137628	-5.961168321	2.50E-09	4.83E-08
ENSMUSG00000064288	62.21900075	-4.284898373	0.54616832	-7.845380654	4.32E-15	1.77E-13
ENSMUSG00000064168	53.64214217	-4.25984298	0.554659883	-7.680099296	1.59E-14	6.03E-13
ENSMUSG00000020092	58.93187123	-4.186786379	0.554063234	-7.556513633	4.14E-14	1.49E-12
ENSMUSG00000026360	1580.007267	-4.156992208	0.309007349	-13.45272925	2.97E-41	7.53E-39
ENSMUSG00000096807	106.5064525	-4.126907994	0.460910924	-8.953808172	3.43E-19	2.09E-17
ENSMUSG00000100210	78.41200682	-4.024931338	0.513875548	-7.832502158	4.78E-15	1.94E-13
ENSMUSG00000101972	77.08179297	-4.023890332	0.483403182	-8.324087397	8.50E-17	4.12E-15
ENSMUSG00000069302	16.56085499	-4.02095879	0.783251851	-5.133672882	2.84E-07	3.95E-06
ENSMUSG00000091089	27.96577346	-3.991308187	0.67678098	-5.897488705	3.69E-09	6.94E-08
ENSMUSG00000069265	173.45305	-3.98979415	0.4183711	-9.53649528	1.48E-21	1.12E-19

	95		14			
ENSMUSG00000009614	33.939813 61	-3.989399891	0.6677953 5	-5.973985727	2.32E-09	4.49E-08
ENSMUSG00000051339	42.801021 01	-3.964962867	0.5938274 76	-6.676960947	2.44E-11	6.27E-10
ENSMUSG00000069301	37.029204 28	-3.945123164	0.6109760 69	-6.457082956	1.07E-10	2.50E-09
ENSMUSG00000026357	117.42415 12	-3.942078702	0.4201119 23	-9.383401158	6.39E-21	4.56E-19
ENSMUSG00000022033	81.014251 54	-3.871145729	0.4652629 84	-8.320338961	8.77E-17	4.23E-15
ENSMUSG00000034041	236.43213 66	-3.846898555	0.3497950 57	-10.99757839	3.93E-28	5.38E-26
ENSMUSG00000043008	245.55097 88	-3.838236753	0.3287640 24	-11.67474686	1.72E-31	2.80E-29
ENSMUSG00000067455	39.130891 41	-3.821292922	0.6062985 24	-6.302659122	2.93E-10	6.41E-09
ENSMUSG00000018459	21.989735 36	-3.803969208	0.7215681 49	-5.271808647	1.35E-07	1.97E-06
ENSMUSG00000026068	73.858255 37	-3.790525197	0.5025195 97	-7.543039553	4.59E-14	1.64E-12
ENSMUSG00000024663	163.98813 5	-3.785077827	0.3731461 1	-10.1436883	3.53E-24	3.59E-22
ENSMUSG00000025154	1368.7827 83	-3.743269802	0.2658530 75	-14.08022004	5.03E-45	1.53E-42
ENSMUSG00000030867	32.090476 29	-3.727635228	0.6370254 9	-5.851626487	4.87E-09	8.87E-08
ENSMUSG00000028396	388.45782 5	-3.682427043	0.3352752 88	-10.98329395	4.60E-28	6.24E-26
ENSMUSG00000052565	488.45787 25	-3.675786555	0.3709814 41	-9.908276115	3.83E-23	3.41E-21
ENSMUSG00000040204	114.84555 87	-3.665306403	0.4354956 61	-8.416401662	3.88E-17	1.97E-15
ENSMUSG00000031788	130.11947	-3.645653133	0.4613372 11	-7.902360887	2.74E-15	1.14E-13
ENSMUSG00000048922	31.759559 11	-3.591543526	0.6244783 89	-5.751269526	8.86E-09	1.56E-07
ENSMUSG00000022885	318.19349 65	-3.561204319	0.3059751 26	-11.63886871	2.61E-31	4.22E-29
ENSMUSG00000041633	36.313330 5	-3.553796558	0.5899393 63	-6.024003107	1.70E-09	3.35E-08
ENSMUSG00000069274	361.96064 2	-3.547661788	0.3732826 29	-9.503956278	2.02E-21	1.51E-19
ENSMUSG00000020492	34.115074 18	-3.541112022	0.6040612 65	-5.862173632	4.57E-09	8.38E-08
ENSMUSG00000017716	78.007568 72	-3.538896279	0.4662918 84	-7.589444293	3.21E-14	1.17E-12
ENSMUSG00000084220	14.740582 53	-3.522597301	0.7736530 32	-4.553200407	5.28E-06	5.64E-05
ENSMUSG00000045382	225.82597 55	-3.50953545	0.3791871 24	-9.255418297	2.13E-20	1.43E-18
ENSMUSG00000017493	276.85086 08	-3.501705097	0.7498973 57	-4.669579197	3.02E-06	3.40E-05
ENSMUSG00000020649	96.264664	-3.501035681	0.4743441	-7.380792641	1.57E-13	5.27E-12

	24		32			
ENSMUSG00000049932	208.72537 19	-3.476393493	0.3513608	-9.894084645	4.42E-23	3.83E-21
ENSMUSG00000027654	11.567067 88	-3.469725097	0.8161793 26	-4.251179839	2.13E-05	0.00019 928
ENSMUSG00000069309	23.036088 44	-3.463030002	0.6918444 03	-5.005504111	5.57E-07	7.19E-06
ENSMUSG00000097654	26.312738 91	-3.456470651	0.6715090 89	-5.147317747	2.64E-07	3.71E-06
ENSMUSG00000033952	76.904210 29	-3.44940819	0.4646543 65	-7.4236001	1.14E-13	3.88E-12
ENSMUSG00000029177	108.05661 98	-3.436730145	0.4252791 74	-8.081115555	6.42E-16	2.88E-14
ENSMUSG00000024590	87.268332 29	-3.428510489	0.4539030 12	-7.553398843	4.24E-14	1.52E-12
ENSMUSG00000030346	65.010169 42	-3.380592425	0.4809450 93	-7.029061059	2.08E-12	5.97E-11
ENSMUSG00000052512	69.947887 38	-3.36273289	0.4607546 55	-7.298315608	2.91E-13	9.31E-12
ENSMUSG00000027715	181.45788 46	-3.362414665	0.3513412 6	-9.570224295	1.07E-21	8.21E-20
ENSMUSG00000036777	110.81910 98	-3.347420034	0.4011289 38	-8.344997627	7.12E-17	3.49E-15
ENSMUSG00000024232	193.57328 63	-3.344524436	0.3583879 83	-9.332133315	1.04E-20	7.22E-19
ENSMUSG00000062727	94.469906 74	-3.336756589	0.4196005 37	-7.952221925	1.83E-15	7.80E-14
ENSMUSG00000026683	75.280624 6	-3.332695414	0.4556555 66	-7.314067171	2.59E-13	8.34E-12
ENSMUSG00000040093	108.97330 09	-3.331880168	0.4109796 11	-8.107166575	5.18E-16	2.36E-14
ENSMUSG00000038332	405.28124 47	-3.315631526	0.2802451 05	-11.83118446	2.69E-32	4.67E-30
ENSMUSG00000036362	49.157081 43	-3.306325759	0.5373150 13	-6.153421508	7.58E-10	1.57E-08
ENSMUSG00000000686	8.5012054 17	-3.303886389	0.8582567 72	-3.849531397	0.000118 344	0.00094 1873
ENSMUSG00000069267	91.020065 93	-3.278329925	0.4212709 04	-7.781999407	7.14E-15	2.84E-13
ENSMUSG00000020658	133.97640 82	-3.253443231	0.3717038 42	-8.752783439	2.08E-18	1.17E-16
ENSMUSG00000046591	21.302884 39	-3.246051967	0.6867456 49	-4.726716469	2.28E-06	2.65E-05
ENSMUSG00000020897	58.127959 53	-3.245112385	0.4863756 07	-6.672029478	2.52E-11	6.47E-10
ENSMUSG00000033857	23.745467 87	-3.236027887	0.6732950 11	-4.806255552	1.54E-06	1.83E-05
ENSMUSG00000002007	10.437662 86	-3.227694377	0.8255693 88	-3.909658501	9.24E-05	0.00075 5792
ENSMUSG00000041064	32.860513 49	-3.227233581	0.6053687 76	-5.331020874	9.77E-08	1.46E-06
ENSMUSG00000044749	44.537771 89	-3.225886254	0.5333505 08	-6.048341951	1.46E-09	2.91E-08
ENSMUSG00000027199	174.23237	-3.214813849	0.3452062	-9.31273268	1.25E-20	8.54E-19

	43		85			
ENSMUSG00000024063	35.314605 99	-3.204168884	0.5780093 58	-5.543455032	2.97E-08	4.80E-07
ENSMUSG00000020808	59.850184 66	-3.152973914	0.4822139 55	-6.538537261	6.21E-11	1.51E-09
ENSMUSG00000004609	86.475463 05	-3.103018815	0.4760520 79	-6.518233936	7.11E-11	1.72E-09
ENSMUSG00000094338	23.507197 19	-3.09917322	0.6588346 01	-4.704023159	2.55E-06	2.93E-05
ENSMUSG00000040522	30.721800 85	-3.09117065	0.6053417 22	-5.106488679	3.28E-07	4.48E-06
ENSMUSG00000024013	115.81962 96	-3.088262644	0.4396852 24	-7.02380356	2.16E-12	6.19E-11
ENSMUSG00000020061	7.1154540 63	-3.083622032	0.8664611 11	-3.558869513	0.000372 455	0.00255 7359
ENSMUSG00000062960	308.79874 88	-3.080768925	0.2950143 42	-10.44277678	1.58E-25	1.76E-23
ENSMUSG00000028555	20.811253 88	-3.074291667	0.6921512 89	-4.441646958	8.93E-06	9.10E-05
ENSMUSG00000036768	42.990849 38	-3.073908719	0.5366591 53	-5.727860419	1.02E-08	1.77E-07
ENSMUSG00000021876	493.63312 37	-3.062728668	0.2913924 67	-10.51066524	7.71E-26	8.81E-24
ENSMUSG00000027435	257.97990 22	-3.0563768	0.3086186 65	-9.903408796	4.02E-23	3.56E-21
ENSMUSG00000005718	13.011426 72	-3.03206776	0.7755736 14	-3.909451926	9.25E-05	0.00075 5988
ENSMUSG00000002799	89.867310 6	-3.031646631	0.5274534 32	-5.74770481	9.05E-09	1.59E-07
ENSMUSG00000060600	360.40733 26	-3.015935126	0.3169447 02	-9.515650858	1.81E-21	1.36E-19
ENSMUSG00000028238	476.22588 29	-3.012737928	0.3043177 13	-9.899975587	4.16E-23	3.66E-21
ENSMUSG00000048779	31.615453 59	-3.008989421	0.5939661 8	-5.065927189	4.06E-07	5.40E-06
ENSMUSG00000075031	263.87452 23	-3.001092108	0.3094431 29	-9.698364006	3.06E-22	2.46E-20
ENSMUSG00000021719	33.043308 66	-2.999359229	0.6006937 38	-4.993158808	5.94E-07	7.64E-06
ENSMUSG00000020914	496.85449 06	-2.990587245	0.2955234 82	-10.11962646	4.52E-24	4.49E-22
ENSMUSG00000003134	11.137063 12	-2.978241797	0.8083246 86	-3.684462254	0.000229 186	0.00167 6334
ENSMUSG00000023015	126.93114 29	-2.974248139	0.3698695 91	-8.04134272	8.89E-16	3.94E-14
ENSMUSG00000022034	36.719931 07	-2.968093426	0.5800966 19	-5.116550121	3.11E-07	4.28E-06
ENSMUSG00000055782	118.32107 75	-2.966026889	0.3949543 63	-7.509796486	5.92E-14	2.09E-12
ENSMUSG00000039748	108.13286 36	-2.963156252	0.3941450 72	-7.517933022	5.56E-14	1.98E-12
ENSMUSG00000024168	12.081726 73	-2.950074351	0.7932587 9	-3.718930553	0.000200 068	0.00150 1606
ENSMUSG00000071516	43.004408	-2.943230769	0.5494496	-5.356688819	8.48E-08	1.28E-06

	7		45			
ENSMUSG00000045287	8.150766223	-2.938467019	0.845215247	-3.476590171	0.000507833	0.003359765
ENSMUSG00000005410	334.5626739	-2.936788239	0.308266388	-9.526787057	1.62E-21	1.23E-19
ENSMUSG00000030272	1290.040164	-2.935386282	0.240246403	-12.21823196	2.48E-34	4.66E-32
ENSMUSG00000026080	72.75750604	-2.92252305	0.443556244	-6.588844342	4.43E-11	1.12E-09
ENSMUSG00000046057	6.230844298	-2.918731713	0.869748668	-3.355833497	0.000791262	0.00494043
ENSMUSG00000041147	29.42418994	-2.916816085	0.602890529	-4.838052591	1.31E-06	1.58E-05
ENSMUSG00000058624	27.44217417	-2.910583079	0.617902591	-4.710423809	2.47E-06	2.85E-05
ENSMUSG00000042489	80.19597862	-2.906023425	0.432723899	-6.715652707	1.87E-11	4.88E-10
ENSMUSG00000069300	151.3397253	-2.902386265	0.394915771	-7.349380499	1.99E-13	6.59E-12
ENSMUSG00000074170	8.026110426	-2.897014838	0.846931552	-3.42060091	0.00062483	0.00403548
ENSMUSG00000046179	12.27299271	-2.89086403	0.796489911	-3.629504895	0.000283965	0.002022737
ENSMUSG00000026817	12.08125863	-2.883366799	0.785248611	-3.671915822	0.000240739	0.001754962
ENSMUSG00000078521	36.18526063	-2.878200819	0.562310202	-5.118528546	3.08E-07	4.24E-06
ENSMUSG00000027326	55.55073954	-2.875089037	0.498240562	-5.770483689	7.90E-09	1.40E-07
ENSMUSG00000006398	75.36843123	-2.865252404	0.443413461	-6.46180744	1.03E-10	2.43E-09
ENSMUSG00000062585	114.4083705	-2.853013439	0.391326439	-7.290622743	3.09E-13	9.83E-12
ENSMUSG00000093656	159.2191903	-2.852097399	0.373037705	-7.645600855	2.08E-14	7.81E-13
ENSMUSG00000028551	48.10088347	-2.851890466	0.518820454	-5.496873614	3.87E-08	6.10E-07
ENSMUSG00000005583	420.9331816	-2.849710589	0.282636372	-10.08260389	6.60E-24	6.32E-22
ENSMUSG00000049791	13.04245776	-2.849628519	0.767309484	-3.713792906	0.000204176	0.001526585
ENSMUSG00000073434	27.01970401	-2.842870595	0.63928274	-4.446969109	8.71E-06	8.89E-05
ENSMUSG00000099722	20.05735638	-2.841698399	0.677966271	-4.191504095	2.77E-05	0.000254204
ENSMUSG00000025141	17.00197296	-2.834843621	0.726307294	-3.903091218	9.50E-05	0.000772916
ENSMUSG00000060093	84.07754222	-2.831456923	0.471482265	-6.005436749	1.91E-09	3.72E-08
ENSMUSG00000024011	5.844857723	-2.831324693	0.871784905	-3.247733102	0.001163283	0.006922622
ENSMUSG00000056145	5.923955195	-2.830547001	0.872249766	-3.245110645	0.00117405	0.006977604
ENSMUSG00000069273	139.84073	-2.829127439	0.4310988	-6.562595765	5.29E-11	1.31E-09

	54		43			
ENSMUSG00000035314	54.65324822	-2.823798386	0.529520402	-5.332747101	9.67E-08	1.45E-06
ENSMUSG00000022540	26.4329086	-2.821489473	0.62600186	-4.507158289	6.57E-06	6.90E-05
ENSMUSG00000039713	18.52891564	-2.819032876	0.743835878	-3.789858702	0.000150733	0.001169098
ENSMUSG00000084883	19.92570791	-2.818803743	0.708776451	-3.976999714	6.98E-05	0.000590053
ENSMUSG00000019942	248.2133329	-2.814110144	0.307442563	-9.153287407	5.52E-20	3.60E-18
ENSMUSG00000085006	39.87500601	-2.799657917	0.562414817	-4.977923469	6.43E-07	8.19E-06
ENSMUSG00000092269	16.59147993	-2.797643998	0.724549556	-3.861218288	0.000112823	0.000902621
ENSMUSG00000004814	5.720201926	-2.778402869	0.872725744	-3.183592196	0.001454598	0.008355098
ENSMUSG00000086564	17.75144788	-2.772025933	0.702304473	-3.947042971	7.91E-05	0.000657594
ENSMUSG00000010307	51.98080134	-2.76967498	0.492744013	-5.620920619	1.90E-08	3.15E-07
ENSMUSG00000003779	100.87066	-2.767469316	0.392572856	-7.049568699	1.79E-12	5.21E-11
ENSMUSG00000061615	75.92359013	-2.763898508	0.429024854	-6.442280628	1.18E-10	2.75E-09
ENSMUSG00000061878	18.15396998	-2.75861478	0.706173263	-3.906427675	9.37E-05	0.000763232
ENSMUSG00000060678	319.9110121	-2.754243477	0.333880175	-8.249197419	1.59E-16	7.51E-15
ENSMUSG00000011148	469.6244405	-2.754066746	0.273265023	-10.07837271	6.89E-24	6.55E-22
ENSMUSG00000063021	70.93040849	-2.753080008	0.487452405	-5.647895017	1.62E-08	2.73E-07
ENSMUSG00000017466	223.2849108	-2.752176731	0.320550537	-8.585781074	9.02E-18	4.93E-16
ENSMUSG00000021127	10.78210758	-2.750049992	0.798219767	-3.44522913	0.000570576	0.003720141
ENSMUSG00000022421	13.98665327	-2.747506805	0.755635478	-3.636021449	0.000276881	0.001977535
ENSMUSG00000023940	72.02616877	-2.747362481	0.436756613	-6.290374088	3.17E-10	6.90E-09
ENSMUSG00000022793	35.63060006	-2.747207811	0.564162394	-4.869533739	1.12E-06	1.37E-05
ENSMUSG00000027765	255.9645819	-2.747113594	0.308176733	-8.914085009	4.92E-19	2.96E-17
ENSMUSG00000034265	27.78367444	-2.745934694	0.615454716	-4.46163564	8.13E-06	8.38E-05
ENSMUSG00000025574	39.7838576	-2.744443695	0.536991637	-5.110775486	3.21E-07	4.38E-06
ENSMUSG00000040710	615.8446641	-2.742792848	0.291110804	-9.421817427	4.43E-21	3.25E-19
ENSMUSG00000034883	15.59921799	-2.738938493	0.724906549	-3.778333219	0.000157882	0.001217651
ENSMUSG00000028463	5.5044294	-2.738476511	0.8734755	-3.135149718	0.001717	0.00959

	75		14		664	2742
ENSMUSG00000054871	203.72018<br>47	-2.736860771	0.3443696<br>39	-7.947450819	1.90E-15	8.05E-14
ENSMUSG00000028873	102.57996<br>16	-2.735274188	0.4017026<br>5	-6.809201257	9.81E-12	2.63E-10
ENSMUSG00000072437	38.257383<br>02	-2.728255594	0.5512958<br>89	-4.948804532	7.47E-07	9.35E-06
ENSMUSG00000026944	42.149819<br>94	-2.727108037	0.5334185<br>39	-5.112510793	3.18E-07	4.36E-06
ENSMUSG00000027961	26.719339<br>48	-2.723629844	0.6457587<br>13	-4.217720625	2.47E-05	0.00022<br>8063
ENSMUSG00000001228	120.81808<br>57	-2.722262696	0.3785267<br>64	-7.19173109	6.40E-13	1.94E-11
ENSMUSG00000071324	36.715986<br>89	-2.719767589	0.5573084<br>44	-4.880183707	1.06E-06	1.30E-05
ENSMUSG00000068855	122.06053<br>19	-2.71290426	0.3680213<br>23	-7.371595309	1.69E-13	5.62E-12
ENSMUSG00000058325	219.28775<br>5	-2.710995352	0.3233135<br>63	-8.385034411	5.07E-17	2.53E-15
ENSMUSG00000021384	231.48022<br>38	-2.710302533	0.3081403<br>13	-8.795676564	1.42E-18	8.05E-17
ENSMUSG00000002055	60.890615<br>56	-2.710143965	0.4626276<br>49	-5.858153896	4.68E-09	8.56E-08
ENSMUSG00000023505	123.37783<br>25	-2.709383719	0.3750648<br>9	-7.223773244	5.06E-13	1.58E-11
ENSMUSG00000021811	143.25094<br>58	-2.70389735	0.3483711<br>95	-7.761541106	8.39E-15	3.29E-13
ENSMUSG00000026605	131.34803<br>11	-2.697990496	0.4096838<br>38	-6.585542907	4.53E-11	1.14E-09
ENSMUSG00000084133	5.5379686<br>21	-2.697381934	0.8746051<br>87	-3.084113809	0.002041<br>595	0.01106<br>1129
ENSMUSG00000035273	23.836178<br>42	-2.695997498	0.6455231<br>39	-4.176453694	2.96E-05	0.00026<br>9987
ENSMUSG00000028678	55.912323<br>71	-2.690110676	0.4883392<br>39	-5.508692447	3.62E-08	5.73E-07
ENSMUSG00000099162	5.2886570<br>25	-2.683680502	0.8743999<br>82	-3.069168066	0.002146<br>558	0.01152<br>7656
ENSMUSG00000026069	92.755941<br>07	-2.677527428	0.4069011<br>29	-6.580289992	4.70E-11	1.17E-09
ENSMUSG00000017861	53.820147<br>66	-2.662573482	0.4804326<br>19	-5.542033108	2.99E-08	4.83E-07
ENSMUSG00000041220	68.397549<br>26	-2.662146587	0.4573424<br>3	-5.820904451	5.85E-09	1.06E-07
ENSMUSG00000089665	34.562185<br>09	-2.662096331	0.5914723<br>86	-4.50079563	6.77E-06	7.09E-05
ENSMUSG00000001436	61.204103<br>06	-2.660893371	0.5134739<br>5	-5.182138973	2.19E-07	3.10E-06
ENSMUSG00000031004	236.55410<br>5	-2.641895092	0.3131454<br>71	-8.43663835	3.27E-17	1.70E-15
ENSMUSG00000012443	244.56149<br>67	-2.640229464	0.3317669<br>38	-7.958084906	1.75E-15	7.51E-14
ENSMUSG00000005824	5.1640012<br>27	-2.638418757	0.8748807<br>87	-3.015746597	0.002563<br>474	0.01342<br>4689
ENSMUSG00000087060	19.797565	-2.632645294	0.6822495	-3.858771714	0.000113	0.00091

	74		57		958	067
ENSMUSG00000032012	54.8980737	-2.630011102	0.487956036	-5.389852589	7.05E-08	1.08E-06
ENSMUSG00000017499	50.22917907	-2.629236405	0.529653784	-4.964066119	6.90E-07	8.70E-06
ENSMUSG00000023990	56.52560205	-2.628604024	0.473297339	-5.553811116	2.80E-08	4.54E-07
ENSMUSG00000013089	99.34543539	-2.621501442	0.414330733	-6.327074565	2.50E-10	5.55E-09
ENSMUSG00000079553	7.021361203	-2.617622995	0.85578781	-3.058729004	0.002222781	0.011853392
ENSMUSG00000027330	43.78745299	-2.617124326	0.515915333	-5.072778725	3.92E-07	5.23E-06
ENSMUSG00000030465	63.0293902	-2.608976732	0.464289056	-5.619294056	1.92E-08	3.17E-07
ENSMUSG00000037235	192.717755	-2.608614568	0.321550939	-8.1126013	4.95E-16	2.27E-14
ENSMUSG00000074743	46.82928739	-2.605796145	0.512246298	-5.086998488	3.64E-07	4.89E-06
ENSMUSG00000052336	14.71912456	-2.604562976	0.737749771	-3.53041516	0.000414908	0.002824352
ENSMUSG00000032750	8.248407452	-2.603746702	0.836928517	-3.111074184	0.001864081	0.010262253
ENSMUSG00000020396	37.46543004	-2.596618953	0.543584048	-4.776849069	1.78E-06	2.10E-05
ENSMUSG00000042708	24.00388444	-2.596027203	0.646578658	-4.015021481	5.94E-05	0.000510217
ENSMUSG00000070390	16.36071209	-2.591532443	0.721311558	-3.592805929	0.000327136	0.002288618
ENSMUSG00000074212	311.6437927	-2.588334137	0.291608772	-8.876050315	6.93E-19	4.06E-17
ENSMUSG00000071478	82.18606786	-2.58039668	0.422523648	-6.107105939	1.01E-09	2.06E-08
ENSMUSG00000024579	36.25943566	-2.579009256	0.55150461	-4.676314954	2.92E-06	3.30E-05
ENSMUSG00000040950	52.53050769	-2.569939352	0.491074767	-5.23329547	1.67E-07	2.40E-06
ENSMUSG00000021696	111.6824548	-2.569452158	0.376003428	-6.833587048	8.28E-12	2.23E-10
ENSMUSG00000027496	142.1061075	-2.56649529	0.347923711	-7.376603568	1.62E-13	5.43E-12
ENSMUSG00000050410	54.84906078	-2.565971774	0.525471741	-4.883177483	1.04E-06	1.28E-05
ENSMUSG00000058099	168.0575523	-2.562239937	0.336012407	-7.62543253	2.43E-14	9.03E-13
ENSMUSG00000024989	73.69791313	-2.551534911	0.436074039	-5.85115068	4.88E-09	8.89E-08
ENSMUSG00000035615	6.896705406	-2.550413377	0.858786028	-2.969789091	0.002980043	0.015262139
ENSMUSG00000022150	264.1266561	-2.545586791	0.302652442	-8.410924334	4.07E-17	2.05E-15
ENSMUSG00000069266	55.58247857	-2.54526066	0.489399997	-5.200777845	1.98E-07	2.82E-06
ENSMUSG00000061482	477.75020	-2.536538136	0.3235617	-7.839426287	4.53E-15	1.85E-13

	07		05			
ENSMUSG00000037661	80.7388163	-2.533257264	0.435844345	-5.812298109	6.16E-09	1.11E-07
ENSMUSG00000057132	9.130509112	-2.523311966	0.825854507	-3.055395284	0.002247641	0.011958042
ENSMUSG00000055805	77.2474137	-2.521887607	0.429973628	-5.865214615	4.49E-09	8.26E-08
ENSMUSG00000015852	10.67646365	-2.51805382	0.804812969	-3.128744091	0.001755551	0.00976058
ENSMUSG00000008318	26.49808272	-2.512766723	0.633403465	-3.967087111	7.28E-05	0.000612486
ENSMUSG00000050538	42.43116078	-2.510870908	0.545216188	-4.605275785	4.12E-06	4.51E-05
ENSMUSG00000050232	12.79920264	-2.5088308	0.767966287	-3.266850175	0.001087512	0.006531268
ENSMUSG00000070348	212.2211152	-2.507601435	0.31512338	-7.957522646	1.76E-15	7.52E-14
ENSMUSG00000039109	7.596573761	-2.507073174	0.844885828	-2.967351436	0.003003774	0.015354988
ENSMUSG00000047250	141.1238764	-2.506261068	0.351251899	-7.135224253	9.66E-13	2.87E-11
ENSMUSG00000022322	59.06025832	-2.505178013	0.502661817	-4.983823968	6.23E-07	7.97E-06
ENSMUSG00000027339	340.6768601	-2.505021095	0.337664526	-7.418668249	1.18E-13	4.01E-12
ENSMUSG00000070577	46.0484071	-2.503875033	0.52597591	-4.760436718	1.93E-06	2.27E-05
ENSMUSG00000100280	22.5530948	-2.502874664	0.677242165	-3.695686408	0.000219294	0.001618426
ENSMUSG00000072618	14.73362008	-2.502548116	0.732605775	-3.415954668	0.000635588	0.004087629
ENSMUSG00000022912	135.016054	-2.501839711	0.355617123	-7.035205982	1.99E-12	5.74E-11
ENSMUSG00000037787	36.53479385	-2.499549419	0.557509967	-4.483416562	7.35E-06	7.63E-05
ENSMUSG00000034023	59.12440246	-2.496522577	0.459845838	-5.429042456	5.67E-08	8.79E-07
ENSMUSG00000032218	217.1313187	-2.493072132	0.30839234	-8.08409228	6.26E-16	2.83E-14
ENSMUSG00000052364	25.30202545	-2.492767683	0.622385089	-4.005185419	6.20E-05	0.000529148
ENSMUSG00000017550	69.70482701	-2.489986683	0.4349379	-5.724924604	1.03E-08	1.79E-07
ENSMUSG00000050103	59.87434549	-2.488406385	0.466473383	-5.33450884	9.58E-08	1.44E-06
ENSMUSG00000055538	15.27281715	-2.487224051	0.734197199	-3.38767848	0.000704868	0.004472325
ENSMUSG00000026932	128.1084871	-2.486489026	0.391882095	-6.344992688	2.22E-10	4.97E-09
ENSMUSG00000045092	162.142598	-2.483577182	0.338416431	-7.338819735	2.15E-13	7.10E-12
ENSMUSG00000056004	107.2228804	-2.483552569	0.380262906	-6.531146034	6.53E-11	1.58E-09
ENSMUSG00000041498	50.947059	-2.479292243	0.4835648	-5.12711403	2.94E-07	4.07E-06

	54		73			
ENSMUSG00000020150	36.75913088	-2.462496591	0.570203435	-4.318628127	1.57E-05	0.000151316
ENSMUSG00000043391	6.249388055	-2.460016582	0.862448522	-2.852363381	0.004339546	0.020913794
ENSMUSG00000071547	106.9481261	-2.453179637	0.37962311	-6.462145141	1.03E-10	2.43E-09
ENSMUSG00000027242	38.68353738	-2.448962515	0.538173115	-4.550510696	5.35E-06	5.71E-05
ENSMUSG00000018341	33.96755457	-2.447178642	0.566181836	-4.32224859	1.54E-05	0.000149273
ENSMUSG00000034614	42.87579689	-2.438410956	0.519033829	-4.697980786	2.63E-06	3.00E-05
ENSMUSG00000026308	11.67026572	-2.434711131	0.771658933	-3.155164836	0.001604075	0.00908048
ENSMUSG00000036611	451.3191592	-2.434437522	0.280339445	-8.683892221	3.82E-18	2.12E-16
ENSMUSG00000047409	11.5290744	-2.432270181	0.773465127	-3.144641041	0.001662907	0.009344053
ENSMUSG00000027306	94.68161743	-2.429549246	0.411774142	-5.900198666	3.63E-09	6.84E-08
ENSMUSG00000002329	39.58720106	-2.42416776	0.547325197	-4.429117777	9.46E-06	9.60E-05
ENSMUSG00000074622	215.304879	-2.422820354	0.390616496	-6.20255513	5.56E-10	1.18E-08
ENSMUSG00000072115	38.24590424	-2.421404042	0.548001388	-4.41860932	9.93E-06	0.000100105
ENSMUSG00000040084	244.6260135	-2.41315951	0.328629837	-7.343093161	2.09E-13	6.89E-12
ENSMUSG00000022385	22.52855656	-2.412625095	0.64747047	-3.72623186	0.000194364	0.001461994
ENSMUSG00000022177	117.9801794	-2.408532575	0.371242852	-6.487754744	8.71E-11	2.07E-09
ENSMUSG00000039959	333.9411958	-2.40763747	0.287833081	-8.364700331	6.03E-17	2.99E-15
ENSMUSG00000039485	19.58129342	-2.405854865	0.67259814	-3.576957355	0.000347617	0.002409757
ENSMUSG00000031562	54.07605612	-2.398172551	0.473643524	-5.063243617	4.12E-07	5.47E-06
ENSMUSG00000069308	16.00823289	-2.39633162	0.714672327	-3.353049403	0.000799264	0.004981318
ENSMUSG00000027546	80.64128942	-2.392332353	0.437577662	-5.467217737	4.57E-08	7.18E-07
ENSMUSG00000029438	17.99279883	-2.388821833	0.748285261	-3.19239461	0.001410984	0.008155858
ENSMUSG00000034773	7.193583563	-2.386633504	0.844097151	-2.827439356	0.00469219	0.022328634
ENSMUSG00000078122	24.76483661	-2.385270587	0.638248684	-3.737211916	0.000186072	0.001406574
ENSMUSG00000005470	101.3165962	-2.384571346	0.40043073	-5.955015849	2.60E-09	4.99E-08
ENSMUSG00000038390	191.7612987	-2.383577816	0.32547062	-7.323480737	2.42E-13	7.87E-12
ENSMUSG00000021822	2335.7941	-2.382129371	0.2209328	-10.78214164	4.18E-27	5.21E-25

	36		58			
ENSMUSG00000014778	63.23974034	-2.379086859	0.465418166	-5.111718948	3.19E-07	4.37E-06
ENSMUSG00000068877	24.960661	-2.366310271	0.632714289	-3.739934929	0.000184068	0.00139373
ENSMUSG00000026458	5.86340148	-2.364321911	0.86619492	-2.729549501	0.006342093	0.028704881
ENSMUSG00000036565	36.63397521	-2.357639515	0.556245715	-4.238485709	2.25E-05	0.000209801
ENSMUSG00000037224	38.68645005	-2.356025922	0.554431939	-4.249441199	2.14E-05	0.000200618
ENSMUSG00000021697	26.88011482	-2.348085791	0.633900914	-3.704184267	0.000212072	0.001572753
ENSMUSG00000026807	27.68763535	-2.345217905	0.621936508	-3.770831708	0.000162704	0.001252029
ENSMUSG00000034413	7.641663985	-2.34259519	0.846345419	-2.767894926	0.005641964	0.026025958
ENSMUSG00000030789	120.297701	-2.338283394	0.372912307	-6.270330451	3.60E-10	7.78E-09
ENSMUSG00000040616	48.63770673	-2.338166276	0.503465366	-4.644145229	3.41E-06	3.82E-05
ENSMUSG00000035283	15.26233811	-2.336786523	0.722664335	-3.23357112	0.001222529	0.007212552
ENSMUSG00000001761	7.380333208	-2.324496135	0.847616459	-2.742391456	0.00609936	0.02778714
ENSMUSG00000036533	206.1148713	-2.323077121	0.348469786	-6.666509453	2.62E-11	6.70E-10
ENSMUSG00000029162	62.72561159	-2.321702364	0.456452931	-5.086400385	3.65E-07	4.90E-06
ENSMUSG00000040913	176.507173	-2.31743603	0.357671886	-6.479223331	9.22E-11	2.17E-09
ENSMUSG00000030041	65.68489392	-2.313066825	0.458098027	-5.049283537	4.43E-07	5.85E-06
ENSMUSG00000022265	331.864884	-2.311455007	0.291089342	-7.940706409	2.01E-15	8.43E-14
ENSMUSG00000089715	111.292222	-2.310233581	0.382970902	-6.032399776	1.62E-09	3.19E-08
ENSMUSG00000022422	51.37120566	-2.308031374	0.490731536	-4.703246491	2.56E-06	2.93E-05
ENSMUSG00000049709	24.97763285	-2.304541394	0.614235183	-3.751887643	0.000175508	0.001335557
ENSMUSG00000045328	127.7425608	-2.293488538	0.36281428	-6.321384422	2.59E-10	5.73E-09
ENSMUSG00000005233	126.9851149	-2.292709119	0.367688577	-6.23546463	4.50E-10	9.65E-09
ENSMUSG00000054200	11.93360473	-2.292364524	0.762537532	-3.006231731	0.002645073	0.013804543
ENSMUSG00000044734	483.974385	-2.291514214	0.328886241	-6.967497968	3.23E-12	9.10E-11
ENSMUSG00000027115	46.31612509	-2.291197363	0.530829371	-4.316259586	1.59E-05	0.00015284
ENSMUSG00000052609	17.82157467	-2.289568841	0.680803147	-3.363040919	0.000770889	0.004837472
ENSMUSG00000022102	195.29192	-2.283422868	0.3210506	-7.11234532	1.14E-12	3.36E-11

	17		19			
ENSMUSG00000053716	139.6236448	-2.28068712	0.348524611	-6.543833765	6.00E-11	1.47E-09
ENSMUSG00000022861	115.3879958	-2.280585325	0.375847126	-6.067853565	1.30E-09	2.60E-08
ENSMUSG00000033032	36.66995893	-2.275125339	0.548004124	-4.151657329	3.30E-05	0.000298988
ENSMUSG00000091405	243.8383272	-2.274767852	0.373758587	-6.086195563	1.16E-09	2.33E-08
ENSMUSG00000081219	8.899741274	-2.268464246	0.812170745	-2.793087858	0.005220751	0.024436609
ENSMUSG00000004698	208.5826057	-2.266326628	0.313847266	-7.221113172	5.16E-13	1.60E-11
ENSMUSG00000040855	199.0234659	-2.266193098	0.317776574	-7.131403895	9.94E-13	2.95E-11
ENSMUSG00000026688	698.7492675	-2.255488353	0.253428636	-8.899895407	5.59E-19	3.32E-17
ENSMUSG00000032245	24.57464265	-2.252356337	0.616009458	-3.656366485	0.000255816	0.001845516
ENSMUSG00000037868	21.01564131	-2.24976536	0.649875955	-3.461838127	0.0005365	0.003526489
ENSMUSG00000001482	134.9684253	-2.249110258	0.37086379	-6.064518351	1.32E-09	2.64E-08
ENSMUSG00000059479	26.01000057	-2.245058796	0.625532619	-3.589035535	0.000331904	0.002313702
ENSMUSG00000036526	15.91865637	-2.244726842	0.703545889	-3.190590518	0.001419824	0.00819658
ENSMUSG00000038740	108.2334778	-2.239984008	0.392287527	-5.710056663	1.13E-08	1.94E-07
ENSMUSG00000026975	31.24908127	-2.23853464	0.581831248	-3.847395007	0.00011938	0.000949019
ENSMUSG00000026833	193.8928362	-2.233088935	0.339133481	-6.584690273	4.56E-11	1.14E-09
ENSMUSG00000032782	57.08554894	-2.232298728	0.513831317	-4.344419375	1.40E-05	0.000136604
ENSMUSG00000034275	19.22790979	-2.229777478	0.702122572	-3.175766694	0.001494411	0.008537266
ENSMUSG00000097283	10.94632723	-2.229521996	0.784821763	-2.840800421	0.004500047	0.021586677
ENSMUSG00000051378	10.2629944	-2.229226468	0.787809976	-2.829649962	0.004659896	0.02221088
ENSMUSG00000022123	8.638410497	-2.228292416	0.818762518	-2.721536913	0.006497913	0.029245942
ENSMUSG00000025328	5.352759108	-2.22794355	0.869977359	-2.5609213	0.0104395	0.042977876
ENSMUSG00000050592	52.54677294	-2.223024416	0.517986802	-4.291662275	1.77E-05	0.000168899
ENSMUSG00000026712	680.2460353	-2.216710079	0.266843997	-8.307138646	9.80E-17	4.71E-15
ENSMUSG00000039157	60.44979793	-2.211579791	0.477014365	-4.636295994	3.55E-06	3.95E-05
ENSMUSG00000038026	5.43185658	-2.21059091	0.871890178	-2.535400634	0.011231879	0.045461577
ENSMUSG00000051517	7.3993450	-2.207667248	0.8383802	-2.633252819	0.008457	0.03629

	68		85		137	9737
ENSMUSG00000046295	12.22800655	-2.207011465	0.774501782	-2.849588622	0.004377581	0.021065571
ENSMUSG00000031016	17.55977579	-2.204082112	0.691024647	-3.189585379	0.00142477	0.008221675
ENSMUSG00000046329	14.38224468	-2.198848706	0.738643659	-2.976873461	0.002912042	0.014957187
ENSMUSG00000041859	485.2158925	-2.197499898	0.278368407	-7.894214438	2.92E-15	1.21E-13
ENSMUSG00000032788	524.7012283	-2.19567559	0.288846986	-7.601518093	2.93E-14	1.08E-12
ENSMUSG00000062075	52.72351164	-2.195442539	0.517816177	-4.239810642	2.24E-05	0.000208708
ENSMUSG00000017713	25.3982306	-2.18915912	0.611606763	-3.579357278	0.00034444	0.002390154
ENSMUSG00000060380	11.39393955	-2.187096107	0.771796923	-2.833771479	0.004600222	0.021974917
ENSMUSG00000014361	155.5618416	-2.184325269	0.35365768	-6.176382964	6.56E-10	1.38E-08
ENSMUSG00000097318	12.32159954	-2.184169763	0.755296896	-2.891802913	0.003830381	0.018839192
ENSMUSG00000044551	20.02541937	-2.183093778	0.677757535	-3.221054234	0.0012772	0.007489954
ENSMUSG00000029650	177.2051873	-2.178912497	0.354398939	-6.148191363	7.84E-10	1.62E-08
ENSMUSG00000024535	272.5489238	-2.177647498	0.289902569	-7.511652993	5.84E-14	2.07E-12
ENSMUSG00000051627	346.7574446	-2.173047193	0.360924581	-6.020779148	1.74E-09	3.41E-08
ENSMUSG00000106820	9.922566151	-2.172422346	0.791954582	-2.743114813	0.00608594	0.027756343
ENSMUSG00000055322	14.72063293	-2.171696938	0.732926861	-2.963047276	0.003046098	0.015538195
ENSMUSG00000020053	513.3181518	-2.171578413	0.319836826	-6.789644707	1.12E-11	3.00E-10
ENSMUSG00000034957	388.4206345	-2.168244403	0.301774906	-7.184972505	6.72E-13	2.04E-11
ENSMUSG00000034127	8.297982249	-2.167989377	0.822293273	-2.636516007	0.008376225	0.036004875
ENSMUSG00000056498	414.1984881	-2.167589453	0.27263629	-7.950480292	1.86E-15	7.88E-14
ENSMUSG00000027698	1520.514272	-2.165218001	0.231393118	-9.357313712	8.18E-21	5.75E-19
ENSMUSG00000022673	232.7754325	-2.163954235	0.315784022	-6.852640045	7.25E-12	1.97E-10
ENSMUSG00000032122	706.852004	-2.154964509	0.253422629	-8.50344153	1.84E-17	9.78E-16
ENSMUSG00000047434	43.52130383	-2.15456814	0.530755865	-4.059433505	4.92E-05	0.000431334
ENSMUSG00000096010	275.2554665	-2.154008063	0.35027322	-6.149508272	7.77E-10	1.61E-08
ENSMUSG00000028702	41.15828593	-2.151013669	0.530395445	-4.055490461	5.00E-05	0.000437556
ENSMUSG00000041774	66.106531	-2.15039424	0.4732383	-4.543998196	5.52E-06	5.88E-05

	93		57			
ENSMUSG00000030263	531.4297328	-2.141586136	0.263958171	-8.113354194	4.92E-16	2.26E-14
ENSMUSG00000039976	295.4217132	-2.137303918	0.36358714	-5.878381504	4.14E-09	7.69E-08
ENSMUSG00000045160	45.0622001	-2.137068599	0.496754146	-4.302064944	1.69E-05	0.000161721
ENSMUSG00000024795	162.2572367	-2.136901482	0.364550492	-5.861743511	4.58E-09	8.39E-08
ENSMUSG00000020156	216.3077578	-2.136653639	0.306228742	-6.977312522	3.01E-12	8.50E-11
ENSMUSG00000040797	73.18750911	-2.129133467	0.444944927	-4.785161798	1.71E-06	2.02E-05
ENSMUSG00000007570	80.0958276	-2.126689196	0.446530703	-4.762694219	1.91E-06	2.25E-05
ENSMUSG00000038886	268.5049369	-2.125155957	0.345212727	-6.156076494	7.46E-10	1.55E-08
ENSMUSG00000050627	201.0201417	-2.125149268	0.315477024	-6.736304412	1.62E-11	4.28E-10
ENSMUSG00000044408	1258.660862	-2.124583359	0.265932673	-7.98917763	1.36E-15	5.89E-14
ENSMUSG00000021175	245.9323792	-2.122974062	0.295154746	-7.192749184	6.35E-13	1.93E-11
ENSMUSG00000027323	50.33729627	-2.120501862	0.498682853	-4.252205281	2.12E-05	0.000198564
ENSMUSG00000040907	83.3163351	-2.115760746	0.405249559	-5.220883524	1.78E-07	2.54E-06
ENSMUSG00000029475	83.25129682	-2.108449474	0.414524675	-5.08642694	3.65E-07	4.90E-06
ENSMUSG00000031860	22.39539819	-2.106181152	0.629126777	-3.347784945	0.000814602	0.005060798
ENSMUSG00000083668	31.23518968	-2.10454589	0.594605521	-3.539398499	0.00040104	0.002739907
ENSMUSG00000029047	147.991767	-2.103093283	0.413167789	-5.090167576	3.58E-07	4.83E-06
ENSMUSG00000089671	9.11752196	-2.098028704	0.8069975	-2.599795792	0.009327925	0.039250603
ENSMUSG00000030978	534.8737548	-2.097999022	0.276236476	-7.594938411	3.08E-14	1.13E-12
ENSMUSG00000028927	31.35231087	-2.092284231	0.565755589	-3.698212215	0.000217123	0.001604138
ENSMUSG00000033610	75.54944316	-2.091665369	0.420555934	-4.97357236	6.57E-07	8.36E-06
ENSMUSG00000040229	14.65257637	-2.084832416	0.722789123	-2.884426936	0.003921267	0.019213284
ENSMUSG00000026429	41.58933096	-2.083484026	0.519708748	-4.008945459	6.10E-05	0.00052177
ENSMUSG00000032402	27.79632779	-2.082222317	0.623241667	-3.340954926	0.000834908	0.005170543
ENSMUSG00000045838	9.347353594	-2.08072458	0.825635515	-2.520149075	0.011730514	0.047145659
ENSMUSG00000052384	1359.178262	-2.07697722	0.265256924	-7.830058465	4.88E-15	1.98E-13
ENSMUSG00000099655	12.726129	-2.074817309	0.7461228	-2.780798626	0.005422	0.02522

	87		19		536	6021
ENSMUSG00000030431	19.91479099	-2.074568559	0.666847837	-3.111007403	0.001864503	0.010262253
ENSMUSG00000028621	82.75220552	-2.069571076	0.427720673	-4.838604268	1.31E-06	1.57E-05
ENSMUSG00000034906	128.081781	-2.06770159	0.364374035	-5.674667757	1.39E-08	2.36E-07
ENSMUSG00000014543	19.05511373	-2.063717438	0.687570042	-3.001465033	0.002686839	0.01399055
ENSMUSG00000032254	75.05035187	-2.061470179	0.417512141	-4.937509533	7.91E-07	9.89E-06
ENSMUSG00000023909	18.20863328	-2.055693712	0.700899836	-2.932935073	0.003357741	0.01694324
ENSMUSG00000025742	252.466113	-2.054796127	0.303458522	-6.771258602	1.28E-11	3.38E-10
ENSMUSG00000048240	13.00851251	-2.053414787	0.755787888	-2.716919414	0.006589265	0.029589081
ENSMUSG00000047003	15.64782481	-2.052384386	0.743970987	-2.758688741	0.005803378	0.026636068
ENSMUSG00000064373	900.944857	-2.049426236	0.286868662	-7.144127299	9.06E-13	2.71E-11
ENSMUSG00000026039	26.89263234	-2.048992184	0.591242547	-3.46556958	0.00052911	0.003482924
ENSMUSG00000038379	42.08741222	-2.047814783	0.534641391	-3.830258595	0.000128009	0.001009412
ENSMUSG00000001517	19.93132651	-2.046664641	0.674749241	-3.03322259	0.002419571	0.012778503
ENSMUSG00000085448	45.68946379	-2.044072801	0.496837917	-4.114164263	3.89E-05	0.000345884
ENSMUSG00000027699	108.3987362	-2.042840792	0.369176314	-5.533509912	3.14E-08	5.06E-07
ENSMUSG00000047747	64.11593975	-2.04208308	0.440304011	-4.637893431	3.52E-06	3.92E-05
ENSMUSG00000081670	29.15452264	-2.039115002	0.576963496	-3.534218397	0.000408983	0.002786126
ENSMUSG00000026791	23.75818321	-2.038988112	0.629259187	-3.240299312	0.001194043	0.007065785
ENSMUSG00000028834	61.16914471	-2.037945036	0.446241835	-4.566907165	4.95E-06	5.32E-05
ENSMUSG00000025001	183.1220817	-2.037038581	0.316928331	-6.427442362	1.30E-10	3.02E-09
ENSMUSG00000026009	11.7844107	-2.034815536	0.759867446	-2.677855917	0.007409508	0.032568093
ENSMUSG00000018983	111.8913584	-2.033613436	0.409276	-4.968806953	6.74E-07	8.55E-06
ENSMUSG00000025732	41.84717537	-2.031681057	0.515634996	-3.94015354	8.14E-05	0.000675131
ENSMUSG00000000753	58.5443597	-2.03121731	0.470229531	-4.319629404	1.56E-05	0.000150737
ENSMUSG00000069272	44.72036754	-2.029429131	0.522626252	-3.883136606	0.000103118	0.00083131
ENSMUSG00000032411	62.03424275	-2.026769138	0.446364211	-4.540617477	5.61E-06	5.95E-05
ENSMUSG00000024056	88.325178	-2.024424084	0.3969482	-5.099969246	3.40E-07	4.63E-06

	06		93			
ENSMUSG00000039396	31.57551233	-2.022824584	0.57760522	-3.502088476	0.000461626	0.003092174
ENSMUSG00000027544	43.31811243	-2.021026481	0.557574062	-3.624678081	0.000289322	0.002056614
ENSMUSG00000048327	36.71711939	-2.020985917	0.530107291	-3.812409207	0.000137619	0.001077136
ENSMUSG00000032047	416.0122527	-2.016966971	0.275693447	-7.315977198	2.56E-13	8.24E-12
ENSMUSG00000040102	81.38339426	-2.011875697	0.411130151	-4.893525064	9.90E-07	1.22E-05
ENSMUSG00000054426	10.73209492	-2.011796137	0.772555563	-2.604079543	0.009212137	0.038882767
ENSMUSG00000005043	25.56951676	-2.008768353	0.638980012	-3.143710781	0.001668202	0.009366125
ENSMUSG00000026238	230.1357213	-2.00862489	0.316468416	-6.346999547	2.20E-10	4.92E-09
ENSMUSG00000055612	283.6340264	-2.005362854	0.28513843	-7.032944852	2.02E-12	5.82E-11
ENSMUSG00000000253	272.7327909	-2.004537121	0.295744485	-6.777935766	1.22E-11	3.24E-10
ENSMUSG00000074604	37.37390575	-2.003940027	0.539667765	-3.71328465	0.000204587	0.001528822
ENSMUSG00000072568	177.2118085	-2.00267787	0.365427274	-5.480373281	4.24E-08	6.69E-07
ENSMUSG00000013483	60.89439045	-1.99294989	0.447940891	-4.449135876	8.62E-06	8.82E-05
ENSMUSG00000025766	73.23550018	-1.990549604	0.42401056	-4.694575546	2.67E-06	3.04E-05
ENSMUSG00000030091	70.42153124	-1.982665846	0.42890485	-4.622623986	3.79E-06	4.19E-05
ENSMUSG00000036896	401.5159095	-1.978407578	0.308107822	-6.421153362	1.35E-10	3.14E-09
ENSMUSG00000026196	33.04300509	-1.976973839	0.560561396	-3.526774858	0.000420654	0.002852581
ENSMUSG00000004105	128.0379407	-1.976736009	0.390949838	-5.056239493	4.28E-07	5.66E-06
ENSMUSG00000030316	55.69929618	-1.974513013	0.461984747	-4.273978796	1.92E-05	0.000181613
ENSMUSG00000042082	89.74001422	-1.971047823	0.392221472	-5.025344	5.03E-07	6.54E-06
ENSMUSG00000002835	101.4835608	-1.969233975	0.390489034	-5.042994305	4.58E-07	6.03E-06
ENSMUSG00000033446	78.73289338	-1.964602955	0.447593711	-4.389255044	1.14E-05	0.000113781
ENSMUSG00000037725	41.04956019	-1.962577206	0.529413706	-3.707076683	0.000209665	0.001560474
ENSMUSG00000036353	34.12132541	-1.962388247	0.551457591	-3.558547892	0.000372911	0.00255921
ENSMUSG00000034194	12.97045702	-1.961925301	0.770205127	-2.547276346	0.010856742	0.044296413
ENSMUSG00000034917	11.19219452	-1.957080964	0.776139784	-2.521557334	0.011683663	0.047019419
ENSMUSG00000097908	13.653321	-1.953676365	0.7532745	-2.593577968	0.009498	0.03982

	76		84		3	0802
ENSMUSG00000035683	40.40884124	-1.953259486	0.512083788	-3.814335726	0.00013655	0.001069379
ENSMUSG00000050947	19.57884732	-1.952308227	0.653017144	-2.989673772	0.002792755	0.014454674
ENSMUSG00000020593	90.18264209	-1.949340129	0.390534937	-4.991461573	5.99E-07	7.68E-06
ENSMUSG00000033065	128.2531077	-1.946759777	0.357618623	-5.443675619	5.22E-08	8.11E-07
ENSMUSG00000097729	29.66466515	-1.946645853	0.575697347	-3.3813702	0.000721253	0.004563607
ENSMUSG00000026126	406.5084309	-1.945345241	0.267189666	-7.280765284	3.32E-13	1.05E-11
ENSMUSG00000060261	187.9114495	-1.942344685	0.322801145	-6.017155505	1.78E-09	3.48E-08
ENSMUSG00000035365	30.24687038	-1.938662359	0.574967338	-3.371778242	0.000746846	0.004703823
ENSMUSG00000042073	26.81959134	-1.937776395	0.600135559	-3.228897817	0.001242683	0.007306792
ENSMUSG00000072407	57.09554652	-1.935308144	0.4741632	-4.081523291	4.47E-05	0.000394837
ENSMUSG00000071604	78.59988266	-1.928159511	0.421136264	-4.57846943	4.68E-06	5.06E-05
ENSMUSG00000046768	35.65522899	-1.926825451	0.545967597	-3.529193784	0.000416828	0.002833643
ENSMUSG00000031403	238.4127844	-1.926001188	0.31524392	-6.109558562	9.99E-10	2.03E-08
ENSMUSG00000034487	123.8958976	-1.924429595	0.371976102	-5.173530192	2.30E-07	3.24E-06
ENSMUSG00000074505	254.8683276	-1.921319081	0.297904853	-6.449438671	1.12E-10	2.63E-09
ENSMUSG00000040945	786.9306557	-1.919571469	0.295005694	-6.506896329	7.67E-11	1.84E-09
ENSMUSG00000051220	92.9512203	-1.919272879	0.392486682	-4.890033133	1.01E-06	1.24E-05
ENSMUSG00000022667	436.9270319	-1.915043015	0.267755005	-7.152221177	8.54E-13	2.57E-11
ENSMUSG00000026809	44.0811149	-1.911508096	0.533872766	-3.58045628	0.000342995	0.002383891
ENSMUSG00000039691	22.87109711	-1.909863724	0.679440583	-2.810935602	0.004939767	0.023312503
ENSMUSG00000069516	13310.47217	-1.909014166	0.236812631	-8.061285236	7.55E-16	3.37E-14
ENSMUSG00000025153	557.526824	-1.908822778	0.265200783	-7.197651373	6.13E-13	1.87E-11
ENSMUSG00000069515	12.10984356	-1.904946794	0.750724487	-2.5374779	0.011165442	0.045254607
ENSMUSG00000038351	25.08237082	-1.904320376	0.603102333	-3.157541053	0.001591058	0.009014243
ENSMUSG00000020160	34.77265923	-1.903289707	0.540153617	-3.52360819	0.000425713	0.002881185
ENSMUSG00000030207	58.01535145	-1.902052179	0.501467761	-3.792970014	0.000148856	0.001155849
ENSMUSG00000055884	95.489373	-1.901691836	0.3816766	-4.982468854	6.28E-07	8.02E-06

	24		13			
ENSMUSG00000038534	33.17167736	-1.900757148	0.552860508	-3.438041099	0.000585939	0.003804022
ENSMUSG00000038732	261.1379974	-1.893976069	0.290499978	-6.519711562	7.04E-11	1.70E-09
ENSMUSG00000042213	45.91437298	-1.893209823	0.490212356	-3.862019795	0.000112453	0.000900219
ENSMUSG00000032741	236.8372799	-1.893081833	0.299037816	-6.3305767	2.44E-10	5.43E-09
ENSMUSG00000047875	144.7888204	-1.892298186	0.369670161	-5.11888268	3.07E-07	4.24E-06
ENSMUSG00000025375	87.6575486	-1.891818179	0.408629711	-4.629663794	3.66E-06	4.06E-05
ENSMUSG00000025464	17.81012765	-1.891186088	0.685129829	-2.760332431	0.005774257	0.026530091
ENSMUSG00000061607	43.42724121	-1.890848946	0.512784482	-3.687414525	0.000226544	0.001664765
ENSMUSG00000021714	32.24943815	-1.886857402	0.561983377	-3.357496821	0.000786516	0.004926512
ENSMUSG00000006221	121.7924953	-1.88682491	0.36797129	-5.127641643	2.93E-07	4.06E-06
ENSMUSG00000028068	89.08839018	-1.886375332	0.426621052	-4.421664899	9.79E-06	9.89E-05
ENSMUSG00000085711	50.41957807	-1.881996316	0.476604845	-3.948756161	7.86E-05	0.000653343
ENSMUSG00000048439	42.31530791	-1.880002762	0.506990918	-3.708158661	0.000208772	0.00155501
ENSMUSG00000031971	22.77040585	-1.876952095	0.62972881	-2.980572057	0.002877105	0.014822567
ENSMUSG00000029163	151.334617	-1.875169454	0.353493249	-5.304682512	1.13E-07	1.68E-06
ENSMUSG00000037474	119.8603969	-1.87455932	0.36759818	-5.099479331	3.41E-07	4.63E-06
ENSMUSG00000025395	108.3151828	-1.873630605	0.366042395	-5.118616392	3.08E-07	4.24E-06
ENSMUSG00000017417	27.68873761	-1.873232513	0.610206393	-3.069834293	0.002141776	0.011510994
ENSMUSG00000021108	1039.687785	-1.871027424	0.23923065	-7.821018844	5.24E-15	2.11E-13
ENSMUSG00000047379	85.82415316	-1.865349724	0.395030884	-4.722035162	2.33E-06	2.70E-05
ENSMUSG00000083679	27.3272575	-1.865320598	0.598194174	-3.118252698	0.001819267	0.010069895
ENSMUSG00000026389	137.0008448	-1.864089278	0.358724643	-5.196434966	2.03E-07	2.88E-06
ENSMUSG00000048120	307.8332794	-1.862851397	0.284680402	-6.543658732	6.00E-11	1.47E-09
ENSMUSG00000032477	81.8689867	-1.860092018	0.416203603	-4.469187686	7.85E-06	8.10E-05
ENSMUSG00000026047	45.84022818	-1.859927999	0.490144104	-3.794655458	0.000147849	0.001151287
ENSMUSG00000070565	200.1463503	-1.844726068	0.333764719	-5.527025357	3.26E-08	5.23E-07
ENSMUSG00000022070	34.434239	-1.842668636	0.5406156	-3.408463263	0.000653	0.00418

	22		66		299	1223
ENSMUSG00000035877	66.04557058	-1.842209402	0.445376749	-4.136294515	3.53E-05	0.000317617
ENSMUSG00000070283	62.8905377	-1.842084657	0.462792576	-3.980367778	6.88E-05	0.000582473
ENSMUSG00000107355	30.88481248	-1.840756075	0.591968148	-3.10955257	0.001873709	0.010292259
ENSMUSG00000025511	321.8498457	-1.835643474	0.317924235	-5.7738394	7.75E-09	1.38E-07
ENSMUSG00000024026	114.0276537	-1.835092126	0.375220428	-4.890704213	1.00E-06	1.24E-05
ENSMUSG00000036223	28.04566961	-1.834019175	0.583255071	-3.144454746	0.001663966	0.009346172
ENSMUSG00000048307	71.9935626	-1.832417491	0.428043684	-4.280912347	1.86E-05	0.000176532
ENSMUSG00000071654	84.14301208	-1.830436125	0.449311448	-4.073869321	4.62E-05	0.000406477
ENSMUSG00000038252	94.50380347	-1.828931806	0.394979406	-4.630448515	3.65E-06	4.05E-05
ENSMUSG00000029710	20.35888517	-1.826248261	0.647101415	-2.82219791	0.004769573	0.022626169
ENSMUSG00000024177	77.48212249	-1.824277778	0.42113726	-4.331789062	1.48E-05	0.000143657
ENSMUSG00000044066	76.00670085	-1.823273329	0.424231624	-4.297825115	1.72E-05	0.000164615
ENSMUSG00000063894	22.06052654	-1.820889975	0.629593266	-2.89216876	0.003825924	0.018839192
ENSMUSG00000029516	26.04353818	-1.820803199	0.60914057	-2.989134674	0.002797688	0.014468797
ENSMUSG00000027955	107.2915155	-1.817387838	0.376729524	-4.824118421	1.41E-06	1.69E-05
ENSMUSG00000039063	117.9155942	-1.815417075	0.364746532	-4.977201748	6.45E-07	8.22E-06
ENSMUSG00000060981	353.2583585	-1.812440173	0.310076197	-5.845144479	5.06E-09	9.16E-08
ENSMUSG00000068854	161.331251	-1.810462936	0.324749634	-5.574949889	2.48E-08	4.06E-07
ENSMUSG00000044788	14.29916097	-1.810313738	0.712997745	-2.539017479	0.011116427	0.045114193
ENSMUSG00000001270	140.0221774	-1.810298355	0.340443507	-5.317470642	1.05E-07	1.57E-06
ENSMUSG00000058392	103.9016665	-1.810076355	0.370536501	-4.885014976	1.03E-06	1.27E-05
ENSMUSG00000039253	17.15829396	-1.805656682	0.704544297	-2.562871759	0.010381038	0.042788611
ENSMUSG00000041594	27.11891407	-1.805631997	0.59204052	-3.049845299	0.002289593	0.012153483
ENSMUSG00000024986	84.50698846	-1.800965316	0.400366839	-4.498287925	6.85E-06	7.16E-05
ENSMUSG00000059498	2270.414264	-1.800635979	0.23233632	-7.750126958	9.18E-15	3.58E-13
ENSMUSG00000024791	93.19420515	-1.800280018	0.412005437	-4.369554029	1.25E-05	0.000123197
ENSMUSG00000026866	26.983810	-1.796696817	0.6044335	-2.97253017	0.002953	0.01514

	99		01		562	3496
ENSMUSG00000040658	60.40399956	-1.796539245	0.512029786	-3.508661591	0.000450368	0.003024149
ENSMUSG00000099583	69.08793508	-1.79560292	0.430303457	-4.17287589	3.01E-05	0.000273731
ENSMUSG00000035284	1416.767555	-1.794259035	0.236328161	-7.592235437	3.14E-14	1.15E-12
ENSMUSG00000021965	47.4728768	-1.792652116	0.482905349	-3.712222529	0.000205447	0.001532746
ENSMUSG00000035064	151.585541	-1.79155554	0.370381413	-4.837055739	1.32E-06	1.58E-05
ENSMUSG00000020988	98.29360289	-1.788809056	0.380060299	-4.70664539	2.52E-06	2.90E-05
ENSMUSG00000047264	24.95520692	-1.788572188	0.624377973	-2.864566443	0.004175805	0.020222407
ENSMUSG00000102224	34.6515518	-1.783203265	0.535801546	-3.32810399	0.000874392	0.005381026
ENSMUSG00000032586	39.93435018	-1.780707199	0.527707583	-3.374420338	0.000739713	0.004669631
ENSMUSG00000046994	131.4685281	-1.77943593	0.3869811	-4.598250219	4.26E-06	4.64E-05
ENSMUSG00000032601	178.150219	-1.777775884	0.318855343	-5.575493481	2.47E-08	4.05E-07
ENSMUSG00000045730	39.48626976	-1.777671734	0.524653547	-3.388277362	0.000703331	0.004464637
ENSMUSG00000045216	185.3670405	-1.776046668	0.340675054	-5.213315882	1.85E-07	2.64E-06
ENSMUSG00000038943	171.0995633	-1.775502684	0.324766398	-5.46701474	4.58E-08	7.18E-07
ENSMUSG00000023050	62.47255211	-1.774287722	0.480259406	-3.694436176	0.000220375	0.001625534
ENSMUSG00000026669	111.7311507	-1.771930782	0.364947321	-4.855305634	1.20E-06	1.46E-05
ENSMUSG00000102549	18.30275637	-1.770613536	0.674747062	-2.624114479	0.008687458	0.037079294
ENSMUSG00000009575	560.6657249	-1.769849496	0.252576816	-7.007173209	2.43E-12	6.94E-11
ENSMUSG00000021176	33.73885544	-1.767541836	0.54477326	-3.244545878	0.001176381	0.006985967
ENSMUSG00000035455	187.9314343	-1.764110675	0.34805465	-5.068487589	4.01E-07	5.34E-06
ENSMUSG00000046791	382.8592891	-1.763413553	0.266270806	-6.622631984	3.53E-11	8.94E-10
ENSMUSG00000046442	137.1830965	-1.757817081	0.34174027	-5.143722397	2.69E-07	3.76E-06
ENSMUSG00000026749	568.5670392	-1.753553062	0.261167326	-6.714289617	1.89E-11	4.91E-10
ENSMUSG00000000028	179.167302	-1.753308401	0.372817282	-4.702862468	2.57E-06	2.93E-05
ENSMUSG00000042606	61.75965454	-1.75080892	0.452631247	-3.868069054	0.000109701	0.000879209
ENSMUSG00000069305	233.7302453	-1.750280028	0.306190038	-5.716319314	1.09E-08	1.88E-07
ENSMUSG00000002870	288.03848	-1.749173609	0.3147023	-5.55818357	2.73E-08	4.44E-07

	75		82			
ENSMUSG00000069268	39.33597193	-1.74711668	0.51824314	-3.371229726	0.000748334	0.004711034
ENSMUSG00000034311	79.18375203	-1.742901469	0.414506065	-4.204767112	2.61E-05	0.000240552
ENSMUSG00000027353	53.97221061	-1.742670865	0.470284582	-3.705566653	0.000210919	0.001565894
ENSMUSG00000040681	307.3733051	-1.742553793	0.284345879	-6.128289255	8.88E-10	1.82E-08
ENSMUSG00000028838	33.4651112	-1.740505939	0.57387291	-3.032911833	0.002422064	0.012782017
ENSMUSG00000031453	685.5956283	-1.740209383	0.254395662	-6.840562344	7.89E-12	2.13E-10
ENSMUSG00000039114	31.25824824	-1.738129846	0.554545296	-3.134333405	0.00172245	0.009607725
ENSMUSG00000035769	33.23527957	-1.737531488	0.560168354	-3.1018023	0.001923464	0.010537742
ENSMUSG00000044986	51.44403685	-1.737168883	0.492070473	-3.530325388	0.000415049	0.002824352
ENSMUSG00000032440	551.49524	-1.735940445	0.257999773	-6.728457259	1.71E-11	4.51E-10
ENSMUSG00000028944	49.29070315	-1.735406896	0.475230773	-3.651714057	0.000260496	0.001874066
ENSMUSG00000023191	20.07851077	-1.733950487	0.649615406	-2.669195451	0.007603319	0.033259971
ENSMUSG00000006800	95.79180647	-1.723908184	0.385846237	-4.467863148	7.90E-06	8.14E-05
ENSMUSG00000058290	69.57849203	-1.7227683	0.422909279	-4.073611962	4.63E-05	0.000406666
ENSMUSG00000028690	53.90760865	-1.722176996	0.459995512	-3.743899565	0.000181186	0.001374948
ENSMUSG00000020032	34.0658602	-1.721963927	0.543183823	-3.170131091	0.001523702	0.008672065
ENSMUSG00000039942	112.5237696	-1.721124401	0.363720197	-4.732001176	2.22E-06	2.59E-05
ENSMUSG00000068101	20.82346935	-1.718950489	0.639896467	-2.686294702	0.007224933	0.03185898
ENSMUSG00000020534	126.7128323	-1.716767194	0.352829568	-4.865712371	1.14E-06	1.39E-05
ENSMUSG00000015968	34.60753361	-1.716489254	0.536263456	-3.200832046	0.001370314	0.007937511
ENSMUSG00000044086	29.85100869	-1.716138953	0.577618298	-2.971060576	0.002967732	0.015210461
ENSMUSG00000032855	184.2108624	-1.715513645	0.392560234	-4.370064763	1.24E-05	0.000123157
ENSMUSG00000038604	23.30518035	-1.713548483	0.62926821	-2.723081279	0.006467614	0.029157452
ENSMUSG00000015342	20.65619967	-1.713064534	0.651140447	-2.630867951	0.008516712	0.036486883
ENSMUSG00000020877	24.79104927	-1.712023254	0.610453161	-2.804512062	0.00503928	0.023684271
ENSMUSG00000039187	49.40880261	-1.711847296	0.483352604	-3.541611817	0.00039769	0.002721091
ENSMUSG00000020321	2191.2019	-1.710515494	0.2278381	-7.507590488	6.02E-14	2.12E-12

	63		46			
ENSMUSG00000007080	69.71831081	-1.710191278	0.431845168	-3.96019547	7.49E-05	0.000627735
ENSMUSG00000083207	23.77980723	-1.70973597	0.658479891	-2.596489269	0.009418185	0.039557531
ENSMUSG00000100455	19.77861435	-1.708384091	0.664306676	-2.571679847	0.010120643	0.04191702
ENSMUSG00000061838	81.270412	-1.708045681	0.398850791	-4.282417684	1.85E-05	0.000175585
ENSMUSG00000019960	93.0820351	-1.706957664	0.386178012	-4.420131675	9.86E-06	9.95E-05
ENSMUSG00000047534	117.1107013	-1.705045659	0.434994771	-3.919692313	8.87E-05	0.000728923
ENSMUSG00000041684	123.6670611	-1.704179861	0.402350106	-4.235564589	2.28E-05	0.000212114
ENSMUSG00000019773	39.40616225	-1.699951681	0.531516398	-3.19830524	0.001382379	0.008004014
ENSMUSG00000091811	42.32692099	-1.699221881	0.512939857	-3.312711729	0.000923962	0.005640487
ENSMUSG00000075391	56.79384998	-1.697931519	0.46347187	-3.663505011	0.000248787	0.001805915
ENSMUSG00000022439	671.8373906	-1.697621613	0.255856246	-6.635060267	3.24E-11	8.23E-10
ENSMUSG00000053040	287.5873341	-1.697607034	0.288868298	-5.876750917	4.18E-09	7.75E-08
ENSMUSG00000024925	145.0392629	-1.697210381	0.344308388	-4.929332071	8.25E-07	1.03E-05
ENSMUSG00000049922	34.25967635	-1.696349126	0.581902457	-2.915177802	0.00355486	0.017716127
ENSMUSG00000071656	35.77952075	-1.695943071	0.545243773	-3.110430887	0.001868146	0.010274054
ENSMUSG00000048058	113.4885171	-1.695706385	0.361784831	-4.687057716	2.77E-06	3.14E-05
ENSMUSG00000031262	92.06821113	-1.694383537	0.387572314	-4.371786828	1.23E-05	0.000122474
ENSMUSG00000022881	120.5052866	-1.693965026	0.360540793	-4.698400461	2.62E-06	2.99E-05
ENSMUSG00000020739	198.014058	-1.693221178	0.310187075	-5.458709642	4.80E-08	7.49E-07
ENSMUSG00000038776	174.8063728	-1.692761936	0.331976061	-5.099048202	3.41E-07	4.63E-06
ENSMUSG00000030122	164.7156761	-1.692730357	0.323183608	-5.237673926	1.63E-07	2.35E-06
ENSMUSG00000063179	79.96788019	-1.686278899	0.411617015	-4.096718158	4.19E-05	0.000371239
ENSMUSG00000037151	49.67528542	-1.682352772	0.483650359	-3.478448305	0.000504326	0.003345491
ENSMUSG00000032589	34.48444971	-1.681330178	0.559822786	-3.003325729	0.002670464	0.013915861
ENSMUSG00000040331	157.3911189	-1.680306112	0.347170727	-4.839999401	1.30E-06	1.56E-05
ENSMUSG00000026430	118.097888	-1.680080673	0.357381127	-4.701089521	2.59E-06	2.96E-05
ENSMUSG00000021993	51.235735	-1.679452909	0.4814098	-3.488613479	0.000485	0.00323

	47		55		533	0206
ENSMUSG00000026281	422.1820368	-1.679370582	0.281932993	-5.956630198	2.57E-09	4.95E-08
ENSMUSG00000036887	63.05391491	-1.679192005	0.481161294	-3.489873411	0.000483249	0.003221271
ENSMUSG00000071041	292.2578308	-1.676815452	0.288306664	-5.816082875	6.02E-09	1.09E-07
ENSMUSG00000015846	709.1028037	-1.67535658	0.27839672	-6.017874716	1.77E-09	3.47E-08
ENSMUSG00000062981	188.2615281	-1.67454633	0.318936634	-5.250404468	1.52E-07	2.20E-06
ENSMUSG00000015880	150.9596246	-1.674407698	0.336935575	-4.969518866	6.71E-07	8.53E-06
ENSMUSG00000036617	23.20201274	-1.669405818	0.612939645	-2.72360555	0.006457358	0.02912789
ENSMUSG00000005370	337.3732345	-1.667710873	0.279805678	-5.960246723	2.52E-09	4.85E-08
ENSMUSG00000028261	80.92355294	-1.667463535	0.404394731	-4.123356234	3.73E-05	0.000333594
ENSMUSG00000041797	130.6711683	-1.665288015	0.364862514	-4.564152116	5.02E-06	5.39E-05
ENSMUSG00000028479	28.93332788	-1.664362793	0.57372632	-2.900969912	0.003720096	0.018392466
ENSMUSG00000000326	683.5294912	-1.664059854	0.249097221	-6.68036297	2.38E-11	6.14E-10
ENSMUSG00000020974	80.16159074	-1.663732775	0.40361046	-4.122125024	3.75E-05	0.000335164
ENSMUSG00000036086	360.8351574	-1.659276337	0.291565818	-5.690915164	1.26E-08	2.16E-07
ENSMUSG00000029363	172.5157114	-1.65802414	0.325751208	-5.089848017	3.58E-07	4.83E-06
ENSMUSG00000070003	90.28660992	-1.657214909	0.415177592	-3.991580812	6.56E-05	0.000558014
ENSMUSG00000033016	36.60146297	-1.656690556	0.553693012	-2.992074164	0.002770889	0.014362723
ENSMUSG00000027884	290.0721614	-1.655524931	0.28849358	-5.738515665	9.55E-09	1.67E-07
ENSMUSG00000006764	46.78803406	-1.655406245	0.489121193	-3.384450049	0.00071321	0.004520464
ENSMUSG00000022554	37.5703643	-1.651910699	0.52923039	-3.121345131	0.001800269	0.009980861
ENSMUSG00000006731	166.5618623	-1.651024008	0.349192287	-4.728122781	2.27E-06	2.63E-05
ENSMUSG00000026646	30.50481885	-1.650291892	0.558745193	-2.953567949	0.003141234	0.01596833
ENSMUSG00000037313	148.1111804	-1.649271961	0.334519021	-4.930278565	8.21E-07	1.02E-05
ENSMUSG00000028194	177.4529494	-1.648780526	0.315361995	-5.228215677	1.71E-07	2.45E-06
ENSMUSG00000021048	331.678507	-1.648673329	0.280096236	-5.886095976	3.95E-09	7.38E-08
ENSMUSG00000038482	57.26586317	-1.646637962	0.449576758	-3.662640322	0.000249629	0.001809183
ENSMUSG00000028743	120.08808	-1.642645237	0.3925146	-4.184927153	2.85E-05	0.00026

	43		55			1502
ENSMUSG00000030357	719.395892	-1.642558732	0.244257995	-6.724687704	1.76E-11	4.61E-10
ENSMUSG00000048497	250.3609766	-1.639611444	0.293329269	-5.589661919	2.28E-08	3.75E-07
ENSMUSG00000042500	180.5917938	-1.636206761	0.347543834	-4.707914801	2.50E-06	2.88E-05
ENSMUSG00000039640	259.115208	-1.635418143	0.295922755	-5.52650351	3.27E-08	5.24E-07
ENSMUSG00000083793	21.34512087	-1.634758949	0.636100731	-2.569968668	0.010170771	0.04211191
ENSMUSG00000029730	112.7737471	-1.63018098	0.378101423	-4.311491264	1.62E-05	0.000155628
ENSMUSG00000049130	62.02191999	-1.630032781	0.4386227	-3.716252673	0.000202199	0.001514286
ENSMUSG00000006641	35.22894019	-1.627272558	0.573221253	-2.838821048	0.004528054	0.021705836
ENSMUSG00000026496	1123.468982	-1.622727771	0.235679435	-6.885317649	5.77E-12	1.57E-10
ENSMUSG00000019822	71.87807378	-1.621507769	0.423119231	-3.832271498	0.000126966	0.001003496
ENSMUSG00000038665	23.49437456	-1.620676519	0.611448235	-2.650553923	0.00803599	0.034940749
ENSMUSG00000041396	51.45498246	-1.616503511	0.466670793	-3.463905468	0.000532394	0.003502858
ENSMUSG00000020142	114.8850191	-1.615637672	0.4011174	-4.027842404	5.63E-05	0.000486118
ENSMUSG00000096965	112.1658681	-1.613928415	0.359324608	-4.491561049	7.07E-06	7.36E-05
ENSMUSG00000015568	8661.86505	-1.612631677	0.251780042	-6.404922591	1.50E-10	3.47E-09
ENSMUSG00000001020	1054.262462	-1.612613012	0.274278073	-5.879482063	4.12E-09	7.65E-08
ENSMUSG00000037509	25.8829289	-1.610955757	0.590147849	-2.729749435	0.006338248	0.028696957
ENSMUSG00000000244	64.15169372	-1.6097393	0.452291494	-3.559074892	0.000372163	0.002556641
ENSMUSG00000045751	49.53968247	-1.60926921	0.498398848	-3.228878271	0.001242768	0.007306792
ENSMUSG00000060224	89.29782252	-1.602497437	0.394825585	-4.05874771	4.93E-05	0.00043205
ENSMUSG00000025436	27.64116952	-1.597765312	0.580857818	-2.750699502	0.005946817	0.027188082
ENSMUSG00000032194	77.2944971	-1.597280918	0.410112105	-3.894742193	9.83E-05	0.000798604
ENSMUSG00000062867	33.14265302	-1.595743017	0.542867823	-2.939468781	0.003287754	0.016669873
ENSMUSG00000036853	113.4875793	-1.593700299	0.35811837	-4.450205388	8.58E-06	8.79E-05
ENSMUSG00000024151	215.2841631	-1.593262813	0.309187846	-5.153057714	2.56E-07	3.60E-06
ENSMUSG00000101878	263.0345466	-1.593007367	0.323896065	-4.918267115	8.73E-07	1.08E-05
ENSMUSG00000021596	287.92132	-1.591483295	0.2803713	-5.676340026	1.38E-08	2.34E-07

	69		81			
ENSMUSG00000031482	46.562292 73	-1.590672539	0.4903650 68	-3.243853697	0.001179 243	0.00699 3304
ENSMUSG00000001911	33.330974 56	-1.5881345	0.5544999 19	-2.864084278	0.004182 167	0.02024 6062
ENSMUSG00000028312	175.61259 16	-1.587663523	0.3192556 58	-4.973016089	6.59E-07	8.38E-06
ENSMUSG00000020623	32.360774 52	-1.585870851	0.5753142 83	-2.756529601	0.005841 832	0.02679 4616
ENSMUSG00000021947	121.45446 94	-1.584577893	0.3910743 45	-4.051858456	5.08E-05	0.00044 3557
ENSMUSG00000054150	135.44531 55	-1.580365816	0.3920679 7	-4.030846529	5.56E-05	0.00048 0855
ENSMUSG00000045917	206.34805 56	-1.578449113	0.3157279 68	-4.999395918	5.75E-07	7.41E-06
ENSMUSG00000021886	244.16439 65	-1.578413484	0.3027995 05	-5.21273469	1.86E-07	2.65E-06
ENSMUSG00000090290	67.941961 24	-1.576888139	0.4403049 02	-3.581354951	0.000341 817	0.00237 797
ENSMUSG00000041058	303.59082 15	-1.576259593	0.3038337 94	-5.187900832	2.13E-07	3.01E-06
ENSMUSG00000037348	472.60745 1	-1.57510332	0.2986093 48	-5.274795752	1.33E-07	1.94E-06
ENSMUSG00000031659	1186.1922 91	-1.574493526	0.2475280 71	-6.360868563	2.01E-10	4.52E-09
ENSMUSG00000096544	79.631530 41	-1.572036917	0.4154029 73	-3.784366074	0.000154 101	0.00119 3022
ENSMUSG00000063275	45.495013 32	-1.570402206	0.5018964 55	-3.128936637	0.001754 401	0.00976 058
ENSMUSG00000027111	688.73142 42	-1.562468629	0.2444700 25	-6.39124828	1.65E-10	3.75E-09
ENSMUSG00000002068	97.507766 85	-1.562052106	0.3768929 17	-4.144551505	3.40E-05	0.00030 7396
ENSMUSG00000040061	216.60561 59	-1.559823696	0.3031751 47	-5.144958975	2.68E-07	3.74E-06
ENSMUSG00000057541	98.439475 07	-1.559551277	0.3781749 85	-4.123887988	3.73E-05	0.00033 3259
ENSMUSG00000049625	66.913404 78	-1.558382697	0.4693897 11	-3.320018865	0.000900 114	0.00551 4554
ENSMUSG00000032417	49.002025 53	-1.557327095	0.5017219 59	-3.103964394	0.001909 463	0.01047 6057
ENSMUSG00000083879	34.963965 74	-1.55459952	0.5601217 63	-2.775467091	0.005512 248	0.02556 5264
ENSMUSG00000024654	94.209066 31	-1.553246626	0.3908054 9	-3.974474939	7.05E-05	0.00059 4513
ENSMUSG00000027763	2118.5689 17	-1.548863431	0.2492020 97	-6.215290521	5.12E-10	1.09E-08
ENSMUSG00000029283	136.33754 77	-1.548040611	0.3470220 83	-4.460928239	8.16E-06	8.40E-05
ENSMUSG00000056367	53.731535 88	-1.545424683	0.4742538 47	-3.258644484	0.001119 459	0.00669 0876
ENSMUSG00000040010	225.66412 27	-1.544904	0.2970334 66	-5.201110906	1.98E-07	2.82E-06
ENSMUSG00000036880	354.49457	-1.542330375	0.2799560	-5.509186791	3.60E-08	5.72E-07

	35		87			
ENSMUSG00000020941	65.25120173	-1.541812784	0.438626562	-3.515092147	0.000439601	0.002956841
ENSMUSG00000018068	151.2421201	-1.541452654	0.33986638	-4.535466711	5.75E-06	6.08E-05
ENSMUSG00000082308	56.14054845	-1.541203381	0.479007358	-3.217494165	0.001293157	0.00757153
ENSMUSG00000022962	376.8016651	-1.540293078	0.287123253	-5.364571001	8.11E-08	1.23E-06
ENSMUSG00000048537	42.03556767	-1.540092694	0.515805491	-2.985801279	0.002828364	0.014608779
ENSMUSG00000026023	31.13824461	-1.539364336	0.593042674	-2.595705848	0.009439684	0.039635683
ENSMUSG00000031903	233.368957	-1.537708938	0.35677465	-4.310028575	1.63E-05	0.000156516
ENSMUSG00000029094	80.71437736	-1.537328303	0.400803993	-3.835611244	0.000125252	0.000991099
ENSMUSG00000039783	23.51696656	-1.53731224	0.615051674	-2.499484684	0.012437408	0.049450151
ENSMUSG00000037341	89.77790042	-1.535126848	0.386173898	-3.975221668	7.03E-05	0.000593082
ENSMUSG00000032228	515.7670817	-1.535076846	0.258089325	-5.947850979	2.72E-09	5.18E-08
ENSMUSG00000019066	93.22536895	-1.532097776	0.387783712	-3.950908016	7.79E-05	0.000648636
ENSMUSG00000041840	99.99785335	-1.530964897	0.383758766	-3.989393944	6.62E-05	0.000562485
ENSMUSG00000035311	532.1413183	-1.530043043	0.257474719	-5.942498159	2.81E-09	5.34E-08
ENSMUSG00000013629	134.9141196	-1.529985116	0.380364108	-4.022422424	5.76E-05	0.000496818
ENSMUSG00000024067	103.3117169	-1.529219263	0.372075846	-4.109966498	3.96E-05	0.000351933
ENSMUSG00000024269	63.56790476	-1.527878468	0.437533669	-3.492024906	0.000479374	0.00319699
ENSMUSG00000045322	29.00898104	-1.527378737	0.603954963	-2.528961314	0.011440064	0.046208686
ENSMUSG00000032555	467.617342	-1.526524882	0.274425783	-5.562614643	2.66E-08	4.35E-07
ENSMUSG00000029290	80.17353456	-1.526402665	0.416885843	-3.661440395	0.000250801	0.001815758
ENSMUSG00000027331	154.5856421	-1.523554966	0.334424533	-4.555751195	5.22E-06	5.58E-05
ENSMUSG00000023031	52.08288185	-1.523233381	0.468445301	-3.251678213	0.001147258	0.006836163
ENSMUSG00000017670	110.829086	-1.522580888	0.370991996	-4.104080157	4.06E-05	0.000360313
ENSMUSG00000037628	30.18741893	-1.522275294	0.561573703	-2.710731089	0.006713505	0.030107521
ENSMUSG00000022978	86.51136648	-1.518542723	0.405830669	-3.741813619	0.000182697	0.001385646
ENSMUSG00000035266	64.93775628	-1.518346787	0.444655036	-3.414662298	0.000638611	0.00410322
ENSMUSG00000032113	44.936230	-1.517494654	0.4917162	-3.086118844	0.002027	0.01099

	69		08		878	8046
ENSMUSG00000062510	98.76608556	-1.516865416	0.41831139	-3.62616331	0.000287663	0.002045889
ENSMUSG00000030802	311.9961353	-1.516738734	0.27662987	-5.482917431	4.18E-08	6.60E-07
ENSMUSG00000020712	69.69990134	-1.515897777	0.43575879	-3.478754332	0.00050375	0.003343291
ENSMUSG00000055760	42.84207817	-1.515804031	0.499279162	-3.035984969	0.002397513	0.012681558
ENSMUSG00000024975	46.0642314	-1.513751344	0.488727834	-3.09732992	0.001952724	0.010649457
ENSMUSG00000037907	28.15536026	-1.51238913	0.573646725	-2.636446902	0.008377931	0.036004875
ENSMUSG00000037466	43.54854493	-1.511056435	0.502786717	-3.005362679	0.002652643	0.013838781
ENSMUSG00000022303	68.93334897	-1.509005266	0.420335166	-3.590004807	0.000330672	0.002307463
ENSMUSG00000032397	179.502933	-1.50640367	0.35557218	-4.236562236	2.27E-05	0.000211346
ENSMUSG00000026832	134.635574	-1.502365512	0.340689463	-4.409779796	1.03E-05	0.000104198
ENSMUSG00000024036	32.48245411	-1.499590708	0.594199451	-2.52371608	0.011612167	0.046793517
ENSMUSG00000017697	55.08969897	-1.499524366	0.452375612	-3.314777202	0.000917162	0.00560397
ENSMUSG00000030376	30.83831642	-1.499491795	0.563343061	-2.66177379	0.00777301	0.033915663
ENSMUSG00000039356	239.2031953	-1.49885639	0.308991814	-4.850796435	1.23E-06	1.49E-05
ENSMUSG00000028671	161.1741781	-1.497360373	0.3463852	-4.322818564	1.54E-05	0.000148993
ENSMUSG00000026158	80.87584749	-1.496625172	0.434966404	-3.440783377	0.000580033	0.003774619
ENSMUSG00000023026	415.4687418	-1.491137419	0.276717369	-5.388665792	7.10E-08	1.08E-06
ENSMUSG00000000823	47.61871875	-1.491095743	0.485192781	-3.073202653	0.002117746	0.011395254
ENSMUSG00000034416	32.09344927	-1.488155544	0.565487338	-2.631633715	0.008497542	0.036438928
ENSMUSG00000035891	1998.478573	-1.487214087	0.236571637	-6.28652743	3.25E-10	7.06E-09
ENSMUSG00000090035	52.30568904	-1.4852952	0.474645404	-3.12927332	0.001752392	0.009754888
ENSMUSG00000043419	101.3509582	-1.482239411	0.39321374	-3.769551416	0.000163541	0.001257057
ENSMUSG00000026622	150.4619693	-1.480110611	0.340439894	-4.347641496	1.38E-05	0.000134902
ENSMUSG00000039031	132.9682706	-1.478393568	0.358266138	-4.126523312	3.68E-05	0.00032968
ENSMUSG00000091408	27.96814251	-1.475514449	0.57304347	-2.574873504	0.010027675	0.041594821
ENSMUSG00000047284	64.37275403	-1.471321519	0.511341415	-2.877376008	0.004009975	0.019571475
ENSMUSG00000039231	55.445726	-1.471045774	0.4586534	-3.207314175	0.001339	0.00780

	54		7		806	3672
ENSMUSG00000029246	262.53363<br>58	-1.469507269	0.2859288<br>42	-5.139416009	2.76E-07	3.84E-06
ENSMUSG00000020865	105.50950<br>36	-1.465826575	0.3643823<br>93	-4.022770039	5.75E-05	0.00049<br>6398
ENSMUSG00000017286	350.82626<br>97	-1.460943677	0.2692298<br>08	-5.426381608	5.75E-08	8.92E-07
ENSMUSG00000015944	63.977555<br>36	-1.460738685	0.4296760<br>07	-3.399628234	0.000674<br>775	0.00429<br>9301
ENSMUSG00000035842	52.033879<br>22	-1.45501736	0.4691625<br>21	-3.101307744	0.001926<br>679	0.01054<br>9397
ENSMUSG00000037940	138.18255<br>47	-1.454424022	0.3424884<br>88	-4.246636236	2.17E-05	0.00020<br>3006
ENSMUSG00000009418	127.70428<br>92	-1.45357522	0.3436360<br>86	-4.229984216	2.34E-05	0.00021<br>7003
ENSMUSG00000038644	98.258417<br>86	-1.451221295	0.3910832<br>31	-3.710773515	0.000206<br>627	0.00154<br>0708
ENSMUSG00000006678	194.79272<br>19	-1.449902491	0.3134437<br>37	-4.625718491	3.73E-06	4.13E-05
ENSMUSG00000105283	35.252299<br>26	-1.446977544	0.5689837<br>06	-2.543091355	0.010987<br>65	0.04471<br>073
ENSMUSG00000044080	292.37678<br>64	-1.445069095	0.2826187<br>1	-5.113140226	3.17E-07	4.35E-06
ENSMUSG00000027379	186.76149<br>27	-1.444194641	0.3173106<br>04	-4.551359528	5.33E-06	5.69E-05
ENSMUSG00000021608	309.12909<br>62	-1.443903676	0.2957792<br>08	-4.881694303	1.05E-06	1.29E-05
ENSMUSG00000024007	201.90935<br>65	-1.442891222	0.3397882<br>96	-4.246441793	2.17E-05	0.00020<br>3044
ENSMUSG00000026365	179.18717<br>73	-1.442887516	0.3391366<br>17	-4.254590756	2.09E-05	0.00019<br>6863
ENSMUSG00000029478	34.043902<br>36	-1.442201782	0.5426377<br>39	-2.657761665	0.007866<br>15	0.03426<br>751
ENSMUSG00000022106	385.00257<br>58	-1.441219466	0.2811418<br>41	-5.126307278	2.95E-07	4.08E-06
ENSMUSG00000040618	599.44592<br>55	-1.440594062	0.2655452<br>84	-5.425041043	5.79E-08	8.97E-07
ENSMUSG00000042700	684.42318<br>53	-1.435594206	0.2471775<br>46	-5.807947478	6.32E-09	1.14E-07
ENSMUSG00000041707	511.93915<br>42	-1.435489313	0.2583392<br>52	-5.556605522	2.75E-08	4.47E-07
ENSMUSG00000106939	30.883842<br>98	-1.434456302	0.5534996<br>95	-2.591611732	0.009552<br>752	0.03998<br>7923
ENSMUSG00000073678	481.84335<br>32	-1.43413588	0.2704513<br>51	-5.302749929	1.14E-07	1.69E-06
ENSMUSG00000073755	92.115474<br>12	-1.432869928	0.3810455<br>8	-3.760363594	0.000169<br>667	0.00129<br>8314
ENSMUSG00000040043	40.603684<br>29	-1.432714911	0.5231234<br>76	-2.738770057	0.006166<br>949	0.02801<br>3933
ENSMUSG00000020441	31.599778<br>75	-1.430382039	0.5480331<br>66	-2.610028238	0.009053<br>475	0.03837<br>8557
ENSMUSG00000022972	33.174287<br>99	-1.429177001	0.5530326<br>04	-2.584254511	0.009758<br>975	0.04062<br>7811
ENSMUSG00000041126	88.934000	-1.428610802	0.4094414	-3.489169871	0.000484	0.00322

	36		59		523	6623
ENSMUSG00000041261	61.66100021	-1.427694911	0.445503567	-3.20467672	0.001352143	0.007868838
ENSMUSG00000037572	84.092684	-1.427683035	0.394869305	-3.615583727	0.000299672	0.00212248
ENSMUSG00000105954	47.42734717	-1.427304425	0.497546179	-2.86868734	0.00412179	0.020045825
ENSMUSG00000045658	176.4886366	-1.427141821	0.31891742	-4.474957248	7.64E-06	7.92E-05
ENSMUSG00000066800	574.7949372	-1.42579907	0.247896319	-5.75159437	8.84E-09	1.56E-07
ENSMUSG00000082279	49.49817085	-1.424958732	0.480933694	-2.962900603	0.00304755	0.015538195
ENSMUSG00000004633	103.2776343	-1.423811504	0.374473115	-3.802172835	0.000143433	0.001119444
ENSMUSG00000029910	102.2242147	-1.423550525	0.380200099	-3.744213975	0.000180959	0.001373988
ENSMUSG00000029319	143.435998	-1.42342488	0.342462642	-4.156438405	3.23E-05	0.00029319
ENSMUSG00000037306	360.5587171	-1.421346846	0.285033625	-4.986593585	6.15E-07	7.86E-06
ENSMUSG00000027956	478.9371727	-1.41928737	0.26228702	-5.411199417	6.26E-08	9.65E-07
ENSMUSG00000074476	62.3636181	-1.418848041	0.449781139	-3.154529878	0.001607569	0.009092751
ENSMUSG00000027198	84.51036908	-1.418255477	0.402764543	-3.52130172	0.000429434	0.002899206
ENSMUSG00000074064	103.9622186	-1.417812149	0.392676638	-3.610635343	0.000305448	0.002161158
ENSMUSG00000081669	77.49952023	-1.415875049	0.441920419	-3.203914068	0.001355729	0.007880716
ENSMUSG00000039914	167.4480998	-1.415109478	0.319829079	-4.424580406	9.66E-06	9.77E-05
ENSMUSG00000041491	153.3679681	-1.412904578	0.337685863	-4.184079738	2.86E-05	0.000262304
ENSMUSG00000103726	37.13386519	-1.412787218	0.522993863	-2.701345689	0.006905952	0.030699341
ENSMUSG00000037339	126.6848496	-1.411038067	0.385174437	-3.663374124	0.000248915	0.001805915
ENSMUSG00000033207	95.78807852	-1.410801518	0.383104556	-3.682549572	0.000230913	0.00168782
ENSMUSG00000033707	104.4789174	-1.409901933	0.391192124	-3.604116359	0.000313217	0.002203611
ENSMUSG00000015289	67.93957713	-1.409590885	0.421466011	-3.344494804	0.000824326	0.005114253
ENSMUSG00000025264	67.33529971	-1.409183991	0.45877893	-3.071597016	0.00212917	0.011451937
ENSMUSG00000041920	111.7596616	-1.40734602	0.369803996	-3.805653902	0.00014143	0.001105073
ENSMUSG00000052142	54.25128323	-1.406478559	0.478950784	-2.936582644	0.003318504	0.016813344
ENSMUSG00000031730	99.70676139	-1.405309885	0.383636121	-3.663132345	0.00024915	0.001806665
ENSMUSG00000021245	41.443214	-1.404912529	0.5302424	-2.649566416	0.008059	0.03496

	13		28		513	5377
ENSMUSG00000010663	803.0320527	-1.403191983	0.252077243	-5.566515898	2.60E-08	4.26E-07
ENSMUSG00000054115	178.4917345	-1.400684697	0.314785611	-4.449646511	8.60E-06	8.81E-05
ENSMUSG00000025086	69.46805994	-1.400430767	0.42483362	-3.296421705	0.000979249	0.005946215
ENSMUSG00000003824	89.80453912	-1.399485538	0.396702421	-3.527796816	0.000419034	0.002842998
ENSMUSG00000022544	101.3709409	-1.398909859	0.385566427	-3.628194161	0.000285411	0.002031975
ENSMUSG00000010142	242.4754503	-1.398411533	0.295897846	-4.725994301	2.29E-06	2.66E-05
ENSMUSG00000020087	116.6900546	-1.396066037	0.399374098	-3.495634906	0.000472935	0.003161756
ENSMUSG00000072082	77.30610864	-1.393111585	0.403351578	-3.453839432	0.000552666	0.003620598
ENSMUSG00000020899	234.0454904	-1.391255255	0.332601831	-4.182945262	2.88E-05	0.000263441
ENSMUSG00000024841	461.1832079	-1.388466939	0.265423584	-5.231136272	1.68E-07	2.42E-06
ENSMUSG00000056069	523.4221007	-1.387696944	0.251220368	-5.523823388	3.32E-08	5.31E-07
ENSMUSG00000028018	52.45896154	-1.387650689	0.467847545	-2.966031788	0.003016693	0.015403792
ENSMUSG00000017485	278.0039369	-1.387480885	0.285106387	-4.866537363	1.14E-06	1.38E-05
ENSMUSG00000020547	251.3751783	-1.387254608	0.292762596	-4.738496744	2.15E-06	2.51E-05
ENSMUSG00000045045	103.0928035	-1.385242343	0.434098795	-3.191076224	0.001417439	0.008186262
ENSMUSG00000037824	162.7361652	-1.384680488	0.323011438	-4.286784696	1.81E-05	0.000172289
ENSMUSG00000040209	134.4185116	-1.382494407	0.411213673	-3.361985499	0.000773842	0.004851557
ENSMUSG00000021377	426.4876055	-1.382288733	0.319922739	-4.320695482	1.56E-05	0.000150222
ENSMUSG00000049624	371.7132458	-1.381858987	0.272173831	-5.077119217	3.83E-07	5.12E-06
ENSMUSG00000032498	83.04843293	-1.381832747	0.392849719	-3.517458917	0.0004357	0.00293717
ENSMUSG00000000552	35.60287428	-1.380224424	0.525099734	-2.628499566	0.008576246	0.0366846
ENSMUSG00000002058	289.5609634	-1.379629447	0.279865438	-4.929617097	8.24E-07	1.03E-05
ENSMUSG00000032279	573.2677006	-1.375313328	0.260226123	-5.285070202	1.26E-07	1.85E-06
ENSMUSG00000053617	257.7909824	-1.37407086	0.299664221	-4.585368429	4.53E-06	4.91E-05
ENSMUSG00000019823	52.16161528	-1.373550385	0.478620117	-2.869813316	0.004107142	0.019995873
ENSMUSG00000026170	196.2855229	-1.372110184	0.308781702	-4.443625302	8.85E-06	9.02E-05
ENSMUSG00000020453	116.01863	-1.371690251	0.3800821	-3.608931267	0.000307	0.00217

	54		21		461	428
ENSMUSG00000006378	306.0734728	-1.3712596	0.302654241	-4.530779402	5.88E-06	6.21E-05
ENSMUSG00000063193	393.1588128	-1.367134628	0.291798143	-4.685206753	2.80E-06	3.17E-05
ENSMUSG00000041429	47.79198137	-1.366908867	0.478027482	-2.859477579	0.004243394	0.020520719
ENSMUSG00000031216	215.6771811	-1.366276994	0.298443649	-4.578006602	4.69E-06	5.07E-05
ENSMUSG00000075585	37.25758478	-1.365200642	0.537889398	-2.538069438	0.011146587	0.045209816
ENSMUSG00000029915	700.0357571	-1.363261161	0.290013311	-4.700684788	2.59E-06	2.96E-05
ENSMUSG00000025150	38.98927893	-1.363215937	0.525301164	-2.595113108	0.00945598	0.039691945
ENSMUSG00000022372	194.1594924	-1.361324887	0.320095962	-4.252864913	2.11E-05	0.000198115
ENSMUSG00000028884	270.5366305	-1.360521533	0.29356796	-4.634434678	3.58E-06	3.98E-05
ENSMUSG00000004880	384.1876046	-1.35894249	0.264522391	-5.137343889	2.79E-07	3.87E-06
ENSMUSG00000027508	237.2213107	-1.357680656	0.301999446	-4.495639566	6.94E-06	7.24E-05
ENSMUSG00000018509	40.155104	-1.355987848	0.520335268	-2.605988737	0.009160947	0.038714395
ENSMUSG00000025730	233.0606139	-1.355707094	0.304187273	-4.456817283	8.32E-06	8.55E-05
ENSMUSG00000052160	1231.338404	-1.354950193	0.244110175	-5.550568276	2.85E-08	4.61E-07
ENSMUSG00000049881	533.0779469	-1.354745069	0.254248944	-5.328419641	9.91E-08	1.48E-06
ENSMUSG00000037139	73.61050615	-1.354511502	0.427440551	-3.16888863	0.00153023	0.008698384
ENSMUSG00000034285	91.71298226	-1.352721727	0.381806916	-3.542947158	0.000395682	0.002710056
ENSMUSG00000031851	172.7321163	-1.350133997	0.352220783	-3.833203668	0.000126485	0.001000276
ENSMUSG00000055302	688.1153076	-1.349123412	0.263259476	-5.124690791	2.98E-07	4.11E-06
ENSMUSG00000068551	38.9126578	-1.349025044	0.516553422	-2.611588631	0.009012262	0.038239336
ENSMUSG00000016503	94.45351592	-1.347681393	0.376641206	-3.578157067	0.000346025	0.002399939
ENSMUSG00000006442	241.9363455	-1.345760688	0.295560369	-4.553251485	5.28E-06	5.64E-05
ENSMUSG00000042826	37.18343999	-1.343377715	0.522750587	-2.569825359	0.010174979	0.042116609
ENSMUSG00000034424	476.6805753	-1.340124082	0.266219609	-5.03390447	4.81E-07	6.28E-06
ENSMUSG00000020692	62.7265748	-1.337282093	0.442647364	-3.02110032	0.002518579	0.013204715
ENSMUSG00000046341	87.57596127	-1.337226586	0.425235393	-3.144673772	0.001662721	0.009344053
ENSMUSG00000035960	163.27830	-1.336906145	0.3200024	-4.17779962	2.94E-05	0.00026

	52		57			8752
ENSMUSG00000028093	58.62526435	-1.334868986	0.440917816	-3.027477994	0.002466037	0.012978891
ENSMUSG00000014907	34.42837904	-1.332964226	0.530764467	-2.511404416	0.012025185	0.048034146
ENSMUSG00000024799	43.70885217	-1.332956836	0.519089362	-2.56787546	0.010232391	0.042290386
ENSMUSG00000091625	36.00693651	-1.332614505	0.525096332	-2.537847672	0.011153652	0.045225089
ENSMUSG00000035772	90.43782751	-1.331560781	0.397613873	-3.348879083	0.000811392	0.00504543
ENSMUSG00000029033	75.0474042	-1.329605308	0.448985642	-2.961353732	0.003062899	0.015606969
ENSMUSG00000005533	170.5990238	-1.322918565	0.345377327	-3.830357297	0.000127957	0.001009412
ENSMUSG00000023992	2328.676667	-1.32147872	0.258531284	-5.111484772	3.20E-07	4.37E-06
ENSMUSG00000083854	51.89114623	-1.321428304	0.46544957	-2.839036471	0.004524998	0.021698777
ENSMUSG00000037318	261.5660735	-1.320944117	0.290955359	-4.540023331	5.62E-06	5.97E-05
ENSMUSG00000031864	228.9232864	-1.31735713	0.296966059	-4.436052839	9.16E-06	9.32E-05
ENSMUSG00000018428	476.9614591	-1.316481188	0.324596822	-4.055742695	5.00E-05	0.000437363
ENSMUSG00000034165	89.39971857	-1.316472259	0.411212308	-3.201441771	0.001367417	0.007925351
ENSMUSG00000025402	44.39058133	-1.313744872	0.517992401	-2.536224219	0.011205496	0.045395012
ENSMUSG00000027395	166.3607755	-1.312973586	0.324617207	-4.04468265	5.24E-05	0.000456781
ENSMUSG00000025384	53.64855623	-1.312240979	0.492054641	-2.666860283	0.007656349	0.033459907
ENSMUSG00000074825	102.1899372	-1.309334159	0.370422026	-3.534709242	0.000408224	0.002784722
ENSMUSG00000036833	1130.885741	-1.307167626	0.263145935	-4.967462733	6.78E-07	8.60E-06
ENSMUSG00000027864	201.0309042	-1.30556402	0.330961585	-3.944759989	7.99E-05	0.000663086
ENSMUSG00000026313	171.8314694	-1.30516905	0.347540396	-3.755445602	0.000173033	0.001319656
ENSMUSG00000031097	141.296005	-1.30412232	0.349790358	-3.72829694	0.000192778	0.00145246
ENSMUSG00000003546	595.2262961	-1.303470211	0.289952845	-4.49545585	6.94E-06	7.24E-05
ENSMUSG00000060510	110.6461231	-1.303249726	0.36866754	-3.535027047	0.000407733	0.002782862
ENSMUSG00000037573	66.66656645	-1.301530346	0.422620479	-3.079667007	0.002072321	0.011188982
ENSMUSG00000001768	748.9549767	-1.300999884	0.244047076	-5.330938227	9.77E-08	1.46E-06
ENSMUSG00000015214	291.7970395	-1.300342647	0.292538363	-4.445032896	8.79E-06	8.97E-05
ENSMUSG00000079293	944.82502	-1.299681825	0.3250274	-3.998682964	6.37E-05	0.00054

	3		74			2544
ENSMUSG00000018819	302.0625649	-1.296605507	0.281554277	-4.605170702	4.12E-06	4.51E-05
ENSMUSG00000031511	374.2592315	-1.296393466	0.270416069	-4.794069637	1.63E-06	1.94E-05
ENSMUSG00000036875	89.09888594	-1.295838723	0.407990364	-3.176150316	0.001492436	0.008537266
ENSMUSG00000050493	110.6884262	-1.295281047	0.372897672	-3.473556268	0.00051361	0.003393935
ENSMUSG00000020918	70.46503604	-1.295112895	0.428932832	-3.019383922	0.002532893	0.013274686
ENSMUSG00000061273	232.7773193	-1.294951819	0.302941581	-4.274592535	1.91E-05	0.000181364
ENSMUSG00000033685	229.94243	-1.292594604	0.322125837	-4.012700802	6.00E-05	0.000514499
ENSMUSG00000041229	174.8279802	-1.292372531	0.337830085	-3.825510481	0.000130501	0.001026117
ENSMUSG00000020598	164.5201504	-1.291382332	0.32812859	-3.935598331	8.30E-05	0.000686405
ENSMUSG00000027894	347.8532704	-1.289289165	0.282691772	-4.560759424	5.10E-06	5.47E-05
ENSMUSG00000069270	63.58410647	-1.288520818	0.442703215	-2.910574795	0.003607646	0.017920233
ENSMUSG00000024247	122.0444446	-1.28826649	0.365673781	-3.522993869	0.000426701	0.002886446
ENSMUSG00000027367	845.2694082	-1.287311754	0.243160014	-5.294093117	1.20E-07	1.77E-06
ENSMUSG00000052688	1575.307226	-1.286174277	0.260204398	-4.942938271	7.70E-07	9.62E-06
ENSMUSG00000018821	158.6564197	-1.285655242	0.372727845	-3.449313646	0.000562013	0.003673054
ENSMUSG00000026630	76.17188678	-1.285641387	0.443439868	-2.899246278	0.00374061	0.018472785
ENSMUSG00000036339	124.0254185	-1.281906135	0.351630099	-3.645609799	0.000266758	0.001914097
ENSMUSG00000026938	58.7439995	-1.280122352	0.45507955	-2.812963911	0.004908716	0.023197915
ENSMUSG00000062762	156.8351328	-1.27916738	0.326369645	-3.919382208	8.88E-05	0.000729423
ENSMUSG00000021556	446.4690993	-1.277596991	0.263116358	-4.855634985	1.20E-06	1.46E-05
ENSMUSG00000035697	420.567471	-1.276983887	0.280099652	-4.559034183	5.14E-06	5.51E-05
ENSMUSG00000034329	84.65436594	-1.276456674	0.408030743	-3.128334556	0.001758	0.009770228
ENSMUSG00000028633	124.4543815	-1.276160907	0.346158259	-3.686640068	0.000227234	0.001668049
ENSMUSG00000031101	454.7088336	-1.275645608	0.262458415	-4.86037229	1.17E-06	1.42E-05
ENSMUSG00000023066	111.1017263	-1.27526884	0.36083752	-3.534191349	0.000409025	0.002786126
ENSMUSG00000026471	180.1027544	-1.274162691	0.315500475	-4.038544444	5.38E-05	0.000467116
ENSMUSG00000026858	47.279934	-1.273971	0.5043756	-2.525837684	0.011542	0.04653

	69		41		28	4125
ENSMUSG00000066621	1322.76336	-1.272862184	0.279157231	-4.559660438	5.12E-06	5.49E-05
ENSMUSG00000005674	275.1264593	-1.272814334	0.288764403	-4.407795153	1.04E-05	0.000105003
ENSMUSG00000044197	408.9664108	-1.270878228	0.306592182	-4.145174935	3.40E-05	0.000306763
ENSMUSG00000038244	92.33055025	-1.269932607	0.428047164	-2.96680533	0.003009114	0.015376551
ENSMUSG00000037243	50.37936654	-1.269816819	0.474569655	-2.675722742	0.00745683	0.032735895
ENSMUSG00000019055	569.3032073	-1.26801482	0.280676855	-4.51770353	6.25E-06	6.59E-05
ENSMUSG00000016087	374.5537632	-1.267944142	0.278814139	-4.547632145	5.43E-06	5.78E-05
ENSMUSG00000028988	150.5096671	-1.266258703	0.328290235	-3.857131792	0.000114725	0.000915637
ENSMUSG00000032349	601.5956379	-1.265179776	0.260123575	-4.863764376	1.15E-06	1.40E-05
ENSMUSG00000047221	60.17081278	-1.264966453	0.438542563	-2.884478178	0.003920629	0.019213284
ENSMUSG00000026012	2185.443591	-1.263222011	0.245519808	-5.145092037	2.67E-07	3.74E-06
ENSMUSG00000027811	65.94758065	-1.262996731	0.434649177	-2.905784245	0.00366334	0.01815771
ENSMUSG00000040699	301.8114591	-1.262492798	0.314906453	-4.009104249	6.09E-05	0.000521745
ENSMUSG00000019857	47.34781183	-1.262359645	0.496175485	-2.544179794	0.010953469	0.044611379
ENSMUSG00000027454	131.0372656	-1.261203072	0.341000654	-3.698535644	0.000216847	0.001602959
ENSMUSG00000028497	796.3768373	-1.26016829	0.249380644	-5.053192058	4.34E-07	5.74E-06
ENSMUSG00000007029	375.4103654	-1.259498689	0.345843841	-3.641813269	0.000270724	0.001939508
ENSMUSG00000057406	277.1154145	-1.258515411	0.287981144	-4.370131299	1.24E-05	0.000123157
ENSMUSG00000087679	143.3472534	-1.25834398	0.355423009	-3.540412264	0.000399502	0.002730764
ENSMUSG00000031388	82.99788862	-1.258209056	0.398757607	-3.155323017	0.001603205	0.009079308
ENSMUSG00000022516	145.9675738	-1.257929776	0.346029917	-3.635320859	0.000277635	0.001980732
ENSMUSG00000042770	208.8398968	-1.256240614	0.317561484	-3.955897295	7.62E-05	0.000636793
ENSMUSG00000027679	60.15133282	-1.256167188	0.440579738	-2.851168766	0.004355884	0.020975894
ENSMUSG00000079020	153.9366456	-1.2551663	0.330846378	-3.793803964	0.000148357	0.001153933
ENSMUSG00000052698	92.04715758	-1.253987823	0.404932274	-3.096784087	0.001956323	0.01066131
ENSMUSG00000026355	1118.132478	-1.252732434	0.251513944	-4.980767318	6.33E-07	8.08E-06
ENSMUSG00000008855	206.17133	-1.250309177	0.3662395	-3.413910923	0.000640	0.00411

	65		43		375	2624
ENSMUSG00000024913	330.2977438	-1.247582418	0.296634415	-4.205791223	2.60E-05	0.000239626
ENSMUSG00000071669	81.71236043	-1.247112224	0.397576151	-3.13678831	0.001708094	0.009554869
ENSMUSG00000027378	93.90980097	-1.246619321	0.380823212	-3.273485656	0.001062298	0.006391041
ENSMUSG00000021003	262.8249828	-1.245797442	0.285012034	-4.371034536	1.24E-05	0.000122808
ENSMUSG00000001524	77.46490598	-1.242291897	0.401593417	-3.093407023	0.001978725	0.010774109
ENSMUSG00000000168	224.8467105	-1.240343407	0.296635551	-4.181371394	2.90E-05	0.000265094
ENSMUSG00000028121	57.45137303	-1.235439834	0.447173387	-2.76277585	0.005731211	0.026366649
ENSMUSG00000021906	119.1562991	-1.234645319	0.388649139	-3.17676072	0.001489299	0.00852583
ENSMUSG00000034959	88.69438584	-1.233826653	0.397717663	-3.102267681	0.001920442	0.010527864
ENSMUSG00000036916	76.87693295	-1.233195059	0.434702442	-2.836871709	0.004555791	0.021815905
ENSMUSG00000032264	500.307622	-1.232558817	0.263554171	-4.676681117	2.92E-06	3.30E-05
ENSMUSG00000027947	73.93586364	-1.231426855	0.407861869	-3.019225258	0.00253422	0.013276563
ENSMUSG00000026192	679.740811	-1.22816209	0.244193998	-5.029452393	4.92E-07	6.42E-06
ENSMUSG00000009905	141.0492103	-1.22657768	0.34095549	-3.597471562	0.000321326	0.002252752
ENSMUSG00000039716	87.05326642	-1.226308075	0.394907633	-3.105303549	0.001900838	0.010436468
ENSMUSG00000004798	141.3013607	-1.225939941	0.33443055	-3.665753449	0.000246611	0.001793954
ENSMUSG00000056124	371.8670289	-1.223302349	0.273010035	-4.480796273	7.44E-06	7.72E-05
ENSMUSG00000024997	910.7735494	-1.220777633	0.24172112	-5.050355691	4.41E-07	5.82E-06
ENSMUSG00000057329	193.9076526	-1.217983458	0.326827025	-3.726691378	0.00019401	0.001460134
ENSMUSG00000002107	217.2166884	-1.215255076	0.310585026	-3.912793511	9.12E-05	0.000747382
ENSMUSG00000032120	77.63877407	-1.214561967	0.434322946	-2.796448999	0.005166756	0.024216962
ENSMUSG00000036202	291.6120883	-1.213245251	0.287417451	-4.221195504	2.43E-05	0.000224879
ENSMUSG00000008226	131.6768792	-1.212632083	0.361561921	-3.353871116	0.000796895	0.004968809
ENSMUSG00000038068	475.619137	-1.210861138	0.260668298	-4.645218267	3.40E-06	3.80E-05
ENSMUSG00000040029	273.7806431	-1.210228021	0.281615014	-4.297455604	1.73E-05	0.000164775
ENSMUSG00000045404	350.6176844	-1.20972594	0.278645866	-4.341445856	1.42E-05	0.000138269
ENSMUSG00000026939	302.62013	-1.209257305	0.2783618	-4.344192361	1.40E-05	0.00013

	79		23			6648
ENSMUSG00000001100	632.0068701	-1.207156778	0.262029544	-4.606949116	4.09E-06	4.48E-05
ENSMUSG00000056209	88.0250066	-1.205037181	0.393406164	-3.063086683	0.002190666	0.011714037
ENSMUSG00000027423	2344.596585	-1.204725771	0.222785875	-5.407550048	6.39E-08	9.82E-07
ENSMUSG00000029705	279.3967079	-1.204097759	0.282200935	-4.266809961	1.98E-05	0.000187133
ENSMUSG00000022797	3414.595888	-1.202361325	0.240013625	-5.009554453	5.46E-07	7.05E-06
ENSMUSG00000048550	84.9354801	-1.201657325	0.388529421	-3.092834826	0.001982544	0.010790024
ENSMUSG00000019080	57.66868562	-1.201270309	0.45252717	-2.654581623	0.007940683	0.034548272
ENSMUSG00000024782	500.2911734	-1.201172571	0.258174929	-4.652553121	3.28E-06	3.67E-05
ENSMUSG00000026113	87.14344687	-1.199917104	0.40519782	-2.961311846	0.003063316	0.015606969
ENSMUSG00000025026	235.4368424	-1.198889313	0.294028867	-4.077454446	4.55E-05	0.000400776
ENSMUSG00000015605	145.6753581	-1.1984133	0.372764403	-3.214934933	0.001304741	0.007618864
ENSMUSG00000030265	162.8994441	-1.197797688	0.32183203	-3.721810069	0.0001978	0.001485395
ENSMUSG00000027160	50.33916545	-1.197622056	0.475413946	-2.519114272	0.011765047	0.047242885
ENSMUSG00000059013	80.18102695	-1.197364457	0.425914633	-2.811278048	0.004934512	0.023295725
ENSMUSG00000004996	211.9797378	-1.196635563	0.343353074	-3.485145915	0.000491869	0.003268063
ENSMUSG00000026526	961.4159762	-1.195818809	0.23729123	-5.039456414	4.67E-07	6.12E-06
ENSMUSG00000027883	287.2725116	-1.193586395	0.280628603	-4.253259945	2.11E-05	0.000197901
ENSMUSG00000031904	179.1081505	-1.190843346	0.316623191	-3.761074302	0.000169185	0.001295353
ENSMUSG00000039206	426.4818904	-1.190417204	0.290210648	-4.101907395	4.10E-05	0.000363478
ENSMUSG00000050989	60.14734658	-1.189809343	0.439858752	-2.704980489	0.00683084	0.030470803
ENSMUSG00000042498	135.2118724	-1.189650704	0.371304323	-3.203977517	0.001355431	0.007880716
ENSMUSG00000032123	55.40110134	-1.188910769	0.466197588	-2.550229346	0.010765207	0.043962208
ENSMUSG00000040964	558.0148059	-1.188371635	0.28707837	-4.139537345	3.48E-05	0.000313572
ENSMUSG00000036944	61.41579732	-1.186274186	0.463673631	-2.55842495	0.010514751	0.043209795
ENSMUSG00000029414	112.0582127	-1.18472656	0.370017549	-3.201811812	0.001365662	0.007921491
ENSMUSG00000030201	434.0835452	-1.182733759	0.271232834	-4.360584743	1.30E-05	0.000127896
ENSMUSG00000036850	296.89665	-1.181963641	0.2981933	-3.963749522	7.38E-05	0.00061

	05		23			9594
ENSMUSG00000030844	423.69567 15	-1.181855011	0.3087756 62	-3.827552351	0.000129 424	0.00101 9399
ENSMUSG00000016757	132.67210 03	-1.181145542	0.3599530 17	-3.28138809	0.001032 975	0.00622 8299
ENSMUSG00000102715	68.160998 42	-1.180839395	0.4287678 76	-2.754029538	0.005886 646	0.02696 4063
ENSMUSG00000073557	161.53615 92	-1.180188814	0.3282103 31	-3.595830788	0.000323 358	0.00226 334
ENSMUSG00000074909	101.19312 56	-1.17999133	0.3785234 24	-3.117353521	0.001824 826	0.01007 6245
ENSMUSG00000026730	141.28552 29	-1.177488076	0.3410547 73	-3.45248966	0.000555 439	0.00363 5286
ENSMUSG00000007379	106.59478 46	-1.176819149	0.3693582 58	-3.186118421	0.001441 956	0.00829 639
ENSMUSG00000061410	160.50762 48	-1.176734758	0.3631021 46	-3.240781616	0.001192 025	0.00705 6889
ENSMUSG00000027173	136.48370 84	-1.17558723	0.3497416 16	-3.361302101	0.000775 759	0.00486 1354
ENSMUSG00000027984	371.71149 04	-1.172881773	0.2837079 27	-4.134117039	3.56E-05	0.00032 0222
ENSMUSG00000030064	197.90030 9	-1.171790297	0.3217365 74	-3.642079862	0.000270 444	0.00193 8513
ENSMUSG00000065954	755.22908 7	-1.171311221	0.2398087 68	-4.88435527	1.04E-06	1.28E-05
ENSMUSG00000021144	77.212275 61	-1.171269755	0.4201361 78	-2.787833604	0.005306 179	0.02477 8538
ENSMUSG00000027405	369.27727 28	-1.166597295	0.2700673 5	-4.319653217	1.56E-05	0.00015 0737
ENSMUSG00000060098	396.65098 86	-1.163951664	0.2928036 62	-3.975195032	7.03E-05	0.00059 3082
ENSMUSG00000085882	54.860063 63	-1.163681628	0.4512289 37	-2.578916227	0.009911 082	0.04116 1023
ENSMUSG00000049792	116.37636 76	-1.162826028	0.3972883 36	-2.926907039	0.003423 512	0.01720 1439
ENSMUSG00000023150	700.90157 99	-1.162821665	0.2568521 89	-4.527201703	5.98E-06	6.31E-05
ENSMUSG00000081664	58.038666 77	-1.160184538	0.4431925 75	-2.617788751	0.008850 158	0.03770 3267
ENSMUSG00000015749	307.76430 76	-1.159792734	0.2921588 9	-3.969732822	7.20E-05	0.00060 6097
ENSMUSG00000022186	3361.0915 03	-1.157825519	0.2358233 51	-4.909715327	9.12E-07	1.13E-05
ENSMUSG00000024451	119.69110 38	-1.157600699	0.3762670 08	-3.076540524	0.002094 178	0.01128 6164
ENSMUSG00000058799	343.16379 4	-1.157399364	0.2747927 13	-4.211899765	2.53E-05	0.00023 3864
ENSMUSG00000000631	266.45485 36	-1.155702349	0.3108029 18	-3.718441111	0.000200 456	0.00150 3694
ENSMUSG00000039018	65.313522 1	-1.154604985	0.4321943 2	-2.671495048	0.007551 419	0.03305 4038
ENSMUSG00000026116	160.47152 37	-1.154335797	0.3207329 81	-3.599055489	0.000319 375	0.00224 3483
ENSMUSG00000052102	231.25131	-1.153249349	0.3113584	-3.703928279	0.000212	0.00157

	71		45		286	349
ENSMUSG00000023942	281.34506 64	-1.152539087	0.3316643 12	-3.475016895	0.000510 821	0.00337 7137
ENSMUSG00000005034	827.70378 3	-1.149023393	0.2368413 01	-4.851448582	1.23E-06	1.48E-05
ENSMUSG00000015202	168.29367 26	-1.147637883	0.3217339 32	-3.567040239	0.000361 036	0.00249 3951
ENSMUSG00000000561	220.59985 69	-1.145589483	0.2954558 06	-3.877363243	0.000105 595	0.00084 8783
ENSMUSG00000038286	102.74723 86	-1.145350621	0.3673762 37	-3.117650262	0.001822 99	0.01007 0164
ENSMUSG00000031666	173.26335 63	-1.1451423	0.3138117 74	-3.649137458	0.000263 122	0.00188 9985
ENSMUSG00000023104	680.99090 68	-1.145031239	0.2562130 71	-4.469058645	7.86E-06	8.10E-05
ENSMUSG00000003038	58.359146 96	-1.144037245	0.4524849 95	-2.528342943	0.011460 235	0.04624 9269
ENSMUSG00000050244	590.61056 59	-1.143318436	0.2476498 11	-4.616673977	3.90E-06	4.30E-05
ENSMUSG00000028102	64.884453 54	-1.142862942	0.4313330 1	-2.649606955	0.008058 546	0.03496 5377
ENSMUSG00000010461	144.50613 45	-1.142027725	0.3355228 93	-3.403725198	0.000664 736	0.00424 5203
ENSMUSG00000053898	606.29784 61	-1.141261759	0.2548146 3	-4.478792124	7.51E-06	7.78E-05
ENSMUSG00000025486	108.46484 16	-1.138451148	0.4203090 14	-2.708605122	0.006756 67	0.03023 1853
ENSMUSG00000024082	195.87106 77	-1.138336717	0.3130693 42	-3.636052985	0.000276 847	0.00197 7535
ENSMUSG00000040022	76.730624 28	-1.137691606	0.4097005 37	-2.776885807	0.005488 246	0.02547 1184
ENSMUSG00000024222	77.885171 46	-1.136012224	0.4292625 93	-2.646427253	0.008134 7	0.03519 9136
ENSMUSG00000004872	89.778093 64	-1.135873343	0.3878107 24	-2.928937426	0.003401 229	0.01711 3581
ENSMUSG00000000149	417.80712 03	-1.132927254	0.2920835 44	-3.878778104	0.000104 982	0.00084 5349
ENSMUSG00000029413	168.77034 98	-1.131263542	0.3386978 38	-3.340037682	0.000837 67	0.00518 3454
ENSMUSG00000027430	104.14510 29	-1.130664431	0.3667337 51	-3.083066195	0.002048 796	0.01108 0799
ENSMUSG00000019528	1077.8055 74	-1.129844862	0.2378861 01	-4.74952028	2.04E-06	2.39E-05
ENSMUSG00000031749	135.98424 28	-1.12820217	0.3546587 08	-3.181092542	0.001467 208	0.00841 6944
ENSMUSG00000046873	100.20178 8	-1.127666566	0.4001937 67	-2.817801424	0.004835 37	0.02289 867
ENSMUSG00000072640	89.486005 01	-1.127509803	0.3978821 5	-2.833778303	0.004600 123	0.02197 4917
ENSMUSG00000001151	210.46523 73	-1.126084212	0.3112408 72	-3.618047345	0.000296 834	0.00210 4558
ENSMUSG00000032340	433.53331 22	-1.12550319	0.2601758 41	-4.325932751	1.52E-05	0.00014 7111
ENSMUSG00000034210	101.01543	-1.12529567	0.3758399	-2.994082323	0.002752	0.01427

	88		23		717	9327
ENSMUSG00000015889	203.5160518	-1.125132561	0.304747496	-3.692015774	0.000222484	0.001638441
ENSMUSG00000035024	93.40136466	-1.124878871	0.376751644	-2.985730489	0.002829019	0.014608779
ENSMUSG00000028278	189.4099791	-1.121995754	0.332095651	-3.378531913	0.00072874	0.004606724
ENSMUSG00000032400	76.51069799	-1.121406465	0.403719002	-2.777690572	0.005474673	0.02543403
ENSMUSG00000030279	111.1770457	-1.121045924	0.363537776	-3.083712332	0.002044352	0.011061129
ENSMUSG00000039197	468.9938086	-1.117975661	0.257195982	-4.346785102	1.38E-05	0.000135236
ENSMUSG00000033624	112.413603	-1.117035299	0.371328669	-3.008211845	0.002627899	0.013741073
ENSMUSG00000020834	58.73509082	-1.116402068	0.4440814	-2.513957953	0.011938467	0.047771295
ENSMUSG00000060152	195.2040179	-1.114740332	0.359586596	-3.10006086	0.001934809	0.010564817
ENSMUSG00000031756	76.37716681	-1.114114463	0.420968703	-2.646549388	0.008131763	0.035199136
ENSMUSG00000033554	153.7075403	-1.112487283	0.32865785	-3.384940549	0.000711937	0.004515082
ENSMUSG00000033031	146.2007713	-1.112148504	0.348697332	-3.189437955	0.001425497	0.008222408
ENSMUSG00000029191	435.7643272	-1.110859769	0.261311577	-4.251092815	2.13E-05	0.00019928
ENSMUSG00000074238	357.3651821	-1.108814058	0.284806173	-3.893223411	9.89E-05	0.000801722
ENSMUSG00000002985	873.5268684	-1.108005872	0.259278459	-4.273420466	1.92E-05	0.000181943
ENSMUSG00000021190	8992.005091	-1.107118341	0.219532727	-5.043067417	4.58E-07	6.03E-06
ENSMUSG00000029672	189.2422612	-1.107003463	0.308198392	-3.591853466	0.000328334	0.002294658
ENSMUSG00000050751	182.5026227	-1.105225789	0.336185411	-3.287548334	0.001010638	0.006112444
ENSMUSG00000029571	579.7685876	-1.105138243	0.254717693	-4.338678754	1.43E-05	0.000139623
ENSMUSG00000037544	101.0457648	-1.104574252	0.380420447	-2.90356173	0.003689442	0.018267271
ENSMUSG00000018196	117.0616333	-1.10378691	0.356010842	-3.100430605	0.001932395	0.010559598
ENSMUSG00000034880	349.721964	-1.103635068	0.280464137	-3.935030986	8.32E-05	0.000687614
ENSMUSG00000030035	108.3136275	-1.102802237	0.376351504	-2.930245335	0.003386945	0.01706547
ENSMUSG00000026879	5709.294686	-1.102354141	0.240499927	-4.583594493	4.57E-06	4.95E-05
ENSMUSG00000055435	144.1615627	-1.101662299	0.340586887	-3.234599871	0.001218133	0.007193716
ENSMUSG00000018659	96.39071006	-1.101627882	0.382905042	-2.877026319	0.004014421	0.019586202
ENSMUSG00000096210	118.63567	-1.100234997	0.3667389	-3.000049166	0.002699	0.01403

	44		89		36	9747
ENSMUSG00000027203	221.7363151	-1.098677511	0.300258696	-3.659103055	0.0002531	0.00183143
ENSMUSG00000071337	104.8584988	-1.0975461	0.36282932	-3.024965295	0.002486617	0.013057119
ENSMUSG00000027490	88.02822269	-1.09675756	0.412538155	-2.658560298	0.007847531	0.034208145
ENSMUSG00000090176	101.7571104	-1.095716894	0.394544457	-2.777169655	0.005483455	0.025462683
ENSMUSG00000041431	104.6425211	-1.095198899	0.404512069	-2.707456671	0.006780092	0.030306967
ENSMUSG00000001999	1117.746488	-1.093263306	0.245454232	-4.454041379	8.43E-06	8.65E-05
ENSMUSG00000079671	166.8145637	-1.09184974	0.321522858	-3.395869731	0.000684109	0.004348662
ENSMUSG00000043252	116.550449	-1.090963654	0.351809062	-3.101010666	0.001928613	0.010551556
ENSMUSG00000039515	574.5957177	-1.090562351	0.287451145	-3.793905047	0.000148296	0.001153933
ENSMUSG00000019990	71.10394305	-1.09055212	0.430945101	-2.53060568	0.011386578	0.046033347
ENSMUSG00000001918	554.1474179	-1.089908498	0.309714652	-3.519073091	0.000433057	0.002920793
ENSMUSG00000073792	82.00929905	-1.089210259	0.394225685	-2.762910436	0.005728848	0.02636463
ENSMUSG00000038545	111.1522387	-1.088867986	0.369061719	-2.950368275	0.003173954	0.016104788
ENSMUSG00000036632	244.6686708	-1.088845664	0.291305438	-3.737814404	0.000185627	0.001403983
ENSMUSG00000047798	88.02202885	-1.085270948	0.384492865	-2.822603612	0.004763543	0.022605385
ENSMUSG00000069631	72.77707178	-1.084387592	0.419323546	-2.586040309	0.009708558	0.040491717
ENSMUSG00000087579	295.4414655	-1.083277064	0.303466951	-3.569670641	0.00035743	0.002472327
ENSMUSG00000018574	191.7243074	-1.081338501	0.314304899	-3.440412489	0.000580828	0.003778002
ENSMUSG00000038046	85.5178514	-1.080968868	0.392916327	-2.751142657	0.005938777	0.027166537
ENSMUSG00000028581	846.3016525	-1.080528801	0.237885081	-4.542230202	5.57E-06	5.92E-05
ENSMUSG00000034192	221.8391504	-1.080001686	0.295751616	-3.651718628	0.000260491	0.001874066
ENSMUSG00000005299	175.1595417	-1.07619984	0.316390839	-3.401488622	0.000670199	0.004272463
ENSMUSG00000005949	128.1517927	-1.075664748	0.343120883	-3.134943986	0.001718869	0.009595562
ENSMUSG00000025728	69.37516157	-1.074229654	0.414150588	-2.593814149	0.009491778	0.03981232
ENSMUSG00000039873	153.5357588	-1.073682243	0.379153196	-2.831790035	0.004628823	0.02208076
ENSMUSG00000025403	688.2542543	-1.072344432	0.274409479	-3.907825764	9.31E-05	0.000760186
ENSMUSG00000031432	174.60825	-1.072090203	0.3325646	-3.223705083	0.001265	0.00742

	16		04		437	4147
ENSMUSG00000038174	408.20379 09	-1.071615094	0.2785924 82	-3.846532709	0.000119 801	0.00095 1811
ENSMUSG00000044042	199.05723 41	-1.069835739	0.3115022 07	-3.434440316	0.000593 779	0.00384 9451
ENSMUSG00000006611	381.46573 07	-1.069288521	0.2649905 05	-4.035195601	5.46E-05	0.00047 3229
ENSMUSG00000028969	183.61341 89	-1.068474723	0.3236542 63	-3.301284259	0.000962 433	0.00585 1884
ENSMUSG00000006288	98.039121 13	-1.068322406	0.3863858 07	-2.764911098	0.005693 831	0.02623 8721
ENSMUSG00000027630	628.57296 54	-1.067999442	0.2482241 69	-4.302560242	1.69E-05	0.00016 1472
ENSMUSG00000023094	95.750716 12	-1.067814208	0.3876987 41	-2.754236974	0.005882 916	0.02695 5987
ENSMUSG00000026918	78.141942 3	-1.067739473	0.4005869 94	-2.665437192	0.007688 829	0.03358 0435
ENSMUSG00000038855	198.95903 55	-1.066291585	0.3181914 32	-3.351100874	0.000804 91	0.00501 1944
ENSMUSG00000022519	74.948917 46	-1.066094772	0.4232681 61	-2.518721865	0.011778 165	0.04725 4027
ENSMUSG00000019876	532.55753 85	-1.06557024	0.2740166 35	-3.888706393	0.000100 78	0.00081 438
ENSMUSG00000005469	258.10477 51	-1.065499239	0.2955646 64	-3.604961511	0.000312 199	0.00219 871
ENSMUSG00000022248	80.297053 1	-1.063347765	0.4025302 89	-2.641659009	0.008250 107	0.03555 5886
ENSMUSG00000065979	138.30870 38	-1.063325581	0.3370592 53	-3.154714106	0.001606 555	0.00909 0764
ENSMUSG00000038759	546.35562 35	-1.063017968	0.2573789 09	-4.130167354	3.62E-05	0.00032 556
ENSMUSG00000020399	203.01654 26	-1.062521653	0.3055664 61	-3.477219491	0.000506 643	0.00335 5989
ENSMUSG00000024169	81.915206 19	-1.060137732	0.3919051 99	-2.705087185	0.006828 646	0.03047 0803
ENSMUSG00000045679	184.11560 6	-1.059913095	0.3327822 65	-3.185004749	0.001447 516	0.00832 1397
ENSMUSG00000033068	125.19274 15	-1.05621234	0.3548029 21	-2.976898658	0.002911 802	0.01495 7187
ENSMUSG00000089774	131.64551 28	-1.055605787	0.3595598 5	-2.935827758	0.003326 59	0.01683 8226
ENSMUSG00000014633	111.79600 61	-1.055071235	0.3609755 52	-2.922832947	0.003468 626	0.01737 4822
ENSMUSG00000031264	179.61683 17	-1.054728584	0.3371075 51	-3.128759888	0.001755 457	0.00976 058
ENSMUSG00000024158	313.86848 91	-1.054607057	0.2755571 2	-3.827181303	0.000129 619	0.00102 035
ENSMUSG00000027634	189.33055 74	-1.054379387	0.3099778 09	-3.401467315	0.000670 251	0.00427 2463
ENSMUSG00000038843	300.18320 9	-1.054294651	0.2770901 12	-3.804880095	0.000141 873	0.00110 7902
ENSMUSG00000018841	109.96522 44	-1.053730814	0.3661027 62	-2.878237812	0.003999 035	0.01952 5038
ENSMUSG00000022814	280.73112	-1.053416613	0.2855218	-3.689443092	0.000224	0.00165

	41		49		745	2434
ENSMUSG00000047547	312.217217	-1.053358493	0.303512476	-3.470560773	0.000519373	0.00342706
ENSMUSG00000074886	180.6979916	-1.052302552	0.361751401	-2.908910786	0.003626903	0.017996637
ENSMUSG00000020029	224.2256211	-1.051376406	0.301676843	-3.485108087	0.000491938	0.003268063
ENSMUSG00000024645	111.0372254	-1.048851806	0.385462167	-2.721023992	0.006508004	0.029272134
ENSMUSG00000051007	123.2101639	-1.046714172	0.361459801	-2.895796905	0.003781971	0.018644572
ENSMUSG00000018932	123.1044461	-1.046650632	0.346037668	-3.024672542	0.002489025	0.013064757
ENSMUSG00000040940	84.9952319	-1.045853146	0.397410101	-2.631672278	0.008496578	0.036438928
ENSMUSG00000006315	385.4755066	-1.044616424	0.265381586	-3.936280733	8.28E-05	0.000685284
ENSMUSG00000006289	81.45169405	-1.043730776	0.395036632	-2.642111368	0.008239096	0.0355196
ENSMUSG00000000594	342.9609059	-1.043653702	0.279188386	-3.738170184	0.000185364	0.001402772
ENSMUSG00000029587	132.6262702	-1.041724931	0.361823211	-2.879099238	0.003988128	0.019478723
ENSMUSG00000052656	162.528847	-1.040806479	0.320565877	-3.24677876	0.001167191	0.00694286
ENSMUSG00000030786	868.7203991	-1.040690247	0.240505477	-4.327095824	1.51E-05	0.000146544
ENSMUSG00000075033	144.4693171	-1.040033486	0.332233599	-3.130428376	0.001745516	0.009720557
ENSMUSG00000036766	72.5162711	-1.039747766	0.412781901	-2.518879252	0.011772902	0.047254027
ENSMUSG00000049091	600.0591952	-1.039629364	0.262900049	-3.954466229	7.67E-05	0.000639837
ENSMUSG00000028044	157.4154957	-1.039133332	0.332495077	-3.1252593	0.001776484	0.009864956
ENSMUSG00000029263	134.7857917	-1.038799414	0.339756614	-3.057481067	0.002232057	0.011898229
ENSMUSG00000051343	305.9959813	-1.038372866	0.325077477	-3.194231964	0.001402034	0.008114391
ENSMUSG00000022360	509.8816678	-1.037908688	0.261879358	-3.963308513	7.39E-05	0.00062036
ENSMUSG00000039660	80.90302796	-1.037094067	0.400845537	-2.587266093	0.009674085	0.040372515
ENSMUSG00000021094	335.5601439	-1.037055431	0.283595742	-3.656808891	0.000255375	0.001843999
ENSMUSG00000037894	240.8055026	-1.036903308	0.333092629	-3.112957827	0.001852225	0.010206973
ENSMUSG00000021054	376.6894135	-1.0364389	0.267254277	-3.878100324	0.000105275	0.000846712
ENSMUSG00000023022	398.9009642	-1.033586005	0.269847465	-3.830260198	0.000128008	0.001009412
ENSMUSG00000054452	386.0147236	-1.033459654	0.276248906	-3.741045236	0.000183257	0.001388353
ENSMUSG00000038323	105.79067	-1.032212893	0.3642128	-2.834092791	0.004595	0.02197

	36		08		599	0544
ENSMUSG00000026134	193.59052 94	-1.0302165	0.3074113 46	-3.351263745	0.000804 436	0.00501 1274
ENSMUSG00000033083	163.72928 33	-1.028985095	0.3203884 59	-3.211679658	0.001319 614	0.00769 9155
ENSMUSG00000014551	164.23329 55	-1.028594404	0.3292674 32	-3.123887467	0.001784 787	0.00990 3039
ENSMUSG00000028292	81.198696 72	-1.028209923	0.4029502 09	-2.551704649	0.010719 735	0.04379 4538
ENSMUSG00000030850	663.42486 29	-1.027483623	0.2427077 26	-4.233419513	2.30E-05	0.00021 4003
ENSMUSG00000078515	448.35336 76	-1.026875548	0.2774860 67	-3.700638228	0.000215 058	0.00159 1452
ENSMUSG00000042699	410.13511 76	-1.026622345	0.2767037 63	-3.710185709	0.000207 107	0.00154 345
ENSMUSG00000016637	79.816069 23	-1.026103012	0.4037534 98	-2.541409592	0.011040 65	0.04489 2903
ENSMUSG00000026974	141.71040 72	-1.025516546	0.3405888 33	-3.011010483	0.002603 799	0.01362 5453
ENSMUSG00000024640	3713.8946 85	-1.025380187	0.2175316 92	-4.713704825	2.43E-06	2.81E-05
ENSMUSG00000018446	228.93426 71	-1.025099045	0.3176265 82	-3.227371714	0.001249 33	0.00733 908
ENSMUSG00000037936	337.72065 78	-1.024022803	0.2732776 51	-3.747188246	0.000178 828	0.00136 0064
ENSMUSG00000038172	81.240948 1	-1.023626443	0.4062546 19	-2.519667213	0.011746 583	0.04718 2568
ENSMUSG00000003848	124.11730 21	-1.023558324	0.3567178 2	-2.869378167	0.004112 797	0.02000 919
ENSMUSG00000022471	266.13345 1	-1.022547878	0.2834554 1	-3.60743821	0.000309 235	0.00218 0076
ENSMUSG00000030541	1532.7167 22	-1.022091069	0.2456839 58	-4.160186434	3.18E-05	0.00028 8611
ENSMUSG00000002871	109.16992 24	-1.021295728	0.3991847 56	-2.55845373	0.010513 881	0.04320 9795
ENSMUSG00000058979	81.023603 75	-1.020676413	0.4085521 07	-2.498277199	0.012479 856	0.04956 1412
ENSMUSG00000041975	175.13205 75	-1.018671224	0.3140816 4	-3.243332605	0.001181 402	0.00699 7025
ENSMUSG00000021311	172.16096 72	-1.017703043	0.3353335 02	-3.034898204	0.002406 169	0.01271 2623
ENSMUSG00000026469	765.94625 59	-1.016829042	0.2383182 18	-4.266686162	1.98E-05	0.00018 7133
ENSMUSG00000049299	213.85607 99	-1.015333047	0.3091233 06	-3.284556767	0.001021 429	0.00616 1394
ENSMUSG00000014748	119.45530 64	-1.015207245	0.3525097 97	-2.879940508	0.003977 502	0.01943 3749
ENSMUSG00000009995	171.44051 51	-1.01483777	0.3416254 78	-2.970615007	0.002972 041	0.01522 6849
ENSMUSG00000003099	185.50842 67	-1.014508901	0.3132464 07	-3.238692857	0.001200 788	0.00710 2633
ENSMUSG00000076437	144.26184 04	-1.013335504	0.3626339 11	-2.794376013	0.005199 998	0.02435 6107
ENSMUSG00000039062	218.58418	-1.01143593	0.3033973	-3.333700782	0.000856	0.00528

	59		34		988	8167
ENSMUSG00000027597	77.68040662	-1.010740446	0.403333525	-2.505966859	0.012211707	0.048682418
ENSMUSG00000015176	497.3675251	-1.010210706	0.265107611	-3.810568483	0.000138648	0.001084569
ENSMUSG00000036918	306.3860566	-1.006976151	0.338024103	-2.979006949	0.002891842	0.014882725
ENSMUSG00000019302	740.9955684	-1.006754946	0.241250708	-4.173065249	3.01E-05	0.000273731
ENSMUSG00000029247	952.3801528	-1.006706712	0.24878648	-4.046468739	5.20E-05	0.0004536
ENSMUSG00000026578	153.5452203	-1.005733814	0.331922966	-3.030021774	0.002445361	0.012889875
ENSMUSG00000000184	235.1878163	-1.005017598	0.291028354	-3.453332245	0.000553707	0.00362568
ENSMUSG00000026709	238.3848376	-1.005004504	0.294682003	-3.41047127	0.000648507	0.004153173
ENSMUSG00000027469	227.2617903	-1.004210231	0.313157873	-3.206721974	0.001342567	0.007816431
ENSMUSG00000027357	456.3605391	-1.002125913	0.257445176	-3.892579887	9.92E-05	0.000802903
ENSMUSG00000029208	353.3185865	-1.001714466	0.276782699	-3.619136852	0.000295587	0.002097889
ENSMUSG00000023110	204.777179	-1.001611655	0.326582467	-3.066948645	0.00216256	0.011599955
ENSMUSG00000029616	3282.444978	-1.001278914	0.224649181	-4.457077958	8.31E-06	8.54E-05
ENSMUSG00000041650	164.2335531	1.001496604	0.321611142	3.113998471	0.001845704	0.01018266
ENSMUSG00000009035	213.5483852	1.001586153	0.326772916	3.065083132	0.002176095	0.01164521
ENSMUSG00000052593	2221.262879	1.001661252	0.22996234	4.355762123	1.33E-05	0.000130369
ENSMUSG00000026209	1016.519509	1.002540574	0.280729257	3.571200897	0.000355348	0.00245962
ENSMUSG00000054277	560.617898	1.0028474	0.25329872	3.959149109	7.52E-05	0.000629721
ENSMUSG00000026289	444.1689091	1.002978733	0.261018217	3.842562202	0.000121757	0.000965109
ENSMUSG00000063362	266.3096876	1.005495232	0.293825652	3.422081174	0.000621438	0.004019255
ENSMUSG00000030591	6325.446858	1.006012667	0.233184428	4.314236058	1.60E-05	0.000153922
ENSMUSG00000033793	5335.100924	1.00728184	0.256469141	3.927497228	8.58E-05	0.000707798
ENSMUSG00000038543	272.8302694	1.008130945	0.328863934	3.065495604	0.002173096	0.011642798
ENSMUSG00000026869	1658.632615	1.009899361	0.22796547	4.43005408	9.42E-06	9.58E-05
ENSMUSG00000014349	676.4668158	1.010776042	0.249311835	4.054264185	5.03E-05	0.000439577
ENSMUSG00000020089	922.9192392	1.010924307	0.267627942	3.777349628	0.000158506	0.001221094
ENSMUSG00000058298	280.46074	1.012196552	0.3028120	3.342656157	0.000829	0.00514

	33		46		807	1274
ENSMUSG00000007987	350.51954<br>83	1.012558453	0.2703150<br>99	3.745844965	0.000179<br>788	0.00136<br>5848
ENSMUSG00000024867	127.16943<br>58	1.013034562	0.3607166<br>41	2.808394306	0.004978<br>922	0.02344<br>8842
ENSMUSG00000007837	166.45215<br>91	1.013442795	0.3492505<br>45	2.901764393	0.003710<br>675	0.01835<br>9134
ENSMUSG00000051510	925.26329<br>73	1.014609943	0.2564312<br>03	3.956655552	7.60E-05	0.00063<br>5551
ENSMUSG00000030203	176.71186<br>02	1.016320156	0.3275815<br>84	3.102494783	0.001918<br>969	0.01052<br>3999
ENSMUSG00000103653	168.04976<br>01	1.016366446	0.3233572<br>11	3.143169265	0.001671<br>292	0.00937<br>5789
ENSMUSG00000080396	134.62594<br>76	1.016526031	0.3668646<br>04	2.770847939	0.005591<br>053	0.02586<br>9474
ENSMUSG00000051029	135.50068<br>23	1.017697237	0.3595743<br>78	2.830282963	0.004650<br>685	0.02217<br>7328
ENSMUSG00000021959	82.592974<br>56	1.017964885	0.3970336<br>36	2.56392606	0.010349<br>559	0.04268<br>4534
ENSMUSG00000021280	166.93485<br>07	1.018707734	0.3522779<br>28	2.891772813	0.003830<br>748	0.01883<br>9192
ENSMUSG00000022280	412.17850<br>68	1.018807606	0.2620516<br>41	3.887812346	0.000101<br>152	0.00081<br>6903
ENSMUSG00000026111	914.26092<br>28	1.021027359	0.2604450<br>36	3.920317988	8.84E-05	0.00072<br>7469
ENSMUSG00000031987	459.13486<br>7	1.021135129	0.2765245<br>83	3.692746294	0.000221<br>845	0.00163<br>4618
ENSMUSG00000084013	735.11669<br>79	1.021982499	0.2481025<br>21	4.119194341	3.80E-05	0.00033<br>9234
ENSMUSG00000028191	727.02696<br>9	1.022409047	0.2477055<br>05	4.127518474	3.67E-05	0.00032<br>8686
ENSMUSG00000064145	450.66538<br>76	1.022587297	0.2610951<br>22	3.916531611	8.98E-05	0.00073<br>6773
ENSMUSG00000003721	741.49826<br>56	1.023981718	0.2553892<br>73	4.009493853	6.08E-05	0.00052<br>121
ENSMUSG00000034088	3072.9449<br>51	1.024675183	0.2292347<br>07	4.46998273	7.82E-06	8.09E-05
ENSMUSG00000049878	430.11346<br>69	1.024747345	0.2779904<br>98	3.686267523	0.000227<br>567	0.00166<br>8842
ENSMUSG00000023330	208.67714<br>9	1.025837518	0.3219159<br>95	3.186662155	0.001439<br>248	0.00828<br>4289
ENSMUSG00000037280	2026.2016<br>73	1.027287501	0.2391496<br>37	4.295584617	1.74E-05	0.00016<br>6055
ENSMUSG00000028458	138.18755<br>24	1.028281893	0.4046354<br>05	2.541255366	0.011045<br>521	0.04489<br>2903
ENSMUSG00000042682	596.35296<br>11	1.028591708	0.2586507<br>14	3.976759592	6.99E-05	0.00059<br>0284
ENSMUSG00000024646	2674.6832<br>74	1.029045061	0.2571257<br>31	4.002108447	6.28E-05	0.00053<br>5745
ENSMUSG00000018102	2069.465	1.029258024	0.2692465<br>29	3.822734602	0.000131<br>98	0.00103<br>6552
ENSMUSG00000074466	83.061606<br>97	1.029299087	0.3967216<br>88	2.594511762	0.009472<br>537	0.03974<br>9272
ENSMUSG00000021559	311.62277	1.029470957	0.2831136	3.63624611	0.000276	0.00197

	7		63		64	7535
ENSMUSG00000032316	137.8599495	1.03010562	0.34000316	3.029694257	0.002448014	0.012898898
ENSMUSG00000083161	154.7958243	1.03040229	0.333415697	3.090443247	0.00199858	0.010852036
ENSMUSG00000028920	362.500647	1.032265953	0.272778726	3.78426122	0.000154166	0.001193022
ENSMUSG00000020048	5539.755207	1.032353442	0.298462916	3.458900209	0.000542386	0.003561762
ENSMUSG00000004535	2241.705446	1.033546798	0.281782577	3.667887525	0.000244563	0.001780942
ENSMUSG00000089542	597.472119	1.033776442	0.263833126	3.918296614	8.92E-05	0.000732277
ENSMUSG00000025793	321.3502465	1.035627528	0.294059972	3.521824214	0.000428588	0.002894924
ENSMUSG00000029780	244.004135	1.036202007	0.352502226	2.939561599	0.003286769	0.016669873
ENSMUSG00000022892	2217.174531	1.038858739	0.222815305	4.662420914	3.13E-06	3.52E-05
ENSMUSG00000038822	209.2800177	1.039949926	0.31762788	3.27411412	0.001059938	0.006382447
ENSMUSG00000024539	369.1199937	1.041677716	0.302084545	3.448298603	0.00056413	0.003683374
ENSMUSG00000029708	337.0627424	1.041940224	0.296916443	3.509203507	0.000449451	0.003019473
ENSMUSG00000023010	6462.624857	1.042761382	0.224885561	4.636853411	3.54E-06	3.94E-05
ENSMUSG00000058407	1148.090535	1.042849395	0.280045525	3.723856671	0.000196202	0.001474207
ENSMUSG00000027171	324.2367807	1.043273858	0.293489715	3.554720342	0.000378381	0.002594155
ENSMUSG00000024423	1393.497547	1.043731578	0.250578232	4.165292286	3.11E-05	0.000282601
ENSMUSG00000070730	423.4451042	1.044710668	0.264038647	3.956658159	7.60E-05	0.000635551
ENSMUSG00000092072	170.4462342	1.045205326	0.378425922	2.761981316	0.005745177	0.026422029
ENSMUSG00000041440	393.9281733	1.04529994	0.269521803	3.878350203	0.000105167	0.00084634
ENSMUSG00000038495	661.7968535	1.046072975	0.251945901	4.151974582	3.30E-05	0.000298772
ENSMUSG00000083678	269.3117787	1.046425	0.300557609	3.481612075	0.000498405	0.00330942
ENSMUSG00000060647	141.2723253	1.049808739	0.386841765	2.713793683	0.006651758	0.029850145
ENSMUSG00000039908	103.6915152	1.049853269	0.371182314	2.828403267	0.004678084	0.022276976
ENSMUSG00000052906	137.1407584	1.050132593	0.370830314	2.831841287	0.004628081	0.02208076
ENSMUSG00000030720	389.1643582	1.051367626	0.273479171	3.844415719	0.00012084	0.000958953
ENSMUSG00000020024	400.550333	1.052964012	0.307773459	3.421230719	0.000623384	0.004028044
ENSMUSG00000024773	295.74317	1.053440551	0.3149287	3.345012017	0.000822	0.00510

	54		79		79	9138
ENSMUSG00000033319	320.9929777	1.053587644	0.280814314	3.751901496	0.000175498	0.001335557
ENSMUSG00000030652	437.883888	1.053775198	0.284357962	3.705805144	0.00021072	0.001565894
ENSMUSG00000020109	260.946457	1.054209693	0.287211212	3.670503271	0.000242073	0.001763751
ENSMUSG00000020464	1023.695051	1.054342176	0.305727924	3.448628976	0.00056344	0.003680624
ENSMUSG00000028759	919.8166796	1.054554852	0.241968127	4.35823868	1.31E-05	0.000128996
ENSMUSG00000057342	312.834873	1.054598765	0.335741441	3.141103946	0.001683123	0.009434437
ENSMUSG00000029925	120.5427661	1.054876596	0.362058213	2.913555216	0.003573386	0.017801985
ENSMUSG00000023827	168.7119761	1.05652025	0.335776845	3.146495259	0.001652399	0.009296442
ENSMUSG00000022489	101.3587251	1.056878468	0.376216285	2.80923105	0.004965999	0.023401959
ENSMUSG00000011752	114.9182816	1.056899498	0.409469949	2.581140571	0.009847448	0.040921549
ENSMUSG00000048911	81.40579267	1.057171093	0.400369515	2.64048848	0.008278661	0.035656519
ENSMUSG00000026171	385.1288547	1.057188488	0.281177311	3.759864134	0.000170006	0.001299458
ENSMUSG00000020948	189.4051712	1.059020639	0.333635408	3.174185393	0.001502577	0.008576766
ENSMUSG00000035673	326.7125225	1.06039231	0.315880811	3.356938042	0.000788108	0.004931971
ENSMUSG00000026456	2641.164695	1.060518508	0.227024298	4.671387666	2.99E-06	3.37E-05
ENSMUSG00000029402	165.1041686	1.063011811	0.332955805	3.192651383	0.00140973	0.008152048
ENSMUSG00000038954	79.29670829	1.068801506	0.413656078	2.583792581	0.009772054	0.040669907
ENSMUSG00000063659	1003.460673	1.069486668	0.23563716	4.538701236	5.66E-06	6.00E-05
ENSMUSG00000024666	75.6708687	1.069905993	0.410428408	2.606802972	0.009139193	0.038646295
ENSMUSG00000028821	1153.001455	1.070222114	0.248130788	4.313137131	1.61E-05	0.000154581
ENSMUSG00000037190	308.1185705	1.070890136	0.317169414	3.376397878	0.000734416	0.00463833
ENSMUSG00000017561	838.5543568	1.071236516	0.237751441	4.505699366	6.62E-06	6.94E-05
ENSMUSG00000018882	981.3008752	1.074071695	0.26018369	4.128128455	3.66E-05	0.00032803
ENSMUSG00000047648	280.1266312	1.074244792	0.336551491	3.191918092	0.001413314	0.008165881
ENSMUSG00000024644	2383.192137	1.074283351	0.24197288	4.439684935	9.01E-06	9.17E-05
ENSMUSG00000047712	75.39267166	1.074466283	0.425871571	2.522981939	0.011636438	0.046877536
ENSMUSG00000026480	1911.4957	1.075215244	0.2384226	4.509703231	6.49E-06	6.83E-05

	47		16			
ENSMUSG00000026490	194.9839084	1.075226784	0.329406567	3.264132815	0.001097997	0.006585578
ENSMUSG00000032745	1329.639965	1.077644333	0.306490734	3.516074758	0.000437978	0.002949623
ENSMUSG00000024277	250.9216278	1.077893838	0.301577906	3.574180389	0.000351327	0.002433013
ENSMUSG00000035840	416.4430662	1.078025104	0.330759023	3.259246241	0.001117087	0.006682528
ENSMUSG00000022876	391.4299088	1.07833597	0.299107171	3.605182602	0.000311934	0.002197969
ENSMUSG00000028557	751.2314311	1.079933384	0.245384855	4.400978137	1.08E-05	0.00010804
ENSMUSG00000052760	351.1237355	1.08002302	0.280789878	3.846374476	0.000119879	0.000951874
ENSMUSG00000055371	896.0570924	1.080785021	0.249432621	4.332973828	1.47E-05	0.000142987
ENSMUSG00000037287	360.0112625	1.081733242	0.269322515	4.016497631	5.91E-05	0.000508118
ENSMUSG00000024472	1269.445569	1.083901825	0.235646462	4.599694878	4.23E-06	4.61E-05
ENSMUSG00000022139	1051.024454	1.084331329	0.275279342	3.939021803	8.18E-05	0.000677913
ENSMUSG00000045414	261.6770861	1.084348012	0.288643836	3.756699001	0.000172169	0.001313798
ENSMUSG00000024066	583.6632766	1.084657012	0.252867537	4.289427683	1.79E-05	0.00017037
ENSMUSG00000021044	578.6860659	1.085977919	0.255079001	4.257417959	2.07E-05	0.000194791
ENSMUSG00000027088	786.3702573	1.086009088	0.255963403	4.242829543	2.21E-05	0.0002062
ENSMUSG00000045896	428.9641153	1.088449887	0.260586394	4.176925238	2.95E-05	0.000269607
ENSMUSG00000033326	1068.599949	1.090052768	0.258186455	4.221959544	2.42E-05	0.000224398
ENSMUSG00000014444	556.1100001	1.091325003	0.271718337	4.016383337	5.91E-05	0.000508118
ENSMUSG00000048621	224.039049	1.094343553	0.347501124	3.149179891	0.001637294	0.009222816
ENSMUSG00000003226	2372.446992	1.095938792	0.265384832	4.129621058	3.63E-05	0.000326121
ENSMUSG00000049323	879.0649369	1.097190085	0.239635364	4.578581671	4.68E-06	5.06E-05
ENSMUSG00000041143	192.7770937	1.097847297	0.332566883	3.30113235	0.000962954	0.005852457
ENSMUSG00000031393	300.2418911	1.098398514	0.284324313	3.863188847	0.000111916	0.000896443
ENSMUSG00000026641	477.3336269	1.098607897	0.266850927	4.116934916	3.84E-05	0.000342353
ENSMUSG00000038888	75.28428264	1.099029301	0.430353073	2.553785183	0.010655898	0.043593754
ENSMUSG00000027012	1878.751079	1.099538743	0.285284429	3.854184208	0.000116116	0.000925216
ENSMUSG00000036402	174.12015	1.10000968	0.3256786	3.377592277	0.000731	0.00462

	65		46		234	0361
ENSMUSG00000004221	1113.417322	1.100793949	0.231118446	4.762899576	1.91E-06	2.25E-05
ENSMUSG00000017631	2718.210504	1.101165277	0.218841472	5.031794335	4.86E-07	6.35E-06
ENSMUSG00000028779	470.4874688	1.101256807	0.27402476	4.018822262	5.85E-05	0.000503519
ENSMUSG00000045210	1223.94335	1.101352733	0.28290985	3.892945877	9.90E-05	0.000802166
ENSMUSG00000025647	1754.20466	1.101753926	0.236755818	4.653545298	3.26E-06	3.66E-05
ENSMUSG00000001750	3129.103569	1.10186011	0.233304027	4.722850787	2.33E-06	2.69E-05
ENSMUSG00000034480	135.8046874	1.101977001	0.361074473	3.051938262	0.002273689	0.012082552
ENSMUSG00000032119	182.4172962	1.102366122	0.313614579	3.515034684	0.000439697	0.002956841
ENSMUSG00000003178	80.013186	1.102468039	0.40831169	2.700064845	0.006932596	0.030797806
ENSMUSG00000026991	854.0134963	1.102627522	0.23896072	4.614262645	3.94E-06	4.34E-05
ENSMUSG00000020827	177.3089308	1.102951552	0.32263824	3.418539457	0.000629582	0.004058523
ENSMUSG00000054364	311.1636076	1.105282661	0.317811153	3.477796962	0.000505553	0.003352008
ENSMUSG00000105452	226.7051161	1.105541905	0.333024554	3.319700875	0.000901139	0.005518372
ENSMUSG00000019189	681.9064634	1.105694297	0.25084774	4.407830409	1.04E-05	0.000105003
ENSMUSG00000024052	574.4838964	1.106326668	0.254176368	4.352594531	1.35E-05	0.000132078
ENSMUSG00000016256	33299.92819	1.107268215	0.219755609	5.038634604	4.69E-07	6.14E-06
ENSMUSG00000058818	166.0460116	1.107417775	0.345771718	3.202742498	0.001361256	0.007905093
ENSMUSG00000026112	3037.258339	1.107516243	0.218777122	5.062303739	4.14E-07	5.49E-06
ENSMUSG00000020230	494.2629239	1.107686981	0.291792558	3.796145419	0.000146963	0.001145044
ENSMUSG00000014599	942.6515879	1.108893566	0.240909848	4.602939959	4.17E-06	4.55E-05
ENSMUSG00000025235	80.25218614	1.109907405	0.409604459	2.709705381	0.0067343	0.03017116
ENSMUSG00000026162	145.7071666	1.110371745	0.352191522	3.152749788	0.001617404	0.009128659
ENSMUSG00000024833	232.5032753	1.112457528	0.311761591	3.568295651	0.000359311	0.002483286
ENSMUSG00000025337	650.9110998	1.113147274	0.245484682	4.534487706	5.77E-06	6.11E-05
ENSMUSG00000066036	1588.427262	1.113394965	0.247000289	4.507666655	6.55E-06	6.89E-05
ENSMUSG00000089417	1672.888078	1.113958435	0.300022791	3.712912717	0.000204888	0.001530237
ENSMUSG00000026317	2011.0068	1.114649704	0.2445565	4.557840974	5.17E-06	5.53E-05

	09		15			
ENSMUSG00000053581	999.64445 67	1.11557675	0.2358557 86	4.729910458	2.25E-06	2.61E-05
ENSMUSG00000020423	326.53024 4	1.115676479	0.2768965 19	4.029218149	5.60E-05	0.00048 3892
ENSMUSG00000018425	824.14779 15	1.116001765	0.2622899 17	4.254840513	2.09E-05	0.00019 6778
ENSMUSG00000042650	1502.9491 08	1.117675033	0.2702624 11	4.135517875	3.54E-05	0.00031 8484
ENSMUSG00000030287	168.81851 7	1.118569778	0.3227499 69	3.465747131	0.000528 76	0.00348 2297
ENSMUSG00000020801	155.06023 38	1.118670505	0.3278151 51	3.412503973	0.000643 69	0.00413 0171
ENSMUSG00000102038	96.091228 58	1.118693808	0.3832428	2.919021068	0.003511 325	0.01756 3032
ENSMUSG00000025019	186.64947 9	1.119820122	0.3196455 6	3.503318241	0.000459 5	0.00307 9437
ENSMUSG00000022335	262.94202 16	1.120152031	0.2953744 97	3.79231126	0.000149 252	0.00115 8264
ENSMUSG00000033799	276.39742 16	1.121006692	0.3442844 76	3.256047744	0.001129 748	0.00674 9429
ENSMUSG00000069089	605.01482 54	1.12117586	0.2583771 35	4.33929984	1.43E-05	0.00013 9328
ENSMUSG00000042419	115.92242 38	1.122114631	0.3835182 18	2.925844398	0.003435 227	0.01724 5343
ENSMUSG00000019487	264.04003 75	1.122651219	0.3078926 38	3.646242492	0.000266 103	0.00191 0393
ENSMUSG00000031570	184.17121 83	1.123327205	0.3136467 6	3.581504252	0.000341 622	0.00237 7818
ENSMUSG00000027944	555.78639 89	1.12388676	0.2571951 39	4.369782269	1.24E-05	0.00012 3157
ENSMUSG00000032018	1082.9153 16	1.1252164	0.2676838 36	4.203527629	2.63E-05	0.00024 168
ENSMUSG00000021733	403.34798 18	1.126233689	0.2857095 03	3.941883891	8.08E-05	0.00067 0683
ENSMUSG00000022255	3305.2412 42	1.127507737	0.2236823 24	5.040665343	4.64E-07	6.09E-06
ENSMUSG00000003031	381.83798 02	1.128261948	0.2698726 06	4.180720548	2.91E-05	0.00026 5499
ENSMUSG00000025226	84.659846 68	1.128338331	0.4492063 24	2.51184872	0.012010 056	0.04798 7702
ENSMUSG00000025484	264.48773 76	1.128943398	0.3027800 35	3.728592601	0.000192 552	0.00145 1555
ENSMUSG00000022529	393.15388 08	1.129181601	0.2706010 87	4.172864252	3.01E-05	0.00027 3731
ENSMUSG00000033159	1216.0820 93	1.129404646	0.2589702 59	4.361136482	1.29E-05	0.00012 7665
ENSMUSG00000052253	702.47630 42	1.129944629	0.2454267 32	4.603999816	4.14E-06	4.53E-05
ENSMUSG00000041645	2011.5710 89	1.130050327	0.2418138 92	4.673223361	2.97E-06	3.35E-05
ENSMUSG00000034271	310.94099 01	1.131648243	0.3145540 46	3.597627362	0.000321 133	0.00225 2752
ENSMUSG00000020246	128.92580	1.131962912	0.3650837	3.100557016	0.001931	0.01055

	09		27		57	9302
ENSMUSG00000002797	110.7658879	1.1330982	0.369571976	3.06597435	0.002169619	0.011628718
ENSMUSG00000063897	567.5443037	1.13663878	0.330724415	3.436815452	0.000588597	0.00381766
ENSMUSG00000032939	631.4343513	1.138084989	0.248857718	4.573235659	4.80E-06	5.17E-05
ENSMUSG00000038497	480.0873365	1.139068278	0.258726254	4.40260028	1.07E-05	0.000107392
ENSMUSG00000089536	701.5006774	1.139674775	0.249142447	4.574390224	4.78E-06	5.15E-05
ENSMUSG00000051306	79.14148802	1.143095542	0.421830544	2.709845359	0.006731458	0.030168292
ENSMUSG00000104560	216.7924673	1.143654991	0.320711773	3.565990051	0.000362485	0.00250018
ENSMUSG00000049421	305.1128072	1.143728389	0.36045171	3.173041929	0.001508507	0.008603451
ENSMUSG00000033739	173.7654986	1.146322931	0.327001541	3.505558186	0.000455651	0.00305663
ENSMUSG00000058046	122.339127	1.146506444	0.405716049	2.825883879	0.004715035	0.022413999
ENSMUSG00000025981	255.0380907	1.14777585	0.312476515	3.67315876	0.000239571	0.001747373
ENSMUSG00000031799	3732.12178	1.148794104	0.2319226	4.953351256	7.29E-07	9.16E-06
ENSMUSG00000030019	480.9883578	1.149909673	0.309742922	3.712464729	0.000205251	0.001532114
ENSMUSG00000056692	1324.893261	1.150692238	0.236909128	4.857103842	1.19E-06	1.45E-05
ENSMUSG00000000708	500.3285763	1.151575795	0.265758692	4.333163235	1.47E-05	0.000142966
ENSMUSG00000025245	83.88730123	1.151848176	0.423199987	2.721758536	0.006493557	0.029235939
ENSMUSG00000048752	200.7226828	1.152634054	0.315137121	3.657563569	0.000254624	0.001839549
ENSMUSG00000039483	272.9175343	1.1534181	0.304710113	3.785296424	0.000153525	0.00118941
ENSMUSG00000029173	280.657277	1.153454773	0.292106962	3.948741127	7.86E-05	0.000653343
ENSMUSG00000066406	1322.27419	1.15404298	0.237056424	4.868220653	1.13E-06	1.37E-05
ENSMUSG00000022009	304.9418305	1.156176297	0.29874979	3.870048907	0.000108814	0.000873188
ENSMUSG00000079179	216.8700925	1.159264398	0.307583579	3.768941119	0.000163942	0.001259428
ENSMUSG00000024677	321.3000164	1.160079941	0.300849605	3.856012837	0.000115251	0.00091886
ENSMUSG00000039844	1046.70493	1.161106813	0.241568733	4.806527735	1.54E-06	1.83E-05
ENSMUSG00000017677	2220.984881	1.161228291	0.226277765	5.131870968	2.87E-07	3.98E-06
ENSMUSG00000051457	91.91340412	1.161341029	0.39129514	2.96794136	0.002998015	0.015342718
ENSMUSG00000024327	321.53923	1.162148968	0.2849506	4.07842249	4.53E-05	0.00039

	97		08			9368
ENSMUSG00000025779	167.9282375	1.162652755	0.361589758	3.215391832	0.001302666	0.007613003
ENSMUSG00000026197	203.6418305	1.163089151	0.324629678	3.5828183	0.000339907	0.002367086
ENSMUSG00000037958	173.8507461	1.163188971	0.358550921	3.244138847	0.001178063	0.006989331
ENSMUSG00000021036	3151.200522	1.16373393	0.234879721	4.954595161	7.25E-07	9.10E-06
ENSMUSG00000033880	561.1318513	1.16398719	0.258640399	4.500407488	6.78E-06	7.10E-05
ENSMUSG00000033416	230.6932072	1.164471372	0.302492568	3.849586721	0.000118317	0.000941873
ENSMUSG00000091549	293.2201327	1.16696006	0.286835495	4.068394885	4.73E-05	0.000415611
ENSMUSG00000057219	78.00267752	1.167786275	0.427122958	2.734075174	0.006255576	0.028341379
ENSMUSG00000036249	365.1535794	1.167940529	0.293753908	3.975914858	7.01E-05	0.000592019
ENSMUSG00000035778	809.5515968	1.168235456	0.248396421	4.703109049	2.56E-06	2.93E-05
ENSMUSG00000025503	4038.654201	1.168249925	0.222559095	5.249167301	1.53E-07	2.22E-06
ENSMUSG00000037826	192.6019134	1.169214859	0.315407731	3.706994928	0.000209733	0.001560474
ENSMUSG00000079316	696.9047048	1.169224195	0.269600855	4.336871243	1.45E-05	0.000140676
ENSMUSG00000010110	791.6812748	1.169385232	0.241241632	4.847360798	1.25E-06	1.51E-05
ENSMUSG00000062743	77.85673429	1.169922073	0.465221997	2.514760871	0.011911314	0.047698199
ENSMUSG00000099597	70.41371389	1.171636253	0.42040585	2.7869171	0.005321209	0.024822045
ENSMUSG00000035529	258.9928237	1.172839615	0.293348063	3.998116109	6.38E-05	0.000543507
ENSMUSG00000046519	364.3837518	1.174818416	0.28747006	4.086750516	4.37E-05	0.000386797
ENSMUSG00000041193	606.5551252	1.174872518	0.261065739	4.500293761	6.79E-06	7.10E-05
ENSMUSG00000029553	587.1602058	1.174960681	0.271447416	4.328501991	1.50E-05	0.000145715
ENSMUSG00000031608	2224.817763	1.174990709	0.245113682	4.793656141	1.64E-06	1.94E-05
ENSMUSG00000046034	1156.163479	1.175755652	0.232530918	5.056341159	4.27E-07	5.66E-06
ENSMUSG00000034269	448.1560656	1.178340112	0.281845783	4.180797385	2.90E-05	0.000265499
ENSMUSG00000062116	231.7850491	1.179098502	0.30099348	3.917355629	8.95E-05	0.0007347
ENSMUSG00000032905	818.4185886	1.18221932	0.247228841	4.781882704	1.74E-06	2.05E-05
ENSMUSG00000024943	1468.235477	1.182740354	0.307852096	3.841910999	0.00012208	0.000967114
ENSMUSG00000081896	450.97433	1.182753129	0.3596794	3.288353295	0.001007	0.00610

	28		58		753	0377
ENSMUSG00000067942	110.9787401	1.18366796	0.414226642	2.85753701	0.004269428	0.020634032
ENSMUSG00000055561	159.9032806	1.183715672	0.334259763	3.54130471	0.000398153	0.002722901
ENSMUSG00000021684	344.0226877	1.184512404	0.282334085	4.195428284	2.72E-05	0.00025001
ENSMUSG00000025017	1646.147684	1.184552692	0.232000395	5.105821877	3.29E-07	4.49E-06
ENSMUSG00000022125	2005.848307	1.184731428	0.224108921	5.286409059	1.25E-07	1.84E-06
ENSMUSG00000055200	63.84174825	1.185230349	0.461302169	2.569314496	0.010189993	0.042140572
ENSMUSG00000038623	373.0848024	1.186650945	0.282106482	4.206393763	2.59E-05	0.000239149
ENSMUSG00000074220	113.2094725	1.187533163	0.361361973	3.28627042	0.001015235	0.006132126
ENSMUSG00000022092	78.12523132	1.189438827	0.413906741	2.87368798	0.004057096	0.019773291
ENSMUSG00000030523	198.5224876	1.190256264	0.30827001	3.861083545	0.000112885	0.000902621
ENSMUSG00000063019	134.5574923	1.191985381	0.34774561	3.42775105	0.000608603	0.003941828
ENSMUSG00000032382	2178.432811	1.193917151	0.25317359	4.715804485	2.41E-06	2.78E-05
ENSMUSG00000019082	79.96320356	1.194802658	0.407364512	2.933006248	0.003356971	0.01694324
ENSMUSG00000025076	314.7289959	1.196466084	0.278049069	4.303075317	1.68E-05	0.000161209
ENSMUSG00000040943	667.5563311	1.196772807	0.26013435	4.600595062	4.21E-06	4.60E-05
ENSMUSG00000027712	8259.261003	1.197118013	0.228973087	5.228204015	1.71E-07	2.45E-06
ENSMUSG00000106590	123.9691466	1.197789589	0.393245019	3.045911663	0.00231976	0.012294009
ENSMUSG00000085823	139.6942314	1.199729874	0.362561276	3.309040299	0.000936164	0.00570735
ENSMUSG00000036890	252.160355	1.199939767	0.30605031	3.920727173	8.83E-05	0.000726671
ENSMUSG00000081227	76.8602189	1.200022313	0.430787377	2.785648739	0.005342073	0.024910889
ENSMUSG00000084289	2513.488836	1.201798424	0.290304484	4.139785953	3.48E-05	0.000313439
ENSMUSG00000029223	483.3261549	1.202968092	0.260321079	4.621093683	3.82E-06	4.22E-05
ENSMUSG00000016528	6121.998437	1.203050007	0.215448307	5.583938095	2.35E-08	3.86E-07
ENSMUSG00000044786	122.3755893	1.203662864	0.356085206	3.380266421	0.000724156	0.00457986
ENSMUSG00000032724	151.901725	1.203723047	0.389262099	3.092320185	0.001985985	0.010800764
ENSMUSG00000026788	374.7761249	1.205107942	0.268397342	4.490014443	7.12E-06	7.41E-05
ENSMUSG00000038608	2987.7658	1.208230961	0.2625408	4.602068097	4.18E-06	4.57E-05

	79		7			
ENSMUSG00000020397	58.93709842	1.208410678	0.466626491	2.589674398	0.009606675	0.040189096
ENSMUSG00000031207	1700.712463	1.208776189	0.228931011	5.280089322	1.29E-07	1.89E-06
ENSMUSG00000024754	212.3346928	1.209654085	0.305738699	3.956496478	7.61E-05	0.000635586
ENSMUSG00000025525	277.4373755	1.210969609	0.339533749	3.566566252	0.000361689	0.002496297
ENSMUSG00000005483	495.2790436	1.211129865	0.268889751	4.504187542	6.66E-06	6.99E-05
ENSMUSG00000083764	338.5401437	1.211189928	0.27516312	4.401716071	1.07E-05	0.000107752
ENSMUSG00000030653	86.59261532	1.213765036	0.406539773	2.98559973	0.002830228	0.014609521
ENSMUSG00000039158	107.99225	1.214134551	0.403812818	3.0066766	0.002641206	0.01378961
ENSMUSG00000021814	2525.094531	1.214170115	0.258553377	4.696013371	2.65E-06	3.02E-05
ENSMUSG00000029073	343.2947511	1.214805475	0.280994902	4.323229589	1.54E-05	0.000148821
ENSMUSG00000036636	2459.415119	1.215028706	0.222753937	5.454577919	4.91E-08	7.65E-07
ENSMUSG00000027878	1100.18462	1.217156621	0.258713044	4.704658892	2.54E-06	2.92E-05
ENSMUSG00000060904	980.1737845	1.217618287	0.256276147	4.751196314	2.02E-06	2.37E-05
ENSMUSG00000022842	836.0795101	1.218410763	0.242380049	5.026860776	4.99E-07	6.50E-06
ENSMUSG00000071640	73.62106358	1.218871646	0.434823262	2.803142688	0.005060727	0.023768769
ENSMUSG00000022148	338.5762468	1.219319414	0.321431104	3.793408295	0.000148594	0.001154477
ENSMUSG00000052920	222.8382516	1.220873447	0.304675927	4.007121462	6.15E-05	0.000525486
ENSMUSG00000067787	421.410145	1.221033169	0.265452551	4.599817041	4.23E-06	4.61E-05
ENSMUSG00000026499	1447.550388	1.221149545	0.239473993	5.099299226	3.41E-07	4.63E-06
ENSMUSG00000035517	844.4906329	1.222024103	0.241135052	5.067799538	4.02E-07	5.35E-06
ENSMUSG00000030109	1262.220381	1.222815427	0.251138291	4.869091932	1.12E-06	1.37E-05
ENSMUSG00000033863	375.1794865	1.222952741	0.279460964	4.376112933	1.21E-05	0.000120419
ENSMUSG00000037720	942.9000464	1.223102257	0.276213758	4.428100412	9.51E-06	9.64E-05
ENSMUSG00000064120	212.5658638	1.225887198	0.31192778	3.930035344	8.49E-05	0.000701212
ENSMUSG00000061132	584.9358982	1.226935576	0.280033017	4.381396123	1.18E-05	0.000117707
ENSMUSG00000021809	187.5319187	1.22790166	0.322321119	3.809560059	0.000139214	0.001088381
ENSMUSG00000083937	442.47305	1.23140437	0.3151466	3.9074017	9.33E-05	0.00076

	22		02			0615
ENSMUSG00000039853	45.14477776	1.233058134	0.490339291	2.514703915	0.011913239	0.047698199
ENSMUSG00000030718	355.5468171	1.23448918	0.275951925	4.473566118	7.69E-06	7.96E-05
ENSMUSG00000065378	122.6954896	1.236119008	0.458588554	2.69548596	0.007028604	0.031129418
ENSMUSG00000004500	157.5411413	1.237166428	0.335616852	3.686246449	0.000227586	0.001668842
ENSMUSG00000027739	286.8764797	1.23735365	0.307144271	4.028574733	5.61E-05	0.000484912
ENSMUSG00000033813	299.16592	1.238985172	0.306507544	4.042266478	5.29E-05	0.000460915
ENSMUSG00000001642	211.723264	1.239201738	0.312448093	3.966104339	7.31E-05	0.000613919
ENSMUSG00000033467	419.1639746	1.239250302	0.336097523	3.687174753	0.000226758	0.001665441
ENSMUSG00000041278	999.7348945	1.239636222	0.266233043	4.656207239	3.22E-06	3.62E-05
ENSMUSG00000007458	2404.890773	1.239908798	0.226811349	5.466696456	4.59E-08	7.19E-07
ENSMUSG00000021242	8764.205671	1.242314093	0.21699199	5.72516107	1.03E-08	1.79E-07
ENSMUSG00000071715	2828.02721	1.242668988	0.235083843	5.28606718	1.25E-07	1.84E-06
ENSMUSG00000001785	1130.162246	1.242866179	0.250367344	4.964170484	6.90E-07	8.70E-06
ENSMUSG00000028439	98.95862257	1.243307566	0.381109353	3.262338108	0.001104973	0.006624519
ENSMUSG00000048163	218.5642884	1.24707719	0.303121366	4.1141184	3.89E-05	0.000345884
ENSMUSG00000031701	2018.825418	1.247714957	0.285530154	4.369818532	1.24E-05	0.000123157
ENSMUSG00000047153	278.0254011	1.248225543	0.305855704	4.081092902	4.48E-05	0.000395315
ENSMUSG00000042772	458.4544564	1.248428924	0.285957593	4.365783435	1.27E-05	0.000125161
ENSMUSG00000056121	573.3494379	1.251741102	0.257648353	4.858331468	1.18E-06	1.44E-05
ENSMUSG00000062070	797.236318	1.253965391	0.248565319	5.044812355	4.54E-07	5.98E-06
ENSMUSG00000052681	3213.934384	1.254211675	0.267703597	4.685075912	2.80E-06	3.17E-05
ENSMUSG00000025521	413.9279518	1.25495111	0.265326554	4.72983609	2.25E-06	2.61E-05
ENSMUSG00000022822	1125.001179	1.257040276	0.25020077	5.024126325	5.06E-07	6.58E-06
ENSMUSG00000031154	230.6543756	1.257235072	0.298511232	4.211684305	2.53E-05	0.00023393
ENSMUSG00000024560	175.3644418	1.258391623	0.331731376	3.793405486	0.000148595	0.001154477
ENSMUSG00000102269	63.31911693	1.258449509	0.439138296	2.865724808	0.004160557	0.020184227
ENSMUSG00000038612	6795.1667	1.259298476	0.2137660	5.891011624	3.84E-09	7.19E-08

	48		82			
ENSMUSG00000050064	71.08837501	1.259380359	0.430869644	2.922880221	0.003468099	0.017374822
ENSMUSG00000080832	529.5020686	1.260139912	0.259962668	4.847387987	1.25E-06	1.51E-05
ENSMUSG00000044477	254.8811369	1.26032977	0.300084001	4.199923248	2.67E-05	0.000245262
ENSMUSG00000014867	3772.366696	1.261288923	0.219974099	5.733806513	9.82E-09	1.71E-07
ENSMUSG00000020900	78.67004699	1.261488941	0.406250088	3.105202873	0.001901486	0.010436468
ENSMUSG00000019794	478.7870426	1.263125675	0.2984667	4.232048922	2.32E-05	0.000215166
ENSMUSG00000078813	80.41741428	1.263284754	0.426722048	2.960439377	0.003072006	0.015645425
ENSMUSG00000026177	3756.098719	1.26331618	0.228849924	5.52028227	3.38E-08	5.39E-07
ENSMUSG00000100018	247.8941137	1.264790218	0.321739456	3.931100761	8.46E-05	0.000698532
ENSMUSG00000051650	84.65338257	1.267537751	0.437255447	2.898849538	0.003745346	0.018483963
ENSMUSG00000075701	1473.608244	1.267562804	0.244581303	5.182582601	2.19E-07	3.10E-06
ENSMUSG00000024661	38239.10065	1.267617242	0.220246114	5.755457937	8.64E-09	1.53E-07
ENSMUSG00000024927	325.3047863	1.268208125	0.285073991	4.448698105	8.64E-06	8.84E-05
ENSMUSG00000029470	1288.84116	1.270926044	0.24075938	5.278822543	1.30E-07	1.90E-06
ENSMUSG00000018707	4839.370205	1.271857125	0.223517345	5.690194304	1.27E-08	2.17E-07
ENSMUSG00000078945	760.5953486	1.272136002	0.247918296	5.131271152	2.88E-07	3.99E-06
ENSMUSG00000026548	187.4108673	1.272344257	0.31684427	4.015677032	5.93E-05	0.000509322
ENSMUSG00000100554	66.75566256	1.272866266	0.453787556	2.804982749	0.005031927	0.023657826
ENSMUSG00000060034	139.6968178	1.273230275	0.348226506	3.656327859	0.000255854	0.001845516
ENSMUSG00000054293	581.0861612	1.274813261	0.330518133	3.857014586	0.00011478	0.000915637
ENSMUSG00000041037	194.6715395	1.275304135	0.358262926	3.559687713	0.000371296	0.002553243
ENSMUSG00000020733	453.1637834	1.275934467	0.290879503	4.386470877	1.15E-05	0.000115163
ENSMUSG00000037622	326.0694405	1.279551236	0.300627989	4.256261169	2.08E-05	0.000195667
ENSMUSG00000022999	93.18567488	1.280268734	0.399599967	3.203875973	0.001355909	0.007880716
ENSMUSG00000047368	380.2366404	1.282099567	0.317417726	4.039155536	5.36E-05	0.000466196
ENSMUSG00000027782	1326.650024	1.282914594	0.259977281	4.934718107	8.03E-07	1.00E-05
ENSMUSG00000050002	386.29248	1.283280614	0.2763446	4.64376928	3.42E-06	3.82E-05

	56		11			
ENSMUSG00000031628	461.65695 73	1.283285213	0.3025309 35	4.241831378	2.22E-05	0.00020 6978
ENSMUSG00000018171	1156.2626 29	1.284950902	0.2684731 75	4.786142602	1.70E-06	2.01E-05
ENSMUSG00000062310	401.91085 44	1.284964093	0.3127227 35	4.108956426	3.97E-05	0.00035 3247
ENSMUSG00000024981	2803.8600 88	1.285741216	0.2426394 19	5.298979112	1.16E-07	1.72E-06
ENSMUSG00000064181	116.82506 84	1.287174252	0.3595860 33	3.57960025	0.000344 12	0.00238 9143
ENSMUSG00000041168	3358.7676 62	1.288393309	0.2598734 68	4.957771647	7.13E-07	8.97E-06
ENSMUSG00000041957	421.40360 98	1.289064305	0.2639690 46	4.883391924	1.04E-06	1.28E-05
ENSMUSG00000065818	278.77681 92	1.289664763	0.3106866 81	4.151014004	3.31E-05	0.00029 9632
ENSMUSG00000029474	1179.9705 85	1.290345963	0.2409208 07	5.355892576	8.51E-08	1.29E-06
ENSMUSG00000022507	686.27802 09	1.290691618	0.2685952 25	4.805340888	1.54E-06	1.84E-05
ENSMUSG00000030275	2026.5942 13	1.292367332	0.2327295 21	5.553087231	2.81E-08	4.55E-07
ENSMUSG00000041096	246.18333 4	1.292503081	0.3307781 59	3.907461979	9.33E-05	0.00076 0615
ENSMUSG00000019970	476.27183 93	1.292816479	0.2754340 87	4.693741777	2.68E-06	3.05E-05
ENSMUSG00000035713	176.97064 8	1.292920735	0.3420392 31	3.780036376	0.000156 805	0.00121 0715
ENSMUSG00000033961	94.845099 55	1.293763514	0.4076308 13	3.173860938	0.001504 257	0.00858 2784
ENSMUSG00000021039	850.62031 83	1.293990825	0.3065961 31	4.220506044	2.44E-05	0.00022 5415
ENSMUSG00000094392	57.090822 95	1.295258895	0.4544946 66	2.849887999	0.004373 463	0.02105 3146
ENSMUSG00000102270	190.44683 57	1.296682171	0.3174953 87	4.084097676	4.42E-05	0.00039 0989
ENSMUSG00000058135	281.79773 36	1.298141836	0.3245778 44	3.999477665	6.35E-05	0.00054 1398
ENSMUSG00000051341	313.89389 61	1.298371997	0.3707708 87	3.501817548	0.000462 096	0.00309 3808
ENSMUSG00000063406	2052.7675 57	1.298383364	0.3072759 96	4.225463034	2.38E-05	0.00022 1256
ENSMUSG00000078908	395.90559 53	1.300051618	0.2806465 82	4.632344379	3.62E-06	4.02E-05
ENSMUSG00000023892	156.52663 64	1.300734238	0.4050394 23	3.211376877	0.001321 006	0.00770 3992
ENSMUSG00000029686	2649.1563 58	1.300964755	0.2769371 36	4.69768978	2.63E-06	3.00E-05
ENSMUSG00000021266	2074.7256 14	1.301231432	0.2245657 59	5.794433826	6.86E-09	1.22E-07
ENSMUSG00000039298	452.15410 72	1.303335759	0.2624068 51	4.966851114	6.80E-07	8.61E-06
ENSMUSG00000028337	381.84624	1.30369803	0.2695988	4.835695616	1.33E-06	1.59E-05

	4		61			
ENSMUSG00000025287	1062.731423	1.304636554	0.249793124	5.222868169	1.76E-07	2.52E-06
ENSMUSG00000071076	1457.548108	1.309692819	0.262131315	4.996323378	5.84E-07	7.52E-06
ENSMUSG00000029313	791.6991733	1.310037968	0.243826815	5.372821559	7.75E-08	1.18E-06
ENSMUSG00000007216	56.60120666	1.310282359	0.506837961	2.585209591	0.009731982	0.04055239
ENSMUSG00000048807	1037.76355	1.310557426	0.288549855	4.541875179	5.58E-06	5.92E-05
ENSMUSG00000020387	305.7258256	1.312177437	0.283486763	4.628707959	3.68E-06	4.07E-05
ENSMUSG00000049516	340.0653016	1.313955183	0.275222516	4.774155847	1.80E-06	2.13E-05
ENSMUSG00000017831	693.9170761	1.314531684	0.289345833	4.543116	5.54E-06	5.90E-05
ENSMUSG00000020409	1088.956023	1.314684679	0.262047734	5.016966409	5.25E-07	6.81E-06
ENSMUSG00000069114	174.3247069	1.314888562	0.330050833	3.983897113	6.78E-05	0.00057495
ENSMUSG00000026796	3571.369119	1.3172804	0.235825233	5.585833143	2.33E-08	3.83E-07
ENSMUSG00000023456	5241.669991	1.318143278	0.22049323	5.978157584	2.26E-09	4.38E-08
ENSMUSG00000027882	501.7215486	1.318434034	0.293574649	4.490966915	7.09E-06	7.38E-05
ENSMUSG00000097039	93.48147349	1.319230811	0.403768276	3.26729684	0.001085798	0.00652383
ENSMUSG00000038615	2507.940815	1.320039087	0.227910887	5.79190887	6.96E-09	1.24E-07
ENSMUSG00000063800	556.5840159	1.321684307	0.303191152	4.359244321	1.31E-05	0.000128497
ENSMUSG00000035954	314.6142015	1.322702618	0.287119741	4.606797894	4.09E-06	4.48E-05
ENSMUSG00000024335	1645.846153	1.323236666	0.225570008	5.866190619	4.46E-09	8.23E-08
ENSMUSG00000020128	790.6675006	1.323455113	0.285186711	4.640661933	3.47E-06	3.87E-05
ENSMUSG00000006373	655.997939	1.325743537	0.263243727	5.036182823	4.75E-07	6.22E-06
ENSMUSG00000015536	991.9958474	1.326594224	0.265730799	4.992248647	5.97E-07	7.67E-06
ENSMUSG00000099061	57.0503849	1.327703952	0.455903121	2.912250194	0.003588351	0.017837631
ENSMUSG00000060798	143.35332	1.328554467	0.339409104	3.914315938	9.07E-05	0.000743126
ENSMUSG00000060131	1865.498248	1.329508022	0.226549614	5.868507116	4.40E-09	8.12E-08
ENSMUSG00000019923	1164.570374	1.330829993	0.260304244	5.11259429	3.18E-07	4.36E-06
ENSMUSG00000068749	1159.786209	1.331049486	0.2349641	5.664905759	1.47E-08	2.49E-07
ENSMUSG00000050490	620.29714	1.332614465	0.2503403	5.323211843	1.02E-07	1.52E-06

	31		03			
ENSMUSG00000030291	317.68487 03	1.332847684	0.3246625 93	4.105331851	4.04E-05	0.00035 8599
ENSMUSG00000014850	238.50746 8	1.334151805	0.3257673 97	4.095412304	4.21E-05	0.00037 2855
ENSMUSG00000004446	1643.0969 66	1.337272021	0.2353658 79	5.68167326	1.33E-08	2.27E-07
ENSMUSG00000090946	1016.7419 22	1.338072636	0.2335341 22	5.729666496	1.01E-08	1.75E-07
ENSMUSG00000034575	258.50214 61	1.338479497	0.3043149 37	4.398336503	1.09E-05	0.00010 9283
ENSMUSG00000018986	297.72936 79	1.339940725	0.2918090 54	4.591840822	4.39E-06	4.77E-05
ENSMUSG00000053063	192.50000 52	1.340201002	0.3566461 12	3.757789469	0.000171 421	0.00130 8816
ENSMUSG00000022216	1062.3425 72	1.340520878	0.2780614 45	4.820951988	1.43E-06	1.71E-05
ENSMUSG00000031983	98.408614 17	1.340724497	0.3893009 63	3.443928031	0.000573 328	0.00373 4536
ENSMUSG00000097048	70.993108 97	1.34098653	0.4231096 7	3.169359209	0.001527 755	0.00869 1255
ENSMUSG00000106755	334.61534 52	1.341403156	0.2957549 41	4.535522388	5.75E-06	6.08E-05
ENSMUSG00000033769	420.04517 49	1.34175844	0.2632282 03	5.097320213	3.44E-07	4.67E-06
ENSMUSG00000056429	1954.4654 65	1.343071651	0.2350724 82	5.713436305	1.11E-08	1.91E-07
ENSMUSG00000027613	1901.9231 35	1.343742171	0.2540131 58	5.290049472	1.22E-07	1.80E-06
ENSMUSG00000034361	69.640035 92	1.347172309	0.4511360 38	2.98617755	0.002824 886	0.01459 8439
ENSMUSG00000069792	336.03910 23	1.347822285	0.2903867 98	4.641472314	3.46E-06	3.86E-05
ENSMUSG00000039086	81.334924 43	1.349615753	0.4057293 22	3.32639442	0.000879 773	0.00541 1712
ENSMUSG00000000275	1656.6342 68	1.352368929	0.2250397 91	6.009465799	1.86E-09	3.64E-08
ENSMUSG00000038301	2612.9305 83	1.354060586	0.2451338 42	5.523760299	3.32E-08	5.31E-07
ENSMUSG00000027552	67.631001 13	1.35670154	0.4381029 34	3.09676433	0.001956 453	0.01066 131
ENSMUSG00000024079	1776.2813 02	1.357268114	0.2904886 74	4.672361566	2.98E-06	3.36E-05
ENSMUSG00000066150	887.32254 12	1.360732942	0.2463492 9	5.523591901	3.32E-08	5.31E-07
ENSMUSG00000026672	87.592739 54	1.361553824	0.3952164 83	3.4450836	0.000570 883	0.00372 0375
ENSMUSG00000032392	46.036315 14	1.363039476	0.5033229 71	2.7080812	0.006767 346	0.03026 9739
ENSMUSG00000057554	2738.1197 4	1.365411575	0.2525626 9	5.40622836	6.44E-08	9.88E-07
ENSMUSG00000025314	1171.6709 73	1.365456955	0.2406292 96	5.674525004	1.39E-08	2.36E-07
ENSMUSG00000022035	664.57281	1.366339954	0.2752334	4.964294694	6.90E-07	8.70E-06

	7		5			
ENSMUSG00000085586	72.875067 65	1.366392002	0.4191215 65	3.2601329	0.001113 6	0.00667 0407
ENSMUSG00000046027	470.55998 71	1.367773018	0.2691308 04	5.082186793	3.73E-07	5.00E-06
ENSMUSG00000003955	514.17307 82	1.369704935	0.2798848 68	4.893815597	9.89E-07	1.22E-05
ENSMUSG00000039770	1424.0798 04	1.372973722	0.2546454 75	5.391706744	6.98E-08	1.07E-06
ENSMUSG00000101828	59.711313 84	1.373949435	0.4890980 42	2.809149326	0.004967 26	0.02340 1959
ENSMUSG00000020585	1664.4550 24	1.374415021	0.2704598 98	5.081770084	3.74E-07	5.00E-06
ENSMUSG00000059326	468.56022 05	1.374600428	0.2816180 31	4.881081022	1.06E-06	1.29E-05
ENSMUSG00000032727	120.70089 14	1.375180674	0.3725595 07	3.69117053	0.000223 224	0.00164 3013
ENSMUSG00000029108	399.58380 76	1.375449676	0.2810213 13	4.894467482	9.86E-07	1.22E-05
ENSMUSG00000099397	649.62488 03	1.377281015	0.2755851 5	4.997660489	5.80E-07	7.47E-06
ENSMUSG00000027244	653.68673 08	1.378684106	0.2506167 09	5.501165942	3.77E-08	5.96E-07
ENSMUSG00000024750	2467.3464 37	1.379021937	0.2396223 55	5.754980325	8.67E-09	1.53E-07
ENSMUSG00000030522	423.19896 79	1.37940277	0.2777086 93	4.967085321	6.80E-07	8.61E-06
ENSMUSG00000026107	709.14527 35	1.381407134	0.2529306 27	5.461604835	4.72E-08	7.37E-07
ENSMUSG00000021891	737.62122 72	1.382636984	0.2451517 77	5.639922339	1.70E-08	2.85E-07
ENSMUSG00000004730	8543.4243 35	1.383078132	0.2190184 9	6.314892095	2.70E-10	5.97E-09
ENSMUSG00000029249	936.22291 63	1.384056029	0.2362741 08	5.85784045	4.69E-09	8.57E-08
ENSMUSG00000018167	1209.7630 91	1.385180398	0.2607178 08	5.312948919	1.08E-07	1.60E-06
ENSMUSG00000047407	705.09004 38	1.385660785	0.2510505 23	5.519449906	3.40E-08	5.41E-07
ENSMUSG00000026455	167.20520 92	1.385824789	0.3393347 14	4.083946415	4.43E-05	0.00039 0992
ENSMUSG00000037965	1093.4764 2	1.386322948	0.2836090 74	4.888147374	1.02E-06	1.25E-05
ENSMUSG00000028542	110.29781 72	1.388908184	0.3676224 22	3.778083435	0.000158 04	0.00121 8187
ENSMUSG00000035969	307.51696 81	1.389798965	0.2967508 7	4.683386326	2.82E-06	3.19E-05
ENSMUSG00000097327	71.997293 39	1.391372005	0.5381809 54	2.585323754	0.009728 76	0.04055 1293
ENSMUSG00000028630	192.05334 53	1.392283937	0.3226539 53	4.315099578	1.60E-05	0.00015 343
ENSMUSG00000054720	81.656371 05	1.39244538	0.4041441 26	3.445417838	0.000570 177	0.00371 9314
ENSMUSG00000045975	75.053242	1.395418087	0.4443593	3.140291837	0.001687	0.00945

	99		65		796	4011
ENSMUSG00000017830	544.96324 94	1.396960626	0.2645998 64	5.279521331	1.30E-07	1.90E-06
ENSMUSG00000084520	44.225655 03	1.398479202	0.5499121 45	2.543095685	0.010987 514	0.04471 073
ENSMUSG00000043279	600.44012 26	1.398699751	0.2655411 36	5.267356212	1.38E-07	2.02E-06
ENSMUSG00000042901	442.72185 04	1.39985498	0.2818798 83	4.966140055	6.83E-07	8.63E-06
ENSMUSG00000042719	1249.1564 44	1.400801954	0.2364957 59	5.923158864	3.16E-09	5.98E-08
ENSMUSG00000035828	327.71985 67	1.403093464	0.3279354 57	4.278565897	1.88E-05	0.00017 828
ENSMUSG00000049643	117.15562	1.404126874	0.3615550 74	3.883576732	0.000102 931	0.00083 0294
ENSMUSG00000054976	64.527142 27	1.406510331	0.5072754 18	2.77267591	0.005559 746	0.02575 9405
ENSMUSG00000045969	189.94920 76	1.407942689	0.3244492 99	4.339484457	1.43E-05	0.00013 931
ENSMUSG00000003865	176.89760 4	1.408382236	0.3404876 94	4.136367519	3.53E-05	0.00031 7617
ENSMUSG00000034748	49.547378 5	1.408804928	0.4915810 03	2.865865278	0.004158 712	0.02018 2417
ENSMUSG00000042608	72.883437 31	1.409414286	0.4383490 97	3.215278182	0.001303 182	0.00761 3003
ENSMUSG00000020115	1111.1165 7	1.410789006	0.2493406 66	5.658078277	1.53E-08	2.58E-07
ENSMUSG00000010406	137.60570 04	1.4114617	0.3631231 69	3.887005346	0.000101 489	0.00081 914
ENSMUSG00000026994	325.26601 35	1.411794142	0.2936136 43	4.808339719	1.52E-06	1.82E-05
ENSMUSG00000037638	116.37825 79	1.412163261	0.3598442 44	3.924373623	8.70E-05	0.00071 6613
ENSMUSG00000031647	545.49892 23	1.412280286	0.2618243 58	5.393998858	6.89E-08	1.05E-06
ENSMUSG00000104349	37.295011 92	1.412605928	0.5393665 15	2.619009315	0.008818 554	0.03758 0311
ENSMUSG00000033487	999.38953 08	1.414927715	0.2633979 97	5.371824134	7.79E-08	1.18E-06
ENSMUSG00000079057	1800.9027 16	1.415126182	0.2511852 85	5.633794118	1.76E-08	2.94E-07
ENSMUSG00000027519	1262.5057 02	1.416792623	0.2374192 79	5.967470843	2.41E-09	4.66E-08
ENSMUSG00000032193	903.63705 29	1.41694615	0.2382571 04	5.947130753	2.73E-09	5.19E-08
ENSMUSG00000033629	1323.1509 27	1.418301327	0.2418300 63	5.864867712	4.49E-09	8.27E-08
ENSMUSG00000044060	103.10463 83	1.419585212	0.3951667 32	3.592370247	0.000327 684	0.00229 1279
ENSMUSG00000052566	203.72862 69	1.421210782	0.3534740 85	4.020693011	5.80E-05	0.00050 0165
ENSMUSG00000056458	42.280997 05	1.422671229	0.5349180 82	2.659605793	0.007823 216	0.03412 3861
ENSMUSG00000051498	273.99562	1.423171481	0.3252427	4.375721175	1.21E-05	0.00012

	94		25			0548
ENSMUSG00000019796	212.79237 64	1.423336243	0.3216014 98	4.425776163	9.61E-06	9.73E-05
ENSMUSG00000086176	52.900810 19	1.423364313	0.4769705 46	2.984176539	0.002843 427	0.01467 2124
ENSMUSG00000022831	2981.6840 03	1.423499172	0.2316278 99	6.145629162	7.96E-10	1.64E-08
ENSMUSG00000024856	515.51160 42	1.423897653	0.2747880 78	5.181802877	2.20E-07	3.10E-06
ENSMUSG00000057101	409.37252 55	1.42552177	0.2719508 63	5.241835794	1.59E-07	2.30E-06
ENSMUSG00000004936	2235.3673 52	1.426282285	0.2239540 07	6.368639287	1.91E-10	4.31E-09
ENSMUSG00000095115	1780.3338 71	1.42881578	0.2310634 19	6.183652038	6.26E-10	1.32E-08
ENSMUSG00000081254	81.131532 13	1.429063188	0.4205227 34	3.398301859	0.000678 056	0.00431 4184
ENSMUSG00000001416	6559.0691 73	1.429559315	0.2224537 18	6.426322419	1.31E-10	3.04E-09
ENSMUSG00000029135	208.22079 97	1.429988804	0.3278143 81	4.362190579	1.29E-05	0.00012 7143
ENSMUSG00000024610	1810.2660 61	1.431378209	0.2544345 25	5.625723202	1.85E-08	3.07E-07
ENSMUSG00000024201	156.18120 1	1.434372574	0.3616590 19	3.966090984	7.31E-05	0.00061 3919
ENSMUSG00000002227	381.09497 69	1.435072912	0.2729493 6	5.257652593	1.46E-07	2.12E-06
ENSMUSG00000001666	131.14633 96	1.436037676	0.3576754 75	4.01491793	5.95E-05	0.00051 0217
ENSMUSG00000047565	523.68596 8	1.437250402	0.3041804 36	4.724992905	2.30E-06	2.67E-05
ENSMUSG00000060802	18112.554 36	1.43734559	0.2685490 1	5.352265461	8.69E-08	1.31E-06
ENSMUSG00000037818	70.657195 53	1.437919441	0.4264453 61	3.371872633	0.000746 59	0.00470 3823
ENSMUSG00000052485	66.827833 8	1.439332785	0.4350269 88	3.308605733	0.000937 618	0.00571 1134
ENSMUSG00000034641	832.60944 33	1.441652784	0.2449960 17	5.884392744	4.00E-09	7.45E-08
ENSMUSG00000010154	105.58079 56	1.442977925	0.3740701 9	3.857505791	0.000114 55	0.00091 4864
ENSMUSG00000044330	86.717704 39	1.444492937	0.4003522 73	3.608054795	0.000308 501	0.00217 8169
ENSMUSG00000032497	1085.0902	1.448992801	0.2370415 61	6.112821701	9.79E-10	1.99E-08
ENSMUSG00000024049	70.055842 61	1.449195282	0.4297304 35	3.37233569	0.000745 335	0.00469 863
ENSMUSG00000037012	763.38463 83	1.449474473	0.2429873 48	5.965226104	2.44E-09	4.72E-08
ENSMUSG00000026728	11369.373 4	1.449689234	0.2285282 33	6.343589202	2.24E-10	5.01E-09
ENSMUSG00000036599	150.03591 53	1.451037863	0.3369046 48	4.306968965	1.66E-05	0.00015 851
ENSMUSG00000032231	5154.1271	1.455184429	0.2230497	6.524036262	6.84E-11	1.66E-09

	86		15			
ENSMUSG00000027569	407.0104859	1.455465306	0.272027676	5.350430973	8.77E-08	1.32E-06
ENSMUSG00000034300	180.5413501	1.456717983	0.324479573	4.489398116	7.14E-06	7.42E-05
ENSMUSG00000086782	36.83639045	1.458406642	0.55257213	2.639305462	0.008307609	0.035736277
ENSMUSG00000067869	232.3331232	1.459482678	0.361440928	4.037956315	5.39E-05	0.000467991
ENSMUSG00000037752	111.8745945	1.459483239	0.381096488	3.829694796	0.000128302	0.001011146
ENSMUSG00000071068	623.2716411	1.460837256	0.248413887	5.880658584	4.09E-09	7.61E-08
ENSMUSG00000043257	255.6192553	1.465681242	0.308282294	4.754347791	1.99E-06	2.33E-05
ENSMUSG00000103622	45.01536712	1.467082655	0.499880298	2.934867932	0.003336897	0.01687534
ENSMUSG00000057143	219.3902319	1.467626836	0.347531472	4.22300411	2.41E-05	0.000223534
ENSMUSG00000022564	1021.709869	1.471539583	0.238887237	6.159975724	7.28E-10	1.52E-08
ENSMUSG00000023048	760.3603197	1.472199049	0.261685213	5.625839656	1.85E-08	3.07E-07
ENSMUSG00000031609	2252.261865	1.472584235	0.23118323	6.369771016	1.89E-10	4.29E-09
ENSMUSG00000020458	5014.28327	1.472717915	0.251567347	5.854169608	4.79E-09	8.75E-08
ENSMUSG00000016206	706.1070206	1.475739915	0.244347438	6.039514569	1.55E-09	3.06E-08
ENSMUSG00000038780	352.4092541	1.477079126	0.294205611	5.020567488	5.15E-07	6.69E-06
ENSMUSG00000030878	156.3138778	1.480286655	0.345089889	4.289568325	1.79E-05	0.00017037
ENSMUSG00000036206	35.36628998	1.480297747	0.543499716	2.723640331	0.006456678	0.02912789
ENSMUSG00000033545	849.9701782	1.482071918	0.251439764	5.894341828	3.76E-09	7.05E-08
ENSMUSG00000026566	269.1388417	1.483780308	0.305601162	4.855283593	1.20E-06	1.46E-05
ENSMUSG00000028894	339.1022844	1.48519889	0.278673272	5.329534764	9.85E-08	1.47E-06
ENSMUSG00000099757	50.18976742	1.486412188	0.509209189	2.919060026	0.003510886	0.017563032
ENSMUSG00000037946	1046.970624	1.487488722	0.237492975	6.263295669	3.77E-10	8.11E-09
ENSMUSG00000053470	352.7906946	1.488517246	0.296086354	5.027307824	4.97E-07	6.49E-06
ENSMUSG00000078851	86.10003956	1.490392684	0.415143238	3.590068557	0.000330591	0.002307463
ENSMUSG00000074482	94.95605547	1.490931109	0.448820527	3.32188708	0.000894109	0.005487577
ENSMUSG00000002847	87.70116324	1.492716282	0.405909287	3.67746275	0.000235565	0.001719076
ENSMUSG00000048216	156.82331	1.494249912	0.3357400	4.450615124	8.56E-06	8.78E-05

	52		88			
ENSMUSG00000024736	107.9868246	1.494252637	0.383751511	3.893802616	9.87E-05	0.000801231
ENSMUSG00000000791	30.14759675	1.495806269	0.595706383	2.510979087	0.012039683	0.048078046
ENSMUSG00000097244	31.91658362	1.496647679	0.562817228	2.659207296	0.007832476	0.034153381
ENSMUSG00000022313	291.5114443	1.497591817	0.291041945	5.145621946	2.67E-07	3.74E-06
ENSMUSG00000074580	35.16933002	1.498804666	0.544615973	2.752039495	0.005922538	0.027110347
ENSMUSG00000024235	111.7739408	1.499511897	0.378716579	3.959456702	7.51E-05	0.000629295
ENSMUSG00000056708	2199.402878	1.500529828	0.271538312	5.526033572	3.28E-08	5.25E-07
ENSMUSG00000040774	1035.882081	1.501470495	0.266513952	5.633740694	1.76E-08	2.94E-07
ENSMUSG00000096954	177.5693449	1.501861024	0.325634827	4.612101964	3.99E-06	4.38E-05
ENSMUSG00000020611	3267.225908	1.503031648	0.227680101	6.601506408	4.07E-11	1.03E-09
ENSMUSG00000030084	435.9820621	1.505959141	0.278630976	5.404851824	6.49E-08	9.94E-07
ENSMUSG00000040552	1585.131293	1.506227987	0.279861018	5.382057124	7.36E-08	1.12E-06
ENSMUSG00000021196	4200.445697	1.506988812	0.222484252	6.773462837	1.26E-11	3.34E-10
ENSMUSG00000041736	4139.061699	1.506996829	0.248411237	6.066540486	1.31E-09	2.61E-08
ENSMUSG00000037242	9561.214154	1.50904219	0.213068794	7.082417658	1.42E-12	4.14E-11
ENSMUSG00000027187	2074.212627	1.509366265	0.279086758	5.408233187	6.36E-08	9.79E-07
ENSMUSG00000000628	1369.787365	1.511259748	0.253524109	5.961009986	2.51E-09	4.83E-08
ENSMUSG00000045763	2213.765786	1.512039887	0.236455548	6.394605234	1.61E-10	3.68E-09
ENSMUSG00000029640	973.1265585	1.51265142	0.237392646	6.371938832	1.87E-10	4.24E-09
ENSMUSG00000027951	814.5367526	1.512914888	0.247367344	6.116065541	9.59E-10	1.96E-08
ENSMUSG00000024835	2114.237134	1.513389528	0.248235832	6.096579675	1.08E-09	2.19E-08
ENSMUSG00000029771	1259.325271	1.513619495	0.236761107	6.393024242	1.63E-10	3.71E-09
ENSMUSG00000063889	324.6447823	1.515632355	0.342888734	4.420187084	9.86E-06	9.95E-05
ENSMUSG00000069913	48.3458007	1.516848473	0.509076739	2.979606723	0.002886187	0.014859199
ENSMUSG00000021725	346.6808234	1.518025512	0.318262715	4.769724632	1.84E-06	2.18E-05
ENSMUSG00000046997	178.7947483	1.519946483	0.321656483	4.72537183	2.30E-06	2.66E-05
ENSMUSG00000006418	1718.2556	1.520398793	0.2362580	6.435331897	1.23E-10	2.87E-09

	83		23			
ENSMUSG00000019854	146.90969 41	1.521984524	0.3435850 41	4.429717076	9.44E-06	9.59E-05
ENSMUSG00000026088	581.80010 5	1.525972446	0.3013226 94	5.064246666	4.10E-07	5.45E-06
ENSMUSG00000095440	35.762923 19	1.526749778	0.5942234 12	2.569319463	0.010189 847	0.04214 0572
ENSMUSG00000018796	1164.2886 21	1.528879239	0.2452451 47	6.234085597	4.54E-10	9.72E-09
ENSMUSG00000002699	469.95557 97	1.529952325	0.2828466 04	5.40912389	6.33E-08	9.75E-07
ENSMUSG00000074577	32.348495 47	1.531331812	0.5861947 88	2.612325873	0.008992 849	0.03818 0606
ENSMUSG00000031015	1779.3596 11	1.531787226	0.2345022 08	6.532080187	6.49E-11	1.58E-09
ENSMUSG00000020937	83.804149 52	1.532799824	0.4215614 46	3.636005709	0.000276 898	0.00197 7535
ENSMUSG00000074813	180.17838 43	1.534320808	0.3236542 59	4.740616773	2.13E-06	2.49E-05
ENSMUSG00000023460	560.09456 56	1.535924301	0.2718203 2	5.65051318	1.60E-08	2.69E-07
ENSMUSG00000037148	1161.5932 04	1.537469782	0.2369004 32	6.489940819	8.59E-11	2.04E-09
ENSMUSG00000056687	30.378195 46	1.538023747	0.5876034 91	2.617451683	0.008858 903	0.03771 5986
ENSMUSG00000049401	2493.3938 26	1.539567813	0.2300351 71	6.692749652	2.19E-11	5.65E-10
ENSMUSG00000037816	294.15232 54	1.540425987	0.2829489 65	5.44418315	5.20E-08	8.10E-07
ENSMUSG00000031429	1031.4238 76	1.541620951	0.2535146 52	6.080993498	1.19E-09	2.40E-08
ENSMUSG00000022848	421.03191 61	1.543888802	0.3033079 84	5.09016868	3.58E-07	4.83E-06
ENSMUSG00000023186	2118.8658 32	1.544041697	0.2570465 77	6.006855707	1.89E-09	3.69E-08
ENSMUSG00000037172	1496.0685 92	1.544152093	0.2475537 97	6.237642538	4.44E-10	9.53E-09
ENSMUSG00000030681	1906.7614 48	1.548664691	0.2355485 73	6.574714809	4.87E-11	1.21E-09
ENSMUSG00000052477	63.057309	1.549033936	0.5033106 45	3.077689599	0.002086 121	0.01124 7162
ENSMUSG00000021892	263.45344 15	1.550187202	0.3008503 35	5.15268564	2.57E-07	3.61E-06
ENSMUSG00000031504	206.72568 39	1.550192995	0.3121817 73	4.965674263	6.85E-07	8.65E-06
ENSMUSG00000039701	210.73057 48	1.551857516	0.3335045 09	4.653183018	3.27E-06	3.66E-05
ENSMUSG00000063236	196.65008 39	1.556567503	0.3118949 83	4.990678231	6.02E-07	7.71E-06
ENSMUSG00000019564	290.39048 27	1.558887143	0.2912037 33	5.353252616	8.64E-08	1.31E-06
ENSMUSG00000024143	1143.9569 54	1.558993711	0.2345720 65	6.646118369	3.01E-11	7.68E-10
ENSMUSG00000024953	1647.3784	1.561061489	0.2475993	6.304789514	2.89E-10	6.33E-09

	6		03			
ENSMUSG00000010095	1834.196498	1.565408157	0.226218487	6.919894909	4.52E-12	1.26E-10
ENSMUSG00000021768	30.06538725	1.565434886	0.583865011	2.681158926	0.007336766	0.032300155
ENSMUSG00000038034	2596.951354	1.566784274	0.279825188	5.59915383	2.15E-08	3.56E-07
ENSMUSG00000048118	363.4593728	1.566899549	0.307584757	5.09420417	3.50E-07	4.73E-06
ENSMUSG00000050565	4342.696209	1.56727796	0.232502094	6.740919761	1.57E-11	4.16E-10
ENSMUSG00000069206	167.5719666	1.567611482	0.348739398	4.495079966	6.95E-06	7.25E-05
ENSMUSG00000000682	2357.689804	1.56930455	0.252986621	6.20311281	5.54E-10	1.17E-08
ENSMUSG00000034574	986.0316242	1.569895891	0.256140302	6.129046782	8.84E-10	1.81E-08
ENSMUSG00000002814	467.6354262	1.575798714	0.28057757	5.616267603	1.95E-08	3.23E-07
ENSMUSG00000001166	148.6214069	1.576200145	0.340527063	4.628707422	3.68E-06	4.07E-05
ENSMUSG00000043017	763.9646586	1.576251029	0.268267586	5.875667109	4.21E-09	7.79E-08
ENSMUSG00000096917	80.95271428	1.577279878	0.413028062	3.818820129	0.000134091	0.001051931
ENSMUSG00000029254	1822.761849	1.577829442	0.316069936	4.992026322	5.97E-07	7.67E-06
ENSMUSG00000047123	115.8472598	1.577856038	0.362969866	4.34707171	1.38E-05	0.000135156
ENSMUSG00000069892	46.6711739	1.579237241	0.521360193	3.029071382	0.002453067	0.012920555
ENSMUSG00000028042	960.8421717	1.579350271	0.25542522	6.183219766	6.28E-10	1.33E-08
ENSMUSG00000042659	656.2481458	1.581495546	0.276008306	5.729883885	1.00E-08	1.75E-07
ENSMUSG00000077704	208.8208329	1.583450126	0.323107817	4.900686527	9.55E-07	1.18E-05
ENSMUSG00000021890	1862.433691	1.585154472	0.226439075	7.000357486	2.55E-12	7.26E-11
ENSMUSG00000036002	165.395579	1.588347212	0.345468371	4.597663192	4.27E-06	4.65E-05
ENSMUSG00000084145	148.8073577	1.588501136	0.33772947	4.703472088	2.56E-06	2.93E-05
ENSMUSG00000024238	28.59294181	1.58875784	0.584103431	2.719994023	0.00652831	0.029353833
ENSMUSG00000026466	1285.291215	1.59016267	0.309290278	5.141327688	2.73E-07	3.81E-06
ENSMUSG00000024561	356.6451107	1.590863497	0.281045163	5.660526167	1.51E-08	2.55E-07
ENSMUSG00000065273	67.33486273	1.591505239	0.484155617	3.287177063	0.001011972	0.006117809
ENSMUSG00000061780	39.68734867	1.59386843	0.586291736	2.718558582	0.006556704	0.029462175
ENSMUSG00000049988	670.64184	1.597740569	0.2600694	6.143514058	8.07E-10	1.66E-08

	36		9			
ENSMUSG00000020393	229.88986<br>58	1.600335524	0.3707940<br>77	4.315968413	1.59E-05	0.00015<br>2935
ENSMUSG00000049657	104.76570<br>72	1.602310171	0.3751389<br>88	4.271244054	1.94E-05	0.00018<br>3601
ENSMUSG00000048285	31.204227<br>98	1.603145096	0.5684497<br>18	2.820205629	0.004799<br>288	0.02275<br>1383
ENSMUSG00000039195	385.70644<br>65	1.604232346	0.3055293<br>62	5.250665057	1.52E-07	2.20E-06
ENSMUSG00000026988	156.80386<br>99	1.60439168	0.3373525<br>53	4.755830849	1.98E-06	2.32E-05
ENSMUSG00000020134	890.63277<br>88	1.606097262	0.2617512<br>45	6.135967991	8.46E-10	1.74E-08
ENSMUSG00000036537	76.783061<br>66	1.609100003	0.4310695<br>58	3.732808253	0.000189<br>357	0.00142<br>9826
ENSMUSG00000025969	3295.2529<br>59	1.610840306	0.2178036<br>08	7.395838471	1.41E-13	4.72E-12
ENSMUSG00000013418	40.462228<br>51	1.615310544	0.5375840<br>82	3.004758882	0.002657<br>915	0.01385<br>9903
ENSMUSG00000025911	120.51343<br>21	1.615365503	0.3712987<br>53	4.350581552	1.36E-05	0.00013<br>3201
ENSMUSG00000015947	3042.2248<br>46	1.615598047	0.2343103<br>02	6.895121709	5.38E-12	1.48E-10
ENSMUSG00000033088	234.38975<br>48	1.615629487	0.3115876<br>57	5.185152407	2.16E-07	3.06E-06
ENSMUSG00000020275	285.71180<br>81	1.61751609	0.2926512<br>41	5.527111673	3.26E-08	5.23E-07
ENSMUSG00000002996	1706.9133<br>99	1.621758949	0.2611828<br>24	6.209286366	5.32E-10	1.13E-08
ENSMUSG00000037613	76.030094<br>39	1.622150682	0.4444957<br>18	3.649418022	0.000262<br>835	0.00188<br>8958
ENSMUSG00000031934	999.29028<br>02	1.622603786	0.2534657<br>39	6.401669087	1.54E-10	3.53E-09
ENSMUSG00000048234	2952.5976<br>7	1.622741389	0.2237587<br>59	7.252191583	4.10E-13	1.29E-11
ENSMUSG00000045005	143.56345<br>87	1.623390441	0.3409779<br>75	4.760983293	1.93E-06	2.26E-05
ENSMUSG00000020108	125.27990<br>01	1.627701387	0.4350910<br>23	3.741059463	0.000183<br>246	0.00138<br>8353
ENSMUSG00000029185	141.23535<br>25	1.628012077	0.3600445<br>86	4.521695754	6.13E-06	6.47E-05
ENSMUSG00000028035	606.50362<br>2	1.628389029	0.2980011<br>97	5.464370758	4.65E-08	7.27E-07
ENSMUSG00000017428	750.69632<br>71	1.630217756	0.2626210<br>32	6.207491232	5.38E-10	1.15E-08
ENSMUSG00000015377	43.860320<br>07	1.630392415	0.5041518<br>86	3.233931004	0.001220<br>989	0.00720<br>6572
ENSMUSG00000025877	5530.6496<br>69	1.631000891	0.2503555<br>58	6.51473809	7.28E-11	1.75E-09
ENSMUSG00000022639	319.37515<br>36	1.633983121	0.2837793<br>11	5.757936045	8.51E-09	1.51E-07
ENSMUSG00000064844	150.53854<br>43	1.634885711	0.4317596<br>49	3.786564388	0.000152<br>745	0.00118<br>4029
ENSMUSG00000032249	434.03760	1.63567729	0.2702203	6.053124877	1.42E-09	2.83E-08

	82		11			
ENSMUSG00000039501	3161.018362	1.639267689	0.230717934	7.105072671	1.20E-12	3.54E-11
ENSMUSG00000085148	417.2868769	1.643093492	0.374988555	4.381716377	1.18E-05	0.000117619
ENSMUSG00000034765	208.9340521	1.643235176	0.342080807	4.803646226	1.56E-06	1.85E-05
ENSMUSG00000048164	154.3352897	1.643938287	0.480714604	3.419780202	0.000626718	0.004043862
ENSMUSG00000035671	333.1644451	1.649412901	0.30248918	5.452799667	4.96E-08	7.72E-07
ENSMUSG00000032350	860.9069396	1.649652795	0.257576951	6.404504712	1.51E-10	3.47E-09
ENSMUSG00000011114	2176.101735	1.649686873	0.238214057	6.925228892	4.35E-12	1.21E-10
ENSMUSG00000031662	1131.009764	1.650459313	0.267401973	6.172203206	6.73E-10	1.42E-08
ENSMUSG00000021067	1199.492026	1.650711729	0.262739853	6.282684987	3.33E-10	7.22E-09
ENSMUSG00000093765	31.30755719	1.653611048	0.652119714	2.535747672	0.011220755	0.045443395
ENSMUSG00000039285	1326.515009	1.653692825	0.245865945	6.725993813	1.74E-11	4.58E-10
ENSMUSG00000044864	675.8081169	1.654865994	0.262226959	6.310815642	2.78E-10	6.11E-09
ENSMUSG00000047509	45.47365033	1.65699594	0.522681528	3.170182702	0.001523431	0.008672065
ENSMUSG00000058317	92.20675647	1.65836944	0.394529992	4.20340525	2.63E-05	0.00024168
ENSMUSG00000091764	22.14037434	1.658687026	0.638791702	2.596600772	0.009415128	0.039557531
ENSMUSG00000022636	3351.205535	1.66121257	0.25116888	6.613926736	3.74E-11	9.46E-10
ENSMUSG00000024589	397.4859075	1.661305294	0.284497685	5.839433429	5.24E-09	9.47E-08
ENSMUSG00000031596	158.1354472	1.665083387	0.34682343	4.800954157	1.58E-06	1.88E-05
ENSMUSG00000019944	74.57255656	1.666290254	0.457590179	3.641446711	0.00027111	0.001941257
ENSMUSG00000027610	354.6778443	1.666548906	0.274683142	6.067168495	1.30E-09	2.60E-08
ENSMUSG00000038550	36.0969191	1.667236827	0.5597734	2.978413811	0.002897445	0.014905963
ENSMUSG00000015697	741.7282075	1.669290501	0.253699012	6.579806864	4.71E-11	1.17E-09
ENSMUSG00000025340	291.4046135	1.671919837	0.308866906	5.413075358	6.20E-08	9.56E-07
ENSMUSG00000079038	27.09776702	1.673478434	0.618379052	2.706234031	0.006805107	0.030398954
ENSMUSG00000020225	899.9911176	1.675512682	0.255039887	6.56961036	5.04E-11	1.25E-09
ENSMUSG00000019732	46.10416342	1.678338655	0.499008633	3.363345933	0.000770038	0.004836559
ENSMUSG00000062300	248.83506	1.681574314	0.3314743	5.073015416	3.92E-07	5.23E-06

	39		16			
ENSMUSG00000034731	270.985949	1.684224772	0.318607532	5.286205131	1.25E-07	1.84E-06
ENSMUSG00000021640	21.6257155	1.685042326	0.644624426	2.613990811	0.008949144	0.038053994
ENSMUSG00000029207	511.1754472	1.688713178	0.265610413	6.357857589	2.05E-10	4.60E-09
ENSMUSG00000037447	207.8991689	1.689530018	0.318728092	5.300850664	1.15E-07	1.71E-06
ENSMUSG00000035248	779.0633175	1.690650501	0.261625759	6.462094964	1.03E-10	2.43E-09
ENSMUSG00000049488	114.1410644	1.690762423	0.386990165	4.369006184	1.25E-05	0.000123417
ENSMUSG00000030157	250.4004427	1.691277263	0.323031391	5.235643679	1.64E-07	2.37E-06
ENSMUSG00000045362	1248.051981	1.691651143	0.280193785	6.037432782	1.57E-09	3.10E-08
ENSMUSG00000022893	1343.147545	1.694900824	0.238822519	7.096905398	1.28E-12	3.74E-11
ENSMUSG00000047067	135.5476644	1.695048323	0.347860029	4.872788427	1.10E-06	1.35E-05
ENSMUSG00000056888	1932.696535	1.697382185	0.280555769	6.050070511	1.45E-09	2.88E-08
ENSMUSG00000022906	2538.209332	1.704408476	0.291423567	5.848560892	4.96E-09	9.00E-08
ENSMUSG00000020868	420.6559239	1.707096323	0.298279239	5.723148314	1.05E-08	1.81E-07
ENSMUSG00000037434	616.2077094	1.715375512	0.26387401	6.500736891	7.99E-11	1.91E-09
ENSMUSG00000091561	36.76217474	1.71981723	0.551637076	3.117660699	0.001822925	0.010070164
ENSMUSG00000026864	30831.62985	1.720144929	0.243156681	7.074224419	1.50E-12	4.38E-11
ENSMUSG00000038540	23.97781649	1.727625353	0.643950045	2.682856174	0.007299638	0.032167696
ENSMUSG00000049680	621.8776087	1.728468967	0.26257777	6.582693446	4.62E-11	1.16E-09
ENSMUSG00000056501	2289.138301	1.731137185	0.28310551	6.114812765	9.67E-10	1.97E-08
ENSMUSG00000031792	1010.961409	1.732783584	0.238507579	7.265109108	3.73E-13	1.18E-11
ENSMUSG00000025278	904.2036767	1.73397693	0.251327773	6.899265088	5.23E-12	1.45E-10
ENSMUSG00000046169	58.37366346	1.73472919	0.466848396	3.715829815	0.000202538	0.001515992
ENSMUSG00000035399	92.75371774	1.734821013	0.391671175	4.429279257	9.45E-06	9.60E-05
ENSMUSG00000036986	128.988304	1.735856025	0.420650458	4.126599634	3.68E-05	0.00032968
ENSMUSG00000029186	1616.69431	1.740904256	0.234871763	7.412147941	1.24E-13	4.21E-12
ENSMUSG00000019979	1116.684441	1.74531256	0.238432269	7.31995113	2.48E-13	8.04E-12
ENSMUSG00000030469	122.59727	1.746109356	0.3708890	4.707902331	2.50E-06	2.88E-05

	01		36			
ENSMUSG00000049659	2233.7747 27	1.746974032	0.2653415 51	6.58386908	4.58E-11	1.15E-09
ENSMUSG00000022867	2567.4230 62	1.74808171	0.2676262 45	6.53180226	6.50E-11	1.58E-09
ENSMUSG00000088148	168.04704 03	1.749024807	0.4392605 66	3.981747836	6.84E-05	0.00057 9458
ENSMUSG00000038774	1199.9407 17	1.749590595	0.2691130 3	6.501322482	7.96E-11	1.90E-09
ENSMUSG00000044827	402.91895 09	1.749717415	0.3051180 08	5.734559646	9.78E-09	1.71E-07
ENSMUSG00000047098	214.94505 33	1.750171043	0.3228897 2	5.420336841	5.95E-08	9.19E-07
ENSMUSG00000024404	2434.8986 19	1.752765854	0.2430460 67	7.211661042	5.53E-13	1.71E-11
ENSMUSG00000036390	96.381271 04	1.753607029	0.4362214 75	4.01999243	5.82E-05	0.00050 134
ENSMUSG00000046805	4830.8422 56	1.754478864	0.2759988 81	6.356833255	2.06E-10	4.63E-09
ENSMUSG00000081875	20.203312 97	1.756944053	0.6734956 68	2.60869392	0.009088 85	0.03848 0904
ENSMUSG00000032690	582.03076 81	1.758377801	0.2746388 8	6.40250864	1.53E-10	3.51E-09
ENSMUSG00000042726	1812.8897 42	1.758936149	0.2345018 53	7.500734539	6.35E-14	2.22E-12
ENSMUSG00000020077	847.80832 26	1.759862981	0.2964525 36	5.936407247	2.91E-09	5.53E-08
ENSMUSG00000027381	1004.1440 85	1.764556333	0.2395142 37	7.367229405	1.74E-13	5.80E-12
ENSMUSG00000032688	672.46392 03	1.767615898	0.2489336 47	7.100751228	1.24E-12	3.64E-11
ENSMUSG00000038507	2075.3012 59	1.768499776	0.2595160 82	6.81460571	9.45E-12	2.54E-10
ENSMUSG00000026104	917.48180 7	1.769148341	0.3104603 29	5.698468299	1.21E-08	2.07E-07
ENSMUSG00000038508	578.84710 66	1.769345102	0.3043436 71	5.813641847	6.11E-09	1.10E-07
ENSMUSG00000017057	1089.8651 05	1.770418876	0.2800598 59	6.321573109	2.59E-10	5.73E-09
ENSMUSG00000032281	29.618378 86	1.770626736	0.6298296 46	2.811278806	0.004934 501	0.02329 5725
ENSMUSG00000056144	30.210425 91	1.772651711	0.5790785 78	3.061159193	0.002204 818	0.01178 0521
ENSMUSG00000015709	37.401925 72	1.773006334	0.5357083 64	3.309648411	0.000934 132	0.00569 75
ENSMUSG00000069793	386.05195 13	1.773475046	0.3540234 85	5.009484178	5.46E-07	7.05E-06
ENSMUSG00000059089	1902.3062 91	1.774510692	0.2807024 69	6.321678253	2.59E-10	5.73E-09
ENSMUSG00000062593	1920.6949 19	1.77516326	0.3211725 51	5.52713254	3.26E-08	5.23E-07
ENSMUSG00000078151	55.169856 16	1.775251908	0.4894554 69	3.626993712	0.000286 74	0.00204 0382
ENSMUSG00000021281	7238.4493	1.778579931	0.2465231	7.214658016	5.41E-13	1.68E-11

	99		1			
ENSMUSG00000088929	85.32387774	1.779378859	0.450428403	3.950414426	7.80E-05	0.00064958
ENSMUSG00000038058	108.1979705	1.780484469	0.375424738	4.74258696	2.11E-06	2.47E-05
ENSMUSG00000051413	130.7983389	1.780990649	0.353243852	5.041816405	4.61E-07	6.06E-06
ENSMUSG00000024892	264.4821564	1.781841515	0.299255447	5.954249225	2.61E-09	5.00E-08
ENSMUSG00000087790	17.28493834	1.782273363	0.714073519	2.495924182	0.012562945	0.049819202
ENSMUSG00000029826	3603.098675	1.784710963	0.221632166	8.052580989	8.11E-16	3.61E-14
ENSMUSG00000028300	1034.090276	1.784733703	0.235827051	7.56797703	3.79E-14	1.37E-12
ENSMUSG00000008384	401.9183765	1.787303261	0.289719488	6.169081946	6.87E-10	1.44E-08
ENSMUSG00000022895	308.2201973	1.791583099	0.299323212	5.985446583	2.16E-09	4.20E-08
ENSMUSG00000001542	868.2136836	1.791714062	0.291071824	6.155573684	7.48E-10	1.56E-08
ENSMUSG00000002233	2617.158588	1.795602323	0.233652881	7.684914124	1.53E-14	5.83E-13
ENSMUSG00000020653	27.60362431	1.796239861	0.593354322	3.027263466	0.002467788	0.012983121
ENSMUSG00000028954	1868.726093	1.797969138	0.247835234	7.254695414	4.03E-13	1.27E-11
ENSMUSG00000053835	397.8800117	1.79808721	0.307433954	5.848694279	4.95E-09	9.00E-08
ENSMUSG00000072235	761.6919649	1.8033668	0.324361298	5.559747139	2.70E-08	4.41E-07
ENSMUSG00000003882	502.9693489	1.803898705	0.367854221	4.903841255	9.40E-07	1.17E-05
ENSMUSG00000048856	23.8612254	1.80824958	0.622602899	2.904338516	0.0036803	0.018235181
ENSMUSG00000058163	146.8004201	1.810541515	0.428852309	4.221829929	2.42E-05	0.000224398
ENSMUSG00000027808	14363.19808	1.81324144	0.24342788	7.448782953	9.42E-14	3.23E-12
ENSMUSG00000025372	264.9701359	1.813700132	0.304620803	5.953960174	2.62E-09	5.00E-08
ENSMUSG00000052144	256.1642277	1.814884647	0.316122124	5.74108709	9.41E-09	1.65E-07
ENSMUSG00000074578	1065.980745	1.816793119	0.262296787	6.926478752	4.31E-12	1.20E-10
ENSMUSG00000094242	98.64261775	1.817508671	0.396237862	4.586913173	4.50E-06	4.88E-05
ENSMUSG00000078453	675.192198	1.824993219	0.294071097	6.205959156	5.44E-10	1.16E-08
ENSMUSG00000019813	179.4921252	1.82507564	0.358095852	5.096612066	3.46E-07	4.68E-06
ENSMUSG00000040562	116.7439824	1.830711117	0.37775574	4.846282728	1.26E-06	1.52E-05
ENSMUSG00000040528	2165.5793	1.831618266	0.2669469	6.861357173	6.82E-12	1.85E-10

	87		35			
ENSMUSG00000035004	1688.26417	1.834709461	0.26633828	6.888643495	5.63E-12	1.54E-10
ENSMUSG00000046546	18.20600437	1.838196972	0.702830771	2.615419029	0.008911805	0.037906979
ENSMUSG00000038217	28.11071678	1.839192982	0.595319354	3.08942246	0.00200546	0.010885084
ENSMUSG00000087912	50.04960885	1.840043652	0.628217145	2.928993048	0.00340062	0.017113581
ENSMUSG00000020901	735.810124	1.840239598	0.257745853	7.139744726	9.35E-13	2.79E-11
ENSMUSG00000059839	219.0356008	1.840652534	0.342991467	5.366467416	8.03E-08	1.22E-06
ENSMUSG00000068040	807.6784331	1.841016701	0.251931583	7.30760582	2.72E-13	8.71E-12
ENSMUSG00000071064	28.36866678	1.841201971	0.589986666	3.120751835	0.0018039	0.009996946
ENSMUSG00000029009	1223.998158	1.847376419	0.255733771	7.223826606	5.05E-13	1.58E-11
ENSMUSG00000064741	259.3186263	1.850404345	0.313353614	5.90516357	3.52E-09	6.65E-08
ENSMUSG00000040128	247.5835446	1.850510134	0.360191885	5.137567534	2.78E-07	3.87E-06
ENSMUSG00000036718	63.99337333	1.854749712	0.452867118	4.095571614	4.21E-05	0.00037284
ENSMUSG00000071350	341.7329056	1.855466888	0.364031877	5.096990135	3.45E-07	4.67E-06
ENSMUSG00000040152	20.46775578	1.860552452	0.66254899	2.808173402	0.004982339	0.02344983
ENSMUSG00000090224	20.16963646	1.860556946	0.7243395	2.568625548	0.010210272	0.042211697
ENSMUSG00000042350	1453.730039	1.861188268	0.234297959	7.943681112	1.96E-15	8.28E-14
ENSMUSG00000026946	1239.054757	1.861939233	0.335055788	5.557102121	2.74E-08	4.46E-07
ENSMUSG00000031709	688.3890526	1.861993802	0.248211353	7.501646404	6.30E-14	2.21E-12
ENSMUSG00000066861	585.4529497	1.862686096	0.260518153	7.149928228	8.68E-13	2.60E-11
ENSMUSG00000020806	182.6810472	1.862948787	0.338421568	5.504816961	3.70E-08	5.85E-07
ENSMUSG00000026942	275.3292753	1.866503927	0.331453143	5.631275395	1.79E-08	2.98E-07
ENSMUSG00000032238	73.08751518	1.868214023	0.448375269	4.166630397	3.09E-05	0.000281134
ENSMUSG00000028124	658.4796848	1.868633349	0.303986981	6.147083477	7.89E-10	1.63E-08
ENSMUSG00000021929	768.5430164	1.869529525	0.288503448	6.480094215	9.17E-11	2.17E-09
ENSMUSG00000019505	1817.615601	1.870423888	0.230665433	8.108817463	5.11E-16	2.34E-14
ENSMUSG00000032596	586.4506671	1.870475773	0.267108006	7.002694526	2.51E-12	7.15E-11
ENSMUSG00000048832	255.81013	1.871495475	0.3070659	6.094766506	1.10E-09	2.21E-08

	6		84			
ENSMUSG00000074151	1129.834644	1.871519727	0.243989748	7.670485102	1.71E-14	6.49E-13
ENSMUSG00000023951	440.7528425	1.871761312	0.275239489	6.800482444	1.04E-11	2.79E-10
ENSMUSG00000029333	850.3787246	1.874546808	0.241118456	7.774381259	7.58E-15	2.99E-13
ENSMUSG00000047945	147.4320158	1.877927902	0.362989361	5.173506728	2.30E-07	3.24E-06
ENSMUSG00000031586	30.83648465	1.878386867	0.581286747	3.231429027	0.001231729	0.007254339
ENSMUSG00000091144	23.9112683	1.878659719	0.625247162	3.004667328	0.002658715	0.013859903
ENSMUSG00000032860	126.3896772	1.879212667	0.368504577	5.099563983	3.40E-07	4.63E-06
ENSMUSG00000064289	1689.095994	1.880849942	0.287300372	6.546632459	5.88E-11	1.45E-09
ENSMUSG00000031639	276.4524306	1.882954116	0.415663187	4.529999706	5.90E-06	6.23E-05
ENSMUSG00000045868	20.53832044	1.883090517	0.670185535	2.809804777	0.004957156	0.023378466
ENSMUSG00000052631	13.86121073	1.883378464	0.734837011	2.562988033	0.010377563	0.042787152
ENSMUSG00000022707	975.1414518	1.883978547	0.270601579	6.962186091	3.35E-12	9.43E-11
ENSMUSG00000092564	141.945437	1.884531595	0.38004836	4.958662618	7.10E-07	8.93E-06
ENSMUSG00000045381	18.79758332	1.884726567	0.677562307	2.781628416	0.005408693	0.025170165
ENSMUSG00000028459	821.6366328	1.887051449	0.269621504	6.998890757	2.58E-12	7.32E-11
ENSMUSG00000028378	1065.380338	1.889544268	0.275002098	6.871017638	6.37E-12	1.74E-10
ENSMUSG00000028163	1847.568131	1.889603724	0.224582242	8.41386082	3.97E-17	2.01E-15
ENSMUSG00000032265	1026.738666	1.894095636	0.24991708	7.578896303	3.49E-14	1.26E-12
ENSMUSG00000098557	587.4902476	1.894106495	0.274850837	6.891397962	5.52E-12	1.52E-10
ENSMUSG00000066258	182.1354073	1.899863822	0.378752542	5.016108442	5.27E-07	6.84E-06
ENSMUSG00000035560	22.97939248	1.901768029	0.659439244	2.883916975	0.003927623	0.01923118
ENSMUSG00000004947	185.7490825	1.902323611	0.336171177	5.658794508	1.52E-08	2.58E-07
ENSMUSG00000022237	99.73760789	1.907592283	0.396951282	4.805608072	1.54E-06	1.84E-05
ENSMUSG00000038368	449.1240499	1.908519033	0.264930248	7.203854789	5.85E-13	1.80E-11
ENSMUSG00000041992	672.7943072	1.908808772	0.261177513	7.308472898	2.70E-13	8.67E-12
ENSMUSG00000026740	609.8107689	1.910506871	0.284201355	6.722370744	1.79E-11	4.68E-10
ENSMUSG00000038412	172.78701	1.910660097	0.3661201	5.218670062	1.80E-07	2.57E-06

	17		18			
ENSMUSG00000084110	34.17512031	1.910756504	0.575346437	3.321053856	0.000896782	0.005498737
ENSMUSG00000022014	792.7662097	1.9127125	0.301197166	6.350366861	2.15E-10	4.82E-09
ENSMUSG00000004040	1031.099421	1.913212508	0.24243989	7.891492215	2.99E-15	1.23E-13
ENSMUSG00000020415	495.5953317	1.913651344	0.286822104	6.671910283	2.52E-11	6.47E-10
ENSMUSG00000078485	116.6574861	1.919792463	0.377223883	5.089265415	3.59E-07	4.84E-06
ENSMUSG00000040296	1440.246085	1.928764797	0.287829013	6.701078452	2.07E-11	5.36E-10
ENSMUSG00000041153	497.5394144	1.930356906	0.297710952	6.483996948	8.93E-11	2.12E-09
ENSMUSG00000070327	12152.75329	1.931542042	0.216879019	8.906080666	5.29E-19	3.16E-17
ENSMUSG00000021930	1047.616729	1.931855133	0.258491813	7.473564102	7.81E-14	2.69E-12
ENSMUSG00000039879	494.588497	1.932057081	0.262833702	7.350872694	1.97E-13	6.53E-12
ENSMUSG00000079197	637.4035984	1.936227881	0.255148442	7.588632978	3.23E-14	1.18E-12
ENSMUSG00000043822	189.6938606	1.937261903	0.334244649	5.795939922	6.79E-09	1.22E-07
ENSMUSG00000072621	508.1398954	1.93909049	0.266598533	7.273447701	3.50E-13	1.11E-11
ENSMUSG00000030595	172.910582	1.94154753	0.343758552	5.64799775	1.62E-08	2.73E-07
ENSMUSG00000099843	76.47646846	1.941639452	0.424500878	4.573935063	4.79E-06	5.16E-05
ENSMUSG00000015312	904.9959826	1.9422486	0.277621633	6.996027579	2.63E-12	7.46E-11
ENSMUSG00000056962	727.8500971	1.943708939	0.2577886	7.539933633	4.70E-14	1.67E-12
ENSMUSG00000039531	219.9377079	1.945760775	0.37081415	5.247266787	1.54E-07	2.23E-06
ENSMUSG00000023952	1428.167431	1.947322869	0.265388598	7.337628225	2.17E-13	7.15E-12
ENSMUSG00000058755	169.4171019	1.947357179	0.331361098	5.876843083	4.18E-09	7.75E-08
ENSMUSG00000027660	436.724197	1.949291357	0.265689438	7.336728835	2.19E-13	7.18E-12
ENSMUSG00000052776	1969.99123	1.954484116	0.248243817	7.873243899	3.46E-15	1.42E-13
ENSMUSG00000023088	1763.417473	1.955765744	0.23227894	8.419901293	3.77E-17	1.93E-15
ENSMUSG00000033713	191.0072006	1.955949873	0.322038674	6.073649005	1.25E-09	2.51E-08
ENSMUSG00000065110	47.11834682	1.957711211	0.528657487	3.703175043	0.000212918	0.001577319
ENSMUSG00000060477	541.1875616	1.957927344	0.267250416	7.326190081	2.37E-13	7.75E-12
ENSMUSG00000022724	667.74587	1.958052096	0.2490284	7.862763387	3.76E-15	1.54E-13

	65		9			
ENSMUSG00000028437	601.0406451	1.959760393	0.278462219	7.037796363	1.95E-12	5.65E-11
ENSMUSG00000024338	2085.016254	1.961349348	0.22610373	8.674555489	4.15E-18	2.29E-16
ENSMUSG00000001123	444.9437856	1.961813623	0.306665707	6.397238353	1.58E-10	3.63E-09
ENSMUSG00000025888	3031.858834	1.964554601	0.344602311	5.700932754	1.19E-08	2.05E-07
ENSMUSG00000040511	391.5517041	1.966031273	0.273075299	7.19959395	6.04E-13	1.85E-11
ENSMUSG00000029992	1129.495745	1.966732715	0.237689053	8.274393325	1.29E-16	6.12E-15
ENSMUSG00000000555	285.645095	1.966742066	0.325467909	6.042814083	1.51E-09	3.00E-08
ENSMUSG00000090145	11.51449876	1.967507334	0.782970373	2.512875841	0.011975147	0.047904084
ENSMUSG00000020902	62.78691084	1.970684941	0.460260769	4.281670467	1.85E-05	0.000176054
ENSMUSG00000025791	1301.384527	1.972693537	0.245513761	8.034961173	9.36E-16	4.14E-14
ENSMUSG00000029084	141.3339976	1.973611355	0.36107595	5.465917507	4.61E-08	7.21E-07
ENSMUSG00000020707	334.7196768	1.976061982	0.286470224	6.897966397	5.28E-12	1.45E-10
ENSMUSG00000059495	288.274608	1.976801741	0.303870086	6.505417387	7.75E-11	1.86E-09
ENSMUSG00000003849	24.35827516	1.980887899	0.635319363	3.117940387	0.001821196	0.010070164
ENSMUSG00000036478	445.383679	1.98508718	0.27538737	7.208345023	5.66E-13	1.74E-11
ENSMUSG00000056130	145.5212638	1.986257511	0.35258632	5.63339358	1.77E-08	2.95E-07
ENSMUSG00000020572	9656.557396	1.987134067	0.258406018	7.689968216	1.47E-14	5.62E-13
ENSMUSG00000027074	812.0007598	1.988229	0.24177303	8.223535118	1.98E-16	9.27E-15
ENSMUSG00000040297	513.522224	1.991531313	0.288211431	6.909966429	4.85E-12	1.34E-10
ENSMUSG00000035235	98.28257065	1.995335392	0.414893828	4.80926747	1.51E-06	1.81E-05
ENSMUSG00000056671	147.7770267	1.995665177	0.348602357	5.724760993	1.04E-08	1.79E-07
ENSMUSG00000027366	5380.550639	1.999058518	0.277910242	7.193180455	6.33E-13	1.93E-11
ENSMUSG00000024155	22.51588034	1.999742069	0.649378778	3.079469389	0.002073697	0.011188982
ENSMUSG00000037997	400.4400089	2.003783614	0.299112475	6.699097442	2.10E-11	5.42E-10
ENSMUSG00000000078	3895.01022	2.00665174	0.242829774	8.263614907	1.41E-16	6.68E-15
ENSMUSG00000031936	24.63925349	2.012624524	0.631547537	3.186813986	0.001438493	0.008283639
ENSMUSG00000085701	23.942030	2.014106879	0.6399200	3.147435007	0.001647	0.00927

	6		85		097	4226
ENSMUSG00000029552	1140.681822	2.014201535	0.24142049	8.343125883	7.24E-17	3.53E-15
ENSMUSG00000090272	810.9241944	2.017903296	0.752972292	2.679917065	0.00736404	0.032389015
ENSMUSG00000003283	1302.619639	2.020346549	0.258777526	7.80727206	5.84E-15	2.34E-13
ENSMUSG00000010067	445.8900826	2.023312334	0.27851867	7.264548302	3.74E-13	1.18E-11
ENSMUSG00000053395	59.97233951	2.023551793	0.490745757	4.123421884	3.73E-05	0.000333594
ENSMUSG00000102196	14.19671654	2.023928017	0.756150175	2.676621765	0.007436854	0.032677807
ENSMUSG00000023106	1641.992137	2.024067601	0.248545174	8.143660835	3.84E-16	1.78E-14
ENSMUSG00000064647	144.9262906	2.029915394	0.383196913	5.297316671	1.18E-07	1.74E-06
ENSMUSG00000021614	14.07390138	2.031359267	0.742368418	2.736322314	0.006213014	0.028185817
ENSMUSG00000019256	48.89493627	2.03245399	0.501607557	4.05188072	5.08E-05	0.000443557
ENSMUSG00000012519	1877.183179	2.034445564	0.263914324	7.708734928	1.27E-14	4.91E-13
ENSMUSG00000024339	610.1825917	2.038228113	0.286456964	7.115303061	1.12E-12	3.31E-11
ENSMUSG00000087006	14.48552998	2.042655522	0.740443356	2.758692485	0.005803312	0.026636068
ENSMUSG00000031652	1092.114885	2.044834146	0.244843365	8.351601206	6.73E-17	3.32E-15
ENSMUSG00000002983	244.5538529	2.045671193	0.349959615	5.845449312	5.05E-09	9.16E-08
ENSMUSG00000081650	16.3989878	2.046760357	0.712742237	2.8716698	0.004083094	0.019892925
ENSMUSG00000038059	414.6942427	2.047545888	0.299557019	6.835245897	8.19E-12	2.21E-10
ENSMUSG00000038831	97.55512001	2.048233936	0.392165456	5.222882086	1.76E-07	2.52E-06
ENSMUSG00000055926	9.700222303	2.05091676	0.802737379	2.554903776	0.010621716	0.043514155
ENSMUSG00000066152	1445.035847	2.05190096	0.229887846	8.925660891	4.43E-19	2.67E-17
ENSMUSG00000055447	2630.121541	2.053411719	0.271012835	7.576806165	3.54E-14	1.28E-12
ENSMUSG00000073411	10286.48283	2.057897233	0.229636018	8.961561211	3.20E-19	1.97E-17
ENSMUSG00000090881	17.18531911	2.062410487	0.729626094	2.826667666	0.004703511	0.022374739
ENSMUSG00000008348	4996.983279	2.06367689	0.259841284	7.942067011	1.99E-15	8.36E-14
ENSMUSG00000032661	1222.899982	2.071455189	0.23715934	8.734444922	2.45E-18	1.36E-16
ENSMUSG00000029490	74.66674303	2.074787542	0.451812837	4.592139426	4.39E-06	4.77E-05
ENSMUSG00000049502	1106.0559	2.079579741	0.2597700	8.00546334	1.19E-15	5.19E-14

	75		66			
ENSMUSG00000033917	1537.492326	2.080684742	0.231980426	8.969225449	2.99E-19	1.85E-17
ENSMUSG00000023032	11.39603679	2.085902343	0.7870298	2.650347347	0.008040906	0.034951034
ENSMUSG00000026482	2193.060857	2.089236855	0.23913178	8.736759517	2.40E-18	1.34E-16
ENSMUSG00000068113	25.98754146	2.095433979	0.6375962	3.286459331	0.001014554	0.006130717
ENSMUSG00000052837	1445.932968	2.096321503	0.32933762	6.365265848	1.95E-10	4.40E-09
ENSMUSG00000045636	75.66388813	2.096456249	0.43515568	4.817715466	1.45E-06	1.74E-05
ENSMUSG00000052727	48.54135328	2.097654791	0.545183775	3.847610448	0.000119276	0.000948735
ENSMUSG00000033355	1085.946858	2.09977217	0.301758736	6.958447006	3.44E-12	9.66E-11
ENSMUSG00000034118	385.6662952	2.101506018	0.297613638	7.06118856	1.65E-12	4.80E-11
ENSMUSG00000073274	147.8370293	2.10188166	0.427065464	4.921684934	8.58E-07	1.07E-05
ENSMUSG00000021871	2505.500846	2.103529277	0.226124533	9.302525708	1.37E-20	9.35E-19
ENSMUSG00000047842	10.09270169	2.104046938	0.800516966	2.628360207	0.008579761	0.036688183
ENSMUSG00000015335	263.6500102	2.106172598	0.324303407	6.494451029	8.33E-11	1.99E-09
ENSMUSG00000078153	1632.079576	2.106360866	0.250051979	8.423692029	3.65E-17	1.87E-15
ENSMUSG00000059142	335.108709	2.113797686	0.36434449	5.80164582	6.57E-09	1.18E-07
ENSMUSG00000028691	19693.96769	2.11478752	0.276978626	7.635201141	2.25E-14	8.40E-13
ENSMUSG00000089672	767.9517729	2.115510513	0.358678735	5.898065049	3.68E-09	6.92E-08
ENSMUSG00000030966	530.5694879	2.118086015	0.293718352	7.211282508	5.54E-13	1.71E-11
ENSMUSG00000063268	1380.137678	2.122986458	0.237019002	8.957030635	3.34E-19	2.04E-17
ENSMUSG00000033781	68.80786389	2.124537481	0.45139684	4.706584742	2.52E-06	2.90E-05
ENSMUSG00000026893	17.30813426	2.125197526	0.708734602	2.998580175	0.002712408	0.014096909
ENSMUSG00000023914	9.0133411	2.127951314	0.816642793	2.605730844	0.009167847	0.038731611
ENSMUSG00000036067	1707.145535	2.130482792	0.248546529	8.571766438	1.02E-17	5.54E-16
ENSMUSG00000049191	20.27220458	2.131014051	0.715750999	2.977312017	0.002907879	0.014949452
ENSMUSG00000083736	14.34868588	2.134520456	0.77099061	2.76854274	0.00563076	0.025983019
ENSMUSG00000005413	3280.929164	2.136704902	0.242606257	8.8072951	1.28E-18	7.35E-17
ENSMUSG00000040435	883.58893	2.137090958	0.2858705	7.475729266	7.68E-14	2.66E-12

	38		66			
ENSMUSG00000050578	26.82053489	2.138173907	0.641298214	3.334133576	0.000855656	0.00528375
ENSMUSG00000027380	132.7341198	2.140226122	0.361437413	5.921429396	3.19E-09	6.03E-08
ENSMUSG00000026479	255.4143921	2.140944805	0.310346048	6.898572811	5.25E-12	1.45E-10
ENSMUSG00000001435	6.950689256	2.147313585	0.857639101	2.503749634	0.012288496	0.048929063
ENSMUSG00000087141	64.58872871	2.149186155	0.487610822	4.407585019	1.05E-05	0.000105028
ENSMUSG00000010358	1183.572578	2.150332959	0.275863514	7.794916139	6.45E-15	2.57E-13
ENSMUSG00000021996	5420.888905	2.150342995	0.266019077	8.083416502	6.30E-16	2.84E-14
ENSMUSG00000025161	1993.541154	2.151506707	0.257592966	8.352350387	6.69E-17	3.31E-15
ENSMUSG00000025432	20.17814349	2.152241176	0.738786455	2.913211473	0.003577323	0.017808647
ENSMUSG00000026222	371.7608519	2.157741455	0.344094602	6.270779731	3.59E-10	7.77E-09
ENSMUSG00000026072	9.261716491	2.159043366	0.815165269	2.648595871	0.008082692	0.03504375
ENSMUSG00000091898	9.281632788	2.159192603	0.816801788	2.643471935	0.008206057	0.035432895
ENSMUSG00000014905	363.213161	2.159628397	0.359718085	6.003669234	1.93E-09	3.76E-08
ENSMUSG00000043415	109.9240746	2.16203785	0.410264403	5.269864585	1.37E-07	1.99E-06
ENSMUSG00000044434	65.41935293	2.165161774	0.465066824	4.655592833	3.23E-06	3.63E-05
ENSMUSG00000097111	9.42436424	2.165950077	0.817352097	2.649959651	0.008050138	0.034965377
ENSMUSG00000041571	103.8354998	2.168840737	0.405773681	5.344951731	9.04E-08	1.36E-06
ENSMUSG00000031584	1064.96329	2.170210875	0.258702474	8.388829215	4.91E-17	2.46E-15
ENSMUSG00000049037	50.61685338	2.171092235	0.537116606	4.042124583	5.30E-05	0.000460915
ENSMUSG00000040466	1685.111878	2.174978834	0.256424695	8.481939838	2.21E-17	1.16E-15
ENSMUSG00000098050	27.46062259	2.17534163	0.612361705	3.552380256	0.000381763	0.002616029
ENSMUSG00000040852	142.9957944	2.179065567	0.353378668	6.166375515	6.99E-10	1.46E-08
ENSMUSG00000029385	982.096746	2.180185037	0.237819195	9.16740568	4.85E-20	3.18E-18
ENSMUSG00000096727	707.8004687	2.183072344	0.275857236	7.913775899	2.50E-15	1.04E-13
ENSMUSG00000055493	27.48315258	2.183415599	0.634771064	3.439689871	0.000582381	0.003782717
ENSMUSG00000024308	2782.26939	2.183951288	0.241890005	9.028695881	1.74E-19	1.10E-17
ENSMUSG00000027333	718.33385	2.195799908	0.2815937	7.79775765	6.30E-15	2.52E-13

			62			
ENSMUSG00000079190	130.78047 92	2.200509461	0.4192247 95	5.248996452	1.53E-07	2.22E-06
ENSMUSG00000039934	442.33888 48	2.202195699	0.2844890 32	7.740880848	9.87E-15	3.83E-13
ENSMUSG00000063506	334.74842 16	2.206184778	0.2880873 14	7.658042105	1.89E-14	7.11E-13
ENSMUSG00000034248	421.90815 32	2.20840406	0.2788218 05	7.92048548	2.37E-15	9.89E-14
ENSMUSG00000031257	7.1133370 06	2.208452117	0.8516147 01	2.593252695	0.009507 289	0.03984 6297
ENSMUSG00000097430	8.4891076 47	2.209390228	0.8343516 68	2.648032374	0.008096 177	0.03509 1116
ENSMUSG00000020839	5.7856010 29	2.212594629	0.8680137 45	2.549031788	0.010802 245	0.04410 032
ENSMUSG00000033737	28.764993 51	2.216416731	0.6216813	3.565197687	0.000363 582	0.00250 6485
ENSMUSG00000019916	2514.3136 62	2.216996153	0.2285984 04	9.698213608	3.07E-22	2.46E-20
ENSMUSG00000032175	665.53703 65	2.217717127	0.2657506 2	8.345106116	7.11E-17	3.49E-15
ENSMUSG00000072844	61.213573 25	2.219940094	0.4655563 06	4.768360058	1.86E-06	2.19E-05
ENSMUSG00000019779	254.81996 68	2.224240342	0.3126662 95	7.113783547	1.13E-12	3.33E-11
ENSMUSG00000082896	303.60527 29	2.226558777	0.2985915 9	7.456870356	8.86E-14	3.04E-12
ENSMUSG00000020277	1501.5385 43	2.231820128	0.2693487 89	8.285985388	1.17E-16	5.57E-15
ENSMUSG00000016356	4.6582057 79	2.237727878	0.8761972 18	2.553908904	0.010652 112	0.04359 128
ENSMUSG00000021322	830.16929 46	2.238401467	0.2516300 99	8.895603025	5.81E-19	3.43E-17
ENSMUSG00000037966	1544.8122 78	2.24249951	0.2606547 93	8.603331172	7.74E-18	4.24E-16
ENSMUSG00000021457	2289.7801 14	2.242920478	0.2266367 18	9.896545006	4.31E-23	3.76E-21
ENSMUSG00000029822	718.81266 73	2.244815953	0.2508374 54	8.949285348	3.58E-19	2.17E-17
ENSMUSG00000073802	168.96647 56	2.249066705	0.3591337 79	6.262476089	3.79E-10	8.14E-09
ENSMUSG00000000730	246.23769 95	2.254893992	0.3046269 67	7.402148323	1.34E-13	4.52E-12
ENSMUSG00000091613	15.217999 75	2.255741722	0.7447864 88	3.028709243	0.002456 009	0.01293 1082
ENSMUSG00000021565	12.686715 49	2.258710306	0.7746308 06	2.915853965	0.003547 165	0.01768 8095
ENSMUSG00000027555	1121.3794 14	2.259945211	0.2726601 28	8.288506385	1.15E-16	5.50E-15
ENSMUSG00000087477	166.09399	2.259954715	0.3454498 13	6.542063795	6.07E-11	1.48E-09
ENSMUSG00000026785	108.88819 32	2.268877246	0.4017720 92	5.647174829	1.63E-08	2.74E-07
ENSMUSG00000033902	152.47780	2.272780661	0.3547725	6.406303093	1.49E-10	3.45E-09

	42		77			
ENSMUSG00000097000	13.99728025	2.273247563	0.755222839	3.010035512	0.002612171	0.01366405
ENSMUSG00000030401	20.52954912	2.279493701	0.677777736	3.363187634	0.00077048	0.004837116
ENSMUSG00000029409	4.706240443	2.280435679	0.876151252	2.602787674	0.00924692	0.039005553
ENSMUSG00000009596	10.18940671	2.280582313	0.8078764	2.822934688	0.004758626	0.022589877
ENSMUSG00000027605	300.7627116	2.280904119	0.297682607	7.662201504	1.83E-14	6.90E-13
ENSMUSG00000020400	625.8632655	2.283170182	0.254122278	8.984533731	2.60E-19	1.63E-17
ENSMUSG00000079429	38.71329223	2.286908959	0.552110168	4.142124333	3.44E-05	0.000310464
ENSMUSG00000070942	4.768334291	2.288994959	0.876133041	2.612611157	0.008985347	0.038160576
ENSMUSG00000042985	7.734275483	2.290807037	0.848778283	2.698946336	0.006955939	0.030875094
ENSMUSG00000015340	6521.251762	2.293063757	0.269378786	8.51241404	1.70E-17	9.12E-16
ENSMUSG00000031503	15.52398441	2.298243273	0.732917282	3.135747145	0.001714169	0.009584787
ENSMUSG00000097534	34.78571557	2.302771948	0.56733946	4.058896145	4.93E-05	0.00043205
ENSMUSG00000043939	4.960472947	2.303473669	0.876136672	2.629125959	0.008560465	0.036628526
ENSMUSG00000065422	4.754275107	2.303562927	0.876072739	2.62941971	0.008553073	0.036618006
ENSMUSG00000020841	3037.772871	2.306044029	0.227291595	10.14575145	3.46E-24	3.54E-22
ENSMUSG00000097712	9.047952276	2.306603711	0.82605708	2.79230548	0.005233392	0.024479059
ENSMUSG00000061232	8706.029702	2.308081914	0.226886642	10.17284177	2.62E-24	2.72E-22
ENSMUSG00000044098	559.1093592	2.310709936	0.288538527	8.008323746	1.16E-15	5.09E-14
ENSMUSG00000094530	6265.419199	2.316661177	0.217262008	10.66298334	1.52E-26	1.82E-24
ENSMUSG00000084140	8.012141781	2.320977643	0.851558977	2.725563003	0.006419192	0.028996385
ENSMUSG00000046718	8459.385091	2.323732077	0.234192194	9.922329327	3.33E-23	2.98E-21
ENSMUSG00000031897	2604.325176	2.328177691	0.264008457	8.818572397	1.16E-18	6.68E-17
ENSMUSG00000020589	636.0666062	2.330479376	0.274033687	8.504353627	1.83E-17	9.74E-16
ENSMUSG00000045875	72.67421046	2.333660304	0.454012481	5.140079628	2.75E-07	3.83E-06
ENSMUSG00000047746	19.77548403	2.334532362	0.684938914	3.408380387	0.000653497	0.004181223
ENSMUSG00000004952	708.0523392	2.335765389	0.287143666	8.134483429	4.14E-16	1.92E-14
ENSMUSG00000004707	583.23945	2.33858401	0.2596545	9.006521659	2.13E-19	1.34E-17

	9		15			
ENSMUSG00000027452	4.9405566 5	2.342035207	0.8758860 54	2.673903981	0.007497 391	0.03287 0041
ENSMUSG00000102437	5.1326953 07	2.344330915	0.8759797 27	2.676238779	0.007445 358	0.03270 4689
ENSMUSG00000030269	1071.6858 99	2.346455108	0.2422478 22	9.686176274	3.45E-22	2.75E-20
ENSMUSG00000059588	737.67909 41	2.346524408	0.2901507 56	8.087259336	6.10E-16	2.77E-14
ENSMUSG00000044468	1443.6425 76	2.34676698	0.2314736 2	10.13837767	3.73E-24	3.76E-22
ENSMUSG00000026628	6892.7307 42	2.347502493	0.2203801 69	10.65205866	1.71E-26	2.03E-24
ENSMUSG00000042677	74.166440 81	2.348631644	0.4406415 96	5.330027092	9.82E-08	1.47E-06
ENSMUSG00000053175	13.260991 84	2.349762583	0.7614732 91	3.085810904	0.002029 979	0.01100 5087
ENSMUSG00000103439	22.580150 01	2.353496103	0.6542122 89	3.597450161	0.000321 352	0.00225 2752
ENSMUSG00000057137	255.69667 34	2.353548418	0.3034762 86	7.755295983	8.81E-15	3.45E-13
ENSMUSG00000029561	1237.0440 98	2.353572867	0.2942177 12	7.999426173	1.25E-15	5.44E-14
ENSMUSG00000043336	212.57658 73	2.354247659	0.3547962 9	6.635491205	3.23E-11	8.22E-10
ENSMUSG00000038305	4.9745321 31	2.354275125	0.8758245 96	2.688066921	0.007186 699	0.03170 0583
ENSMUSG00000024678	4.9745321 31	2.354275125	0.8758245 96	2.688066921	0.007186 699	0.03170 0583
ENSMUSG00000025854	2167.4351 8	2.355728064	0.2400776 66	9.812358229	9.96E-23	8.43E-21
ENSMUSG00000034640	1724.1055 78	2.356289787	0.2714367 79	8.680805158	3.93E-18	2.17E-16
ENSMUSG00000028517	72.016483 44	2.361702498	0.4711951 23	5.012153949	5.38E-07	6.97E-06
ENSMUSG00000090709	10.529366 86	2.362451744	0.8006683 18	2.950599758	0.003171 576	0.01609 8685
ENSMUSG00000057963	349.85873 14	2.369096831	0.2938331 65	8.062727822	7.46E-16	3.34E-14
ENSMUSG00000056008	30.155489 26	2.3741402	0.6441724 46	3.685566206	0.000228 195	0.00167 2411
ENSMUSG00000020250	4028.1146 42	2.378452954	0.2254412 26	10.55021298	5.07E-26	5.84E-24
ENSMUSG00000045795	283.70994 68	2.380917305	0.2967601 83	8.023034902	1.03E-15	4.55E-14
ENSMUSG00000040033	647.98791 37	2.383366876	0.2615619 15	9.112056232	8.08E-20	5.25E-18
ENSMUSG00000044716	88.178654 45	2.386745171	0.4530137 88	5.268592773	1.37E-07	2.00E-06
ENSMUSG00000038213	315.49993 13	2.387083084	0.2912666 04	8.195526191	2.49E-16	1.17E-14
ENSMUSG00000060519	2734.7785 4	2.388148416	0.2368420 84	10.08329424	6.55E-24	6.32E-22
ENSMUSG00000018143	1144.0715	2.391523549	0.2651933	9.018038407	1.91E-19	1.21E-17

	55		2			
ENSMUSG00000102518	9.550560171	2.391827578	0.819945417	2.917057071	0.003533511	0.01764824
ENSMUSG00000094935	9.550560171	2.391827578	0.819945417	2.917057071	0.003533511	0.01764824
ENSMUSG00000033581	1108.113125	2.392017056	0.257418161	9.292339944	1.51E-20	1.02E-18
ENSMUSG00000078486	143.8793018	2.392019752	0.360079537	6.643031625	3.07E-11	7.83E-10
ENSMUSG00000025225	666.8337308	2.392425806	0.286885089	8.339317386	7.47E-17	3.63E-15
ENSMUSG00000026764	119.3407336	2.395922603	0.381570995	6.279100447	3.41E-10	7.37E-09
ENSMUSG00000008393	599.0240706	2.398960896	0.258573258	9.277683689	1.73E-20	1.16E-18
ENSMUSG00000026031	4870.571779	2.399288165	0.218524115	10.97951211	4.80E-28	6.44E-26
ENSMUSG00000002307	1238.372161	2.404304811	0.249619481	9.631879716	5.86E-22	4.57E-20
ENSMUSG00000043740	120.9820751	2.404978892	0.427781879	5.621974674	1.89E-08	3.13E-07
ENSMUSG00000070034	164.4153317	2.407418107	0.377258118	6.381355339	1.76E-10	3.99E-09
ENSMUSG00000079808	135.7663298	2.409924449	0.366638774	6.573021234	4.93E-11	1.22E-09
ENSMUSG00000017009	4274.326936	2.410203631	0.25115382	9.596523879	8.27E-22	6.40E-20
ENSMUSG00000035164	955.0171679	2.413120893	0.256223053	9.418047548	4.60E-21	3.35E-19
ENSMUSG00000030921	403.2254924	2.41405711	0.337372495	7.155465094	8.34E-13	2.51E-11
ENSMUSG00000024897	97.48398303	2.41665201	0.40174946	6.015321122	1.80E-09	3.51E-08
ENSMUSG00000027580	1457.277192	2.416838039	0.248983339	9.706826383	2.82E-22	2.31E-20
ENSMUSG00000092021	5.477140026	2.422098893	0.875437925	2.766728312	0.005662192	0.026110479
ENSMUSG00000020184	2024.8714	2.435823299	0.247372653	9.846776797	7.08E-23	6.02E-21
ENSMUSG00000053846	125.0823784	2.436574859	0.386627788	6.302120376	2.94E-10	6.42E-09
ENSMUSG00000026358	2287.704448	2.441494165	0.286745956	8.514485092	1.67E-17	8.99E-16
ENSMUSG00000073489	4102.274543	2.447150687	0.6569849	3.724820292	0.000195454	0.001469393
ENSMUSG00000041135	315.4306939	2.447467347	0.314650337	7.778371927	7.35E-15	2.91E-13
ENSMUSG00000025492	236.1968255	2.449645882	0.381746901	6.416937173	1.39E-10	3.22E-09
ENSMUSG00000050533	125.7932995	2.450205245	0.374052852	6.550425251	5.74E-11	1.41E-09
ENSMUSG00000039997	1077.147234	2.451316718	0.353041922	6.9434154	3.83E-12	1.07E-10
ENSMUSG00000050914	101.29436	2.455381854	0.4143695	5.925584309	3.11E-09	5.90E-08

	91		75			
ENSMUSG00000086798	8.1229059 89	2.45894882	0.8650297 12	2.842617756	0.004474 47	0.02148 6548
ENSMUSG00000030144	1621.9026 95	2.459112074	0.2915249 25	8.435340744	3.30E-17	1.71E-15
ENSMUSG00000083899	926.73303 13	2.464768285	0.2449043 81	10.0642066	7.95E-24	7.47E-22
ENSMUSG00000073405	8.4526558 28	2.466590462	0.8428779 92	2.926390874	0.003429 198	0.01722 1384
ENSMUSG00000023030	1935.2259 06	2.466806205	0.2289076 26	10.77642648	4.45E-27	5.49E-25
ENSMUSG00000031844	10.067227 25	2.466877781	0.8149829 07	3.026907386	0.002470 696	0.01299 2466
ENSMUSG00000078921	17.362193 63	2.46739187	0.7224055 92	3.415521553	0.000636 6	0.00409 2215
ENSMUSG00000024395	52.777019 52	2.46899773	0.5214255 43	4.735091641	2.19E-06	2.55E-05
ENSMUSG00000079419	752.03852 17	2.476169875	0.2781734 63	8.901531624	5.51E-19	3.28E-17
ENSMUSG00000042807	19.438773 28	2.476389309	0.7155133 08	3.460996854	0.000538 179	0.00353 5832
ENSMUSG00000001627	2849.4351 51	2.479412932	0.2554737 55	9.705157136	2.87E-22	2.34E-20
ENSMUSG00000028538	203.92819 44	2.488803369	0.3690872 81	6.7431296	1.55E-11	4.10E-10
ENSMUSG00000050711	9.9526141 65	2.491426161	0.8159045 59	3.053575488	0.002261 318	0.01202 1476
ENSMUSG00000042349	601.63521 93	2.493201315	0.2582926 61	9.652621603	4.79E-22	3.75E-20
ENSMUSG00000097754	132.61500 4	2.495581772	0.4135219 17	6.03494439	1.59E-09	3.14E-08
ENSMUSG00000026489	80.835445 12	2.495945012	0.4519787 65	5.522261671	3.35E-08	5.34E-07
ENSMUSG00000021453	246.18078 78	2.49602437	0.3111685 58	8.021454305	1.05E-15	4.59E-14
ENSMUSG00000072109	5.7114562 33	2.497031198	0.8745219 47	2.855309929	0.004299 485	0.02075 5351
ENSMUSG00000063286	1685.1097 52	2.498353894	0.3301630 26	7.56703113	3.82E-14	1.37E-12
ENSMUSG00000061802	14.706721 68	2.498625773	0.7539731	3.313945517	0.000919 894	0.00561 816
ENSMUSG00000048852	19.695145 4	2.498860682	0.7186308 84	3.477252006	0.000506 581	0.00335 5989
ENSMUSG00000038437	594.92247 3	2.503096708	0.2827733 34	8.851954579	8.60E-19	4.99E-17
ENSMUSG00000084817	28.154432 93	2.504070693	0.6313700 21	3.966090582	7.31E-05	0.00061 3919
ENSMUSG00000100392	5.5052583 93	2.504822182	0.8741164 88	2.865547345	0.004162 89	0.02018 7653
ENSMUSG00000053820	7.1774390 89	2.514349909	0.8566528 66	2.935086088	0.003334 552	0.01686 9705
ENSMUSG00000082088	269.99870 48	2.522461702	0.4136351 89	6.098276372	1.07E-09	2.17E-08
ENSMUSG00000055413	18.388298	2.522713238	0.7230666	3.488908287	0.000484	0.00322

	14		53		998	8213
ENSMUSG00000038094	356.37263<br>32	2.525056935	0.2975275<br>06	8.486801659	2.12E-17	1.12E-15
ENSMUSG00000021831	2148.4989<br>49	2.531720236	0.2298378<br>06	11.01524712	3.23E-28	4.56E-26
ENSMUSG00000032691	313.09256<br>74	2.533343142	0.3002297<br>01	8.438016399	3.23E-17	1.68E-15
ENSMUSG00000004837	741.83214<br>43	2.538520982	0.2711650<br>01	9.361536219	7.86E-21	5.55E-19
ENSMUSG00000037129	5.6915399<br>36	2.544738194	0.8734789<br>97	2.913336444	0.003575<br>891	0.01780<br>7989
ENSMUSG00000003206	33.092076<br>91	2.546450908	0.5886356<br>53	4.326022212	1.52E-05	0.00014<br>7111
ENSMUSG00000040264	5.6774807<br>53	2.547119237	0.8734426<br>56	2.916183702	0.003543<br>418	0.01768<br>8095
ENSMUSG00000023947	131.95109<br>97	2.551126488	0.4072012<br>99	6.265025412	3.73E-10	8.03E-09
ENSMUSG00000021466	467.31617<br>75	2.552701703	0.2783881<br>55	9.169577299	4.75E-20	3.13E-18
ENSMUSG00000026896	1681.6418<br>21	2.554553088	0.3034623<br>21	8.418023969	3.83E-17	1.95E-15
ENSMUSG00000106227	5.8356439<br>29	2.558980095	0.8734135<br>66	2.929860713	0.003391<br>14	0.01708<br>0327
ENSMUSG00000035448	7.4258144<br>8	2.561449116	0.8544062<br>85	2.997928693	0.002718<br>213	0.01412<br>1723
ENSMUSG00000079505	5.8778214<br>79	2.562064156	0.8732983<br>76	2.93377868	0.003348<br>629	0.01691<br>1672
ENSMUSG00000027995	2525.7072<br>59	2.562772588	0.2507245<br>91	10.22146483	1.59E-24	1.69E-22
ENSMUSG00000095580	15.410174<br>62	2.564053653	0.8239170<br>42	3.112028907	0.001858<br>063	0.01023<br>503
ENSMUSG00000042129	3685.3825<br>9	2.564740298	0.2344995<br>14	10.93708152	7.66E-28	1.01E-25
ENSMUSG00000067212	2458.7300<br>63	2.568837309	0.2648459<br>25	9.699365045	3.03E-22	2.46E-20
ENSMUSG00000038065	113.36423<br>2	2.569910273	0.4315956<br>53	5.954439662	2.61E-09	5.00E-08
ENSMUSG00000021270	5418.7564<br>55	2.570324852	0.2256332<br>62	11.39160436	4.60E-30	7.17E-28
ENSMUSG00000037405	1130.4723<br>71	2.57489103	0.2532910<br>3	10.1657411	2.82E-24	2.91E-22
ENSMUSG00000032021	5.8016684<br>48	2.577522109	0.8728785<br>44	2.952898921	0.003148<br>05	0.01599<br>7044
ENSMUSG00000020334	182.18166	2.580010193	0.3374872<br>75	7.644762877	2.09E-14	7.84E-13
ENSMUSG00000000732	164.36733<br>77	2.581786895	0.3453525<br>38	7.475801138	7.67E-14	2.66E-12
ENSMUSG00000027639	4699.9640<br>01	2.583311284	0.2771367<br>89	9.321430373	1.15E-20	7.90E-19
ENSMUSG00000003208	177.00524<br>55	2.588661255	0.3408299<br>86	7.595168748	3.07E-14	1.13E-12
ENSMUSG00000061852	14.067875<br>14	2.589519566	0.7681886<br>29	3.370942329	0.000749<br>115	0.00471<br>3786
ENSMUSG00000031955	9.3418859	2.589931929	0.8379031	3.090968044	0.001995	0.01083

	94		72		051	7168
ENSMUSG00000038151	70.72292675	2.596317755	0.45225417	5.740837619	9.42E-09	1.65E-07
ENSMUSG00000035274	6.228123312	2.60264919	0.872921803	2.981537614	0.002868048	0.014782474
ENSMUSG00000028780	14.23561293	2.607533096	0.773052017	3.37303705	0.000743439	0.004688835
ENSMUSG00000030747	641.9583504	2.621803344	0.34324209	7.638350375	2.20E-14	8.22E-13
ENSMUSG00000046879	3837.586777	2.622705209	0.296791884	8.836849499	9.85E-19	5.69E-17
ENSMUSG00000091649	677.9421804	2.623141511	0.329125277	7.970039659	1.59E-15	6.84E-14
ENSMUSG00000018648	65.78531301	2.627799461	0.466007256	5.638966837	1.71E-08	2.86E-07
ENSMUSG00000024679	4807.086784	2.633491351	0.280348846	9.393622941	5.80E-21	4.16E-19
ENSMUSG00000039384	49.11492149	2.636681255	0.51958476	5.074593129	3.88E-07	5.19E-06
ENSMUSG00000041886	6.29021716	2.636788382	0.872142241	3.023346718	0.002499956	0.013112094
ENSMUSG00000107215	38.54742378	2.638607488	0.578494816	4.561160122	5.09E-06	5.46E-05
ENSMUSG00000100398	81.11555065	2.641436111	0.448582786	5.888402754	3.90E-09	7.29E-08
ENSMUSG00000104654	6.084019319	2.645041552	0.871541902	3.03489889	0.002406163	0.012712623
ENSMUSG00000025408	3225.100135	2.648391306	0.255058956	10.38344761	2.95E-25	3.21E-23
ENSMUSG00000029298	176.7168004	2.653279245	0.349029494	7.601876895	2.92E-14	1.08E-12
ENSMUSG00000051726	6.222266199	2.654434495	0.871427449	3.046076296	0.00231849	0.012292033
ENSMUSG00000024672	1253.898262	2.667170091	0.346773618	7.691386985	1.46E-14	5.57E-13
ENSMUSG00000022346	15.03774291	2.669133988	0.757738408	3.522500591	0.000427496	0.002888971
ENSMUSG00000011008	3634.036835	2.670958651	0.248055109	10.76760185	4.90E-27	5.99E-25
ENSMUSG00000063450	84.76603407	2.675236239	0.456301408	5.862870883	4.55E-09	8.36E-08
ENSMUSG00000015837	24316.15746	2.684990855	0.217750755	12.33056964	6.20E-35	1.18E-32
ENSMUSG00000069873	65.10851073	2.692390984	0.496660349	5.420990396	5.93E-08	9.17E-07
ENSMUSG00000025481	6.318335527	2.69944091	0.870295159	3.101753334	0.001923782	0.010537742
ENSMUSG00000070883	80.86439243	2.708366311	0.491963891	5.505213618	3.69E-08	5.84E-07
ENSMUSG00000032204	493.9044331	2.708735659	0.267501003	10.12607663	4.23E-24	4.23E-22
ENSMUSG00000025757	1234.088175	2.709956322	0.261844252	10.3494971	4.21E-25	4.54E-23
ENSMUSG00000028793	1549.3559	2.711359277	0.2318351	11.69520359	1.35E-31	2.23E-29

	74		5			
ENSMUSG00000087203	12.023767 05	2.718143016	0.7982363 06	3.405185902	0.000661 19	0.00422 6498
ENSMUSG00000026483	92.620979 11	2.718982837	0.4178290 8	6.507404509	7.65E-11	1.84E-09
ENSMUSG00000032000	458.41550 62	2.719676224	0.2711538 35	10.03001203	1.13E-23	1.05E-21
ENSMUSG00000019122	1135.6369 66	2.720533171	0.2756418 19	9.86981288	5.63E-23	4.82E-21
ENSMUSG00000044703	154.71078 79	2.724604901	0.3772554 41	7.222175225	5.12E-13	1.59E-11
ENSMUSG00000032997	167.07624 09	2.725580958	0.4338234 2	6.282696676	3.33E-10	7.22E-09
ENSMUSG00000021403	374.39239 83	2.728388962	0.3587573 44	7.605109704	2.85E-14	1.05E-12
ENSMUSG00000063354	33.861135 85	2.732749055	0.5930295 39	4.608116251	4.06E-06	4.46E-05
ENSMUSG00000021759	558.00885 61	2.739038352	0.2748755 9	9.964647473	2.18E-23	2.00E-21
ENSMUSG00000056116	1046.6946 93	2.742626731	0.2483965 77	11.04132257	2.41E-28	3.48E-26
ENSMUSG00000084850	18.545357 51	2.744327361	0.7139629 64	3.843795128	0.000121 146	0.00096 0826
ENSMUSG00000026725	22.304459 54	2.745366214	0.6809267 07	4.031808687	5.53E-05	0.00047 9192
ENSMUSG00000020227	62.984165 16	2.749322514	0.4851232 98	5.667265466	1.45E-08	2.46E-07
ENSMUSG00000018906	13.581334 66	2.755948417	0.7733382 35	3.563703812	0.000365 659	0.00251 9533
ENSMUSG00000042622	394.88161 04	2.762776457	0.3114403 86	8.870964002	7.25E-19	4.23E-17
ENSMUSG00000078606	34.307170 29	2.773868947	0.6309249 07	4.396512033	1.10E-05	0.00011 0125
ENSMUSG00000087263	18.355994 17	2.779625091	0.7380297 7	3.766277736	0.000165 699	0.00127 2219
ENSMUSG00000044350	755.20415 72	2.783882146	0.3163382 24	8.800334359	1.36E-18	7.76E-17
ENSMUSG00000005686	455.01469 37	2.791464667	0.2807514 34	9.942833171	2.71E-23	2.44E-21
ENSMUSG00000029379	6.9310719 34	2.793174664	0.8683330 99	3.216708735	0.001296 702	0.00758 4847
ENSMUSG00000023913	16.635778 81	2.803343114	0.7507321 41	3.73414559	0.000188 354	0.00142 3035
ENSMUSG00000038037	13.203550 16	2.813715085	0.7951222 24	3.538720213	0.000402 072	0.00274 5587
ENSMUSG00000037321	2439.7269 54	2.821586737	0.2349383 34	12.00990359	3.15E-33	5.68E-31
ENSMUSG00000051439	3593.7493 14	2.831351856	0.2292284 67	12.35165901	4.77E-35	9.34E-33
ENSMUSG00000106734	396.96200 13	2.833000294	0.3338423 81	8.48604149	2.14E-17	1.12E-15
ENSMUSG00000047281	40.514042 51	2.846362926	0.5749635 32	4.950510365	7.40E-07	9.28E-06
ENSMUSG00000107179	7.1653881	2.849572991	0.8666829	3.287906986	0.001009	0.00610

	41		7		352	7358
ENSMUSG00000038587	83.70557668	2.852170231	0.467166811	6.105250131	1.03E-09	2.08E-08
ENSMUSG00000063234	268.0720318	2.85247186	0.339455511	8.403080133	4.35E-17	2.18E-15
ENSMUSG00000040483	766.3431168	2.853498913	0.309419882	9.222092954	2.91E-20	1.93E-18
ENSMUSG00000079491	28.64180693	2.856643149	0.652572429	4.377511252	1.20E-05	0.000119736
ENSMUSG00000067889	132.3692423	2.867081951	0.384463793	7.457352297	8.83E-14	3.04E-12
ENSMUSG00000024805	159.3773256	2.871048338	0.385780597	7.442179212	9.90E-14	3.38E-12
ENSMUSG00000028645	1287.190264	2.880253131	0.271619634	10.60399458	2.86E-26	3.37E-24
ENSMUSG00000035208	733.20283	2.881137166	0.316971293	9.089583911	9.94E-20	6.43E-18
ENSMUSG00000013584	7.447739012	2.884645134	0.8658941	3.331406386	0.000864084	0.00532476
ENSMUSG00000037580	1056.259206	2.893884266	0.262534815	11.02285907	2.96E-28	4.23E-26
ENSMUSG00000069874	801.4731003	2.896183022	0.304547856	9.509779721	1.91E-21	1.43E-19
ENSMUSG00000030748	327.2740468	2.898033236	0.326343485	8.880315893	6.67E-19	3.92E-17
ENSMUSG00000057596	29.65562491	2.903629359	0.663717255	4.374798662	1.22E-05	0.000120971
ENSMUSG00000027827	64.42299455	2.910125705	0.493569094	5.896085752	3.72E-09	6.99E-08
ENSMUSG00000054822	9.506541978	2.9215478	0.836492535	3.492616705	0.000478313	0.003191467
ENSMUSG00000053113	159.1970793	2.922726997	0.379814599	7.695141269	1.41E-14	5.44E-13
ENSMUSG00000085363	7.461798196	2.92369289	0.864260805	3.382882661	0.000717293	0.00454207
ENSMUSG00000029605	190.1569015	2.937442962	0.354462674	8.287030427	1.16E-16	5.54E-15
ENSMUSG00000029380	7.523892044	2.942276717	0.863577175	3.407080228	0.000656618	0.004199231
ENSMUSG00000068245	2832.724671	2.946612405	0.315537429	9.338392645	9.78E-21	6.84E-19
ENSMUSG00000036528	523.4317421	2.954027417	0.273884419	10.78567165	4.02E-27	5.06E-25
ENSMUSG00000039209	56.35987367	2.956296278	0.510188502	5.794517643	6.85E-09	1.22E-07
ENSMUSG00000023067	472.405513	2.959573085	0.296976955	9.965665797	2.15E-23	1.99E-21
ENSMUSG00000000982	4533.434186	2.962093098	0.30381565	9.749639628	1.85E-22	1.53E-20
ENSMUSG00000001156	411.6938122	2.963984751	0.280321563	10.57351678	3.95E-26	4.59E-24
ENSMUSG00000063851	50.28559655	2.964008452	0.524304549	5.653219029	1.57E-08	2.65E-07
ENSMUSG00000047735	2474.0536	2.976771167	0.3279308	9.077435592	1.11E-19	7.12E-18

	58		5			
ENSMUSG00000103219	7.916371427	2.991442744	0.862317921	3.469071756	0.00052226	0.003444453
ENSMUSG00000027646	648.4414157	2.994603505	0.342524105	8.742752583	2.27E-18	1.28E-16
ENSMUSG00000030717	1236.337364	3.003552688	0.240227191	12.5029672	7.19E-36	1.47E-33
ENSMUSG00000026981	1739.474723	3.003918203	0.306375919	9.804681177	1.07E-22	9.04E-21
ENSMUSG00000054072	106.6262711	3.005579436	0.833102476	3.60769476	0.00030893	0.002179043
ENSMUSG00000025591	533.0331002	3.006388527	0.294571758	10.20596323	1.86E-24	1.97E-22
ENSMUSG00000037995	11.87718672	3.012559489	0.813988301	3.700986227	0.000214763	0.001590129
ENSMUSG00000035356	588.6907258	3.016721058	0.287200287	10.5038929	8.29E-26	9.39E-24
ENSMUSG00000030107	4236.145633	3.018161001	0.234147668	12.8899896	5.13E-38	1.17E-35
ENSMUSG00000070427	22.91749492	3.024939676	0.693763836	4.360186448	1.30E-05	0.000128036
ENSMUSG00000078349	51.50447541	3.032256573	0.532140648	5.698223924	1.21E-08	2.07E-07
ENSMUSG00000037553	514.1637713	3.034015486	0.275527091	11.01167757	3.36E-28	4.69E-26
ENSMUSG00000055994	167.3982159	3.037077261	0.391001528	7.767430674	8.01E-15	3.15E-13
ENSMUSG00000040328	12.82479324	3.037487885	0.803551424	3.780079029	0.000156779	0.001210715
ENSMUSG00000047415	115.0266569	3.051842411	0.418760599	7.287797425	3.15E-13	1.00E-11
ENSMUSG00000070031	393.0640771	3.057636438	0.329547839	9.278277924	1.72E-20	1.16E-18
ENSMUSG00000045502	1089.840845	3.108333709	0.262802333	11.82764884	2.81E-32	4.81E-30
ENSMUSG00000025498	3888.314422	3.118554224	0.258801333	12.04999291	1.94E-33	3.54E-31
ENSMUSG00000052270	8.475216057	3.119850101	0.857240297	3.639411388	0.000273262	0.001955642
ENSMUSG00000022441	103.522974	3.126710361	0.422644644	7.397965181	1.38E-13	4.66E-12
ENSMUSG00000023206	63.19350373	3.126881365	0.49696781	6.291919315	3.14E-10	6.84E-09
ENSMUSG00000039329	8.599403752	3.130965592	0.856941404	3.653651903	0.000258537	0.001862905
ENSMUSG00000032374	520.6409495	3.131724502	0.292899107	10.69216131	1.11E-26	1.34E-24
ENSMUSG00000032802	9844.889763	3.140575779	0.252007232	12.46224467	1.20E-35	2.42E-33
ENSMUSG00000078616	15.63595203	3.158611093	0.777557578	4.062221476	4.86E-05	0.000426486
ENSMUSG00000039304	93.17482427	3.167379006	0.513872796	6.163741364	7.10E-10	1.48E-08
ENSMUSG00000055523	13.020649	3.168516071	0.8050315	3.935890416	8.29E-05	0.00068

	39		78			5985
ENSMUSG00000067203	83.20193741	3.173305293	0.472079766	6.721968451	1.79E-11	4.68E-10
ENSMUSG00000001119	8.805601592	3.178799622	0.855039368	3.717723115	0.000201026	0.00150665
ENSMUSG00000062232	915.5237073	3.193516964	0.256657737	12.44270677	1.53E-35	3.04E-33
ENSMUSG00000037921	510.0245414	3.201801659	0.314474304	10.18144128	2.40E-24	2.51E-22
ENSMUSG00000092277	11.53923481	3.205313651	0.82062983	3.905919009	9.39E-05	0.000764385
ENSMUSG00000026102	89.16926145	3.211294799	0.456246303	7.038511386	1.94E-12	5.63E-11
ENSMUSG00000043943	9.246115639	3.216982195	0.854191958	3.766111547	0.00016581	0.001272353
ENSMUSG00000035498	33.07493439	3.222266485	0.623650949	5.166778772	2.38E-07	3.35E-06
ENSMUSG00000024424	57.01385142	3.228616823	0.522912456	6.174297023	6.65E-10	1.40E-08
ENSMUSG00000024190	206.0986736	3.22975481	0.344230992	9.382521876	6.44E-21	4.57E-19
ENSMUSG00000041936	82.04196945	3.230770323	0.474879019	6.803354529	1.02E-11	2.74E-10
ENSMUSG00000060183	9.418337999	3.240919244	0.853312477	3.798045065	0.000145842	0.001136952
ENSMUSG00000078920	649.4055052	3.242081059	0.341216604	9.501533679	2.07E-21	1.53E-19
ENSMUSG00000097077	27.76954862	3.244568218	0.656002146	4.945971957	7.58E-07	9.48E-06
ENSMUSG00000079457	35.1151936	3.24495778	0.606909754	5.34668912	8.96E-08	1.35E-06
ENSMUSG00000024014	574.9334103	3.250595502	0.285773327	11.37473374	5.59E-30	8.60E-28
ENSMUSG00000073555	23.08415915	3.256297921	0.721684098	4.512082132	6.42E-06	6.76E-05
ENSMUSG00000030494	9.350387038	3.25930698	0.851875396	3.826037228	0.000130223	0.001024513
ENSMUSG00000018899	1266.244959	3.266653206	0.258436756	12.6400488	1.27E-36	2.68E-34
ENSMUSG00000057346	135.3202681	3.27423866	0.843699209	3.880812766	0.000104108	0.000838801
ENSMUSG00000031712	674.9987614	3.275235222	0.325438029	10.06408266	7.96E-24	7.47E-22
ENSMUSG00000018930	3036.712488	3.277236182	0.239578796	13.67915791	1.35E-42	3.56E-40
ENSMUSG00000028602	82.45265587	3.296606405	0.460033243	7.166017791	7.72E-13	2.33E-11
ENSMUSG00000003863	53.87170761	3.297320899	0.592891484	5.561423952	2.68E-08	4.37E-07
ENSMUSG00000029392	110.3034113	3.298175452	0.414605307	7.954976451	1.79E-15	7.65E-14
ENSMUSG00000045827	1667.636247	3.307575151	0.305601862	10.82315118	2.67E-27	3.39E-25
ENSMUSG00000034422	4728.2213	3.310017823	0.2572095	12.86895503	6.73E-38	1.49E-35

	83		26			
ENSMUSG00000026980	31.598609 85	3.314270649	0.6327134 78	5.238185632	1.62E-07	2.34E-06
ENSMUSG00000072620	6906.7157 89	3.321837177	0.2361823 2	14.06471565	6.26E-45	1.86E-42
ENSMUSG00000024789	2112.0317 79	3.340094187	0.2538480 72	13.15784736	1.53E-39	3.69E-37
ENSMUSG00000013846	538.21062 28	3.340574561	0.2748171 66	12.15562553	5.36E-34	9.92E-32
ENSMUSG00000032515	295.84532 64	3.340672135	0.3153295 61	10.5942244	3.17E-26	3.71E-24
ENSMUSG00000066363	33.061174 18	3.351925959	0.6690608 63	5.009896924	5.45E-07	7.05E-06
ENSMUSG00000053318	214.15583 46	3.368780933	0.3382262 32	9.960140921	2.28E-23	2.08E-21
ENSMUSG00000040329	24.345952 4	3.374638941	0.7010133 47	4.813943919	1.48E-06	1.77E-05
ENSMUSG00000097028	15.870903 93	3.380745059	0.7766117 04	4.3531987	1.34E-05	0.00013 1809
ENSMUSG00000044103	10.513766	3.394352083	0.8472138 17	4.006488108	6.16E-05	0.00052 6568
ENSMUSG00000078853	1490.0028 18	3.406377931	0.2944304 86	11.56937918	5.89E-31	9.28E-29
ENSMUSG00000043263	1534.3933 65	3.41253808	0.7716815 51	4.422210271	9.77E-06	9.87E-05
ENSMUSG00000075010	10.575859 85	3.413306303	0.8461968 89	4.033702258	5.49E-05	0.00047 5946
ENSMUSG00000026536	621.26345 09	3.44253435	0.3560825 98	9.667797224	4.13E-22	3.25E-20
ENSMUSG00000029798	1575.1231 34	3.449381139	0.3488598 74	9.887583504	4.71E-23	4.06E-21
ENSMUSG00000049608	18.427830 22	3.454803268	0.7540135 75	4.581884705	4.61E-06	4.98E-05
ENSMUSG00000021701	7657.9400 63	3.457420201	0.2440861 13	14.16475585	1.51E-45	4.72E-43
ENSMUSG00000039699	18.413771 04	3.45807399	0.7535491 64	4.589048936	4.45E-06	4.83E-05
ENSMUSG00000067297	83.917497 18	3.468579005	0.4735152 9	7.325167906	2.39E-13	7.79E-12
ENSMUSG00000043421	632.41482 51	3.474164704	0.3338004 33	10.40790952	2.28E-25	2.50E-23
ENSMUSG00000044162	300.79004 91	3.475522035	0.3444203 98	10.09092974	6.06E-24	5.93E-22
ENSMUSG00000079584	41.569431 05	3.475637679	0.6026656 76	5.7671074	8.06E-09	1.43E-07
ENSMUSG00000054404	894.54807 69	3.476758255	0.2908873 61	11.9522493	6.32E-33	1.11E-30
ENSMUSG00000040584	182.16818 51	3.486623933	0.3737525 16	9.328696898	1.07E-20	7.42E-19
ENSMUSG00000033538	1637.3876 88	3.500943747	0.3148480 99	11.11946922	1.01E-28	1.47E-26
ENSMUSG00000060509	86.146476 52	3.502667002	0.5085511 3	6.88754148	5.68E-12	1.55E-10
ENSMUSG00000073530	51.430460	3.524919931	0.5583514	6.313084653	2.74E-10	6.03E-09

	46		44			
ENSMUSG00000024737	6310.669785	3.533614868	0.24177891	14.61506657	2.25E-48	7.35E-46
ENSMUSG00000039960	173.535985	3.541217111	0.394684006	8.972284312	2.90E-19	1.81E-17
ENSMUSG00000051682	45.52780024	3.541850375	0.595049314	5.952196384	2.65E-09	5.05E-08
ENSMUSG00000086866	12.39415278	3.547003945	0.842528639	4.209950595	2.55E-05	0.000235573
ENSMUSG00000074862	14.77748416	3.571266673	0.799019625	4.469560647	7.84E-06	8.09E-05
ENSMUSG00000027737	6811.607427	3.579144762	0.247341502	14.4704578	1.86E-47	5.94E-45
ENSMUSG00000026765	18.21406601	3.581852636	0.76275644	4.695932346	2.65E-06	3.02E-05
ENSMUSG00000035352	314.4127247	3.591637339	0.344991216	10.41080808	2.21E-25	2.45E-23
ENSMUSG00000031722	227.1514052	3.593315888	0.361176846	9.948909861	2.55E-23	2.31E-21
ENSMUSG00000028527	787.6674207	3.599632773	0.262299643	13.72336129	7.36E-43	1.98E-40
ENSMUSG00000024074	406.4590681	3.606540924	0.318279899	11.33134997	9.18E-30	1.38E-27
ENSMUSG00000024401	1380.261937	3.62276367	0.302820382	11.96340762	5.52E-33	9.83E-31
ENSMUSG00000020651	12.33440389	3.629955425	0.836458489	4.339671932	1.43E-05	0.000139291
ENSMUSG00000089844	24.047066	3.630174674	0.714033522	5.084039562	3.69E-07	4.95E-06
ENSMUSG00000019850	1119.094893	3.649761672	0.265791973	13.73164748	6.56E-43	1.80E-40
ENSMUSG00000028525	208.5290596	3.661052171	0.3505119	10.44487268	1.55E-25	1.74E-23
ENSMUSG00000041827	4923.829404	3.661831905	0.228219872	16.04519305	6.18E-58	2.57E-55
ENSMUSG00000009376	214.8840855	3.684930633	0.358725242	10.27229257	9.39E-25	1.01E-22
ENSMUSG00000018925	12.40235485	3.685504825	0.832965895	4.424556692	9.66E-06	9.77E-05
ENSMUSG00000034266	24.91637987	3.696942878	0.707019356	5.228913246	1.71E-07	2.45E-06
ENSMUSG00000037411	82.13388188	3.699903306	0.49489699	7.476107919	7.66E-14	2.66E-12
ENSMUSG00000079138	48.07233886	3.73168997	0.608239137	6.13523488	8.50E-10	1.75E-08
ENSMUSG00000089929	348.5859514	3.756072441	0.344265508	10.91039431	1.03E-27	1.33E-25
ENSMUSG00000006403	119.6267807	3.771191068	0.809589953	4.65814954	3.19E-06	3.59E-05
ENSMUSG00000023341	458.5420734	3.773182412	0.332157253	11.35962675	6.64E-30	1.01E-27
ENSMUSG00000038260	73.96977674	3.80276254	0.555350217	6.847503476	7.51E-12	2.04E-10
ENSMUSG00000006818	7763.0855	3.809686853	0.2286689	16.66027133	2.55E-62	1.29E-59

	6		56			
ENSMUSG00000020838	13.5598767	3.816757739	0.82669189	4.616904782	3.90E-06	4.29E-05
ENSMUSG00000022144	13.5598767	3.816757739	0.82669189	4.616904782	3.90E-06	4.29E-05
ENSMUSG00000043410	13.87620305	3.819468071	0.827520589	4.615556546	3.92E-06	4.32E-05
ENSMUSG00000041481	253.1977111	3.854883094	0.394892305	9.761859231	1.64E-22	1.36E-20
ENSMUSG00000028599	5845.209695	3.881346174	0.222765514	17.42346068	5.48E-68	3.13E-65
ENSMUSG00000079339	1647.533864	3.904030811	0.748250389	5.217545979	1.81E-07	2.59E-06
ENSMUSG00000045664	58.71233854	3.9248868	0.547610662	7.167294349	7.65E-13	2.31E-11
ENSMUSG00000062488	310.7383266	3.935652096	0.402954763	9.766982449	1.56E-22	1.30E-20
ENSMUSG00000078566	915.8405679	3.939279226	0.259363355	15.18826446	4.23E-52	1.61E-49
ENSMUSG00000028037	347.117074	3.948282806	0.739550198	5.338762423	9.36E-08	1.41E-06
ENSMUSG00000001281	722.3892481	3.950280259	0.361907676	10.91516019	9.76E-28	1.27E-25
ENSMUSG00000024772	2929.809057	3.981227066	0.244081284	16.31107064	8.23E-60	3.76E-57
ENSMUSG00000016283	28.49150757	3.999951884	0.702376996	5.694878831	1.23E-08	2.11E-07
ENSMUSG00000040253	2169.485102	4.005313778	0.301565327	13.28174504	2.95E-40	7.36E-38
ENSMUSG00000027514	73.02366663	4.019319929	0.519514335	7.736687251	1.02E-14	3.95E-13
ENSMUSG00000035385	4530.728332	4.025902788	0.258426426	15.57852597	1.02E-54	4.11E-52
ENSMUSG00000074896	1844.294814	4.047412383	0.349247815	11.58894117	4.69E-31	7.47E-29
ENSMUSG00000099974	291.6612673	4.057204184	0.345396961	11.74649648	7.36E-32	1.23E-29
ENSMUSG00000028965	45.67779756	4.087637671	0.609313815	6.708591811	1.97E-11	5.10E-10
ENSMUSG00000027962	28.2386476	4.115734376	0.713610372	5.767481156	8.05E-09	1.43E-07
ENSMUSG00000024675	280.1650149	4.135684005	0.427748879	9.668485904	4.10E-22	3.25E-20
ENSMUSG00000024743	38.66873195	4.138488243	0.656039036	6.308295721	2.82E-10	6.20E-09
ENSMUSG00000035929	645.9049207	4.149145227	0.281941389	14.71633957	5.06E-49	1.78E-46
ENSMUSG00000027360	154.7996123	4.152085733	0.411700979	10.08519763	6.42E-24	6.24E-22
ENSMUSG00000060550	24.97663308	4.159032501	0.751348067	5.535427166	3.10E-08	5.01E-07
ENSMUSG00000039232	676.0098354	4.16311523	0.276548023	15.05386003	3.26E-51	1.21E-48
ENSMUSG00000035107	725.46862	4.165239416	0.2717910	15.32515463	5.19E-53	2.03E-50

	33		2			
ENSMUSG00000073409	24.700139 32	4.183418872	0.7476871 92	5.595145825	2.20E-08	3.64E-07
ENSMUSG00000030156	111.54404 25	4.19105838	0.4615206 58	9.080976781	1.08E-19	6.92E-18
ENSMUSG00000039236	478.85898 61	4.267169183	0.3316191 26	12.86768115	6.84E-38	1.49E-35
ENSMUSG00000072594	217.50286 56	4.290326101	0.3920658 62	10.94287087	7.19E-28	9.57E-26
ENSMUSG00000031488	508.47811 84	4.297032667	0.3068906 35	14.00183706	1.52E-44	4.43E-42
ENSMUSG00000042638	19.141288 12	4.322629822	0.8012228 22	5.395040809	6.85E-08	1.05E-06
ENSMUSG00000003541	1798.5381 4	4.346635558	0.2603832 46	16.69322287	1.47E-62	7.74E-60
ENSMUSG00000081957	79.801534 04	4.354636039	0.5332208 82	8.166664476	3.17E-16	1.48E-14
ENSMUSG00000023868	19.782142 9	4.369811577	0.7988195 21	5.470336493	4.49E-08	7.07E-07
ENSMUSG00000004359	49.779166 94	4.371500657	0.6126161 96	7.135790214	9.62E-13	2.87E-11
ENSMUSG00000027456	20.270691 61	4.400615643	0.7975357 53	5.517765975	3.43E-08	5.46E-07
ENSMUSG00000001348	36.682233 14	4.444758072	0.7012562 79	6.338279179	2.32E-10	5.18E-09
ENSMUSG00000037849	86.473377 07	4.448003653	0.5469720 11	8.132049832	4.22E-16	1.95E-14
ENSMUSG00000003617	310.57173 48	4.45581082	0.3553708 08	12.53848297	4.60E-36	9.55E-34
ENSMUSG00000054203	505.80295 51	4.46465718	0.7815153 37	5.712820938	1.11E-08	1.91E-07
ENSMUSG00000030142	5665.1666 03	4.485220574	0.3059442 28	14.66025556	1.16E-48	3.87E-46
ENSMUSG00000034459	4123.1914 1	4.486924058	0.6128346 92	7.321589519	2.45E-13	7.96E-12
ENSMUSG00000017002	435.17700 98	4.511794099	0.3408539 38	13.23673749	5.38E-40	1.32E-37
ENSMUSG00000045932	11370.597 29	4.524607533	0.3053001 94	14.82019214	1.08E-49	3.91E-47
ENSMUSG00000026532	22.069068 24	4.524764881	0.7911244 31	5.71940987	1.07E-08	1.85E-07
ENSMUSG00000048388	22.503725 17	4.557284889	0.7891849 3	5.774672979	7.71E-09	1.37E-07
ENSMUSG00000009185	23.418728 75	4.576261439	0.7899142 79	5.79336462	6.90E-09	1.23E-07
ENSMUSG00000079499	281.32238 57	4.685079733	0.3635450 27	12.88720623	5.31E-38	1.19E-35
ENSMUSG00000035692	3837.2443 04	4.68964309	0.2446828 75	19.16620887	7.09E-82	6.07E-79
ENSMUSG00000021367	32.795225 92	4.733962379	0.7305702 67	6.479818016	9.18E-11	2.17E-09
ENSMUSG00000079363	26.498814 92	4.766779487	0.7796925 52	6.113665541	9.74E-10	1.98E-08
ENSMUSG00000082292	703.68352	4.790597016	0.3505735	13.66502765	1.64E-42	4.25E-40

	61		33			
ENSMUSG00000024164	2604.046097	4.898059596	0.2410026	20.32367947	7.94E-92	7.77E-89
ENSMUSG00000021025	1981.977347	4.910489912	0.260164398	18.87456528	1.85E-79	1.41E-76
ENSMUSG00000053338	200.9865075	4.920728404	0.416449893	11.81589546	3.23E-32	5.47E-30
ENSMUSG00000068246	156.0044289	4.930798135	0.452551891	10.89554199	1.21E-27	1.55E-25
ENSMUSG00000004296	32.68359348	4.951054585	0.77474154	6.390588774	1.65E-10	3.76E-09
ENSMUSG00000016496	3088.064852	4.952085072	0.272315324	18.18511349	6.77E-74	4.22E-71
ENSMUSG00000024778	178.9498731	4.956656226	0.437819065	11.32124346	1.03E-29	1.53E-27
ENSMUSG00000104713	68.74384177	4.96344487	0.64112019	7.741832105	9.80E-15	3.82E-13
ENSMUSG00000050824	57.99074348	5.017388906	0.641425107	7.822252129	5.19E-15	2.10E-13
ENSMUSG00000026604	626.6940536	5.107897318	0.30687968	16.64462543	3.31E-62	1.62E-59
ENSMUSG00000027219	101.330768	5.206692413	0.544563415	9.561223299	1.16E-21	8.91E-20
ENSMUSG00000090231	36.37407111	5.236829835	0.7549846	6.936339938	4.02E-12	1.13E-10
ENSMUSG00000027611	7304.513486	5.247996619	0.255058476	20.57566057	4.54E-94	5.18E-91
ENSMUSG00000097804	53.87601401	5.252734665	0.701262027	7.490402255	6.87E-14	2.39E-12
ENSMUSG00000007872	53.43731341	5.256703877	0.718406213	7.317174856	2.53E-13	8.19E-12
ENSMUSG00000020178	37.91821523	5.282305846	0.75328574	7.012353435	2.34E-12	6.70E-11
ENSMUSG00000000386	789.1988733	5.286115347	0.360102405	14.67947806	8.73E-49	2.99E-46
ENSMUSG00000020638	10290.36304	5.309743422	0.237493402	22.35743555	1.02E-110	2.00E-107
ENSMUSG00000044701	100.0745993	5.403641257	0.575244386	9.393644481	5.80E-21	4.16E-19
ENSMUSG00000035373	1100.301849	5.406164059	0.283365323	19.07842502	3.82E-81	3.08E-78
ENSMUSG00000028268	4489.158759	5.426720503	0.280157501	19.37024881	1.38E-83	1.26E-80
ENSMUSG00000026390	343.9360578	5.439662261	0.396013668	13.73604677	6.18E-43	1.73E-40
ENSMUSG00000043953	1565.667944	5.49900235	0.26893968	20.44697292	6.39E-93	6.74E-90
ENSMUSG00000102037	56.84895232	5.509433073	0.690604723	7.977693879	1.49E-15	6.45E-14
ENSMUSG00000009093	45.10937678	5.52478149	0.741800431	7.447800321	9.49E-14	3.24E-12
ENSMUSG00000027399	775.1810501	5.53269111	0.343751049	16.0950523	2.76E-58	1.22E-55
ENSMUSG00000035513	46.407490	5.554953262	0.7405769	7.500845935	6.34E-14	2.22E-12

	47		04			
ENSMUSG00000000204	1201.218539	5.580826083	0.347081067	16.07931581	3.56E-58	1.53E-55
ENSMUSG00000097418	102.6587652	5.595521111	0.594546734	9.411406697	4.90E-21	3.55E-19
ENSMUSG00000078763	130.5793772	5.607097385	0.555170889	10.09976837	5.54E-24	5.46E-22
ENSMUSG00000020641	38159.3902	5.664042547	0.27050838	20.93851045	2.39E-97	2.98E-94
ENSMUSG00000050395	67.44643773	5.681327356	0.674365471	8.424700848	3.62E-17	1.86E-15
ENSMUSG00000086196	95.87029331	5.728771963	0.607308709	9.433047607	3.98E-21	2.94E-19
ENSMUSG00000033213	183.1775305	5.760152453	0.523528758	11.00255213	3.71E-28	5.14E-26
ENSMUSG00000055301	74.63589002	5.823371704	0.683008846	8.526056049	1.51E-17	8.20E-16
ENSMUSG00000038179	1329.084092	6.019197812	0.327714318	18.36721034	2.40E-75	1.69E-72
ENSMUSG00000028270	5442.313622	6.105374969	0.281967759	21.65274144	5.73E-104	9.81E-101
ENSMUSG00000017652	3141.32453	6.14130602	0.270978473	22.66344607	1.03E-113	2.35E-110
ENSMUSG00000034855	2515.46526	6.358400208	0.301406956	21.09573149	8.70E-99	1.33E-95
ENSMUSG00000038239	89.93874765	6.463217733	0.699934156	9.234036766	2.61E-20	1.73E-18
ENSMUSG00000038067	329.4772072	6.693779271	0.509019979	13.1503272	1.69E-39	4.00E-37
ENSMUSG00000035042	966.8273657	6.791007096	0.371520462	18.27895845	1.22E-74	7.94E-72
ENSMUSG00000032487	5355.07442	6.871714439	0.270547253	25.39931329	2.57E-142	1.17E-138
ENSMUSG00000020010	283.9907344	7.000426656	0.534604437	13.0945914	3.54E-39	8.21E-37
ENSMUSG00000058427	1605.458927	7.60446219	0.362953513	20.95161481	1.81E-97	2.49E-94
ENSMUSG00000105504	831.173061	7.648310734	0.448363698	17.05827383	3.03E-65	1.66E-62
ENSMUSG00000055202	304.5914371	7.742650034	0.610207579	12.68855107	6.84E-37	1.47E-34
ENSMUSG00000027398	2158.568781	7.776485561	0.332008457	23.42255262	2.52E-121	8.63E-118
ENSMUSG00000022126	27597.67987	7.806775298	0.249419681	31.29975654	4.70E-215	3.22E-211
ENSMUSG00000025746	3629.502479	7.947421447	0.644389365	12.33325979	6.00E-35	1.16E-32
ENSMUSG00000026822	829.3653347	8.587684863	0.524286658	16.37975092	2.67E-60	1.26E-57
ENSMUSG00000040026	20282.47714	8.72974326	0.246413691	35.42718441	6.52E-275	8.93E-271
ENSMUSG00000020826	2940.45378	8.999226306	0.387632384	23.21587843	3.15E-119	8.63E-116
ENSMUSG00000026875	1405.0647	9.141379918	0.4977373	18.36586999	2.46E-75	1.69E-72

	5		75			

Supplementary Table S2. Differentially expressed genes of pair-wise comparison (LPS+AGE vs. LPS)

AGE+LPS vs. LPS						
ID	baseMean	log2FoldChange	lfcSE	stat	p-value	padj
ENSMUSG00000024675	292.1186807	-4.054262089	0.364187442	-11.13235005	8.73E-29	5.05E-25
ENSMUSG00000078763	143.4086599	-3.389507588	0.368336211	-9.202211154	3.51E-20	6.76E-17
ENSMUSG00000000386	884.1753946	-3.125237971	0.312938854	-9.986736818	1.74E-23	5.04E-20
ENSMUSG00000038179	1520.658853	-3.090753014	0.263124063	-11.74637159	7.37E-32	8.52E-28
ENSMUSG00000038239	100.3134772	-2.97006745	0.3907085	-7.601747717	2.92E-14	2.60E-11
ENSMUSG00000000204	1388.952378	-2.761856205	0.302252776	-9.137571003	6.39E-20	1.06E-16
ENSMUSG00000039200	93.06414599	-2.662554321	0.369080331	-7.214023881	5.43E-13	3.49E-10
ENSMUSG00000024677	265.7673853	-2.61929971	0.31650261	-8.275760214	1.28E-16	1.48E-13
ENSMUSG00000044703	159.2131103	-2.584771957	0.359307919	-7.193751712	6.30E-13	3.84E-10
ENSMUSG00000082292	812.2499639	-2.583666245	0.317395604	-8.140208021	3.95E-16	4.15E-13
ENSMUSG00000054203	588.8428428	-2.505496189	0.363160454	-6.899143789	5.23E-12	2.24E-09
ENSMUSG00000037849	99.32443054	-2.448850135	0.371658018	-6.588987763	4.43E-11	1.51E-08
ENSMUSG00000045932	13327.11599	-2.410170301	0.290538423	-8.295530328	1.08E-16	1.39E-13
ENSMUSG00000020826	3615.348046	-2.404904702	0.238806272	-10.07052569	7.46E-24	2.87E-20
ENSMUSG00000019813	172.0816907	-2.302079849	0.336549235	-6.840246854	7.91E-12	3.26E-09
ENSMUSG00000060509	95.77476261	-2.259301139	0.388849919	-5.810213726	6.24E-09	1.15E-06
ENSMUSG00000006403	143.225801	-2.177065035	0.349934173	-6.221355904	4.93E-10	1.36E-07
ENSMUSG00000091649	732.5662394	-2.176844541	0.310930554	-7.001063458	2.54E-12	1.17E-09
ENSMUSG00000020638	12734.12211	-2.161325119	0.241518342	-8.948906758	3.59E-19	5.19E-16
ENSMUSG00000069793	376.0609986	-2.158072671	0.323707495	-6.666736806	2.62E-11	9.16E-09
ENSMUSG00000056008	30.48450643	-2.146039119	0.435007404	-4.933339298	8.08E-07	7.56E-05
ENSMUSG00000040584	212.2941713	-2.106523354	0.31141473	-6.764366463	1.34E-11	4.99E-09
ENSMUSG00000067889	147.1600672	-2.103349324	0.325368253	-6.46451922	1.02E-10	3.18E-08
ENSMUSG00000058163	142.1454039	-2.099144023	0.373500551	-5.6201899	1.91E-08	3.20E-06

ENSMUSG00000054072	122.8665882	-2.099010147	0.405820791	-5.172258774	2.31E-07	2.62E-05
ENSMUSG00000054404	1048.12103	-2.080560037	0.279113402	-7.454174623	9.04E-14	7.47E-11
ENSMUSG00000039236	584.2477758	-2.049559695	0.280615432	-7.30380251	2.80E-13	1.90E-10
ENSMUSG00000105504	1057.464569	-2.003858306	0.293214455	-6.834104782	8.25E-12	3.29E-09
ENSMUSG00000020641	48324.28925	-1.993935432	0.26133728	-7.629739736	2.35E-14	2.27E-11
ENSMUSG00000078851	80.18899365	-1.990084341	0.370601906	-5.369870772	7.88E-08	1.01E-05
ENSMUSG00000078853	1760.59528	-1.96049692	0.292686531	-6.698282004	2.11E-11	7.62E-09
ENSMUSG00000050395	83.26932379	-1.947761154	0.364024286	-5.350635188	8.76E-08	1.10E-05
ENSMUSG00000079419	831.7478975	-1.939766015	0.276443202	-7.016870015	2.27E-12	1.09E-09
ENSMUSG00000068245	3245.108969	-1.937092609	0.305730293	-6.335952481	2.36E-10	6.82E-08
ENSMUSG00000028037	433.1613607	-1.932645055	0.315860776	-6.118661137	9.44E-10	2.32E-07
ENSMUSG00000031382	545.9355177	-1.932081848	0.271095987	-7.126928992	1.03E-12	5.65E-10
ENSMUSG00000028268	5704.105228	-1.930078323	0.272926749	-7.071781466	1.53E-12	7.69E-10
ENSMUSG00000092564	142.4180457	-1.915253926	0.342892988	-5.585573322	2.33E-08	3.74E-06
ENSMUSG00000078920	760.1799963	-1.897207085	0.325936209	-5.820792634	5.86E-09	1.10E-06
ENSMUSG00000038587	94.65037939	-1.871545005	0.377368604	-4.959461344	7.07E-07	6.81E-05
ENSMUSG00000044701	125.6881556	-1.865944206	0.350544613	-5.322986397	1.02E-07	1.26E-05
ENSMUSG00000036905	493.5270694	-1.863243187	0.288509688	-6.458165069	1.06E-10	3.22E-08
ENSMUSG00000045827	1974.787173	-1.854854668	0.315107522	-5.88641825	3.95E-09	7.73E-07
ENSMUSG00000022441	121.250693	-1.829932656	0.336933534	-5.431138399	5.60E-08	7.68E-06
ENSMUSG00000068246	198.9234129	-1.823840065	0.304622764	-5.987208715	2.13E-09	4.75E-07
ENSMUSG00000030157	250.3935215	-1.820128071	0.325756056	-5.587395964	2.30E-08	3.74E-06
ENSMUSG00000026536	752.7559669	-1.807772146	0.305478348	-5.917840524	3.26E-09	6.50E-07
ENSMUSG00000054976	60.09507822	-1.793590571	0.40587933	-4.419024174	9.91E-06	0.000647708
ENSMUSG00000028270	7127.036207	-1.780014405	0.273024533	-6.519613399	7.05E-11	2.26E-08
ENSMUSG00000079339	1810.820817	-1.774631715	0.442395915	-4.011410717	6.04E-05	0.002747669
ENSMUSG00000040428	84.37477433	-1.767783997	0.375013084	-4.713926191	2.43E-06	0.000194226
ENSMUSG00000015947	3087.7550	-1.764542247	0.2407185	-7.330313177	2.30E-13	1.66E-10

	32		35			
ENSMUSG00000008393	675.49879<br>58	-1.759410995	0.2591246<br>15	-6.789825788	1.12E-11	4.33E-09
ENSMUSG00000035208	863.84883<br>88	-1.757689467	0.2902383<br>16	-6.056021449	1.40E-09	3.29E-07
ENSMUSG00000070883	90.087206<br>88	-1.741089055	0.4034216<br>05	-4.315805188	1.59E-05	0.00092<br>8673
ENSMUSG00000047746	20.150481<br>79	-1.740834856	0.4417413<br>08	-3.940846884	8.12E-05	0.00351<br>6297
ENSMUSG00000070427	25.106583<br>18	-1.731588346	0.4387750<br>75	-3.946414566	7.93E-05	0.00348
ENSMUSG00000104713	86.252539<br>76	-1.728482618	0.3903123<br>29	-4.428460212	9.49E-06	0.00062<br>7987
ENSMUSG00000002602	29.609967<br>09	-1.728302905	0.4293067<br>29	-4.025799707	5.68E-05	0.00262<br>6281
ENSMUSG00000025492	266.26747<br>45	-1.710341494	0.3398775<br>94	-5.032227852	4.85E-07	4.92E-05
ENSMUSG00000003208	204.36193<br>33	-1.698954958	0.3043551<br>94	-5.582145432	2.38E-08	3.76E-06
ENSMUSG00000072844	65.900689<br>71	-1.692003089	0.3799506<br>49	-4.45321805	8.46E-06	0.00057<br>538
ENSMUSG00000034459	5344.9719<br>19	-1.690808148	0.3105741<br>36	-5.444137011	5.21E-08	7.52E-06
ENSMUSG00000073555	26.209199<br>12	-1.69047968	0.4383116<br>89	-3.856798077	0.000114<br>882	0.00462<br>8503
ENSMUSG00000070034	186.29993<br>65	-1.688087623	0.3277381<br>22	-5.150720986	2.59E-07	2.89E-05
ENSMUSG00000026548	178.11637<br>44	-1.679349485	0.3105218<br>43	-5.408152506	6.37E-08	8.46E-06
ENSMUSG00000026532	25.939141<br>92	-1.671409374	0.4389404<br>99	-3.807826747	0.000140<br>193	0.00530<br>9508
ENSMUSG00000029798	1959.8762<br>57	-1.667402491	0.2877732<br>25	-5.794154368	6.87E-09	1.24E-06
ENSMUSG00000007837	148.63561<br>54	-1.662475416	0.3375912<br>33	-4.924521892	8.46E-07	7.82E-05
ENSMUSG00000047067	139.24486<br>72	-1.659632681	0.3215601<br>57	-5.161188797	2.45E-07	2.75E-05
ENSMUSG00000037731	385.25265<br>85	-1.655740737	0.2776585<br>53	-5.963226127	2.47E-09	5.20E-07
ENSMUSG00000067212	2881.5556<br>96	-1.645042028	0.2503095<br>26	-6.572031262	4.96E-11	1.64E-08
ENSMUSG00000027962	33.737354<br>69	-1.63048373	0.4264542<br>23	-3.823349946	0.000131<br>651	0.00509<br>1228
ENSMUSG00000000881	41.654170<br>45	-1.62958483	0.4136939<br>29	-3.939107434	8.18E-05	0.00352<br>867
ENSMUSG00000078921	18.186375<br>56	-1.600458045	0.4424683<br>1	-3.61711338	0.000297<br>907	0.00946<br>3452
ENSMUSG00000026466	1324.4627<br>34	-1.599238428	0.2928389<br>01	-5.46115431	4.73E-08	7.01E-06
ENSMUSG00000027639	5515.7102<br>61	-1.596734365	0.2904433<br>47	-5.497575971	3.85E-08	5.94E-06
ENSMUSG00000074896	2369.4110<br>47	-1.595134573	0.3104259<br>07	-5.138535596	2.77E-07	2.99E-05
ENSMUSG00000010067	491.25361<br>01	-1.591563165	0.2715481<br>96	-5.861070667	4.60E-09	8.86E-07

ENSMUSG00000039304	111.2677166	-1.589820288	0.393509945	-4.040101929	5.34E-05	0.002523984
ENSMUSG00000050914	115.2388485	-1.581994574	0.349851463	-4.521903559	6.13E-06	0.000442906
ENSMUSG00000031712	841.52237	-1.576530859	0.296169175	-5.323075425	1.02E-07	1.26E-05
ENSMUSG00000016496	4136.47832	-1.573408604	0.254890506	-6.172880374	6.71E-10	1.72E-07
ENSMUSG00000073489	4881.710082	-1.57308011	0.335950896	-4.682470356	2.83E-06	0.000221446
ENSMUSG00000009185	28.6601057	-1.552659381	0.433980131	-3.577719969	0.000346604	0.010519128
ENSMUSG00000043822	206.2443565	-1.54344184	0.315951893	-4.885053314	1.03E-06	9.34E-05
ENSMUSG00000034792	79.92056064	-1.541936437	0.400223435	-3.852689023	0.000116828	0.004674322
ENSMUSG00000057346	181.1535218	-1.537324224	0.308632972	-4.981075793	6.32E-07	6.25E-05
ENSMUSG00000097893	29.29337779	-1.537106007	0.426827825	-3.601231964	0.000316713	0.009951496
ENSMUSG00000030717	1534.883489	-1.536405174	0.24433879	-6.288011729	3.22E-10	9.07E-08
ENSMUSG00000050345	19.82859712	-1.535099193	0.441593871	-3.476269246	0.000508442	0.014235133
ENSMUSG00000027748	41.87455716	-1.531032873	0.407758846	-3.754750846	0.000173514	0.00626982
ENSMUSG00000033737	30.32788893	-1.526027214	0.430766529	-3.542585391	0.000396225	0.011628302
ENSMUSG00000043263	1754.274129	-1.518704469	0.442330161	-3.433418298	0.000596022	0.016216
ENSMUSG00000069874	985.780408	-1.514995005	0.285479637	-5.306840863	1.12E-07	1.36E-05
ENSMUSG00000082088	320.7701582	-1.514279954	0.324759411	-4.662774661	3.12E-06	0.000240491
ENSMUSG00000026358	2698.782973	-1.511869347	0.278788665	-5.422994326	5.86E-08	7.88E-06
ENSMUSG00000021775	249.7337608	-1.50899308	0.298218363	-5.060027377	4.19E-07	4.37E-05
ENSMUSG00000023341	591.9201425	-1.508667316	0.308260293	-4.894134444	9.87E-07	8.99E-05
ENSMUSG00000070031	490.00948	-1.502227229	0.2983244	-5.035549321	4.76E-07	4.88E-05
ENSMUSG00000036896	105.1756141	-1.500970979	0.361854349	-4.147997621	3.35E-05	0.001731332
ENSMUSG00000026893	17.89377752	-1.494229654	0.442396866	-3.377577399	0.000731274	0.019044409
ENSMUSG00000055202	423.3393467	-1.494066657	0.274335237	-5.446134706	5.15E-08	7.52E-06
ENSMUSG00000057337	19.81773875	-1.4899567	0.440508165	-3.382358875	0.000718662	0.018929129
ENSMUSG00000055069	98.59262679	-1.489898468	0.344412333	-4.325914966	1.52E-05	0.000896133
ENSMUSG00000037921	646.2005068	-1.483380217	0.284127823	-5.220819995	1.78E-07	2.08E-05
ENSMUSG00000024357	402.1268306	-1.48284435	0.296458217	-5.001866252	5.68E-07	5.66E-05

ENSMUSG00000033213	248.762932	-1.477765445	0.329154408	-4.489581203	7.14E-06	0.000505071
ENSMUSG00000027360	205.0675308	-1.469913767	0.309662085	-4.746831583	2.07E-06	0.000173133
ENSMUSG00000015377	44.30256422	-1.464384705	0.400931537	-3.652455772	0.000259744	0.00852215
ENSMUSG00000024679	5843.559714	-1.46364825	0.26924097	-5.436201821	5.44E-08	7.68E-06
ENSMUSG00000052485	66.44364115	-1.463149223	0.377621145	-3.874648548	0.000106779	0.004347472
ENSMUSG00000030156	146.6735739	-1.462312814	0.327636434	-4.463217959	8.07E-06	0.000555697
ENSMUSG00000035352	404.0835004	-1.461888872	0.313506707	-4.663022637	3.12E-06	0.000240491
ENSMUSG00000024164	3564.146859	-1.455933831	0.241767267	-6.022046938	1.72E-09	3.93E-07
ENSMUSG00000034855	3488.86687	-1.454827728	0.276317365	-5.265060803	1.40E-07	1.65E-05
ENSMUSG00000031639	303.0186896	-1.442935049	0.35740282	-4.037279419	5.41E-05	0.002531438
ENSMUSG00000056116	1292.544798	-1.442575975	0.254937461	-5.658548438	1.53E-08	2.60E-06
ENSMUSG00000057137	301.3278349	-1.438446929	0.296639296	-4.849144906	1.24E-06	0.000106996
ENSMUSG00000079457	43.15944821	-1.427941666	0.402594287	-3.546850295	0.000389866	0.011470792
ENSMUSG00000067297	106.9540024	-1.42636168	0.353686347	-4.032843479	5.51E-05	0.002558998
ENSMUSG00000079190	151.8205343	-1.423452989	0.340596626	-4.179292691	2.92E-05	0.001551018
ENSMUSG00000030107	5388.190418	-1.420423649	0.237974539	-5.96880513	2.39E-09	5.12E-07
ENSMUSG00000036890	249.332259	-1.418564725	0.291300057	-4.869771529	1.12E-06	9.94E-05
ENSMUSG00000091144	24.5960026	-1.41688355	0.433920894	-3.265303812	0.001093467	0.02605204
ENSMUSG00000039908	96.0452729	-1.410135918	0.363692736	-3.877272705	0.000105634	0.004316061
ENSMUSG00000031897	3103.062139	-1.40207818	0.257721933	-5.440274956	5.32E-08	7.59E-06
ENSMUSG00000026946	1385.913144	-1.398803435	0.29652804	-4.717272045	2.39E-06	0.000193594
ENSMUSG00000018986	304.1223332	-1.397002845	0.288057166	-4.849741686	1.24E-06	0.000106996
ENSMUSG00000046879	4691.502692	-1.389920722	0.299189307	-4.645622987	3.39E-06	0.000259633
ENSMUSG00000029392	141.849601	-1.386324479	0.324105123	-4.27739144	1.89E-05	0.001066594
ENSMUSG00000074578	1174.561807	-1.385201644	0.298292415	-4.643770926	3.42E-06	0.000260249
ENSMUSG00000021871	2927.090904	-1.382189174	0.237534461	-5.818899583	5.92E-09	1.10E-06
ENSMUSG00000017652	4395.769794	-1.37977512	0.278704094	-4.950681202	7.40E-07	7.07E-05
ENSMUSG00000021044	562.19958	-1.376837752	0.2674773	-5.147492821	2.64E-07	2.91E-05

	91		53			
ENSMUSG00000048852	21.96956914	-1.376673799	0.441398463	-3.118891237	0.00181533	0.036981636
ENSMUSG00000002307	1503.484955	-1.371955194	0.245071211	-5.598189967	2.17E-08	3.58E-06
ENSMUSG00000068923	126.8704963	-1.361601877	0.335639607	-4.05673779	4.98E-05	0.002417682
ENSMUSG00000090176	42.472507	-1.360092503	0.403695768	-3.369102699	0.000754133	0.01942103
ENSMUSG00000039209	70.35523933	-1.349780152	0.372133959	-3.627135122	0.000286583	0.009256322
ENSMUSG00000021423	53.28161583	-1.348309713	0.387956476	-3.475414892	0.000510064	0.014246058
ENSMUSG00000097554	106.069765	-1.341429694	0.408684692	-3.282309615	0.001029605	0.024906529
ENSMUSG00000039994	108.8099102	-1.338513276	0.356788229	-3.751562318	0.000175736	0.006315842
ENSMUSG00000034422	6204.813848	-1.337367139	0.266106596	-5.025682031	5.02E-07	5.04E-05
ENSMUSG00000003863	66.6626878	-1.335874672	0.394757469	-3.384038998	0.000714279	0.018856628
ENSMUSG00000078616	18.14658564	-1.33585043	0.4423136	-3.020143245	0.002526552	0.047170627
ENSMUSG00000029385	1167.426444	-1.334582521	0.245790125	-5.429764609	5.64E-08	7.68E-06
ENSMUSG00000020415	567.698177	-1.334493551	0.274323274	-4.864674923	1.15E-06	0.000101194
ENSMUSG00000044786	121.3902389	-1.333113201	0.330075636	-4.038811284	5.37E-05	0.002525188
ENSMUSG00000020707	384.6978932	-1.332211328	0.282431251	-4.716940221	2.39E-06	0.000193594
ENSMUSG00000018143	1384.72695	-1.331380363	0.273117786	-4.874747929	1.09E-06	9.77E-05
ENSMUSG00000079491	35.29913617	-1.330915196	0.4158009	-3.200847319	0.001370241	0.030335904
ENSMUSG00000043953	2214.216926	-1.328469466	0.250618427	-5.300765313	1.15E-07	1.39E-05
ENSMUSG00000030672	24.62239198	-1.320950331	0.435442971	-3.033578263	0.00241672	0.045515478
ENSMUSG00000025498	5100.032064	-1.307866336	0.238119524	-5.492478367	3.96E-08	6.03E-06
ENSMUSG00000026102	114.9359178	-1.30702911	0.346430584	-3.772845618	0.000161396	0.005905772
ENSMUSG00000045975	75.77543692	-1.306164173	0.374349867	-3.489153566	0.000484553	0.013632318
ENSMUSG00000024805	202.5152258	-1.300367008	0.322387306	-4.033555238	5.49E-05	0.002558998
ENSMUSG00000033781	79.25183791	-1.298833482	0.365515796	-3.553426411	0.000380248	0.011302834
ENSMUSG00000071068	667.2508221	-1.293831008	0.260492033	-4.966873616	6.80E-07	6.61E-05
ENSMUSG00000031936	27.20436287	-1.291819501	0.430954641	-2.997576495	0.002721356	0.049789611
ENSMUSG00000002814	511.0656941	-1.287759389	0.273736612	-4.704373961	2.55E-06	0.000200629

ENSMUSG00000064090	195.4326254	-1.286876879	0.328819545	-3.913626486	9.09E-05	0.003809097
ENSMUSG00000034206	102.7700219	-1.286292194	0.338081142	-3.80468483	0.000141985	0.005347788
ENSMUSG00000040253	2972.774381	-1.284720087	0.283112361	-4.537845265	5.68E-06	0.0004133
ENSMUSG00000023206	81.72782996	-1.28205439	0.359576008	-3.565461438	0.000363217	0.010937209
ENSMUSG00000001156	528.757561	-1.281638576	0.29303674	-4.373644675	1.22E-05	0.000739724
ENSMUSG00000033538	2197.391109	-1.280800799	0.280725737	-4.562463031	5.06E-06	0.000377155
ENSMUSG00000027438	50.98162574	-1.28039093	0.396812226	-3.226692236	0.001252301	0.02878892
ENSMUSG00000099757	52.73503292	-1.277618209	0.395174232	-3.233050402	0.00122476	0.028469505
ENSMUSG00000055447	3121.427478	-1.272513757	0.251943013	-5.050799955	4.40E-07	4.54E-05
ENSMUSG00000024672	1595.198616	-1.258645249	0.283882792	-4.433679268	9.26E-06	0.000619178
ENSMUSG00000028458	134.605752	-1.258562361	0.342635847	-3.673177726	0.000239553	0.00807173
ENSMUSG00000022335	264.2898074	-1.254152308	0.286392939	-4.37913139	1.19E-05	0.000728979
ENSMUSG00000062488	424.5936677	-1.251998215	0.32795266	-3.817618729	0.000134746	0.005159164
ENSMUSG00000024900	103.4821722	-1.251842938	0.346970979	-3.607918277	0.000308664	0.009751579
ENSMUSG00000047735	3172.445884	-1.251647798	0.335020934	-3.736028623	0.000186949	0.006570504
ENSMUSG00000045502	1428.505484	-1.250927766	0.278084588	-4.498371439	6.85E-06	0.000488758
ENSMUSG00000038213	388.9715055	-1.249625816	0.283270332	-4.411424968	1.03E-05	0.00066032
ENSMUSG00000037997	469.1926817	-1.245051564	0.301467848	-4.129964681	3.63E-05	0.001848139
ENSMUSG00000030966	635.3790553	-1.243405841	0.283803089	-4.381227296	1.18E-05	0.000725841
ENSMUSG00000035929	902.4303301	-1.243008148	0.256240378	-4.850945662	1.23E-06	0.000106996
ENSMUSG00000078566	1264.134233	-1.238893614	0.269034489	-4.604962062	4.13E-06	0.000309754
ENSMUSG00000032727	126.3104339	-1.232228203	0.335012399	-3.678157006	0.000234925	0.007966105
ENSMUSG00000023919	76.325793	-1.231899331	0.361808366	-3.404839262	0.00066203	0.017761146
ENSMUSG00000029605	249.1814591	-1.227458249	0.294506266	-4.167851044	3.07E-05	0.001616112
ENSMUSG00000035692	5488.531011	-1.218173612	0.238759853	-5.1020873	3.36E-07	3.60E-05
ENSMUSG00000026896	2126.176971	-1.21579539	0.28936503	-4.201597517	2.65E-05	0.001425412
ENSMUSG00000004952	871.4239976	-1.213613536	0.283324002	-4.283482962	1.84E-05	0.001044931
ENSMUSG00000059089	2188.2615	-1.210943216	0.2722688	-4.447600305	8.68E-06	0.00058

	76		94			7177
ENSMUSG00000079685	51.04228063	-1.209555355	0.392090718	-3.084886482	0.002036299	0.040456564
ENSMUSG00000025888	3540.896117	-1.207978709	0.328600576	-3.676130837	0.000236798	0.00800613
ENSMUSG00000072594	306.5012258	-1.192258811	0.295122431	-4.039878657	5.35E-05	0.002523984
ENSMUSG00000071350	396.587189	-1.186602322	0.329927049	-3.596559679	0.000322454	0.010104421
ENSMUSG00000004637	191.292594	-1.186601481	0.310699127	-3.819133617	0.000133921	0.005159164
ENSMUSG00000034218	482.9748332	-1.178297385	0.301060986	-3.913816266	9.08E-05	0.003809097
ENSMUSG00000032815	68.49781738	-1.176329769	0.37974858	-3.097654164	0.001950589	0.039089535
ENSMUSG00000027366	6415.057252	-1.1716703	0.29126918	-4.022637402	5.76E-05	0.002651201
ENSMUSG00000035248	891.4728528	-1.169694459	0.267953198	-4.365293887	1.27E-05	0.000760594
ENSMUSG00000024897	121.7891457	-1.169655747	0.326804145	-3.579072555	0.000344816	0.01049238
ENSMUSG00000045795	357.4629995	-1.165161912	0.271867561	-4.285770277	1.82E-05	0.001042428
ENSMUSG00000074220	115.8504003	-1.163424436	0.331058462	-3.514256751	0.000440986	0.012716023
ENSMUSG00000004500	163.8205183	-1.154581226	0.308519946	-3.742322789	0.000182327	0.006467019
ENSMUSG00000029366	242.5337639	-1.154299631	0.315257535	-3.661449781	0.000250792	0.008285452
ENSMUSG00000046410	377.4712465	-1.150328143	0.307073126	-3.746104904	0.000179602	0.006389945
ENSMUSG00000018899	1711.633763	-1.149733358	0.291540198	-3.943652941	8.02E-05	0.003488452
ENSMUSG00000021453	314.8672862	-1.145321746	0.279503499	-4.097700931	4.17E-05	0.002088718
ENSMUSG00000030921	506.5375592	-1.144148942	0.32241598	-3.54867318	0.000387177	0.011420743
ENSMUSG00000037321	3218.407045	-1.142833024	0.259227857	-4.40860422	1.04E-05	0.00066032
ENSMUSG00000038642	6870.221987	-1.140810965	0.27018719	-4.222298484	2.42E-05	0.001312773
ENSMUSG00000037190	312.726656	-1.140474511	0.3040491	-3.75095507	0.000176162	0.006315842
ENSMUSG00000027219	147.4466198	-1.121765668	0.315952562	-3.550424342	0.000384611	0.011374049
ENSMUSG00000036249	382.3997329	-1.120305319	0.284943622	-3.93167361	8.44E-05	0.003590475
ENSMUSG00000029780	247.7587796	-1.118390898	0.314260692	-3.558799832	0.000372553	0.011102665
ENSMUSG00000072258	128.108361	-1.118301526	0.326959874	-3.420302043	0.000625516	0.01693875
ENSMUSG00000025743	454.6435336	-1.117762714	0.271655862	-4.114627623	3.88E-05	0.001958161
ENSMUSG00000024397	165.0346098	-1.115380683	0.310980583	-3.586656992	0.000334944	0.010300432

ENSMUSG00000039501	3647.354491	-1.113719733	0.236757947	-4.70404371	2.55E-06	0.000200629
ENSMUSG00000033880	586.7018337	-1.111685656	0.269383161	-4.126782278	3.68E-05	0.001865672
ENSMUSG00000036009	194.8560127	-1.107916781	0.300153405	-3.691168461	0.000223226	0.007614056
ENSMUSG00000023067	636.5186622	-1.107810693	0.267630655	-4.139326608	3.48E-05	0.001782169
ENSMUSG00000032265	1233.886435	-1.104130052	0.254869984	-4.332130577	1.48E-05	0.000875665
ENSMUSG00000010051	121.3410194	-1.102554084	0.327930499	-3.362157797	0.000773359	0.019740291
ENSMUSG00000070327	14696.53746	-1.100320552	0.241741291	-4.551645052	5.32E-06	0.000392023
ENSMUSG00000063286	2160.937728	-1.099722667	0.316046961	-3.479617912	0.000502129	0.014092528
ENSMUSG00000032374	702.6199789	-1.097621449	0.315978443	-3.473722569	0.000513291	0.014301661
ENSMUSG00000054676	333.9678291	-1.094124746	0.293162614	-3.732142818	0.000189858	0.006652502
ENSMUSG00000030102	428.7124817	-1.093077605	0.269700669	-4.052928786	5.06E-05	0.002447118
ENSMUSG00000040033	832.2258609	-1.089556588	0.256688376	-4.244666653	2.19E-05	0.001216998
ENSMUSG00000022014	956.7448843	-1.086074501	0.286012605	-3.797295933	0.000146283	0.005438816
ENSMUSG00000079808	174.4721819	-1.080981247	0.307135798	-3.51955472	0.000432272	0.012496904
ENSMUSG00000029561	1583.449357	-1.078876187	0.279253324	-3.863431851	0.000111805	0.004520288
ENSMUSG00000051316	238.4953835	-1.078684884	0.302652987	-3.564097931	0.00036511	0.010937209
ENSMUSG00000037638	127.4526645	-1.075090485	0.334865106	-3.210518101	0.001324959	0.029748551
ENSMUSG00000019796	234.862388	-1.073086562	0.292853352	-3.664245441	0.000248069	0.008242585
ENSMUSG00000019794	518.6785443	-1.071037662	0.282024586	-3.797674797	0.00014606	0.005438816
ENSMUSG00000045414	270.5778607	-1.067838204	0.29958108	-3.564438066	0.000364637	0.010937209
ENSMUSG00000024308	3479.317076	-1.067305428	0.277294292	-3.84899891	0.000118602	0.004728929
ENSMUSG00000026764	152.0468182	-1.062886871	0.316721306	-3.355905812	0.000791055	0.020059144
ENSMUSG00000053318	299.1963472	-1.058313909	0.279811275	-3.782241826	0.000155422	0.005741686
ENSMUSG00000021037	1648.37291	-1.055249156	0.240786548	-4.382508755	1.17E-05	0.000725442
ENSMUSG00000043740	157.277682	-1.05402167	0.326544567	-3.227803415	0.001247447	0.02878892
ENSMUSG00000024787	260.6156506	-1.052683102	0.29409292	-3.579423479	0.000344353	0.01049238
ENSMUSG00000041075	106.7219461	-1.047700117	0.337060347	-3.108345811	0.001881378	0.037833691
ENSMUSG00000081957	114.65601	-1.047481917	0.3364782	-3.113074884	0.001851	0.03749

	33		27		49	349
ENSMUSG00000008384	478.7309459	-1.046756874	0.280130634	-3.736674055	0.00018647	0.006570504
ENSMUSG00000040195	112.6924123	-1.046177386	0.330752484	-3.163022014	0.001561405	0.033310939
ENSMUSG00000005483	535.2127547	-1.042254594	0.263088874	-3.961606496	7.44E-05	0.003298209
ENSMUSG00000029426	1164.95629	-1.042191708	0.248010661	-4.202205282	2.64E-05	0.001425412
ENSMUSG00000040483	1045.715571	-1.037514528	0.262355941	-3.954606571	7.67E-05	0.003383312
ENSMUSG00000023186	2436.986358	-1.034724827	0.27246311	-3.797669439	0.000146063	0.005438816
ENSMUSG00000025647	1858.114749	-1.032093614	0.239956852	-4.30116334	1.70E-05	0.000987235
ENSMUSG00000054423	329.5851224	-1.031639981	0.305607713	-3.37570008	0.000736281	0.019131733
ENSMUSG00000032376	456.7109845	-1.030027782	0.26408503	-3.900364148	9.60E-05	0.003966444
ENSMUSG00000025757	1655.923008	-1.026636365	0.26965158	-3.807269984	0.000140509	0.005309508
ENSMUSG00000079625	587.7616661	-1.02499536	0.261088593	-3.925852717	8.64E-05	0.003660474
ENSMUSG00000042350	1772.125651	-1.023397547	0.241795423	-4.23249347	2.31E-05	0.001272561
ENSMUSG00000029771	1448.850379	-1.017276936	0.251125943	-4.050863577	5.10E-05	0.002458532
ENSMUSG00000083899	1218.611802	-1.016820677	0.261324222	-3.891031112	9.98E-05	0.004107504
ENSMUSG00000039298	499.0240595	-1.016622084	0.268515598	-3.78608204	0.000153041	0.005671843
ENSMUSG00000029298	235.5360594	-1.016333278	0.300685584	-3.380053227	0.000724718	0.019011113
ENSMUSG00000096727	900.6623296	-1.015799017	0.27436775	-3.702326592	0.000213631	0.007395867
ENSMUSG00000021707	108.00409	-1.014643674	0.335710591	-3.022376122	0.002507987	0.046925332
ENSMUSG00000044811	338.4567599	-1.013577069	0.281897205	-3.595555589	0.0003237	0.010116062
ENSMUSG00000038352	198.4492578	-1.013371749	0.319437492	-3.172363217	0.001512038	0.032618828
ENSMUSG00000035521	210.6766351	-1.009976265	0.306383645	-3.296443145	0.000979174	0.023936982
ENSMUSG00000087477	211.8415589	-1.008855736	0.297879304	-3.38679365	0.000707146	0.018753954
ENSMUSG00000022901	1368.190338	-1.008428665	0.256333589	-3.934048078	8.35E-05	0.003577124
ENSMUSG00000036986	155.8578541	-1.008104404	0.316878135	-3.181363099	0.001465838	0.03191993
ENSMUSG00000051029	139.2587823	-1.006236295	0.331701791	-3.033557013	0.00241689	0.045515478
ENSMUSG00000032673	407.8301796	-1.002864791	0.274728184	-3.650389183	0.000261843	0.008528715
ENSMUSG00000020526	148.6873065	1.001295122	0.319921553	3.129814515	0.001749167	0.036181788

ENSMUSG00000050967	124.2788711	1.001726136	0.326256447	3.070364268	0.002137978	0.042043272
ENSMUSG00000056313	129.5759823	1.003599231	0.329071055	3.049794921	0.002289977	0.043796651
ENSMUSG00000057191	132.0067643	1.005999212	0.327842753	3.068541862	0.002151062	0.042157167
ENSMUSG00000006517	349.0603471	1.013685553	0.289939732	3.49619401	0.000471945	0.013439512
ENSMUSG00000025586	257.1550601	1.01437481	0.285682118	3.550711595	0.000384191	0.011374049
ENSMUSG00000027993	191.1316685	1.018724802	0.300917464	3.385396077	0.000710756	0.018806576
ENSMUSG00000030786	946.500579	1.024271792	0.249324991	4.108179394	3.99E-05	0.002004875
ENSMUSG00000028803	114.0038554	1.027799901	0.337859994	3.042088202	0.00234943	0.044605086
ENSMUSG00000048578	2470.231977	1.032174296	0.242970232	4.248151261	2.16E-05	0.001204015
ENSMUSG00000064923	1034.218063	1.032238813	0.325959234	3.166772729	0.001541407	0.033067334
ENSMUSG00000037366	675.3388545	1.037038865	0.256456207	4.043726901	5.26E-05	0.002513672
ENSMUSG00000036632	265.6501713	1.038029075	0.286588866	3.622014663	0.000292318	0.009380398
ENSMUSG00000043510	146.4840255	1.041442732	0.323508584	3.219212053	0.001285434	0.02920132
ENSMUSG00000033554	167.715526	1.044847836	0.305880148	3.415873314	0.000635778	0.017176409
ENSMUSG00000071637	400.1037977	1.045390577	0.333202352	3.137404551	0.001704508	0.03544824
ENSMUSG00000020736	222.5265798	1.046933898	0.310617362	3.370493819	0.000750336	0.01936637
ENSMUSG00000030263	330.3630157	1.047316262	0.283033196	3.700330125	0.000215319	0.007409928
ENSMUSG00000020547	237.6433885	1.051224118	0.286951394	3.663422233	0.000248868	0.008245439
ENSMUSG00000071337	117.0104996	1.054960569	0.327200417	3.224203012	0.001263238	0.0289244
ENSMUSG00000017485	265.3749169	1.057554281	0.29714858	3.559008366	0.000372258	0.011102665
ENSMUSG00000038776	145.2477836	1.060805313	0.330643945	3.208301044	0.001335217	0.029820457
ENSMUSG00000023349	149.9074422	1.067596316	0.33011861	3.233977983	0.001220789	0.028459636
ENSMUSG00000027395	165.1837332	1.069047296	0.305752967	3.496441287	0.000471508	0.013439512
ENSMUSG00000030272	500.3533035	1.072849061	0.263758692	4.06754012	4.75E-05	0.002318066
ENSMUSG00000030802	277.3400447	1.074485857	0.280047469	3.836799033	0.000124648	0.004880636
ENSMUSG00000038963	1701.926065	1.07461918	0.248713365	4.320713443	1.56E-05	0.000912864
ENSMUSG00000081534	1408.555732	1.074766075	0.244743638	4.391395356	1.13E-05	0.000707766
ENSMUSG00000093956	2733.1092	1.076583305	0.2900719	3.71143489	0.000206	0.00715

	91		9		088	6132
ENSMUSG00000018068	134.6894832	1.07890413	0.319209727	3.379922478	0.000725063	0.019011113
ENSMUSG00000009995	200.1609871	1.07971982	0.31966029	3.377710195	0.000730921	0.019044409
ENSMUSG00000025934	136.8393423	1.08038629	0.355201396	3.041616111	0.002353118	0.044605086
ENSMUSG00000028969	206.6683461	1.084832586	0.297745582	3.643488446	0.000268968	0.008736163
ENSMUSG00000024067	95.67095177	1.089573584	0.344858268	3.159482276	0.001580497	0.03359428
ENSMUSG00000025511	245.7112458	1.090033949	0.314898924	3.461535961	0.000537102	0.01485769
ENSMUSG00000023021	529.3975226	1.091572927	0.260612671	4.188487545	2.81E-05	0.001496368
ENSMUSG00000037706	681.3679045	1.096704232	0.262606802	4.176221726	2.96E-05	0.001564917
ENSMUSG00000046982	159.2846578	1.097154531	0.30750105	3.567970036	0.000359758	0.010889732
ENSMUSG00000014551	192.6231895	1.099590286	0.304065837	3.61629013	0.000298855	0.009467575
ENSMUSG00000040945	575.9297325	1.099764622	0.306369846	3.589663395	0.000331105	0.01020952
ENSMUSG00000031659	1035.25558	1.109221934	0.26167158	4.238985121	2.25E-05	0.001242234
ENSMUSG00000039640	225.1304797	1.121299945	0.3053185	3.672558147	0.000240134	0.00807173
ENSMUSG00000045404	375.5129467	1.123403033	0.281276186	3.993950038	6.50E-05	0.002923665
ENSMUSG00000026833	122.7884033	1.127149949	0.336942887	3.345225536	0.000822157	0.020711546
ENSMUSG00000028278	211.8769711	1.130698348	0.294640714	3.837549572	0.000124268	0.004880636
ENSMUSG00000021496	64.42635848	1.131295954	0.369104555	3.06497424	0.002176887	0.042412389
ENSMUSG00000046223	661.7337799	1.13951841	0.297105627	3.835398283	0.000125361	0.004880636
ENSMUSG00000044303	692.1367042	1.140568988	0.274926835	4.148627356	3.34E-05	0.001731332
ENSMUSG00000027357	541.2564693	1.142858271	0.260662559	4.384435868	1.16E-05	0.000722916
ENSMUSG00000028680	67.54515415	1.144768462	0.370159362	3.092636795	0.001983868	0.039687652
ENSMUSG00000037940	135.1557822	1.15226682	0.320792454	3.591938665	0.000328227	0.01017004
ENSMUSG00000050578	92.72588597	1.15260495	0.356043171	3.237261777	0.001206827	0.028305345
ENSMUSG00000006288	119.3912645	1.153701878	0.343685269	3.356855772	0.000788342	0.020034289
ENSMUSG00000022372	198.8784796	1.155683636	0.301318299	3.835424662	0.000125348	0.004880636
ENSMUSG00000024841	457.2931954	1.157966163	0.269520621	4.29639172	1.74E-05	0.001003672
ENSMUSG00000022386	53.49093864	1.161002933	0.383216281	3.029628413	0.002448548	0.045961947

ENSMUSG00000057411	252.8400747	1.165722154	0.330822032	3.523713783	0.000425544	0.012363219
ENSMUSG00000022742	125.0658189	1.167876082	0.324878075	3.594813473	0.000324624	0.010117026
ENSMUSG00000031730	102.6376494	1.169286146	0.344261177	3.396508881	0.000682513	0.018268293
ENSMUSG00000039230	486.3729501	1.170516596	0.262370132	4.461318009	8.15E-06	0.00055733
ENSMUSG00000020321	1830.841441	1.170538913	0.245568875	4.766642005	1.87E-06	0.000158102
ENSMUSG00000001482	88.6877201	1.170631483	0.365237363	3.205125222	0.001350037	0.030077999
ENSMUSG00000062585	53.34096985	1.17503658	0.388323869	3.025919015	0.002478787	0.046454153
ENSMUSG00000056758	54.1062551	1.180183081	0.382294442	3.087104991	0.002021162	0.04022495
ENSMUSG00000060261	142.5159881	1.180473794	0.317981519	3.712397492	0.000205305	0.007150435
ENSMUSG00000010142	244.8129332	1.185145335	0.28932203	4.096284452	4.20E-05	0.002092474
ENSMUSG00000078789	75.70523453	1.186248974	0.36976059	3.208154156	0.001335899	0.029820457
ENSMUSG00000032295	123.95779	1.188816249	0.324223208	3.666659944	0.000245739	0.008199094
ENSMUSG00000041654	506.4092626	1.196775469	0.269206792	4.445561941	8.77E-06	0.000589326
ENSMUSG00000022150	143.3443239	1.202374796	0.316325449	3.801068805	0.000144073	0.005408829
ENSMUSG00000020864	373.9275449	1.204602623	0.280511379	4.294309298	1.75E-05	0.001008098
ENSMUSG00000024892	766.7415392	1.20933722	0.255109229	4.740468324	2.13E-06	0.000176108
ENSMUSG00000083854	59.39638185	1.212406156	0.377214538	3.214102412	0.00130853	0.029609651
ENSMUSG00000021240	378.7173886	1.216872161	0.308465887	3.944916482	7.98E-05	0.003483203
ENSMUSG00000026749	484.5858191	1.22565721	0.276787327	4.428155091	9.50E-06	0.000627987
ENSMUSG00000046245	302.5351676	1.226966337	0.277312048	4.424497043	9.67E-06	0.000635093
ENSMUSG00000018574	235.1322843	1.227203973	0.303437761	4.044335054	5.25E-05	0.002513672
ENSMUSG00000020692	70.71312477	1.23000639	0.365671242	3.36369462	0.000769066	0.019682378
ENSMUSG00000100104	148.3652284	1.230589385	0.32624718	3.771954085	0.000161974	0.005908221
ENSMUSG00000001128	228.6219771	1.24254757	0.29420619	4.223390301	2.41E-05	0.001312589
ENSMUSG00000028480	74.7401936	1.248065962	0.361089545	3.456389089	0.000547464	0.015072218
ENSMUSG00000024026	98.27553409	1.254841623	0.346494273	3.621536407	0.000292859	0.009380398
ENSMUSG00000020142	111.6323341	1.257911605	0.351086509	3.582910685	0.000339787	0.010421631
ENSMUSG00000018509	48.895452	1.258066157	0.3973843	3.165867146	0.001546	0.03310

	63		81		214	9022
ENSMUSG00000057181	132.1495623	1.258502306	0.32190366	3.909561961	9.25E-05	0.003859773
ENSMUSG00000065767	423.0878788	1.260024873	0.32463142	3.881401473	0.000103856	0.004258474
ENSMUSG00000040029	316.1673226	1.261809721	0.276839971	4.557902955	5.17E-06	0.000382964
ENSMUSG00000034863	172.5572918	1.265226579	0.346508757	3.651355283	0.00026086	0.00852215
ENSMUSG00000089917	283.458601	1.265819642	0.289921267	4.366080685	1.26E-05	0.000760594
ENSMUSG00000021696	65.45336066	1.265997801	0.374804673	3.377753515	0.000730806	0.019044409
ENSMUSG00000034445	150.7063378	1.267568076	0.316211812	4.008604444	6.11E-05	0.002758797
ENSMUSG00000001020	1010.914198	1.283270189	0.287515369	4.463309893	8.07E-06	0.000555697
ENSMUSG00000033287	28.71059051	1.285312797	0.426760951	3.011786329	0.002597153	0.048281165
ENSMUSG00000036775	388.0347925	1.290720806	0.292499648	4.412726007	1.02E-05	0.00066032
ENSMUSG00000047786	129.4698414	1.291116684	0.321196409	4.019710824	5.83E-05	0.002672282
ENSMUSG00000051705	39.40548451	1.296412003	0.409189573	3.168243008	0.001533633	0.032961701
ENSMUSG00000017002	1658.865	1.301530279	0.269203814	4.834739373	1.33E-06	0.000114181
ENSMUSG00000030707	3461.161888	1.303939329	0.269783015	4.833289188	1.34E-06	0.000114181
ENSMUSG00000038172	108.8583286	1.307697657	0.334082664	3.914293673	9.07E-05	0.003809097
ENSMUSG00000017716	29.33578754	1.31622414	0.427474881	3.0790678	0.002076494	0.041113874
ENSMUSG00000025728	93.7096012	1.320015014	0.34630769	3.811682656	0.000138024	0.005249907
ENSMUSG00000005299	226.9795218	1.320661411	0.299587535	4.408265559	1.04E-05	0.00066032
ENSMUSG00000044716	303.0577958	1.321960501	0.292637649	4.517397217	6.26E-06	0.000449624
ENSMUSG00000022228	167.1993765	1.324877522	0.325131567	4.074896609	4.60E-05	0.00225551
ENSMUSG00000024925	139.7880194	1.330805026	0.32935521	4.040637538	5.33E-05	0.002523984
ENSMUSG00000045160	37.84139481	1.331949415	0.407958825	3.264911394	0.001094983	0.02605204
ENSMUSG00000039043	41.91872675	1.332517159	0.407148973	3.272800001	0.001064878	0.025652467
ENSMUSG00000055302	762.7822171	1.333801974	0.262868967	5.074018397	3.90E-07	4.09E-05
ENSMUSG00000022885	99.78370795	1.334676124	0.341754176	3.905368882	9.41E-05	0.003908752
ENSMUSG00000010663	869.2220045	1.336318805	0.257616275	5.187245281	2.13E-07	2.47E-05
ENSMUSG00000018334	35.045698	1.337443876	0.417359465	3.20453707	0.001352799	0.03008156

ENSMUSG00000051339	15.80466884	1.341628415	0.442374959	3.032785621	0.002423077	0.045557782
ENSMUSG00000002329	29.79137218	1.347797934	0.42694074	3.15687356	0.001594705	0.033772109
ENSMUSG00000030785	51.60626706	1.351349296	0.405471384	3.332785863	0.000859811	0.021554317
ENSMUSG00000021918	59.0351763	1.365412006	0.386023925	3.537117564	0.00040452	0.011811767
ENSMUSG00000020407	76.55951622	1.367569421	0.386406123	3.539202255	0.000401338	0.011748542
ENSMUSG00000028247	83.18609973	1.371375592	0.355801769	3.854324824	0.000116049	0.004659304
ENSMUSG00000024247	155.3490772	1.371654136	0.354552703	3.868688979	0.000109422	0.004439466
ENSMUSG00000026558	1050.877697	1.388428703	0.258309588	5.375056778	7.66E-08	1.01E-05
ENSMUSG00000000318	147.4199209	1.39031165	0.315432425	4.407637071	1.05E-05	0.00066032
ENSMUSG00000040950	36.05618893	1.402338387	0.413959104	3.387625425	0.000705005	0.018740161
ENSMUSG00000089715	83.52621144	1.409314807	0.373011768	3.778204678	0.000157963	0.005798497
ENSMUSG00000037071	16.3726183	1.412920783	0.441474957	3.200455113	0.001372107	0.030335904
ENSMUSG00000042286	70.19556331	1.418149277	0.371874999	3.813510665	0.000137007	0.005228407
ENSMUSG00000027398	8881.804055	1.419941279	0.234331431	6.059542567	1.37E-09	3.29E-07
ENSMUSG00000001524	101.6091559	1.424870536	0.339430109	4.197831888	2.69E-05	0.001442605
ENSMUSG00000049792	158.0707258	1.437837868	0.33570448	4.283046409	1.84E-05	0.001044931
ENSMUSG00000036564	22.58426866	1.440863615	0.43586055	3.305790384	0.000947089	0.023350088
ENSMUSG00000034194	16.3733296	1.441909939	0.442508501	3.258490937	0.001120065	0.026594058
ENSMUSG00000024667	38.31144768	1.442373671	0.412759382	3.494466108	0.000475011	0.013462135
ENSMUSG00000078484	86.97482753	1.448116711	0.36525903	3.964629463	7.35E-05	0.003269218
ENSMUSG00000046329	17.30257565	1.455966017	0.442523355	3.290145031	0.001001358	0.024427632
ENSMUSG00000026821	246.3124838	1.467389175	0.311319672	4.713448281	2.44E-06	0.000194226
ENSMUSG00000022304	145.1067726	1.467463822	0.332736265	4.410291195	1.03E-05	0.00066032
ENSMUSG00000025731	58.94500101	1.468011368	0.410045527	3.580117992	0.000343439	0.01049238
ENSMUSG00000103726	49.76464245	1.471174257	0.391512708	3.757666679	0.000171505	0.006216658
ENSMUSG00000005150	103.3258743	1.474209526	0.354819451	4.154815985	3.26E-05	0.001695643
ENSMUSG00000003849	103.3903257	1.475749635	0.368000461	4.0101842	6.07E-05	0.00275115
ENSMUSG00000094050	198.31747	1.495483829	0.3029309	4.936715278	7.94E-07	7.53E-05

	04		46			
ENSMUSG00000090230	23.44250907	1.50963614	0.43739276	3.451442905	0.000557598	0.015278442
ENSMUSG00000058325	129.698633	1.52232748	0.322663837	4.717998439	2.38E-06	0.000193594
ENSMUSG00000025153	518.7085297	1.52478735	0.280690106	5.432280359	5.56E-08	7.68E-06
ENSMUSG00000058427	7117.287536	1.526857774	0.253591958	6.02092348	1.73E-09	3.93E-07
ENSMUSG00000027698	1200.208612	1.529174	0.247048218	6.189779509	6.02E-10	1.58E-07
ENSMUSG00000083679	32.95174035	1.54234209	0.421401344	3.660031259	0.000252184	0.008307718
ENSMUSG00000021993	60.58275849	1.549835743	0.379232881	4.086765205	4.37E-05	0.002161537
ENSMUSG00000003549	51.36177759	1.556609454	0.395458149	3.936217928	8.28E-05	0.003558129
ENSMUSG00000001289	468.6910368	1.562854009	0.270663969	5.774148715	7.73E-09	1.38E-06
ENSMUSG00000046380	30.79535371	1.586629758	0.4358859	3.640011655	0.000272626	0.008830171
ENSMUSG00000055805	57.92750499	1.58722225	0.381573479	4.159676546	3.19E-05	0.001667471
ENSMUSG00000038067	1569.53546	1.587259679	0.296122993	5.360136558	8.32E-08	1.06E-05
ENSMUSG00000047284	94.79601302	1.616201255	0.396229337	4.078954037	4.52E-05	0.00222594
ENSMUSG00000021596	341.6339263	1.644420634	0.276968896	5.937203272	2.90E-09	5.88E-07
ENSMUSG00000071654	90.84622526	1.646804094	0.347367411	4.740813448	2.13E-06	0.000176108
ENSMUSG00000034041	80.47055622	1.647278194	0.37633095	4.377206267	1.20E-05	0.000731574
ENSMUSG00000028671	209.7960576	1.655502632	0.313970992	5.272788481	1.34E-07	1.60E-05
ENSMUSG00000049130	79.75722677	1.665360699	0.359107442	4.637499819	3.53E-06	0.000266515
ENSMUSG00000027546	69.6857243	1.665892837	0.38412915	4.336804004	1.45E-05	0.000861679
ENSMUSG00000021384	155.0953715	1.680164783	0.326873128	5.14011291	2.75E-07	2.99E-05
ENSMUSG00000027399	3863.478321	1.705658413	0.278728098	6.119434762	9.39E-10	2.32E-07
ENSMUSG00000059498	2451.839645	1.717498731	0.242484875	7.082910753	1.41E-12	7.42E-10
ENSMUSG00000001270	163.4835296	1.750043165	0.315425996	5.548189396	2.89E-08	4.51E-06
ENSMUSG00000026068	33.78693834	1.760205438	0.424589701	4.145662118	3.39E-05	0.001741301
ENSMUSG00000000168	344.2617923	1.767073208	0.275387461	6.416679977	1.39E-10	4.13E-08
ENSMUSG00000047264	37.20605449	1.771404101	0.418947903	4.228220476	2.36E-05	0.001290819
ENSMUSG00000043207	237.6739054	1.793077198	0.312161766	5.744064108	9.24E-09	1.62E-06

ENSMUSG00000043008	90.37831251	1.807413524	0.355210834	5.088283778	3.61E-07	3.83E-05
ENSMUSG00000048307	89.85017965	1.817114863	0.35124983	5.173283256	2.30E-07	2.62E-05
ENSMUSG00000004609	58.59619887	1.819585294	0.405746423	4.4845381	7.31E-06	0.00051208
ENSMUSG00000018500	34.47979535	1.836782025	0.429700449	4.274563892	1.92E-05	0.001074976
ENSMUSG00000026047	62.21398368	1.857289807	0.378055512	4.912743626	8.98E-07	8.24E-05
ENSMUSG00000009681	31.48552433	1.865221817	0.424909834	4.389688515	1.14E-05	0.000709488
ENSMUSG00000044103	80.75006771	1.927521785	0.387534406	4.973808139	6.57E-07	6.43E-05
ENSMUSG00000022747	102.0709427	1.94558294	0.341208061	5.702042722	1.18E-08	2.04E-06
ENSMUSG00000017737	18.22723895	1.983458595	0.442518886	4.482201001	7.39E-06	0.000514603
ENSMUSG00000018916	19.34671877	1.986369849	0.442519716	4.488771412	7.16E-06	0.000505071
ENSMUSG00000026829	24.31110873	1.986374913	0.437266748	4.542707451	5.55E-06	0.000406434
ENSMUSG00000029379	62.04263505	1.991934053	0.403809252	4.932858881	8.10E-07	7.56E-05
ENSMUSG00000047250	132.800826	2.015615703	0.325154132	6.198954603	5.68E-10	1.53E-07
ENSMUSG00000030960	62.76297434	2.04843119	0.381453621	5.370066179	7.87E-08	1.01E-05
ENSMUSG00000041220	68.49951531	2.085280634	0.381677951	5.463455852	4.67E-08	7.01E-06
ENSMUSG00000070283	96.96031381	2.117548739	0.355357261	5.958929142	2.54E-09	5.24E-07
ENSMUSG00000024669	179.7678294	2.234781907	0.311555133	7.172990178	7.34E-13	4.24E-10
ENSMUSG00000074604	66.55794993	2.285892389	0.382860623	5.970560176	2.36E-09	5.12E-07
ENSMUSG00000037787	65.74973885	2.686822048	0.385951356	6.961556179	3.37E-12	1.50E-09
ENSMUSG00000062345	80.6483036	2.94018333	0.400029776	7.349911192	1.98E-13	1.53E-10
ENSMUSG00000049723	1151.579155	3.22164517	0.330957961	9.7343033	2.15E-22	4.98E-19

Supplementary Table S3. Differentially expressed genes of pair-wise comparison 3 (LPS+FruArg vs. LPS)

FruArg+LPS vs. LPS						
ID	baseMean	log2FoldChange	lfcSE	stat	p-value	padj
ENSMUSG00000000386	788.0235432	-2.235481835	0.343278528	-6.512151663	7.41E-11	2.30E-07
ENSMUSG00000019813	144.1251398	-2.200449304	0.354098668	-6.214226441	5.16E-10	9.60E-07
ENSMUSG00000078763	132.7147174	-2.042579125	0.367241344	-5.561953075	2.67E-08	2.07E-05

ENSMUSG00000000204	1251.715467	-1.974215744	0.329098912	-5.998852236	1.99E-09	2.12E-06
ENSMUSG00000033213	189.6375752	-1.901908615	0.366527544	-5.188992339	2.11E-07	0.000140514
ENSMUSG00000035352	319.1598008	-1.783134413	0.320620945	-5.561503212	2.67E-08	2.07E-05
ENSMUSG00000097554	81.38381408	-1.726250162	0.406400722	-4.247655256	2.16E-05	0.004101725
ENSMUSG00000038179	1470.714169	-1.714070338	0.285978004	-5.99371391	2.05E-09	2.12E-06
ENSMUSG00000028037	370.8285516	-1.646890566	0.356036332	-4.625625022	3.73E-06	0.001085865
ENSMUSG00000070883	76.26694314	-1.636373477	0.394783144	-4.144993274	3.40E-05	0.005183147
ENSMUSG00000069793	334.4582932	-1.63338279	0.350248717	-4.663494001	3.11E-06	0.000992325
ENSMUSG00000038239	95.49179304	-1.631397143	0.388771476	-4.196288162	2.71E-05	0.004674829
ENSMUSG00000039200	87.59188832	-1.625094294	0.369763707	-4.394953485	1.11E-05	0.002577126
ENSMUSG00000047675	275.138289	-1.614019346	0.3725385	-4.332490048	1.47E-05	0.003190024
ENSMUSG00000038587	81.8999377	-1.568281847	0.383536829	-4.088999359	4.33E-05	0.005966529
ENSMUSG00000092564	122.2164513	-1.52592675	0.385707333	-3.956177706	7.62E-05	0.008609893
ENSMUSG00000050395	73.43209207	-1.509043627	0.378121861	-3.990892305	6.58E-05	0.007851761
ENSMUSG00000020826	3394.404724	-1.494103427	0.299247654	-4.99286596	5.95E-07	0.000368996
ENSMUSG00000030717	1281.882864	-1.471125041	0.299941448	-4.904707413	9.36E-07	0.000440876
ENSMUSG00000078851	71.89363012	-1.462475329	0.380655054	-3.841996354	0.000122038	0.012209014
ENSMUSG00000026358	2259.42816	-1.459942732	0.312027306	-4.678894143	2.88E-06	0.000991888
ENSMUSG00000070031	403.5893175	-1.459523051	0.349722689	-4.173372498	3.00E-05	0.00489886
ENSMUSG00000106246	78.54075009	-1.44709363	0.401622201	-3.603121604	0.000314418	0.0219951
ENSMUSG00000091649	682.4533969	-1.441954951	0.326105462	-4.421744252	9.79E-06	0.002335717
ENSMUSG00000055069	81.83830253	-1.438068562	0.367383652	-3.914350985	9.06E-05	0.009694094
ENSMUSG00000054404	964.2846047	-1.435045346	0.308110471	-4.657567597	3.20E-06	0.000992325
ENSMUSG00000088148	147.5400981	-1.421627631	0.363508575	-3.910850332	9.20E-05	0.009723923
ENSMUSG00000030156	122.6512579	-1.417760587	0.344944326	-4.110114248	3.95E-05	0.005660602
ENSMUSG00000035208	763.4709203	-1.385979771	0.323686308	-4.281860977	1.85E-05	0.003668879
ENSMUSG00000034855	2959.04242	-1.373247166	0.293314897	-4.68181869	2.84E-06	0.000991888

ENSMUSG00000037921	543.3578592	-1.371889662	0.330069249	-4.156369206	3.23E-05	0.005098988
ENSMUSG00000039304	96.31359957	-1.362657112	0.391519615	-3.480431275	0.000500607	0.029293395
ENSMUSG00000045827	1792.063232	-1.338214043	0.337378772	-3.966503389	7.29E-05	0.00837759
ENSMUSG00000037849	96.70123855	-1.334492573	0.37521825	-3.556576935	0.000375718	0.024617498
ENSMUSG00000027639	4847.805523	-1.331916026	0.300747236	-4.428689165	9.48E-06	0.002321288
ENSMUSG00000054423	256.1062211	-1.330276287	0.323610045	-4.110738543	3.94E-05	0.005660602
ENSMUSG00000021037	1292.617184	-1.318481795	0.28052631	-4.700029007	2.60E-06	0.000991888
ENSMUSG00000047735	2612.958919	-1.315515668	0.339473159	-3.875168432	0.000106551	0.011015003
ENSMUSG00000082088	270.5857457	-1.308928722	0.374702904	-3.493244135	0.00047719	0.028738541
ENSMUSG00000046169	51.75186101	-1.308321684	0.396665275	-3.298301528	0.000972716	0.045939855
ENSMUSG00000058163	130.807983	-1.307755624	0.398443248	-3.282162841	0.001030141	0.046982507
ENSMUSG00000079419	772.1754949	-1.303131707	0.30161	-4.320585209	1.56E-05	0.00329057
ENSMUSG00000086109	162.1138329	-1.290945573	0.318256802	-4.056301598	4.99E-05	0.006626558
ENSMUSG00000028268	5266.375486	-1.288379408	0.31833284	-4.047271429	5.18E-05	0.006790366
ENSMUSG00000020641	44858.07141	-1.28156365	0.322277594	-3.976583156	6.99E-05	0.008130831
ENSMUSG00000015947	2820.807939	-1.280422236	0.258684663	-4.949741603	7.43E-07	0.000384111
ENSMUSG00000001156	446.3700841	-1.275903222	0.276262054	-4.618452667	3.87E-06	0.001090012
ENSMUSG00000039236	549.3315939	-1.27413331	0.323864258	-3.934158463	8.35E-05	0.009247366
ENSMUSG00000068245	3033.176058	-1.271579067	0.323495342	-3.930749234	8.47E-05	0.009269144
ENSMUSG00000004500	131.6972901	-1.269162875	0.339836757	-3.734625071	0.000187995	0.016147409
ENSMUSG00000027360	178.0897205	-1.26237533	0.322452562	-3.914917969	9.04E-05	0.009694094
ENSMUSG00000044703	163.1411244	-1.259161763	0.332208925	-3.790270723	0.000150483	0.013739245
ENSMUSG00000029798	1748.834986	-1.244719429	0.33342242	-3.733160561	0.000189092	0.016147409
ENSMUSG00000023341	522.1455292	-1.244439679	0.312439315	-3.982980437	6.81E-05	0.008015144
ENSMUSG00000033538	1845.980564	-1.239625002	0.303273282	-4.087485031	4.36E-05	0.005966529
ENSMUSG00000053846	128.4683327	-1.23784384	0.345185532	-3.586024686	0.000335757	0.022567742
ENSMUSG00000048621	186.3475727	-1.233239325	0.333532156	-3.697512531	0.000217722	0.017715493

ENSMUSG00000034459	4853.779035	-1.217570437	0.334062506	-3.644738377	0.000267664	0.019343251
ENSMUSG00000067889	140.7074705	-1.216788152	0.353418167	-3.442913426	0.000575484	0.03168224
ENSMUSG00000021707	84.71581859	-1.212129779	0.362823339	-3.340826368	0.000835294	0.040903049
ENSMUSG00000074578	1037.783792	-1.19787739	0.255750554	-4.683772407	2.82E-06	0.000991888
ENSMUSG00000034218	400.0492899	-1.188928018	0.323986958	-3.669678632	0.000242856	0.018744527
ENSMUSG00000049871	108.0922325	-1.18792045	0.354853896	-3.347632538	0.00081505	0.040122887
ENSMUSG00000025888	2987.052833	-1.187223967	0.323090885	-3.674582051	0.000238239	0.018626692
ENSMUSG00000022901	1097.099171	-1.18680554	0.27528465	-4.311194024	1.62E-05	0.003357201
ENSMUSG00000026946	1205.449904	-1.180496929	0.32072247	-3.680742821	0.000232555	0.018359105
ENSMUSG00000018986	259.7588669	-1.174341551	0.349675368	-3.358376536	0.000784017	0.039217729
ENSMUSG00000021044	492.6941229	-1.172065258	0.267283886	-4.385095099	1.16E-05	0.002630879
ENSMUSG00000040584	209.8967949	-1.168021736	0.302373972	-3.862838218	0.000112077	0.011404973
ENSMUSG00000052920	193.3262336	-1.160438151	0.317289979	-3.657342582	0.000254844	0.01881798
ENSMUSG00000019794	416.6720185	-1.158750376	0.33780259	-3.430259003	0.000603005	0.032618383
ENSMUSG00000016496	3765.252695	-1.143350017	0.276727713	-4.131678765	3.60E-05	0.005404175
ENSMUSG00000036890	219.9650858	-1.14177245	0.310228221	-3.680427421	0.000232843	0.018359105
ENSMUSG00000029780	205.8025908	-1.135164924	0.325832067	-3.483895658	0.000494172	0.029099856
ENSMUSG00000003208	190.0434793	-1.133706436	0.31967243	-3.546462969	0.00039044	0.025339773
ENSMUSG00000063800	497.0545246	-1.129231256	0.323578933	-3.489816986	0.000483351	0.028738541
ENSMUSG00000106734	434.1288001	-1.128685733	0.334417953	-3.375075182	0.000737955	0.037762916
ENSMUSG00000105504	1022.138923	-1.111177289	0.338927597	-3.278509336	0.001043569	0.04713285
ENSMUSG00000030876	384.1726748	-1.106661912	0.30179046	-3.666987728	0.000245425	0.018744527
ENSMUSG00000028270	6699.543535	-1.104542981	0.320968902	-3.441277251	0.000578975	0.031686959
ENSMUSG00000083161	130.7685655	-1.091383425	0.331359485	-3.293653794	0.000988942	0.046005593
ENSMUSG00000037997	402.1925124	-1.088780129	0.331985685	-3.279599624	0.001039545	0.04713285
ENSMUSG00000029486	521.6336851	-1.082955451	0.294208821	-3.680907483	0.000232405	0.018359105
ENSMUSG00000027018	740.3823963	-1.082849269	0.310212985	-3.490663904	0.000481822	0.028738541

ENSMUSG00000020290	1337.948514	-1.081261059	0.306261491	-3.530515885	0.00041475	0.025776094
ENSMUSG00000040253	2576.560947	-1.080432382	0.320290411	-3.373289822	0.000742757	0.037762916
ENSMUSG00000037731	362.2574435	-1.072247278	0.288848892	-3.712139146	0.000205515	0.017226226
ENSMUSG00000032407	665.8011878	-1.053703304	0.282263196	-3.733052414	0.000189173	0.016147409
ENSMUSG00000017652	3991.105128	-1.051701453	0.251119135	-4.188057801	2.81E-05	0.004754602
ENSMUSG00000021282	1462.245736	-1.047007454	0.283173849	-3.697401634	0.000217818	0.017715493
ENSMUSG00000038774	1191.878379	-1.040390386	0.293758858	-3.541647706	0.000397636	0.025339773
ENSMUSG00000025757	1382.547506	-1.038094261	0.280864846	-3.696063336	0.000218968	0.017715493
ENSMUSG00000059089	1896.017781	-1.0360684	0.300211078	-3.451133143	0.000558238	0.031288237
ENSMUSG00000025059	1222.576392	-1.025024488	0.267910991	-3.825988934	0.000130248	0.012891795
ENSMUSG00000020089	804.2540069	-1.01966484	0.268903239	-3.791939598	0.000149475	0.013739245
ENSMUSG00000055202	390.2755106	-1.0137141	0.301118212	-3.366498805	0.000761289	0.038335504
ENSMUSG00000031382	547.2964187	-1.010682235	0.261745092	-3.861322588	0.000112775	0.011404973
ENSMUSG00000025408	3638.625782	-1.010260265	0.244891948	-4.125330677	3.70E-05	0.005467244
ENSMUSG00000030084	911.8767558	1.004444018	0.250386782	4.011569665	6.03E-05	0.007482447
ENSMUSG00000060143	888.5320097	1.016751362	0.309270525	3.287579249	0.001010527	0.046315011
ENSMUSG00000063234	681.06567	1.019962484	0.255389435	3.993753634	6.50E-05	0.007851761
ENSMUSG00000030802	232.0892233	1.020236846	0.29971867	3.403981633	0.000664112	0.035107385
ENSMUSG00000031659	844.4036599	1.021003849	0.275496409	3.70605139	0.000210516	0.017487832
ENSMUSG00000001020	740.5787285	1.024316164	0.282486389	3.626072627	0.000287764	0.020437866
ENSMUSG00000037936	321.05326	1.024840207	0.289108137	3.544833495	0.000392861	0.025339773
ENSMUSG00000038459	589.3879999	1.024960287	0.271916094	3.769399122	0.000163641	0.014639578
ENSMUSG00000003380	1004.732644	1.030924463	0.271831095	3.792518527	0.000149127	0.013739245
ENSMUSG00000058135	596.5118048	1.032088518	0.291376485	3.54211328	0.000396935	0.025339773
ENSMUSG00000091498	218.0733511	1.032176984	0.313548523	3.291921049	0.000995055	0.046052656
ENSMUSG00000044080	227.1296573	1.033241417	0.29976608	3.446825661	0.000567214	0.031412878
ENSMUSG00000031722	615.5477545	1.045397386	0.259062357	4.035311793	5.45E-05	0.00694994

ENSMUSG00000021596	212.2590238	1.053425899	0.311770313	3.378852497	0.000727891	0.037623852
ENSMUSG00000024621	3006.737841	1.063744398	0.236666136	4.494704727	6.97E-06	0.001800493
ENSMUSG00000044716	225.3388219	1.082355762	0.295361024	3.664517912	0.000247805	0.018744527
ENSMUSG00000065767	321.9079926	1.086834175	0.312464592	3.478263465	0.000504674	0.029346771
ENSMUSG00000051682	138.3789985	1.090794124	0.331491139	3.290567966	0.000999853	0.046052656
ENSMUSG00000053113	430.7562763	1.093146316	0.288425905	3.790042069	0.000150622	0.013739245
ENSMUSG00000028969	180.9113083	1.098895394	0.317613935	3.459846286	0.000540484	0.030476736
ENSMUSG00000057322	602.7972013	1.101995447	0.333734528	3.302012095	0.000959939	0.045801412
ENSMUSG00000005413	7979.751405	1.108514436	0.244486631	4.534049285	5.79E-06	0.001538178
ENSMUSG00000000489	252.9704291	1.113375421	0.330044369	3.373411355	0.000742429	0.037762916
ENSMUSG00000038803	531.1886664	1.118049975	0.318516015	3.510184488	0.000447796	0.027409822
ENSMUSG00000060126	315.1381483	1.126022771	0.341861254	3.293800507	0.000988426	0.046005593
ENSMUSG00000036775	304.8542977	1.126167417	0.307155081	3.666445675	0.000245945	0.018744527
ENSMUSG00000037089	317.8417969	1.134520534	0.297392292	3.814895556	0.000136241	0.013342969
ENSMUSG00000027398	6635.223668	1.1480283	0.270734809	4.240416315	2.23E-05	0.004151549
ENSMUSG00000095969	260.3139287	1.152756688	0.3232487	3.566160324	0.00036225	0.023903349
ENSMUSG00000017002	1304.768555	1.161749855	0.271287249	4.282360708	1.85E-05	0.003668879
ENSMUSG00000049686	195.072437	1.171947721	0.32000883	3.662235579	0.000250024	0.018759848
ENSMUSG00000054452	394.2891567	1.180228177	0.270850947	4.357482185	1.32E-05	0.002914527
ENSMUSG00000069792	760.2006766	1.185231216	0.253696548	4.671846051	2.99E-06	0.000991888
ENSMUSG00000043207	143.3576274	1.188020301	0.336029092	3.535468594	0.000407053	0.025741228
ENSMUSG00000000318	115.9664677	1.188347239	0.34323269	3.462220449	0.000535738	0.03039334
ENSMUSG00000055302	610.6615696	1.189574902	0.289830074	4.104387399	4.05E-05	0.005714738
ENSMUSG00000027399	2489.18968	1.215776718	0.291631009	4.168886985	3.06E-05	0.004910117
ENSMUSG00000023349	142.7760995	1.216980359	0.339271261	3.587042289	0.00033445	0.022567742
ENSMUSG00000060803	332.967851	1.218283286	0.288529356	4.222389366	2.42E-05	0.004325037
ENSMUSG00000020766	690.6683896	1.224065383	0.327258502	3.740362361	0.000183755	0.016128848

ENSMUSG00000050824	191.9032757	1.227352667	0.310396261	3.954147719	7.68E-05	0.008609893
ENSMUSG00000093956	2571.802564	1.247875587	0.292683171	4.26357137	2.01E-05	0.00389964
ENSMUSG00000046982	151.5829173	1.252427022	0.321227015	3.898884482	9.66E-05	0.010102352
ENSMUSG00000038508	1525.089896	1.262967735	0.301842303	4.18419725	2.86E-05	0.004754602
ENSMUSG00000057666	1129.214062	1.266884306	0.305577356	4.145871017	3.39E-05	0.005183147
ENSMUSG00000024247	123.5833644	1.274509453	0.342710716	3.718907504	0.000200086	0.016923663
ENSMUSG00000060063	1076.639944	1.275159588	0.260120073	4.902196016	9.48E-07	0.000440876
ENSMUSG00000025934	133.6150302	1.276178145	0.35171521	3.628441729	0.000285137	0.020407042
ENSMUSG00000026821	195.8017388	1.280406026	0.338889328	3.77824239	0.000157939	0.014266656
ENSMUSG00000094050	151.91199	1.28249569	0.31895243	4.02096228	5.80E-05	0.007287407
ENSMUSG00000059498	1625.910389	1.286839625	0.242905114	5.297704959	1.17E-07	8.39E-05
ENSMUSG00000063354	115.8933184	1.306552031	0.35823823	3.647159684	0.000265155	0.019343251
ENSMUSG00000037656	96.34440162	1.313890421	0.378175838	3.474284413	0.000512218	0.029417743
ENSMUSG00000050708	4264.690978	1.314058557	0.279260228	4.705498395	2.53E-06	0.000991888
ENSMUSG00000024669	90.92302213	1.326608545	0.354548906	3.741679987	0.000182794	0.016128848
ENSMUSG00000078484	76.10056501	1.341447883	0.386810774	3.467969282	0.000524407	0.029933036
ENSMUSG00000081400	1812.907996	1.345959829	0.31802181	4.232287815	2.31E-05	0.004220114
ENSMUSG00000034445	138.5645982	1.346515087	0.33024244	4.077353254	4.56E-05	0.006142158
ENSMUSG00000082806	639.8285461	1.355860399	0.357743137	3.790038878	0.000150624	0.013739245
ENSMUSG00000017390	140.0247652	1.362765693	0.341018905	3.996158789	6.44E-05	0.007851761
ENSMUSG00000031765	826.4154082	1.369688183	0.289020459	4.739069984	2.15E-06	0.000907992
ENSMUSG00000089715	77.12586391	1.37738729	0.390195055	3.529996786	0.000415565	0.025776094
ENSMUSG00000044303	686.3202313	1.396868171	0.281997663	4.953474281	7.29E-07	0.000384111
ENSMUSG00000027546	52.76309568	1.403175495	0.391408539	3.584938386	0.000337158	0.022567742
ENSMUSG00000046223	677.5048773	1.43666259	0.302105548	4.755498867	1.98E-06	0.000877044
ENSMUSG00000031749	165.0887641	1.447714995	0.324348316	4.463457717	8.06E-06	0.002027958
ENSMUSG00000021384	119.6880011	1.453932391	0.345521011	4.207942051	2.58E-05	0.004523972

ENSMUSG00000038776	157.9846457	1.463122027	0.320490178	4.56526324	4.99E-06	0.001365137
ENSMUSG00000070283	60.83404915	1.482541006	0.390416602	3.797330852	0.000146263	0.013739245
ENSMUSG00000037706	725.809802	1.498610915	0.261615765	5.728289792	1.01E-08	9.44E-06
ENSMUSG00000027864	234.4244075	1.564397253	0.315070136	4.96523495	6.86E-07	0.000384111
ENSMUSG00000024892	804.4959704	1.570664055	0.260222236	6.035856433	1.58E-09	2.10E-06
ENSMUSG00000058427	6462.18354	1.642248652	0.249884492	6.572031085	4.96E-11	2.30E-07
ENSMUSG00000074604	42.27489479	1.644720703	0.406709544	4.043968794	5.26E-05	0.006791134
ENSMUSG00000081534	1755.611743	1.712799106	0.267808412	6.395613535	1.60E-10	3.72E-07
ENSMUSG00000038067	1503.757823	1.778121886	0.289316252	6.145945384	7.95E-10	1.23E-06
ENSMUSG00000031762	79.1129036	1.911681429	0.413039137	4.628330003	3.69E-06	0.001085865
ENSMUSG00000049723	620.3971016	2.582604332	0.329528074	7.837281659	4.60E-15	4.28E-11

Supplementary Table S4. Canonical pathways respond to LPS stimulation (LPS vs. UNT).

Ingenuity Canonical Pathways	-log(p-value)	Ratio	z-score	Molecules
Death Receptor Signaling	1.22E01	4.22E-01	3.244	RELA,Naip1 (includes others),NFKBIE,TNFSF10,PARP12,NFKB1,PARP9,FAS,BCL2,TANK,IKBKG,NFKBIA,TIPARP,BID,TNFSF15,PARP11,TNFRSF1B,NFKBIB,BIRC3,PARP14,NAIP,MAP3K14,PARP16,CASP3,PARP10,ZC3HAV1,APAF1,PARP8,IKBKE,TBK1,NFKB2,PARP1,DAXX,TRAF2,HSPB7,CFLAR,TNF,CASP7
Toll-like Receptor Signaling	1.15E01	4.57E-01	1.964	MAP2K6,RELA,IL1A,TICAM2,IL1RL1,TLR8,TNFAIP3,Ubb,NFKB1,IKBKG,IL36G,NFKBIA,TICAM1,TLR1,TLR3,TRAF1,MAP3K14,NFKB2,IRAK3,TLR9,TLR2,LY96,IL1RN,IL12B,TLR6,CD14,IL1B,MAP2K3,EIF2AK2,UBC,TNF,IRAK2
IL-10 Signaling	1.1E01	4.62E-01	NA	MAP2K6,RELA,SOCS3,IL1A,IL1RL1,NFKBIE,IL6,NFKB1,HMOX1,IKBKG,IL36G,NFKBIA,IL1RL2,NFKBIB,MAP3K14,IL4R,FCGR2A,BLVRA,TYK2,BLVRB,IKBKE,IL1R1,STAT3,NFKB2,IL18RAP,IL1RN,CD14,IL1B,MAP2K3,TNF
TNFR2 Signaling	1.07E01	6.55E-01	2.000	NAIP,MAP3K14,RELA,Naip1 (includes others),NFKBIE,TNFAIP3,TBK1,IKBKE,NFKB2,NFKB1,TANK,IKBKG,TRAF2,NFKBIA,NFKBIB,TNFRSF1B,TNF,BIRC3,TRAF1

Role of Pattern Recognition Receptors in Recognition of Bacteria and Viruses	1.02E01	3.53E-01	3.402	RELA,IL1A,PIK3R5,Oas1b,TLR8,C1QC,C1QA,CCL5,IL6,NFKB1,RNASEL,IFIH1,TICAM1,TLR1,CASP1,OSM,NOD1,TLR3,RIPK2,OAS1,C5AR1,NLRP3,C3,OAS2,NFKB2,OAS3,TLR9,TLR2,NOD2,IRF7,CLEC7A,IL12B,SYK,DDX58,TLR6,Oas1d (includes others),IL1B,PRKCH,EIF2AK2,TNF,C3AR1
Role of PKR in Interferon Induction and Antiviral Response	9.46E00	5.38E-01	NA	MAP2K6,RELA,CASP3,NFKBIE,APAF1,IKBKE,NFKB2,NFKB1,RNASEL,FCGR1A,IRF1,IKBKG,TRAF2,NFKBIA,BID,MAP2K3,EIF2AK2,TLR3,NFKBIB,STAT1,TNF
IL-6 Signaling	9.35E00	3.45E-01	2.596	MAP2K6,SOCS3,SOCS1,RELA,IL1A,IL1RL1,NFKBIE,PIK3R5,SRF,KRAS,JAK2,IL6,NFKB1,VEGFA,IKBKG,IL36G,NFKBIA,IL1RL2,NFKBIB,TNFRSF1B,MAPKAPK2,MAP2K1,MCL1,MAP3K14,ABCB1,IL6R,IKBKE,STAT3,IL1R1,CEBPB,NFKB2,IL18RAP,TRAF2,IL1RN,CD14,IL1B,HSPB7,MAP2K3,TNF
Role of Macrophages, Fibroblasts and Endothelial Cells in Rheumatoid Arthritis	9.16E00	2.52E-01	NA	SOCS1,SOCS3,PLCB2,TLR8,MMP13,KRAS,IL6,FCGR1A,CCND1,MYC,VEGFA,PLCD3,IKBKG,IL1RL2,TLR1,OSM,NFKBIB,ADAMTS4,MAP3K14,IL6R,STAT3,PPP3CC,TLR9,IL7,TLR2,IL1RN,PRKCH,FZD5,MAP2K3,TNF,MAP2K6,RELA,IL1A,ICAM1,IL1RL1,NFKBIE,LRP6,PIK3R5,JAK2,CCL5,NFKB1,NFATC1,IL36G,NFKBIA,CCL2,SMO,CEBPA,TLR3,MAPKAPK2,TNFRSF1B,NOS2,MAP2K1,FCGR3A/FCGR3B,TRAF1,SRC,VCAM1,LRP5,C5AR1,DAAM1,IL15,IKBKE,IL1R1,CEBPB,IRAK3,IL18RAP,TRAF2,FZD4,CSF1,TLR6,NFATC2,IL1B,IRAK2
TREM1 Signaling	8.71E00	4.06E-01	NA	RELA,Naip1 (includes others),ICAM1,IL1RL1,TLR8,IL6,JAK2,NFKB1,CXCL3,CCL2,TLR1,CASP1,NOD1,TLR3,NLRP3,NLRP10,ITGA5,STAT3,NFKB2,TLR9,TLR2,NLRC5,NOD2,CD40,TLR6,IL1B,TNF,ITGAX
Activation of IRF by Cytosolic Pattern Recognition Receptors	8.33E00	4.51E-01	1.877	DHX58,RELA,NFKBIE,ZBP1,TBK1,IKBKE,IL6,NFKB2,NFKB1,ADAR,ISG15,IFIH1,TANK,IKBKG,IRF7,NFKBIA,CD40,DDX58,STAT2,STAT1,NFKBIB,IFIT2,TNF
TWEAK Signaling	8.03E00	5.29E-01	0.471	NAIP,MAP3K14,RELA,Naip1 (includes others),CASP3,NFKBIE,APAF1,IKBKE,NFKB2,NFKB1,TRAF2,IKBKG,NFKBIA,BID,NFKBIB,BIRC3,CASP7,TRAF1
Interferon Signaling	7.93E00	5.48E-01	2.840	RELA,IFIT3,SOCS1,OAS1,PTPN2,TYK2,IFI35,JAK2,PSMB8,TAP1,IRF1,ISG15,BCL2,IFIT1,IFITM3,STAT2,STAT1
Communication between Innate and Adaptive Immune Cells	7.34E00	3.77E-01	NA	B2M,IL1A,HLA-A,IL15,TLR8,CCL5,IL6,TLR9,Ccl9,TLR2,HLA-G,CXCL10,CD28,IL36G,CCL4,CD40,IL1RN,IL12B,CCL3L3,TLR6,TLR1,IL1B,TNFRSF13B,TLR3,TNF,HLA-E

Small Cell Lung Cancer Signaling	7.05E00	3.66E-01	NA	RELA,SUV39H1,NFKBIE,PIK3R5,NFKB1,CCND1,CDKN2B,BCL2,MYC,IKBKG,NFKBIA,BID,NFKBIB,TRAF1,TFDP1,APAF1,IKBKE,NFKB2,SKP2,TRAF2,CCNE1,E2F1,CKS1B,CDKN1B,PTGS2,RXRA
Molecular Mechanisms of Cancer	6.93E00	2.18E-01	NA	PRKACB,RAPGEF1,Naip1 (includes others),PLCB2,SUV39H1,SMAD3,CDKN2C,ARHGEF1,KRAS,CCND1,MYC,TGFBR2,CCND3,RHOB,GNA13,NFKBIB,BIRC3,E2F2,CDC25A,ARHGEF4,CASP3,TFDP1,CDK7,TYK2,PTCH1,ITGA5,RHOJ,AURKA,NFKB2,Aph1c,DAXX,RHOQ,CCND2,E2F1,PRKACA,PRKCH,FZD5,MAP2K3,CFLAR,RAP1B,MAP2K6,RELA,NFKBIE,ARHGEF7,LRP6,PIK3R5,JAK2,NFKB1,CDKN2B,FAS,BCL2,CHEK1,CDC25B,NFKBIA,FANCD2,CDK5,E2F5,RHOU,SMO,BID,MAP2K1,SRC,NAIP,LRP5,ARHGEF12,RHOC,GNA12,PRKAR2A,APAF1,MDM2,CCNE1,FZD4,CDKN1A,PRKAG2,CDKN1B,ADCY7,CASP7,BCL2L11
Induction of Apoptosis by HIV1	6.89E00	3.9E-01	1.460	NAIP,RELA,MAP3K14,Naip1 (includes others),CASP3,CXCR4,NFKBIE,APAF1,IKBKE,NFKB2,NFKB1,FAS,BCL2,DAXX,IKBKG,TRAF2,NFKBIA,BID,NFKBIB,TNFRSF1B,TNF,BIRC3,TRAF1
TNFR1 Signaling	6.81E00	4.26E-01	2.065	NAIP,RELA,MAP3K14,Naip1 (includes others),CASP3,NFKBIE,APAF1,TNFAIP3,IKBKE,NFKB2,NFKB1,TANK,IKBKG,TRAF2,NFKBIA,BID,NFKBIB,TNF,BIRC3,CASP7
Type I Diabetes Mellitus Signaling	6.8E00	3.17E-01	NA	MAP2K6,SOCS1,SOCS3,RELA,HLA-A,NFKBIE,JAK2,NFKB1,FAS,BCL2,HLA-G,CD28,IKBKG,NFKBIA,BID,NOS2,STAT1,TNFRSF1B,NFKBIB,MAP3K14,CASP3,APAF1,IKBKE,NFKB2,IL1R1,IRF1,TRAF2,IL12B,IL1B,MAP2K3,TNF,HLA-E
Role of RIG1-like Receptors in Antiviral Innate Immunity	6.75E00	5E-01	3.051	DHX58,RELA,NFKBIE,TBK1,IKBKE,NFKB2,NFKB1,TANK,IFIH1,TRAF2,IRF7,IKBKG,NFKBIA,DDX58,NFKBIB,TRIM25
Mismatch Repair in Eukaryotes	6.71E00	6.88E-01	NA	MSH2,RFC4,MSH3,MSH6,RFC2,SLC19A1,RFC1,RFC5,POLD1,MLH1,EXO1
Role of BRCA1 in DNA Damage Response	6.71E00	3.46E-01	-1.342	FANCM,BARD1,MLH1,CHEK1,FANCE,RAD51,GADD45A,FANCD2,RFC2,E2F5,SLC19A1,STAT1,BRIP1,E2F2,RBL2,FAAP100,TOPBP1,MDC1,PLK1,RFC1,RFC5,MSH2,RFC4,CDKN1A,E2F1,MSH6,BRCA2
NF-κB Signaling	6.69E00	2.68E-01	1.372	MAP2K6,PRKACB,AZI2,RELA,IL1A,NFKBIE,PIK3R5,TLR8,TNFAIP3,KRAS,NFKB1,TGFBR2,TANK,TNIP1,IL36G,IKBKG,NFKBIA,TLR1,IGF1R,TLR3,TNFRSF1B,NFKBIB,MAP3K14,RELB,TBK1,NFKB2,IL1R1,MALT1,IRAK3,TLR9,TLR2,TRAF2,CD40,CARD11,IL1RN,BCL10,PELI1,TLR6,PRKACA,IL1B,MAP3K8,EIF2AK2,KDR,TNF

Aryl Hydrocarbon Receptor Signaling	6.62E00	2.86E-01	1.000	RELA,IL1A,NFIX,GSTM5,POLA1,IL6,NFKB1,CCND1,FAS,CHEK1,MYC,CCNA2,HSP90B1,CCND3,AHR,GSTM1,SRC,RBL2,TFDP1,NQO1,APAF1,MDM2,NFKB2,CCNE1,CCND2,MGST2,CDKN1A,E2F1,ALDH1A2,HSP90AA1,IL1B,HSPB7,NCOR2,CDKN1B,RXRA,TNF,MGST3,MCM7
Hepatic Fibrosis / Hepatic Stellate Cell Activation	6.6E00	2.61E-01	NA	IGFBP4,MYH10,RELA,IL1A,ICAM1,IL1RL1,SMAD3,KLF6,CXCR3,MMP13,CCL5,IL6,COL4A2,NFKB1,FAS,BCL2,TGFBR2,VEGFA,CXCL3,COL6A1,EDN1,IGF1,IL1RL2,CCL2,IGF1R,SERPINE1,TNFRSF1B,STAT1,COL18A1,TIMP2,IL4R,VCAM1,IL6R,BAMBI,COL20A1,IL1R1,NFKB2,IL18RAP,MET,LY96,CD40,CSF1,CD14,IL1B,KDR,TNF
Cell Cycle: G1/S Checkpoint Regulation	6.58E00	3.77E-01	1.342	HDAC9,RBL2,HDAC4,TFDP1,SUV39H1,SMAD3,CUL1,CDKN2C,MDM2,CCND1,CDKN2B,HDAC5,SKP2,MYC,CCNE1,CCND2,CCND3,CDKN1A,E2F1,E2F5,CDKN1B,E2F2,CDC25A
Cyclins and Cell Cycle Regulation	6.5E00	3.47E-01	-2.236	SUV39H1,CUL1,CDKN2C,CCNB2,CDKN2B,CCND1,CCNA2,CCND3,E2F5,E2F2,CDC25A,HDAC9,HDAC4,TFDP1,WEE1,CDK7,CDK1,CCNB1,HDAC5,SKP2,CCNE1,CCND2,PPP2R4,CDKN1A,E2F1,CDKN1B
Cell Cycle: G2/M DNA Damage Checkpoint Regulation	6.45E00	4.08E-01	2.236	CDK7,WEE1,CUL1,CCNB2,MDM2,PLK1,AURKA,CDK1,SKP2,CHEK1,CCNB1,CDC25B,TOP2B,KAT2B,GADD45A,CDKN1A,CKS1B,TOP2A,BORA,SFN
CD40 Signaling	6.14E00	3.59E-01	1.091	MAP2K6,RELA,MAP3K14,ICAM1,NFKBIE,PTGS1,PIK3R5,TNFAIP3,IKBKE,NFKB2,STAT3,NFKB1,TANK,IKBKG,TRAF2,NFKBIA,CD40,MAP2K3,PTGS2,MAPKAPK2,NFKBIB,MAP2K1,TRAF1
Estrogen-mediated S-phase Entry	6.11E00	5.42E-01	-2.309	TFDP1,CCND1,CDK1,SKP2,MYC,CCNA2,CCNE1,E2F1,CDKN1A,E2F5,CDKN1B,E2F2,CDC25A
Pancreatic Adenocarcinoma Signaling	5.96E00	2.98E-01	1.606	RELA,SMAD3,SUV39H1,PIK3R5,KRAS,JAK2,NFKB1,CDKN2B,CCND1,BCL2,RAD51,TGFBR2,VEGFA,HMOX1,E2F5,STAT1,MAP2K1,E2F2,TFDP1,TYK2,MDM2,STAT3,NFKB2,BIRC5,PLD4,CCNE1,CDKN1A,E2F1,BRCA2,PTGS2,CDKN1B
NRF2-mediated Oxidative Stress Response	5.8E00	2.53E-01	2.138	AKR7A2,MAP2K6,GSTM5,DNAJB4,PRDX1,MAF,PIK3R5,GCLC,KRAS,MAFG,HMOX1,SOD2,SCARB1,ABCC1,DNAJA2,JUND,GCLM,DNAJB1,FKBP5,MAP2K1,GSTM1,DNAJB12,DNAJC9,NQO1,DNAJC19,DNAJC1,JUNB,DNAJB9,MAFK,MAFF,DNAJB14,TXNRD1,GSR,ERP29,MGST2,CAT,MAP2K3,PRKCH,SQSTM1,HACD3,MGST3,EPHX1,FTH1
Apoptosis Signaling	5.54E00	3.07E-01	0.392	RELA,Naip1 (includes others),NFKBIE,KRAS,NFKB1,FAS,BCL2,IKBKG,NFKBIA,BID,NFKBIB,TNFRSF1B,MAP2K1,BIRC3,MCL1,NAIP,MAP3K14,CASP3,APAF1,IKBKE,NFKB2,CDK1,PARP1,BCL2A1,TNF,BCL2L11,CASP7

Dendritic Cell Maturation	5.41E00	2.55E-01	4.226	B2M,RELA,IL1A,PLCB2,ICAM1,HLA-A,NFKBIE,PIK3R5,JAK2,IL6,NFKB1,FCGR1A,PLCD3,IL36G,IKBKG,NFKBIA,IL1RL2,LY75,TLR3,NFKBIB,COL18A1,STAT1,TNFRSF1B,FCGR3A/FCGR3B,MAP3K14,FCGR2A,RELB,IL15,IKBKE,NFKB2,TLR9,TLR2,CD40,IL12B,IL1RN,TREM2,IL1B,STAT2,TNF
Hereditary Breast Cancer Signaling	5.38E00	2.7E-01	NA	FANCM,GADD45B,GADD45G,BARD1,PIK3R5,Ubb,KRAS,CCND1,MLH1,CHEK1,RAD51,FANCE,FANCD2,GADD45A,RFC2,SLC19A1,HDAC9,HDAC4,FAAP100,WEE1,RFC1,RFC5,CDK1,HDAC5,CCNB1,RFC4,MSH2,H2AFX,CDKN1A,E2F1,MSH6,BRCA2,UBC,SFN
iNOS Signaling	5.35E00	3.95E-01	4.000	RELA,NFKBIE,TYK2,IKBKE,JAK2,NFKB2,IRAK3,NFKB1,IRF1,LY96,IKBKG,NFKBIA,CD14,NOS2,NFKBIB,STAT1,IRAK2
PPAR Signaling	5.23E00	2.97E-01	-3.272	RELA,IL1A,IL1RL1,NFKBIE,KRAS,NFKB1,IKBKG,HSP90B1,IL36G,NFKBIA,IL1RL2,TNFRSF1B,NFKBIB,MAP2K1,MAP3K14,IKBKE,IL1R1,NFKB2,IL18RAP,TRAF2,IL1RN,IL1B,HSP90AA1,PTGS2,NCOR2,RXRA,TNF
Colorectal Cancer Metastasis Signaling	5.17E00	2.24E-01	1.640	PRKACB,MSH3,SMAD3,TLR8,MMP13,KRAS,IL6,CCND1,MYC,VEGFA,TGFBR2,RHOB,TLR1,GNG12,CASP3,TYK2,IL6R,RHOJ,NFKB2,STAT3,TLR9,TLR2,RHOQ,PRKACA,FZD5,TNF,RELA,LRP6,PIK3R5,JAK2,NFKB1,GNG7,MLH1,RHOU,SMO,TLR3,STAT1,NOS2,MAP2K1,PTGER4,SRC,LRP5,RHOC,PRKAR2A,BIRC5,FZD4,MSH2,TLR6,PRKAG2,MSH6,PTGS2,ADCY7
Retinoic acid Mediated Apoptosis Signaling	5.03E00	3.78E-01	3.000	PARP16,CASP3,PARP10,ZC3HAV1,APAF1,TNFSF10,PARP8,PARP12,PARP9,IRF1,PARP1,TIPARP,BID,PARP11,CFLAR,RXRA,PARP14
Hepatic Cholestasis	5.01E00	2.48E-01	NA	PRKACB,RELA,IL1A,IL1RL1,NFKBIE,IL6,NFKB1,IKBKG,IL36G,NFKBIA,IL1RL2,ABCC1,OSM,TNFRSF1B,NFKBIB,ABCB1,MAP3K14,CYP27A1,PRKAR2A,IKBKE,IRAK3,IL1R1,NFKB2,IL18RAP,TRAF2,LY96,IL12B,IL1RN,PRKACA,PRKAG2,CD14,IL1B,PRKCH,RXRA,ABCC3,ADCY7,TNF,IRAK2
Crosstalk between Dendritic Cells and Natural Killer Cells	4.93E00	3.19E-01	NA	RELA,IL15RA,HLA-A,CD69,IL15,TNFSF10,IL6,NFKB2,TLR9,NFKB1,FAS,HLA-G,CD28,TLN2,CD40,IL12B,TREM2,TLR3,TNFRSF1B,TNF,HLA-E,PVRL2
LXR/RXR Activation	4.92E00	2.73E-01	-3.138	RELA,APOE,IL1A,IL1RL1,IL6,NFKB1,LYZ,IL36G,IL1RL2,CCL2,FASN,LPL,TLR3,NOS2,TNFRSF1B,C3,SERPINF1,IL1R1,NFKB2,IL18RAP,LY96,LDLR,IL1RN,CD14,IL1B,PTGS2,NCOR2,RXRA,TNF,HADH
p38 MAPK Signaling	4.84E00	2.7E-01	1.671	MAP2K6,IL1A,DDIT3,IL1RL1,SRF,FAS,CDC25B,TGFBR2,MYC,IL36G,IL1RL2,DUSP10,PLA2G5,EEF2K,MAPKAPK2,STAT1,TNFRSF1B,IL1R1,IRAK3,IL18RAP,DAXX,TRAF2,IL1RN,DUSP1,IL1B,MAP2K3,MEF2C,HSPB7,TNF,IRAK2

CD27 Signaling in Lymphocytes	4.82E00	3.53E-01	0.258	MAP2K6,RELA,MAP3K14,CASP3,NFKBIE,APAF1,IKBKE,NFKB2,NFKB1,MAP3K12,TRAF2,IKBKG,NFKBIA,BID,MAP2K3,MAP3K8,NFKBIB,MAP2K1
PEDF Signaling	4.81E00	3.14E-01	0.688	ARHGAP22,RELA,GDNF,NFKBIE,SRF,SERPINF1,PIK3R5,IKBKE,KRAS,NFKB2,ZEB1,NFKB1,FAS,BCL2,TCF12,IKBKG,SOD2,NFKBIA,DOCK3,CFLAR,NFKBIB,CASP7
Glucocorticoid Receptor Signaling	4.77E00	2.12E-01	NA	PRKACB,SGK1,SMAD3,SLPI,KRAS,IL6,HSPA5,FCGR1A,TGFBR2,CXCL3,IKBKG,NFKBIB,SERPINE1,MAP3K14,CDK7,STAT3,PPP3CC,IL1RN,DUSP1,PRKACA,HSP90AA1,TNF,RELA,ICAM1,NFKBIE,PIK3R5,JAK2,CCL5,NFKB1,NFATC1,BCL2,HSP90B1,NFKBIA,KAT2B,PCK2,CCL2,CEBPA,STAT1,FKBP5,NOS2,MAP2K1,ADRB2,VCAM1,IKBKE,CEBPB,TAF7L,TRAF2,GTF2H4,CDKN1A,FKBP4,PRKAG2,NFATC2,IL1B,PTGS2,NCOR2,PLAU
Cell Cycle Control of Chromosomal Replication	4.75E00	4.62E-01	NA	MCM5,MCM3,MCM6,CDC45,MCM2,CDK5,CDK7,CDC6,CDC7,MCM4,MCM7,RPA2
4-1BB Signaling in T Lymphocytes	4.73E00	4.33E-01	2.530	RELA,MAP3K14,IKBKG,TRAF2,TNFRSF9,NFKBIA,NFKBIE,IKBKE,NFKB2,NFKBIB,NFKB1,MAP2K1,TRAF1
Acute Phase Response Signaling	4.73E00	2.42E-01	2.874	MAP2K6,SOCS3,SOCS1,RELA,IL1A,Saa3,NFKBIE,KRAS,CP,JAK2,IL6,NFKB1,HMOX1,IKBKG,IL36G,NFKBIA,SOD2,CFB,OSM,NFKBIB,SERPINE1,TNFRSF1B,MAP2K1,MAP3K14,C3,IL6R,SERPINF1,IKBKE,STAT3,IL1R1,CEBPB,NFKB2,TRAF2,HP,IL1RN,IL1B,MAP2K3,TNF
Altered T Cell and B Cell Signaling in Rheumatoid Arthritis	4.71E00	2.96E-01	NA	RELA,MAP3K14,IL1A,RELB,IL15,TLR8,IL6,NFKB2,NFKB1,TLR9,FAS,TLR2,CD28,IL36G,CD40,IL12B,IL1RN,CSF1,TLR1,TLR6,IL1B,TNFRSF13B,TLR3,TNF
GADD45 Signaling	4.67E00	5.26E-01	NA	CCNE1,CCND2,GADD45B,GADD45A,CCND3,GADD45G,CDKN1A,CCND1,CDK1,CCNB1
Granulocyte Adhesion and Diapedesis	4.61E00	2.42E-01	NA	IL1A,ICAM1,IL1RL1,MMP13,CCL24,CCL5,SDC4,CXCL10,CXCL3,IL36G,IL1RL2,CCL2,CCL3L3,Ccl8,Ccl2,Cxcl3,TNFRSF1B,VCAM1,Cxcl11,C5AR1,CXCR4,FPR2,ITGA6,ITGA5,IL1R1,CSF3,IL18RAP,SELPLG,Ccl9,CCL4,ITGAM,IL1RN,IL1B,CXCL2,TNF,Ccl7,MSN
Antigen Presentation Pathway	4.54E00	4.44E-01	NA	HLA-G,B2M,PSMB9,NLRC5,HLA-A,PSMB8,CD74,TAP1,TAP2,TAPBP,HLA-E,MR1
IL-17A Signaling in Fibroblasts	4.37E00	4.06E-01	NA	RELA,NFKBIE,LCN2,IKBKE,CEBPB,NFKB2,IL6,NFKBIZ,NFKB1,IKBKG,NFKBIA,CCL2,NFKBIB

Role of Hypercytokinemia/hyperchemokinemia in the Pathogenesis of Influenza	4.35E00	4.29E-01	NA	CXCL10,IL36G,IL1A,CCL4,CCL2,IL1RN,IL12B,IL15,IL1B,CCL5,IL6,TNF
Role of IL-17A in Arthritis	4.34E00	3.4E-01	NA	MAP2K6,RELA,NFKBIE,PIK3R5,MMP13,CCL5,NFKB2,NFKB1,CXCL3,NFKBIA,CCL2,MAP2K3,PTGS2,NFKBIB,MAPKAPK2,NOS2,MAP2K1
Differential Regulation of Cytokine Production in Intestinal Epithelial Cells by IL-17A and IL-17F	4.27E00	5.29E-01	NA	IL1A,CCL4,CCL2,IL12B,LCN2,IL1B,CCL5,CSF3,TNF
Role of JAK1, JAK2 and TYK2 in Interferon Signaling	4.19E00	4.76E-01	0.000	SOCS1,RELA,PTPN2,TYK2,STAT2,STAT3,JAK2,NFKB2,STAT1,NFKB1
April Mediated Signaling	4.11E00	3.68E-01	0.000	MAP3K14,RELA,NFKBIE,IKBKE,NFKB2,NFKB1,NFATC1,TRAF2,IKBKG,NFKBIA,NFATC2,TNFRSF13B,NFKBIB,TRAF1
Purine Nucleotides De Novo Biosynthesis II	4.1E00	6.36E-01	NA	ADSSL1,IMPDH2,PAICS,PFAS,PPAT,ATIC,GART
PPARα/RXRα Activation	4.04E00	2.26E-01	-1.807	MAP2K6,PRKACB,RELA,PLCB2,IL1RL1,SMAD3,NFKBIE,KRAS,JAK2,IL6,NFKB1,TGFBR2,PLCD3,HSP90B1,IKBKG,NFKBIA,IL1RL2,FASN,LPL,NFKBIB,MAP2K1,MAP3K14,PRKAR2A,BCL3,IKBKE,NFKB2,IL1R1,IL18RAP,HELZ2,PRKACA,PRKAG2,HSP90AA1,IL1B,MEF2C,MAP2K3,NCOR2,RXRA,ADCY7
IL-1 Signaling	3.98E00	2.7E-01	0.853	PRKACB,MAP2K6,RELA,MAP3K14,IL1A,NFKBIE,GNA12,PRKAR2A,IKBKE,IRAK3,NFKB2,IL1R1,NFKB1,GNG7,IKBKG,NFKBIA,PRKACA,PRKAG2,MAP2K3,GNA13,NFKBIB,ADCY7,GNG12,IRAK2
Role of CHK Proteins in Cell Cycle Checkpoint Control	3.98E00	3.21E-01	0.000	MDC1,RFC1,PLK1,RFC5,CDK1,RAD1,CHEK1,RFC4,PPP2R4,E2F1,CDKN1A,RFC2,E2F5,SLC19A1,CLSPN,E2F2,CDC25A

Role of Osteoblasts, Osteoclasts and Chondrocytes in Rheumatoid Arthritis	3.92E00	2.11E-01	NA	MAP2K6,RELA,IL1A,ADAM17,Naip1 (includes others),IL1RL1,NFKBIE,LRP6,PIK3R5,MMP13,IL6,NFKB1,NFATC1,SMURF1,BCL2,IKBKG,IL36G,NFKBIA,IGF1,IL1RL2,SMO,NFKBIB,TNFRSF1B,BIRC3,ADAMTS4,SRC,MAP3K14,NAIP,LRP5,ITGA5,IKBKE,PPP3CC,IL1R1,GSN,IL7,IL18RAP,TRAF2,FZD4,IL1RN,CSF1,NFATC2,IL1B,FZD5,MAP2K3,TNF
Differential Regulation of Cytokine Production in Macrophages and T Helper Cells by IL-17A and IL-17F	3.88E00	5.33E-01	NA	CCL4,CCL2,IL12B,IL1B,CCL5,IL6,CSF3,TNF
IL-8 Signaling	3.88E00	2.2E-01	1.183	RELA,PLCB2,ICAM1,PIK3R5,KRAS,NFKB1,CCND1,GNG7,NOX1,BCL2,RAB11FIP2,VEGFA,HMOX1,IKBKG,RHOB,CCND3,RHOU,CYBB,GNA13,NFKBIB,MAP2K1,GNG12,SRC,VCAM1,RHOC,GNA12,RHOJ,IKBKE,IRAK3,PLD4,CCND2,ITGAM,RHOQ,NCF2,PRKCH,PTGS2,KDR,ITGAX,IRAK2
Mitotic Roles of Polo-Like Kinase	3.87E00	2.97E-01	-1.604	KIF23,ESPL1,CDC20,WEE1,PTTG1,PRC1,CDC7,CCNB2,PLK1,CDK1,CCNB1,CDC25B,HSP90B1,PPP2R4,PLK2,HSP90AA1,FBXO5,KIF11,CDC25A
ATM Signaling	3.86E00	3.05E-01	-0.577	GADD45B,GADD45G,MDC1,CCNB2,MDM2,CBX5,CDK1,CHEK1,CCNB1,RAD51,NFKBIA,FANCD2,SMC2,GADD45A,H2AFX,CDKN1A,BID,CDC25A
B Cell Activating Factor Signaling	3.83E00	3.5E-01	0.905	MAP3K14,RELA,NFKBIE,IKBKE,NFKB2,NFKB1,NFATC1,TRAF2,IKBKG,NFKBIA,NFATC2,TNFRSF13B,NFKBIB,TRAF1
Cholecystokinin/Gastrin-mediated Signaling	3.78E00	2.58E-01	1.400	MAP2K6,IL1A,PLCB2,SRF,KRAS,BCAR1,IL36G,RHOB,RHOU,GNA13,MAP2K1,SRC,ITPR2,RHOC,GNA12,RHOJ,RHOQ,IL1RN,CREM,IL1B,MEF2C,MAP2K3,PRKCH,PTGS2,TNF
Chronic Myeloid Leukemia Signaling	3.74E00	2.61E-01	NA	RELA,HDAC9,RBL2,HDAC4,TFDP1,SUV39H1,SMAD3,PIK3R5,MDM2,IKBKE,KRAS,NFKB2,NFKB1,CCND1,HDAC5,TGFBR2,MYC,IKBKG,CDKN1A,E2F1,E2F5,CDKN1B,MAP2K1,E2F2
IL-15 Production	3.59E00	4.17E-01	NA	RELA,IL15,TYK2,JAK2,NFKB2,IL6,STAT1,NFKB1,FRK,IRF1
Pyridoxal 5'-phosphate Salvage Pathway	3.47E00	2.86E-01	NA	PDXK,MAP2K6,DAPK1,SGK1,CDK7,PLK1,TTK,CDK1,PNPO,CDK5,PIM1,GRK6,MAP2K3,MAP3K8,PRKCH,EIF2AK2,NEK2,MAP2K1
HMGB1 Signaling	3.45E00	2.39E-01	2.746	MAP2K6,RELA,IL1A,ICAM1,PIK3R5,KRAS,IL6,NFKB1,KAT2B,CCL2,RHOB,RHOU,OSM,SERPINE1,TNFRSF1B,MAP2K1,KAT2A,VCAM1,RHOC,RHOJ,IL1R1,NFKB2,RHOQ,IL12B,IL1B,MAP2K3,TNF

Pyrimidine Deoxyribonucleotides De Novo Biosynthesis I	3.39E00	4.29E-01	NA	DUT,NME4,AK1,CMPK2,AK8,RRM2,AK4,DTYMK,RRM1
PI3K/AKT Signaling	3.37E00	2.33E-01	2.746	RELA,NFKBIE,GDF15,KRAS,JAK2,NFKB1,CCND1,BCL2,IKBKG,HSP90B1,GYS1,NFKBIA,INPP5B,NFKBIB,MAP2K1,MCL1,TYK2,ITGA5,IKBKE,MDM2,NFKB2,PPP2R4,CDKN1A,HSP90AA1,MAP3K8,PTGS2,CDKN1B,SFN
MIF-mediated Glucocorticoid Regulation	3.18E00	3.55E-01	3.162	RELA,LY96,NFKBIA,NFKBIE,PLA2G5,CD14,CD74,PTGS2,NFKB2,NFKBIB,NFKB1
phagosome formation	3.16E00	2.4E-01	NA	MRC1,PLCB2,RHOC,FCGR2A,PIK3R5,TLR8,ITGA5,RHOJ,TLR9,FCGR1A,TLR2,PLCD3,Fcrls,CLEC7A,RHOQ,RHOB,SYK,TLR1,TLR6,RHOU,PRKCH,TLR3,FCGR3A/FCGR3B,MARCO
Regulation of IL-2 Expression in Activated and Anergic T Lymphocytes	3.11E00	2.56E-01	NA	RELA,NFKBIE,SMAD3,TOB1,IKBKE,KRAS,PPP3CC,MALT1,NFKB2,NFKB1,NFATC1,TGFBR2,CD28,IKBKG,NFKBIA,CARD11,BCL10,NFATC2,NFKBIB,MAP2K1
Production of Nitric Oxide and Reactive Oxygen Species in Macrophages	3.02E00	2.07E-01	3.430	RAP1B,APOE,RELA,NFKBIE,PIK3R5,JAK2,NFKB1,IKBKG,LYZ,NFKBIA,RHOB,RHOU,CYBB,NOS2,STAT1,TNFRSF1B,NFKBIB,MAP2K1,MAP3K14,RHOC,TYK2,IKBKE,RHOJ,NFKB2,NCF4,IRF1,TLR2,MAP3K12,RHOQ,PPP2R4,NCF2,CAT,MAP3K8,PRKCH,TNF
Relaxin Signaling	3.02E00	2.2E-01	0.229	RAP1B,PRKACB,RELA,GUCY2C,NFKBIE,PIK3R5,NFKB1,GNG7,VEGFA,NFKBIA,PDE7B,GNA13,NFKBIB,NOS2,MAP2K1,Gucy2g,APEX1,GNG12,PDE2A,PDE10A,GNA12,PRKAR2A,PDE4B,NFKB2,PDE1B,PRKACA,PRKAG2,PDE8B,ADCY7
IL-12 Signaling and Production in Macrophages	2.96E00	2.26E-01	NA	RELA,APOE,IL12RB1,MAF,PIK3R5,NFKB1,JMJD6,IKBKG,LYZ,NOS2,NFKBIB,STAT1,MAP2K1,IKBKE,IL12RB2,CEBPB,NFKB2,IRF1,TLR2,CD40,IL12B,PRKCH,MAP3K8,REL,RXRA,TNF
RANK Signaling in Osteoclasts	2.94E00	2.44E-01	1.698	MAP2K6,RELA,MAP3K14,SRC,NFKBIE,PIK3R5,IKBKE,NFKB2,PPP3CC,NFKB1,GSN,NFATC1,MAP3K12,IKBKG,TRAF2,NFKBIA,NFATC2,MAP3K8,NFKBIB,MAP2K1,BIRC3
Lymphotoxin β Receptor Signaling	2.94E00	2.83E-01	1.732	RELA,MAP3K14,IKBKG,TRAF2,VCAM1,NFKBIA,CASP3,RELB,PIK3R5,APAF1,IKBKE,NFKB2,NFKB1,TNFSF14,TRAF1
Complement System	2.92E00	3.33E-01	0.000	CFD,ITGAM,C3,C5AR1,CFB,C1QC,C1QA,C1QBP,CFH,C3AR1,ITGAX
OX40 Signaling Pathway	2.91E00	2.66E-01	0.000	B2M,RELA,H2-T24,H2-T22,H2-T10,HLA-A,NFKBIE,H2-K2/H2-Q9,NFKB2,NFKB1,BCL2,HLA-G,TRAF2,NFKBIA,H2-M2,NFKBIB,HLA-E

Salvage Pathways of Pyrimidine Ribonucleotides	2.87E00	2.41E-01	NA	MAP2K6,DAPK1,NME4,CMPK2,SGK1,AK8,CDK7,PLK1,TTK,CDK1,AK1,CDK5,PIM1,GRK6,AK4,MAP3K8,MAP2K3,PRKCH,EIF2AK2,NEK2,MAP2K1
Atherosclerosis Signaling	2.84E00	2.25E-01	NA	CCR3,APOE,RELA,VCAM1,IL1A,ICAM1,CXCR4,MMP13,NFKB2,IL6,NFKB1,PLA2G7,SELPLG,IL36G,LYZ,CD40,CCL2,IL1RN,CSF1,PLA2G5,LPL,IL1B,COL18A1,TNF,TNFSF14
IL-15 Signaling	2.83E00	2.62E-01	NA	RELA,IL15RA,VCAM1,TYK2,IL15,PIK3R5,KRAS,IL6,STAT3,JAK2,NFKB2,NFKB1,BCL2,TRAF2,SYK,TNF,MAP2K1
Prostate Cancer Signaling	2.82E00	2.44E-01	NA	RELA,TFDP1,SUV39H1,NFKBIE,PIK3R5,MDM2,KRAS,NFKB2,NFKB1,CCND1,BCL2,HSP90B1,CCNE1,NFKBIA,CDKN1A,E2F1,HSP90AA1,CDKN1B,NFKBIB,MAP2K1
MIF Regulation of Innate Immunity	2.79E00	3.08E-01	3.464	RELA,LY96,NFKBIA,NFKBIE,PLA2G5,CD14,PTGS2,CD74,NFKB2,NFKBIB,NFKB1,NOS2
5-aminoimidazole Ribonucleotide Biosynthesis I	2.75E00	1E00	NA	PFAS,PPAT,GART
Glutathione Biosynthesis	2.75E00	1E00	NA	GCLC,GCLM,GSS
Role of JAK family kinases in IL-6-type Cytokine Signaling	2.74E00	3.6E-01	NA	SOCS1,SOCS3,IL6R,TYK2,OSM,STAT3,JAK2,IL6,STAT1
Gαq Signaling	2.72E00	2.08E-01	1.569	RELA,PLCB2,RGS18,NFKBIE,PIK3R5,NFKB1,NFATC1,GNG7,HMOX1,IKBKG,GYS1,NFKBIA,RHOB,RHOU,NFKBIB,MAP2K1,GNG12,RGS2,RHOC,ITPR2,IKBKE,RHOJ,PPP3CC,NFKB2,BTK,PLD4,RHOQ,NFATC2,PRKCH,ADRA1A
Pyrimidine Ribonucleotides De Novo Biosynthesis	2.7E00	3.33E-01	NA	NME4,AK1,CMPK2,ENTPD1,AK8,ENTPD6,AK4,CAD,UMPS,CTPS1
Folate Transformations I	2.67E00	5.56E-01	NA	MTHFR,SHMT1,MTR,MTHFD1,SHMT2
STAT3 Pathway	2.65E00	2.47E-01	-0.943	SOCS1,SOCS3,SRC,PTPN2,TYK2,KRAS,JAK2,STAT3,BCL2,MYC,TGFBR2,MAP3K12,PIM1,CDKN1A,IGF1R,KDR,MAP2K1,CDC25A

Protein Kinase A Signaling	2.63E00	1.72E-01	-0.714	PRKACB,MYH10,PLCB2,PTPN14,SMAD3,NTN1,TGFBR2,PLCD3,GYS1,PDE7B,GNA13,NFKBIB,APEX1,GNG12,CDC25A,PDE2A,PDE10A,ITPR2,PTCH1,PTPN18,NFKB2,PDE4B,PPP3CC,EPM2A,AKAP13,DUSP1,PDE1B,CREM,PRKACA,HIST1H1D,PRKCH,H1F0,EBI3,AKAP12,RAP1B,FLNB,RELA,PTPN2,TNNI2,NFKBIE,DUSP6,NFKB1,GNG7,NFATC1,CDC25B,CDKN3,DUSP5,NFKBIA,PTPRJ,DUSP10,SMO,MAP2K1,PRKAR2A,Hist1h1e,ADD3,PRKAG2,NFATC2,PDE8B,PTGS2,SFN,ADCY7,DUSP16,AKAP1
Pathogenesis of Multiple Sclerosis	2.58E00	6.67E-01	NA	CXCL10,CCL4,CXCR3,CCL5
Tumoricidal Function of Hepatic Natural Killer Cells	2.52E00	3.64E-01	1.633	M6PR,SERPINB9,ICAM1,CASP3,APAF1,BID,CASP7,FAS
Xenobiotic Metabolism Signaling	2.47E00	1.81E-01	NA	MAP2K6,RELA,IL1A,CAMK1,GSTM5,UGT1A6,MAF,PIK3R5,GCLC,KRAS,IL6,NFKB1,HMOX1,HS6ST1,HSP90B1,UST,CHST10,NOS2,MAP2K1,AHR,GSTM1,ABCB1,MAP3K14,HDAC4,NQO1,CHST12,NFKB2,ESD,HDAC5,MAP3K12,MGST2,PPP2R4,SMOX,ALDH1A2,CAT,HSP90AA1,IL1B,MAP3K8,MAP2K3,PRKCH,NCOR2,RXRA,ABCC3,TNF,MGST3
p53 Signaling	2.43E00	2.23E-01	2.324	HDAC9,GADD45B,TOPBP1,GADD45G,PIK3R5,APAF1,MDM2,BIRC5,CCND1,FAS,CHEK1,BCL2,CCND2,KAT2B,GADD45A,THBS1,CDKN1A,E2F1,ADCK3,PML,SFN
Agranulocyte Adhesion and Diapedesis	2.43E00	1.96E-01	NA	MYH10,IL1A,ICAM1,MMP13,CCL24,CCL5,SDC4,ITGB7,CXCL10,CXCL3,IL36G,CCL2,CCL3L3,Ccl8,Ccl2,Cxcl3,VCAM1,C5AR1,Cxcl11,CXCR4,ITGA6,ITGA5,IL1R1,SELPLG,Ccl9,CCL4,IL1RN,IL1B,CXCL2,TNF,Ccl7,MSN
PI3K Signaling in B Lymphocytes	2.42E00	2.08E-01	3.138	BLNK,RELA,IL4R,PLCB2,ATF3,C3,ITPR2,NFKBIE,IKBKE,KRAS,NFKB2,PPP3CC,MALT1,NFKB1,NFATC1,BTK,PLCD3,IKBKG,NFKBIA,CD40,BCL10,SYK,NFATC2,PIK3AP1,NFKBIB,MAP2K1
LPS-stimulated MAPK Signaling	2.39E00	2.39E-01	0.728	MAP2K6,RELA,MAP3K14,NFKBIE,SRF,PIK3R5,IKBKE,KRAS,NFKB2,NFKB1,IKBKG,NFKBIA,CD14,MAP2K3,PRKCH,NFKBIB,MAP2K1
Graft-versus-Host Disease Signaling	2.37E00	2.89E-01	NA	HLA-G,CD28,IL36G,IL1A,HLA-A,IL1RN,IL1B,IL6,TNF,FAS,HLA-E
IL-17A Signaling in Airway Cells	2.36E00	2.5E-01	2.138	RELA,NFKBIE,TYK2,PIK3R5,IKBKE,IL6,STAT3,JAK2,NFKB2,NFKB1,CXCL3,IKBKG,NFKBIA,NFKBIB,MAP2K1

Protein Ubiquitination Pathway	2.34E00	1.8E-01	NA	B2M,USP12,USP18,USP35,CDC20,HLA-A,DNAJB4,PSMB10,USP53,CUL1,Ubb,PSMB8,HSPA5,TAP1,SMURF1,UCHL1,PSMD10,HSP90B1,USP42,DNAJB1,NEDD4L,BIRC3,HSPA4L,PSMB9,DNAJB12,DNAJC9,DNAJC19,PSMD5,PSME2,MDM2,DNAJC1,DNAJB9,DNAJB14,PSMD8,SKP2,PSMD11,PSME1,PSMA5,UBE2E2,HSP90AA1,HSPB7,UBC,TAP2,USP25
IL-17 Signaling	2.34E00	2.42E-01	NA	MAP2K6,RELA,MAP3K14,PIK3R5,KRAS,IL6,CEBPB,JAK2,NFKB1,CXCL10,CCL2,MAP2K3,PTGS2,MAPKAPK2,NOS2,MAP2K1
Gustation Pathway	2.34E00	2.13E-01	NA	PRKACB,PDE2A,PLCB2,PDE10A,ITPR2,P2RX4,PRKAR2A,PDE4B,PANX1,GNG7,P2RY13,P2RY2,LPAR6,PDE7B,P2RY6,PDE1B,PRKACA,PRKAG2,P2RY12,P2RY1,PDE8B,ADCY7,APEX1
G-Protein Coupled Receptor Signaling	2.33E00	1.79E-01	NA	PRKACB,RELA,PLCB2,RGS18,DUSP6,NFKBIE,PIK3R5,KRAS,NFKB1,IKBKG,ADRB1,NFKBIA,PDE7B,CNR2,RGS10,NFKBIB,APEX1,MAP2K1,HCAR2,PTGER4,ADRB2,SRC,RGS2,PDE2A,PTGIR,PDE10A,PRKAR2A,FPR2,IKBKE,PDE4B,STAT3,NFKB2,XCR1,P2RY13,DUSP1,PDE1B,S1PR1,PRKACA,PRKAG2,P2RY12,PDE8B,MAP3K8,ADORA2A,ADCY7,ADRA1A
B Cell Receptor Signaling	2.31E00	1.93E-01	1.257	MAP2K6,RAP1B,BLNK,RELA,NFKBIE,PIK3R5,KRAS,NFKB1,NFATC1,IKBKG,NFKBIA,INPP5B,RASSF5,NFKBIB,MAP2K1,MAP3K14,FCGR2A,IKBKE,MALT1,PPP3CC,NFKB2,BTK,MAP3K12,BCL10,PAG1,SYK,NFATC2,MAP2K3,MEF2C,MAP3K8,PIK3AP1,BCL2A1
Role of JAK1 and JAK3 in γc Cytokine Signaling	2.29E00	2.46E-01	NA	BLNK,SOCS1,SOCS3,IL15RA,IL4R,IL15,PIK3R5,KRAS,STAT3,JAK2,CRLF2,IL7,IL7R,SYK,STAT1
Phospholipase C Signaling	2.27E00	1.82E-01	1.414	BLNK,RAP1B,RELA,PLCB2,ARHGEF7,KRAS,ARHGEF1,NFKB1,GNG7,NFATC1,Ppp1r12b,HMOX1,RHOB,PLA2G5,RHOU,GNA13,MAP2K1,GNG12,HDAC9,SRC,ARHGEF4,HDAC4,ARHGEF12,ITPR2,FCGR2A,RHOC,ITGA5,RHOJ,PPP3CC,NFKB2,HDAC5,BTK,PLD4,RHOQ,SYK,NFATC2,MEF2C,PRKCH,ADCY7,LCP2
Granzyme B Signaling	2.23E00	4E-01	0.000	CASP3,APAF1,BID,LMNB2,LMNB1,PARP1
γ-glutamyl Cycle	2.2E00	4.55E-01	NA	GGCT,GCLC,GCLM,GSS,ANPEP
Heme Degradation	2.19E00	7.5E-01	NA	HMOX1,BLVRA,BLVRB
Acetate Conversion to Acetyl-CoA	2.19E00	7.5E-01	NA	ACSS2,ACSS1,ACSL1
Role of IL-17F in Allergic Inflammatory Airway Diseases	2.19E00	2.75E-01	3.000	CXCL10,RELA,CCL4,IGF1,CCL2,MMP13,IL1B,NFKB2,IL6,NFKB1,MAP2K1

Endoplasmic Reticulum Stress Pathway	2.16E00	3.5E-01	NA	TRAF2,HSP90B1,CASP3,DDIT3,HSPA5,CASP7,MBTPS2
cAMP-mediated signaling	2.09E00	1.79E-01	0.686	PRKACB,AKAP12,RGS18,CAMK1,DUSP6,ADRB1,PDE7B,CNR2,RGS10,HCAR2,MAP2K1,APEX1,PTGER4,ADRB2,SRC,RGS2,PDE2A,PTGIR,PDE10A,FPR2,PRKAR2A,STAT3,PPP3CC,PDE4B,XCR1,P2RY13,AKAP13,DUSP1,PKIB,CREM,PDE1B,S1PR1,PRKACA,P2RY12,PDE8B,ADORA2A,ADCY7,AKAP1
Antiproliferative Role of TOB in T Cell Signaling	2.02E00	3.08E-01	NA	TGFBR2,CCNA2,CCNE1,SMAD3,CUL1,TOB1,CDKN1B,SKP2
Role of Cytokines in Mediating Communication between Immune Cells	1.98E00	2.7E-01	NA	IL36G,IL1A,IL1RN,IL12B,IL15,IL27,IL1B,IL6,CSF3,TNF
VDR/RXR Activation	1.97E00	2.18E-01	1.414	SERPINB1,LRP5,IL1RL1,CEBPB,CCL5,THBD,CXCL10,GADD45A,MXD1,CDKN1A,CEBPA,CD14,PRKCH,NCOR2,CDKN1B,RXRA,HSD17B2
JAK/Stat Signaling	1.96E00	2.22E-01	2.000	RELA,SOCS1,SOCS3,TYK2,PIK3R5,KRAS,IL6,STAT3,CEBPB,JAK2,NFKB2,NFKB1,CDKN1A,STAT2,STAT1,MAP2K1
T Helper Cell Differentiation	1.96E00	2.27E-01	NA	IL4R,IL12RB1,IL6R,IL12RB2,STAT3,IL6,TGFBR2,CD28,CD40,IL12B,ICOS,STAT1,TNFRSF1B,TNF,ICOSLG/LOC102723996
Ovarian Cancer Signaling	1.95E00	1.94E-01	NA	PRKACB,SRC,TFDP1,SUV39H1,PTGS1,PRKAR2A,PIK3R5,KRAS,CCND1,MLH1,BCL2,RAD51,VEGFA,FZD4,MSH2,EDN1,E2F1,PRKAG2,PRKACA,MSH6,SMO,BRCA2,FZD5,PTGS2,MAP2K1
Cytotoxic T Lymphocyte-mediated Apoptosis of Target Cells	1.92E00	2.96E-01	2.449	B2M,CASP3,HLA-A,APAF1,BID,CASP7,FAS,BCL2
Allograft Rejection Signaling	1.9E00	2.36E-01	NA	B2M,H2-T24,H2-T22,H2-T10,HLA-A,H2-K2/H2-Q9,FAS,HLA-G,CD28,CD40,H2-M2,TNF,HLA-E
NF-κB Activation by Viruses	1.9E00	2.19E-01	NA	MAP3K14,RELA,NFKBIE,ITGA6,PIK3R5,ITGA5,KRAS,IKBKE,NFKB2,NFKB1,TRAF2,IKBKG,NFKBIA,PRKCH,EIF2AK2,NFKBIB
fMLP Signaling in Neutrophils	1.87E00	2E-01	0.229	RELA,PLCB2,ITPR2,NFKBIE,FPR2,PIK3R5,KRAS,PPP3CC,NFKB2,NFKB1,NFATC1,NOX1,GNG7,NFKBIA,NCF2,CYBB,NFATC2,PRKCH,NFKBIB,MAP2K1,GNG12
Tec Kinase Signaling	1.86E00	1.85E-01	2.985	RELA,GTF2I,PIK3R5,TNFSF10,JAK2,NFKB1,GNG7,FAS,RHOB,RHOU,HCK,GNA13,STAT1,GNG12,SRC,RHOC,GNA12,TYK2,ITGA5,RHOJ,STAT3,NFKB2,FRK,BTK,RHOQ,STAT2,PRKCH,TNF

Oncostatin M Signaling	1.85E00	2.73E-01	1.667	TYK2,MMP13,OSM,KRAS,STAT3,PLAU,JAK2,STAT1,MAP2K1
Uridine-5'-phosphate Biosynthesis	1.84E00	1E00	NA	CAD,UMPS
Glycine Degradation (Creatine Biosynthesis)	1.84E00	1E00	NA	GAMT,GATM
Glycine Biosynthesis I	1.84E00	1E00	NA	SHMT1,SHMT2
Glycogen Biosynthesis II (from UDP-D-Glucose)	1.84E00	6E-01	NA	GYS1,GYG1,GBE1
Folate Polyglutamylation	1.84E00	6E-01	NA	SHMT1,MTHFD1,SHMT2
Pyrimidine Ribonucleotides Interconversion	1.82E00	2.86E-01	NA	NME4,AK1,CMPK2,ENTPD1,AK8,ENTPD6,AK4,CTPS1
Sonic Hedgehog Signaling	1.82E00	2.86E-01	1.633	PRKACB,PTCH1,PRKAR2A,PRKACA,SMO,PRKAG2,CDK1,CCNB1
Primary Immunodeficiency Signaling	1.81E00	2.56E-01	NA	IL7R,BLNK,BTK,IKBKG,CD40,ICOS,ADA,TNFRSF13B,TAP1,TAP2
Purine Nucleotides Degradation II (Aerobic)	1.8E00	3.33E-01	NA	NT5C3A,GDA,IMPDH2,XDH,PNP,ADA
Glycolysis I	1.8E00	3.04E-01	NA	PGK1,TPI1,ENO3,PGAM1,PFKP,PFKL,PFKM
Antioxidant Action of Vitamin C	1.79E00	2.02E-01	-3.153	RELA,PLCB2,SLC2A1,NFKBIE,IKBKE,NFKB2,JAK2,NFKB1,PLA2G7,TXNRD1,PLD4,HMOX1,PLCD3,IKBKG,NFKBIA,CSF2RA,PLA2G5,NFKBIB,TNF
IL-9 Signaling	1.77E00	2.65E-01	1.667	SOCS3,RELA,PIK3R5,BCL3,STAT3,NFKB2,STAT1,NFKB1,TNF
Type II Diabetes Mellitus Signaling	1.76E00	1.93E-01	1.698	RELA,SOCS1,SOCS3,MAP3K14,NFKBIE,PIK3R5,IKBKE,NFKB2,CEBPB,NFKB1,SMPD2,TRAF2,IKBKG,ACSBG1,NFKBIA,ACSL5,PRKAG2,PRKCH,TNFRSF1B,NFKBIB,TNF,ACSL1
Cardiac Hypertrophy Signaling	1.75E00	1.71E-01	-1.715	MAP2K6,PRKACB,PLCB2,PIK3R5,SRF,KRAS,IL6,GNG7,TGFBR2,PLCD3,ADRB1,RHOB,IGF1,RHOU,IGF1R,GNA13,MAPKAPK2,MAP2K1,GNG12,ADRB2,MAP3K14,CACNA1D,RHOC,GNA12,IL6R,PRKAR2A,RHOJ,PPP3CC,MAP3K12,RHOQ,PRKACA,PRKAG2,MEF2C,MAP3K8,MAP2K3,ADCY7,ADRA1A

Role of NFAT in Regulation of the Immune Response	1.71E00	1.79E-01	1.043	BLNK,RELA,PLCB2,NFKBIE,PIK3R5,KRAS,NFKB1,FCGR1A,NFATC1,GNG7,CD28,IKBKG,NFKBIA,GNA13,NFKBIB,MAP2K1,GNG12,FCGR3A/FCGR3B,FCGR2A,ITPR2,GNA12,IKBKE,PPP3CC,NFKB2,BTK,SYK,NFATC2,MEF2C,LCP2
Amyotrophic Lateral Sclerosis Signaling	1.7E00	1.98E-01	NA	NAIP,Naip1 (includes others),CACNA1D,GDNF,CASP3,APAF1,PIK3R5,NEFH,GRINA,BCL2,VEGFA,RAB5A,IGF1,CAT,CASP1,BID,BIRC3,RNF19A,CASP7
T Cell Receptor Signaling	1.7E00	1.98E-01	NA	RELA,PIK3R5,IKBKE,KRAS,PPP3CC,MALT1,NFKB2,NFKB1,NFATC1,BTK,CD28,IKBKG,NFKBIA,CARD11,BCL10,PAG1,NFATC2,MAP2K1,LCP2
Neuroprotective Role of THOP1 in Alzheimer's Disease	1.69E00	2.57E-01	NA	PRKACB,HLA-G,ECE2,HLA-A,PRKAR2A,PRKACA,PRKAG2,HLA-E,APP
DNA damage-induced 14-3-3σ Signaling	1.68E00	3.16E-01	NA	CCNE1,CCNB2,SFN,CDK1,CCNB1,RAD1
Acute Myeloid Leukemia Signaling	1.64E00	2.05E-01	NA	MAP2K6,RELA,PIK3R5,KRAS,STAT3,NFKB2,NFKB1,CCND1,MYC,CSF2RA,PIM1,CEBPA,MAP2K3,IDH2,PML,MAP2K1
Glioblastoma Multiforme Signaling	1.63E00	1.81E-01	0.426	SRC,PLCB2,RHOC,ITPR2,PIK3R5,RHOJ,MDM2,KRAS,CCND1,MYC,PLCD3,CCNE1,FZD4,RHOQ,IGF1,RHOB,E2F1,CDKN1A,E2F5,IGF1R,RHOU,SMO,FZD5,CDKN1B,MAP2K1,E2F2
Role of MAPK Signaling in the Pathogenesis of Influenza	1.61E00	2.12E-01	NA	MAP2K6,CASP3,KRAS,CCL5,PLA2G7,RABGEF1,BCL2,CXCL10,CCL2,PLA2G5,MAP2K3,PTGS2,TNF,MAP2K1
Selenocysteine Biosynthesis II (Archaea and Eukaryotes)	1.57E00	5E-01	NA	SEPHS2,PSTK,SEPSECS
PKCθ Signaling in T Lymphocytes	1.57E00	1.88E-01	1.528	RELA,MAP3K14,NFKBIE,PIK3R5,IKBKE,KRAS,PPP3CC,MALT1,NFKB2,NFKB1,NFATC1,MAP3K12,CD28,IKBKG,NFKBIA,CARD11,BCL10,NFATC2,MAP3K8,NFKBIB,LCP2
Erythropoietin Signaling	1.56E00	2.09E-01	NA	SOCS1,SOCS3,SRC,RELA,NFKBIE,PIK3R5,KRAS,JAK2,NFKB2,NFKB1,NFKBIA,PRKCH,NFKBIB,MAP2K1
AMPK Signaling	1.55E00	1.72E-01	-0.229	PRKACB,AK8,PIK3R5,PFKL,CCND1,CCNA2,AK1,ADRB1,KAT2B,GYS1,PCK2,FASN,EEF2K,ADRB2,SRC,SLC2A1,STRADA,AK3,PRKAR2A,PFKP,PFKM,PPP2R4,CDKN1A,PRKACA,PRKAG2,AK4,MAP2K3,MLYCD,PPAT,ADRA1A

UVA-Induced MAPK Signaling	1.52E00	1.95E-01	2.500	PARP16,PLCB2,CASP3,PARP10,ZC3HAV1,PIK3R5,PARP8,PARP12,KRAS,PARP9,SMPD2,PARP1,PLCD3,TIPARP,PARP11,STAT1,PARP14
Renin-Angiotensin Signaling	1.51E00	1.87E-01	1.213	PRKACB,RELA,ITPR2,PIK3R5,PRKAR2A,KRAS,NFKB2,STAT3,JAK2,CCL5,NFKB1,NOX1,CCL2,PRKACA,PRKAG2,PRKCH,STAT1,TNF,MAP2K1,ADCY7
Androgen Signaling	1.51E00	1.87E-01	0.577	PRKACB,RELA,SRC,CDK7,GNA12,SMAD3,PRKAR2A,NFKB2,NFKB1,CCND1,GNG7,KAT2B,GTF2H4,PRKACA,PRKAG2,HSP90AA1,PRKCH,GNA13,DNAJB1,GNG12
Glioma Signaling	1.5E00	1.91E-01	-1.069	RBL2,CAMK1,TFDP1,SUV39H1,PIK3R5,CDKN2C,MDM2,KRAS,CCND1,CDKN2B,IGF1,CDKN1A,E2F1,E2F5,IGF1R,PRKCH,MAP2K1,E2F2
TR/RXR Activation	1.5E00	1.98E-01	NA	UCP2,SLC2A1,PIK3R5,MDM2,BCL3,PFKP,KLF9,HP,SLC16A3,ADRB1,LDLR,SCARB1,FASN,NCOR2,TBL1XR1,RXRA
Triacylglycerol Biosynthesis	1.48E00	2.5E-01	NA	AGPAT4,PPAPDC1B,LPIN1,PPAP2B,DGAT2,LPIN2,Ppap2a,ELOVL6
Netrin Signaling	1.47E00	2.37E-01	NA	PRKACB,PRKG1,PRKAR2A,PRKACA,PRKAG2,NFATC2,PPP3CC,NTN1,NFATC1
Hematopoiesis from Multipotent Stem Cells	1.46E00	3.64E-01	NA	CSF1,IL15,CSF3,IL7
γ-linolenate Biosynthesis II (Animals)	1.45E00	3.12E-01	NA	ACSBG1,ACSL5,CYB5A,ACSL1,FADS1
PTEN Signaling	1.42E00	1.81E-01	0.655	RELA,CASP3,PIK3R5,ITGA5,IKBKE,KRAS,NFKB2,NFKB1,CNKSR3,CCND1,BCAR1,BCL2,TGFBR2,IKBKG,INPP5B,CDKN1A,IGF1R,CDKN1B,KDR,MAP2K1,BCL2L11
Nitric Oxide Signaling in the Cardiovascular System	1.42E00	1.88E-01	0.000	PRKACB,PDE2A,CACNA1D,GUCY2C,ITPR2,PIK3R5,PRKAR2A,VEGFA,HSP90B1,ADRB1,PRKG1,PDE1B,PRKACA,PRKAG2,HSP90AA1,PRKCH,KDR,MAP2K1
Role of PI3K/AKT Signaling in the Pathogenesis of Influenza	1.41E00	2.16E-01	1.000	KPNA3,RELA,NFKBIA,NFKBIE,PIK3R5,CCL5,NFKB2,NFKBIB,NFKB1,MAP2K1,MLH1
Cell Cycle Regulation by BTG Family Proteins	1.41E00	2.42E-01	0.378	CCNE1,PPP2R4,E2F1,BTG2,E2F5,BTG1,CCND1,E2F2
Docosahexaenoic Acid (DHA) Signaling	1.4E00	2.31E-01	NA	CASP3,APAF1,SERPINF1,PIK3R5,BID,IL1B,BCL2A1,APP,BCL2

Methylglyoxal Degradation I	1.39E00	6.67E-01	NA	HAGH,GLO1
Inosine-5'-phosphate Biosynthesis II	1.39E00	6.67E-01	NA	PAICS,ATIC
P2Y Purigenic Receptor Signaling Pathway	1.39E00	1.79E-01	-0.447	PRKACB,RELA,PLCB2,PRKAR2A,PIK3R5,KRAS,NFKB2,NFKB1,GNG7,MYC,PLCD3,P2RY2,P2RY6,PRKACA,PRKAG2,P2RY12,P2RY1,PRKCH,MAP2K1,ADCY7,GNG12
RAR Activation	1.38E00	1.66E-01	NA	PRKACB,RELA,SMAD3,JAK2,NFKB1,VEGFA,TNIP1,KAT2B,PNRC1,MAPKAPK2,MAP2K1,SRC,PRMT2,RELB,CDK7,PRKAR2A,SNW1,NFKB2,PARP1,ADH7,DUSP1,GTF2H4,ALDH1A2,PRKACA,PRKAG2,PRKCH,NCOR2,REL,PML,RXRA,ADCY7
Superpathway of Serine and Glycine Biosynthesis I	1.37E00	4.29E-01	NA	PSAT1,SHMT1,SHMT2
Pregnenolone Biosynthesis	1.37E00	4.29E-01	NA	MICAL1,MICAL2,MICAL3
CD28 Signaling in T Helper Cells	1.36E00	1.8E-01	2.183	RELA,ITPR2,NFKBIE,PIK3R5,IKBKE,PPP3CC,MALT1,NFKB2,NFKB1,NFATC1,CD28,IKBKG,NFKBIA,CARD11,BCL10,SYK,NFATC2,NFKBIB,MAP2K1,LCP2
phagosome maturation	1.36E00	1.8E-01	NA	M6PR,B2M,PRDX5,HLA-A,PRDX1,TCIRG1,ATP6V0D2,VPS37C,TAP1,NOX1,ATP6V0A1,DYNC1H1,RAB5A,TUBA1A,Dync1i2,ATP6V1H,NCF2,RAB7B,CYBB,BET1L
Ceramide Signaling	1.34E00	1.92E-01	0.535	RELA,SPHK2,PIK3R5,KRAS,CERK,NFKB2,NFKB1,SMPD2,BCL2,PPP2R4,SPHK1,S1PR1,TNFRSF1B,TNF,MAP2K1
Angiopoietin Signaling	1.34E00	2E-01	-1.732	RELA,DOK2,NFKBIE,PIK3R5,IKBKE,KRAS,NFKB2,NFKB1,BIRC5,IKBKG,TNIP1,NFKBIA,NFKBIB
Sphingosine-1-phosphate Signaling	1.33E00	1.81E-01	-0.471	PLCB2,CASP3,RHOC,GNA12,PIK3R5,RHOJ,CASP4,SMPD2,PLCD3,NAAA,RHOQ,RHOB,SPHK1,CASP1,S1PR1,RHOU,GNA13,ADCY7,CASP7
Unfolded protein response	1.3E00	2.08E-01	NA	TRAF2,HSP90B1,DDIT3,PPP1R15A,CEBPA,DNAJA2,CEBPB,DNAJB9,HSPA5,MBTPS2,BCL2
Gαs Signaling	1.3E00	1.79E-01	0.000	PRKACB,SRC,RGS2,PTGIR,PRKAR2A,GNG7,ADRB1,ADD3,RAPGEF2,PRKAG2,PRKACA,HCK,ADORA2A,MAP2K1,ADCY7,HCAR2,GNG12,PTGER4,ADRB2
Chemokine Signaling	1.3E00	1.97E-01	0.832	Ppp1r12b,SRC,CCR3,PLCB2,CCL4,CAMK1,CCL2,CXCR4,CCL24,KRAS,CCL5,MAP2K1,NOX1
Cardiac β-adrenergic Signaling	1.28E00	1.73E-01	-0.500	PRKACB,AKAP12,PDE2A,CACNA1D,PDE10A,PRKAR2A,SLC8A2,PDE4B,GNG7,AKAP13,ADRB1,PDE7B,PPP2R4,PKIB,PDE1B,PRKACA,PRKAG2,PDE8B,ADCY7,APEX1,GNG12,AKAP1

PXR/RXR Activation	1.23E00	1.97E-01	NA	PRKACB,GSTM1,ABCB1,RELA,PCK2,PRKAR2A,PRKACA,PRKAG2,IL6,RXRA,ABCC3,TNF
LPS/IL-1 Mediated Inhibition of RXR Function	1.23E00	1.6E-01	2.138	APOE,IL1A,GSTM5,IL1RL1,HS6ST1,IL36G,ACSBG1,UST,IL1RL2,SCARB1,ACSL5,CHST10,TNFRSF1B,GSTM1,ABCB1,IL1R1,CHST12,IL18RAP,TRAF2,LY96,MGST2,SMOX,IL1RN,ALDH1A2,CAT,CD14,IL1B,RXRA,ABCC3,TNF,ACSL1,MGST3
Superoxide Radicals Degradation	1.21E00	3.75E-01	NA	SOD2,CAT,NQO1
Ubiquinol-10 Biosynthesis (Eukaryotic)	1.21E00	3.08E-01	NA	MICAL1,MICAL2,COQ2,MICAL3
Guanosine Nucleotides Degradation III	1.21E00	3.08E-01	NA	NT5C3A,GDA,XDH,PNP
iCOS-iCOSL Signaling in T Helper Cells	1.2E00	1.76E-01	1.604	RELA,ITPR2,NFKBIE,PIK3R5,IKBKE,PPP3CC,NFKB2,NFKB1,NFATC1,CD28,IKBKG,NFKBIA,CD40,ICOS,NFATC2,NFKBIB,LCP2,ICOSLG/LOC102723996
HGF Signaling	1.2E00	1.76E-01	0.500	RAP1B,RAPGEF1,MAP3K14,PIK3R5,ITGA5,KRAS,IL6,STAT3,CCND1,MET,DOCK1,MAP3K12,ETS2,CDKN1A,MAP3K8,PRKCH,PTGS2,MAP2K1
GM-CSF Signaling	1.18E00	1.94E-01	1.897	CSF2RA,PIM1,HCK,PIK3R5,KRAS,PPP3CC,STAT3,JAK2,STAT1,BCL2A1,CCND1,MAP2K1
3-phosphoinositide Degradation	1.16E00	1.67E-01	NA	SOCS3,PTPN2,PPFIA3,PPFIBP2,MTMR14,MTMR1,MDP1,PPM1K,DUSP14,CDC25B,INPP4A,ITPK1,INPP4B,PTPRJ,PPP2R4,DUSP1,DUSP10,INPP5B,PALD1,EYA4,NUDT4,CDC25A,DUSP16
Adipogenesis pathway	1.16E00	1.69E-01	NA	HDAC9,HDAC4,DDIT3,CDK7,SMAD3,SAP30,CEBPB,HDAC5,CTNNBIP1,FZD4,LPIN1,EGR2,CDK5,GTF2H4,LPL,SETDB1,CEBPA,SMO,FZD5,TBL1XR1,TNF
RhoA Signaling	1.16E00	1.71E-01	0.243	PLXNA1,ARHGEF12,NRP2,ANLN,GNA12,CDC42EP3,ARHGEF1,CDC42EP2,PIP5K1B,Ppp1r12b,LPAR6,RHPN2,RAPGEF2,IGF1,PLEKHG5,CIT,BAIAP2,IGF1R,GNA13,MSN
Role of NFAT in Cardiac Hypertrophy	1.16E00	1.61E-01	-1.347	MAP2K6,PRKACB,PLCB2,CAMK1,PIK3R5,KRAS,IL6,GNG7,TGFBR2,PLCD3,IGF1,IGF1R,MAP2K1,GNG12,HDAC9,SRC,HDAC4,ITPR2,PRKAR2A,SLC8A2,PPP3CC,HDAC5,PRKAG2,PRKACA,MAP2K3,MEF2C,PRKCH,ADCY7
Arsenate Detoxification I (Glutaredoxin)	1.13E00	5E-01	NA	GLRX2,PNP
PRPP Biosynthesis I	1.13E00	5E-01	NA	PRPS2,PRPS1
Spermine and Spermidine Degradation I	1.13E00	5E-01	NA	SMOX,PAOX

dTMP De Novo Biosynthesis	1.13E00	5E-01	NA	SHMT1,SHMT2
PDGF Signaling	1.12E00	1.82E-01	2.673	MYC,SRC,INPP5B,TYK2,SPHK2,SPHK1,PIK3R5,SRF,KRAS,EIF2AK2,JAK2,STAT3,STAT1,MAP2K1
MSP-RON Signaling Pathway	1.11E00	2.05E-01	NA	TLR2,ITGAM,CCL2,CSF1,IL12B,PIK3R5,JAK2,NOS2,TNF
Urate Biosynthesis/Inosine 5'-phosphate Degradation	1.1E00	2.86E-01	NA	NT5C3A,IMPDH2,XDH,PNP
Aldosterone Signaling in Epithelial Cells	1.09E00	1.62E-01	0.632	PLCB2,DNAJB12,DNAJC9,SGK1,ITPR2,DNAJB4,DNAJC19,PIK3R5,DNAJC1,KRAS,PIP5K1B,DNAJB9,HSPA5,DNAJB14,PLCD3,HSP90B1,DUSP1,HSP90AA1,HSPB7,PRKCH,DNAJB1,MAP2K1,HSPA4L,AHCY
Bladder Cancer Signaling	1.07E00	1.76E-01	NA	DAPK1,TFDP1,SUV39H1,MMP13,MDM2,KRAS,CCND1,MYC,VEGFA,THBS1,E2F1,CDKN1A,FGF11,MAP2K1,RASSF1
Prostanoid Biosynthesis	1.07E00	3.33E-01	NA	PTGS1,PTGS2,TBXAS1
Dolichyl-diphosphooligosaccharide Biosynthesis	1.07E00	3.33E-01	NA	ALG5,ALG6,DPAGT1
UDP-N-acetyl-D-galactosamine Biosynthesis II	1.07E00	3.33E-01	NA	HK1,GNPDA1,GALE
Myc Mediated Apoptosis Signaling	1.07E00	1.9E-01	NA	MYC,IGF1,CASP3,IGF1R,APAF1,PIK3R5,BID,KRAS,SFN,FAS,BCL2
Prolactin Signaling	1.05E00	1.81E-01	0.905	MYC,SOCS3,SOCS1,PIK3R5,PRKCH,NMI,KRAS,JAK2,CEBPB,STAT3,STAT1,MAP2K1,IRF1
Corticotropin Releasing Hormone Signaling	1.04E00	1.68E-01	-0.775	PRKACB,RAP1B,GUCY2C,ITPR2,PTCH1,PRKAR2A,VEGFA,PRKAG2,SMO,PRKACA,MEF2C,PRKCH,JUND,PTGS2,NOS2,MAP2K1,ADCY7,Gucy2g
Granzyme A Signaling	1.01E00	2.67E-01	NA	Hist1h1e,HIST1H1D,H1F0,APEX1
Adenosine Nucleotides Degradation II	1.01E00	2.67E-01	NA	NT5C3A,XDH,PNP,ADA
IGF-1 Signaling	1.01E00	1.7E-01	-1.604	PRKACB,SOCS1,SOCS3,IGFBP4,SRF,PIK3R5,PRKAR2A,KRAS,STAT3,JAK2,IGF1,IGF1R,PRKACA,PRKAG2,SFN,MAP2K1

Ethanol Degradation IV	9.97E-01	2.38E-01	NA	ALDH1A2,CAT,ACSS2,ACSS1,ACSL1
tRNA Splicing	9.64E-01	2.06E-01	NA	PDE2A,PDE7B,PDE10A,PDE1B,PDE8B,PDE4B,APEX1
Histidine Degradation VI	9.56E-01	3E-01	NA	MICAL1,MICAL2,MICAL3
Glycine Betaine Degradation	9.56E-01	3E-01	NA	SARDH,SHMT1,SHMT2
Protein Citrullination	9.43E-01	4E-01	NA	PADI3,PADI2
Tetrahydrofolate Salvage from 5,10-methenyltetrahydrofolate	9.43E-01	4E-01	NA	MTHFD1,GART
Ceramide Biosynthesis	9.43E-01	4E-01	NA	SPTLC2,KDSR
Adenine and Adenosine Salvage III	9.43E-01	4E-01	NA	PNP,ADA
Purine Ribonucleosides Degradation to Ribose-1-phosphate	9.43E-01	4E-01	NA	PNP,ADA
Polyamine Regulation in Colon Cancer	9.28E-01	2.27E-01	NA	MYC,PSME1,MXD1,PSME2,KRAS
IL-17A Signaling in Gastric Cells	9.28E-01	2.27E-01	NA	CXCL10,RELA,CCL5,NFKB1,TNF
Breast Cancer Regulation by Stathmin1	9.27E-01	1.52E-01	NA	PRKACB,PLCB2,CAMK1,ARHGEF7,PIK3R5,ARHGEF1,KRAS,GNG7,E2F5,GNA13,MAP2K1,GNG12,E2F2,ARHGEF4,ARHGEF12,ITPR2,PRKAR2A,CDK1,CCNE1,TUBA1A,PPP2R4,CDKN1A,E2F1,PRKACA,PRKAG2,PRKCH,CDKN1B,ADCY7
Xanthine and Xanthosine Salvage	9.17E-01	1E00	NA	PNP
Histamine Biosynthesis	9.17E-01	1E00	NA	HDC
Adenine and Adenosine Salvage VI	9.17E-01	1E00	NA	ADK

UDP-N-acetyl-D-galactosamine Biosynthesis I	9.17E-01	1E00	NA	GALE
Systemic Lupus Erythematosus Signaling	8.88E-01	1.52E-01	NA	IL1A,HLA-A,PIK3R5,KRAS,IL6,FCGR1A,NFATC1,HLA-G,CD28,IL36G,LSM3,FCGR3A/FCGR3B,PRPF38A,FCGR2A,IL6R,TLR9,SNRNP35,CD40,IL1RN,CD72,CREM,NFATC2,IL1B,LSM5,TNF,HLA-E
Ethanol Degradation II	8.86E-01	2.07E-01	NA	ADH7,ALDH1A2,ACSS2,ACSS1,ADHFE1,ACSL1
α-Adrenergic Signaling	8.77E-01	1.67E-01	0.905	PRKACB,ITPR2,PRKAR2A,SLC8A2,KRAS,GNG7,GYS1,PRKACA,PRKAG2,PRKCH,MAP2K1,ADCY7,GNG12,ADRA1A
Wnt/Ca+ pathway	8.76E-01	1.79E-01	0.000	PLCD3,RELA,PLCB2,FZD4,SMO,NFATC2,FZD5,NFKB2,NFKB1,NFATC1
Antiproliferative Role of Somatostatin Receptor 2	8.74E-01	1.75E-01	0.000	RAP1B,SRC,GUCY2C,CDKN1A,PIK3R5,KRAS,CDKN1B,Gucy2g,MAP2K1,GNG12,GNG7
Oleate Biosynthesis II (Animals)	8.57E-01	2.73E-01	NA	FADS6,CYB5A,FADS1
Oxidative Ethanol Degradation III	8.5E-01	2.35E-01	NA	ALDH1A2,ACSS2,ACSS1,ACSL1
Gα12/13 Signaling	8.45E-01	1.58E-01	0.775	RELA,SRC,NFKBIE,GNA12,PIK3R5,ARHGEF1,IKBKE,KRAS,NFKB2,NFKB1,BTK,IKBKG,LPAR6,NFKBIA,MEF2C,GNA13,NFKBIB,MAP2K1
Endothelin-1 Signaling	8.37E-01	1.51E-01	1.606	SRC,PLCB2,CASP3,GUCY2C,ITPR2,GNA12,PTGS1,PIK3R5,KRAS,CASP4,PLA2G7,PLD4,MYC,HMOX1,PLCD3,EDN1,ECE2,PLA2G5,CASP1,PRKCH,PTGS2,GNA13,NOS2,ADCY7,CASP7
D-myo-inositol-5-phosphate Metabolism	8.22E-01	1.53E-01	NA	SOCS3,PLCB2,PTPN2,PPFIA3,PPFIBP2,MDP1,PPM1K,DUSP14,PLD4,CDC25B,ITPK1,PLCD3,PTPRJ,PPP2R4,DUSP1,DUSP10,PALD1,EYA4,NUDT4,CDC25A,DUSP16
Reelin Signaling in Neurons	8.13E-01	1.65E-01	NA	SRC,APOE,ARHGEF4,ARHGEF12,ITGA6,PIK3R5,ITGA5,ARHGEF1,FRK,APP,CDK5,CNR2,HCK
Glutathione-mediated Detoxification	8.06E-01	2.08E-01	NA	GSTM1,MGST2,GSTM5,ANPEP,MGST3
Non-Small Cell Lung Cancer Signaling	8.05E-01	1.69E-01	0.378	TFDP1,ITPR2,SUV39H1,E2F1,PIK3R5,KRAS,RASSF5,RXRA,CCND1,MAP2K1,RASSF1

Semaphorin Signaling in Neurons	8.04E-01	1.76E-01	NA	MET,PLXNA1,ARHGEF12,RHOQ,CDK5,RHOB,RHOC,RHOU,RHOJ
Zymosterol Biosynthesis	8.02E-01	3.33E-01	NA	TM7SF2,LBR
GDP-glucose Biosynthesis	8.02E-01	3.33E-01	NA	HK1,PGM1
GNRH Signaling	7.81E-01	1.53E-01	-2.524	PRKACB,MAP2K6,RELA,MAP3K14,SRC,PLCB2,ITPR2,PRKAR2A,KRAS,NFKB2,NFKB1,MAP3K12,PRKACA,PRKAG2,MAP3K8,MAP2K3,PRKCH,MAP2K1,ADCY7
Leptin Signaling in Obesity	7.78E-01	1.64E-01	1.342	PRKACB,PLCD3,SOCS3,PLCB2,PIK3R5,PRKAR2A,PRKACA,PRKAG2,STAT3,JAK2,ADCY7,MAP2K1
Choline Biosynthesis III	7.71E-01	2.5E-01	NA	PLD4,HMOX1,CEPT1
BER pathway	7.71E-01	2.5E-01	NA	POLE,APEX1,PARP1
Actin Nucleation by ARP-WASP Complex	7.68E-01	1.73E-01	1.000	RHOQ,RHOB,RHOC,GNA12,BAIAP2,RHOU,ITGA5,RHOJ,KRAS
Gαi Signaling	7.49E-01	1.53E-01	1.387	PRKACB,SRC,FPR2,PRKAR2A,KRAS,STAT3,GNG7,XCR1,P2RY13,CNR2,RGS10,S1PR1,PRKACA,PRKAG2,P2RY12,ADCY7,HCAR2,GNG12
Melatonin Signaling	7.42E-01	1.64E-01	-1.508	PRKACB,MAP2K6,PLCD3,PLCB2,RORA,PRKAR2A,PRKACA,PRKAG2,MAP2K3,PRKCH,MAP2K1
Growth Hormone Signaling	7.42E-01	1.64E-01	-0.333	SOCS1,SOCS3,IGF1,IGF1R,SRF,CEBPA,PIK3R5,PRKCH,STAT3,JAK2,STAT1
Macropinocytosis Signaling	7.42E-01	1.64E-01	1.134	MET,MRC1,SRC,RAB5A,CSF1,PIK3R5,ITGA5,CD14,KRAS,PRKCH,ITGB7
Role of JAK2 in Hormone-like Cytokine Signaling	7.37E-01	1.88E-01	NA	SOCS1,SOCS3,TYK2,STAT3,JAK2,STAT1
Thyroid Cancer Signaling	7.31E-01	1.79E-01	NA	CXCL10,MYC,GDNF,KRAS,RXRA,CCND1,MAP2K1
GPCR-Mediated Integration of Enteroendocrine Signaling Exemplified by an L Cell	7.12E-01	1.62E-01	NA	PRKACB,PLCD3,PLCB2,ADRB1,ITPR2,SSTR5,PRKAR2A,PRKACA,PRKAG2,ADCY7,ADRB2
D-myo-inositol (1,4,5)-Trisphosphate	7.01E-01	1.92E-01	NA	PLD4,PLCD3,PLCB2,PI4K2B,PIP5K1B

Biosynthesis				
Fatty Acid Activation	6.95E-01	2.31E-01	NA	ACSBG1,ACSL5,ACSL1
Cholesterol Biosynthesis I	6.95E-01	2.31E-01	NA	TM7SF2,SC5D,LBR
Cholesterol Biosynthesis II (via 24,25-dihydrolanosterol)	6.95E-01	2.31E-01	NA	TM7SF2,SC5D,LBR
Cholesterol Biosynthesis III (via Desmosterol)	6.95E-01	2.31E-01	NA	TM7SF2,SC5D,LBR
Inhibition of Angiogenesis by TSP1	6.93E-01	1.82E-01	1.342	VEGFA,TGFBR2,CD47,CASP3,THBS1,KDR
Airway Pathology in Chronic Obstructive Pulmonary Disease	6.91E-01	2.86E-01	NA	CXCL3,TNF
Sphingosine and Sphingosine-1-phosphate Metabolism	6.91E-01	2.86E-01	NA	NAAA,SGPP1
Glucose and Glucose-1-phosphate Degradation	6.91E-01	2.86E-01	NA	HK1,PGM1
D-myo-inositol (1,4,5,6)-Tetrakisphosphate Biosynthesis	6.83E-01	1.49E-01	NA	SOCS3,PTPN2,PPFIA3,PPFIBP2,MDP1,PPM1K,DUSP14,CDC25B,ITPK1,PTPRJ,PPP2R4,DUSP1,DUSP10,PALD1,EYA4,NUDT4,CDC25A,DUSP16
D-myo-inositol (3,4,5,6)-tetrakisphosphate Biosynthesis	6.83E-01	1.49E-01	NA	SOCS3,PTPN2,PPFIA3,PPFIBP2,MDP1,PPM1K,DUSP14,CDC25B,ITPK1,PTPRJ,PPP2R4,DUSP1,DUSP10,PALD1,EYA4,NUDT4,CDC25A,DUSP16

Estrogen-Dependent Breast Cancer Signaling	6.75E-01	1.61E-01	0.333	SRC,RELA,IGF1,IGF1R,PIK3R5,KRAS,NFKB2,NFKB1,CCND1,HSD17B2
Regulation of Cellular Mechanics by Calpain Protease	6.67E-01	1.64E-01	1.134	CCNA2,SRC,CCNE1,TLN2,ITGA5,KRAS,CDKN1B,CCND1,CDK1
ILK Signaling	6.5E-01	1.42E-01	1.528	MAP2K6,MYH10,FLNB,RELA,CASP3,LIMS2,RHOC,PIK3R5,VIM,RHOJ,NFKB2,NFKB1,CCND1,ITGB7,VEGFA,MYC,DOCK1,RHOQ,RHOB,PPP2R4,RHOU,PTGS2,NOS2,TNF,TESK1
Asparagine Degradation I	6.43E-01	5E-01	NA	ASRGL1
Guanine and Guanosine Salvage I	6.43E-01	5E-01	NA	PNP
L-DOPA Degradation	6.43E-01	5E-01	NA	COMT
S-methyl-5-thio-α-D-ribose 1-phosphate Degradation	6.43E-01	5E-01	NA	MRI1
Palmitate Biosynthesis I (Animals)	6.43E-01	5E-01	NA	FASN
Spermidine Biosynthesis I	6.43E-01	5E-01	NA	SRM
Fatty Acid Biosynthesis Initiation II	6.43E-01	5E-01	NA	FASN
Adenine and Adenosine Salvage I	6.43E-01	5E-01	NA	PNP
Formaldehyde Oxidation II (Glutathione-dependent)	6.43E-01	5E-01	NA	ESD
Glioma Invasiveness Signaling	6.36E-01	1.61E-01	1.000	RHOQ,RHOB,RHOC,PIK3R5,RHOU,RHOJ,KRAS,PLAU,TIMP2
Superpathway of Inositol Phosphate Compounds	6.29E-01	1.41E-01	NA	SOCS3,PLCB2,PTPN2,PPFIA3,PPFIBP2,PIK3R5,MDP1,PIP5K1B,PPM1K,CDC25B,PLCD3,PTPRJ,ITPKB,DUSP10,INPP5B,EYA4,PALD1,PI4K2B,NUDT4,CDC25A,DUSP14,PLD4,ITPK1,DUSP1,PPP2R4,DUSP16

DNA Double-Strand Break Repair by Homologous Recombination	6.29E-01	2.14E-01	NA	RAD51,POLA1,BRCA2
G Beta Gamma Signaling	6.26E-01	1.51E-01	-1.941	PRKACB,SRC,GNA12,PRKAR2A,KRAS,GNG7,BTK,KCNJ9,PRKACA,PRKAG2,PRKCH,GNA13,GNG12
Melanoma Signaling	6.2E-01	1.67E-01	NA	E2F1,CDKN1A,PIK3R5,MDM2,KRAS,CCND1,MAP2K1
Hematopoiesis from Pluripotent Stem Cells	6.14E-01	1.71E-01	NA	IL1A,CSF1,IL12B,IL6,CSF3,IL7
Stearate Biosynthesis I (Animals)	6.14E-01	1.71E-01	NA	ACSBG1,FASN,ACSL5,ACOT9,ACSL1,ELOVL6
Thrombin Signaling	6.13E-01	1.4E-01	1.633	RELA,PLCB2,CAMK1,PIK3R5,ARHGEF1,KRAS,NFKB1,GNG7,Ppp1r12b,PLCD3,RHOB,RHOU,GNA13,MAP2K1,GNG12,SRC,ARHGEF4,ARHGEF12,RHOC,ITPR2,GNA12,RHOJ,NFKB2,RHOQ,PRKCH,ADCY7
Tryptophan Degradation III (Eukaryotic)	6.13E-01	1.9E-01	NA	KMO,ACAT1,HADH,KYNU
3-phosphoinositide Biosynthesis	6.03E-01	1.42E-01	NA	SOCS3,PTPN2,PPFIA3,PPFIBP2,PIK3R5,MDP1,PIP5K1B,PPM1K,DUSP14,CDC25B,ITPK1,PTPRJ,DUSP1,PPP2R4,DUSP10,PALD1,PI4K2B,EYA4,NUDT4,CDC25A,DUSP16
ERK/MAPK Signaling	6.01E-01	1.4E-01	0.600	PRKACB,RAP1B,SRC,RAPGEF1,DUSP6,PRKAR2A,SRF,PIK3R5,ITGA5,KRAS,STAT3,BCAR1,NFATC1,MYC,DOCK1,TLN2,DUSP1,PPP2R4,ETS2,PLA2G5,PRKAG2,PRKACA,HSPB7,STAT1,MAP2K1
Sucrose Degradation V (Mammalian)	6E-01	2.5E-01	NA	KHK,TPI1
Phosphatidylethanolamine Biosynthesis II	6E-01	2.5E-01	NA	CEPT1,ETNK1
Tryptophan Degradation to 2-amino-3-carboxymuconate Semialdehyde	6E-01	2.5E-01	NA	KMO,KYNU
Ketolysis	6E-01	2.5E-01	NA	ACAT1,OXCT1

Nur77 Signaling in T Lymphocytes	5.96E-01	1.6E-01	NA	HDAC9,CD28,CASP3,APAF1,PPP3CC,RXRA,NFATC1,BCL2
Telomerase Signaling	5.93E-01	1.47E-01	1.667	MYC,HDAC9,HSP90B1,HDAC4,ETS2,PPP2R4,CDKN1A,E2F1,DKC1,PIK3R5,HSP90AA1,KRAS,MAP2K1,HDAC5
Hypoxia Signaling in the Cardiovascular System	5.91E-01	1.54E-01	2.449	VEGFA,HSP90B1,NFKBIA,EDN1,NFKBIE,NQO1,HSP90AA1,UBE2E2,MDM2,NFKBIB
CXCR4 Signaling	5.86E-01	1.41E-01	0.943	SRC,PLCB2,RHOC,ITPR2,CXCR4,GNA12,PIK3R5,RHOJ,KRAS,BCAR1,GNG7,DOCK1,RHOQ,RHOB,RHOU,PRKCH,GNA13,ELMO2,MAP2K1,ADCY7,GNG12
Autoimmune Thyroid Disease Signaling	5.77E-01	1.67E-01	NA	HLA-G,CD28,CD40,HLA-A,FAS,HLA-E
GPCR-Mediated Nutrient Sensing in Enteroendocrine Cells	5.45E-01	1.46E-01	NA	PRKACB,PLCD3,PLCB2,FFAR4,ITPR2,PRKAR2A,PRKACA,PRKAG2,PRKCH,ADCY7,GNG12,GNG7
Renal Cell Carcinoma Signaling	5.4E-01	1.49E-01	1.342	VEGFA,MET,RAPGEF1,SLC2A1,PIK3R5,Ubb,KRAS,FH,UBC,MAP2K1
Fcγ Receptor-mediated Phagocytosis in Macrophages and Monocytes	5.37E-01	1.44E-01	0.832	SRC,FCGR2A,FYB,FCGR1A,PLD4,DOCK1,HMOX1,TLN2,SYK,HCK,PRKCH,LCP2,FCGR3A/FCGR3B
Germ Cell-Sertoli Cell Junction Signaling	5.36E-01	1.38E-01	NA	MAP2K6,MAP3K14,SRC,RHOC,ITGA6,PIK3R5,RHOJ,KRAS,GSN,BCAR1,TGFBR2,MAP3K12,RHOQ,TUBA1A,RHOB,RHOU,MAP3K8,MAP2K3,TNF,MAP2K1,PVRL2
Cellular Effects of Sildenafil (Viagra)	5.17E-01	1.39E-01	NA	PRKACB,MYH10,PDE2A,PLCB2,CACNA1D,GUCY2C,ITPR2,PRKAR2A,PDE4B,Ppp1r12b,PLCD3,PRKG1,PDE1B,PRKACA,PRKAG2,CACNG8,ADCY7
Extrinsic Prothrombin Activation Pathway	5.17E-01	1.88E-01	NA	PROS1,F13A1,THBD

Methionine Degradation I (to Homocysteine)	5.17E-01	1.88E-01	NA	SUV39H2,PRMT5,AHCY
Virus Entry via Endocytic Pathways	5.02E-01	1.43E-01	NA	B2M,FLNB,SRC,HLA-A,CLTB,ITGA6,PIK3R5,ITGA5,TFRC,KRAS,PRKCH,ITGB7
Eicosanoid Signaling	5.01E-01	1.48E-01	NA	LTA4H,PTGIR,PTGS1,PLA2G5,FPR2,PTGS2,TBXAS1,PLA2G7,PTGER4
Superpathway of Methionine Degradation	4.97E-01	1.61E-01	NA	PCCA,SUV39H2,PRMT5,MTR,AHCY
Diphthamide Biosynthesis	4.94E-01	3.33E-01	NA	DPH5
Trehalose Degradation II (Trehalase)	4.94E-01	3.33E-01	NA	HK1
Tetrahydrobiopterin Biosynthesis I	4.94E-01	3.33E-01	NA	GCH1
D-glucuronate Degradation I	4.94E-01	3.33E-01	NA	CRYL1
Methionine Salvage II (Mammalian)	4.94E-01	3.33E-01	NA	MTR
Thiosulfate Disproportionation III (Rhodanese)	4.94E-01	3.33E-01	NA	TST
1D-myo-inositol Hexakisphosphate Biosynthesis V (from Ins(1,3,4)P3)	4.94E-01	3.33E-01	NA	ITPK1
Tetrahydrobiopterin Biosynthesis II	4.94E-01	3.33E-01	NA	GCH1
4-aminobutyrate Degradation I	4.94E-01	3.33E-01	NA	SUCLG2
N-acetylglucosamine Degradation I	4.94E-01	3.33E-01	NA	GNPDA1

Caveolar-mediated Endocytosis Signaling	4.94E-01	1.45E-01	NA	B2M,FLNB,SRC,RAB5A,ITGAM,HLA-A,ITGA6,ITGA5,ITGB7,ITGAX
Phospholipases	4.86E-01	1.48E-01	NA	PLD4,PLCD3,HMOX1,PLCB2,PLA2G5,LIPG,PLA1A,PLA2G7
IL-22 Signaling	4.83E-01	1.67E-01	1.000	SOCS3,TYK2,STAT3,STAT1
Gluconeogenesis I	4.83E-01	1.67E-01	NA	PGK1,ENO3,PGAM1,MDH1
Signaling by Rho Family GTPases	4.81E-01	1.32E-01	2.353	RELA,ARHGEF7,PIK3R5,ARHGEF1,CDC42EP2,PIP5K1B,NFKB1,NOX1,GNG7,RHOB,CIT,BAIAP2,CYBB,RHOU,GNA13,MAP2K1,GNG12,ARHGEF4,ARHGEF12,RHOC,GNA12,CDC42EP3,ITGA5,VIM,RHOJ,NFKB2,MAP3K12,RHOQ,NCF2,MSN
ERK5 Signaling	4.78E-01	1.45E-01	0.707	MYC,SRC,SGK1,GNA12,KRAS,MAP3K8,MEF2C,GNA13,SFN
IL-4 Signaling	4.72E-01	1.43E-01	NA	SOCS1,IL4R,IL13RA1,INPP5B,TYK2,PIK3R5,NFATC2,KRAS,JAK2,NFATC1
Glutathione Redox Reactions I	4.7E-01	1.76E-01	NA	GSR,MGST2,MGST3
Mitochondrial L-carnitine Shuttle Pathway	4.7E-01	1.76E-01	NA	ACSBG1,ACSL5,ACSL1
Noradrenaline and Adrenaline Degradation	4.64E-01	1.56E-01	NA	ADH7,SMOX,COMT,ALDH1A2,ADHFE1
Fatty Acid β-oxidation I	4.64E-01	1.56E-01	NA	ACSBG1,ACSL5,ACAA2,ACSL1,HADH
NAD Phosphorylation and Dephosphorylation	4.62E-01	2E-01	NA	ACP6,Acp5
Thrombopoietin Signaling	4.61E-01	1.45E-01	1.414	MYC,PIK3R5,KRAS,PRKCH,STAT3,JAK2,STAT1,MAP2K1
ErbB2-ErbB3 Signaling	4.38E-01	1.43E-01	1.134	MYC,TYK2,PIK3R5,KRAS,STAT3,CDKN1B,CCND1,MAP2K1
BMP signaling pathway	4.3E-01	1.39E-01	-0.632	PRKACB,RELA,PRKAR2A,PRKACA,PRKAG2,KRAS,NFKB2,NFKB1,MAP2K1,SMURF1
Cysteine Biosynthesis III (mammalia)	4.27E-01	1.67E-01	NA	SUV39H2,PRMT5,AHCY
B Cell Development	4.12E-01	1.54E-01	NA	IL7R,SPN,CD40,IL7

Leukotriene Biosynthesis	4.08E-01	1.82E-01	NA	LTA4H,MGST2
eNOS Signaling	4.07E-01	1.31E-01	-1.387	PRKACB,CASP3,ITPR2,PIK3R5,PRKAR2A,HSPA5,VEGFA,CCNA2,LPAR6,AQP9,HSP90B1,PRKG1,PRKACA,PRKAG2,HSP90AA1,PRKCH,KDR,ADCY7
Sperm Motility	4.07E-01	1.33E-01	0.775	PRKACB,PDE2A,PLCB2,ITPR2,PRKAR2A,PDE4B,PLA2G7,FRK,PLCD3,PRKG1,PLA2G5,PDE1B,PRKACA,PRKAG2,PRKCH
Airway Inflammation in Asthma	3.95E-01	2.5E-01	NA	TNF
Eumelanin Biosynthesis	3.95E-01	2.5E-01	NA	DDT
Methylmalonyl Pathway	3.95E-01	2.5E-01	NA	PCCA
Glutathione Redox Reactions II	3.95E-01	2.5E-01	NA	GSR
Melatonin Degradation II	3.95E-01	2.5E-01	NA	SMOX
NAD Biosynthesis III	3.95E-01	2.5E-01	NA	NAMPT
N-acetylglucosamine Degradation II	3.95E-01	2.5E-01	NA	GNPDA1
Molybdenum Cofactor Biosynthesis	3.95E-01	2.5E-01	NA	MOCS2
SAPK/JNK Signaling	3.92E-01	1.33E-01	-0.577	MINK1,MAP3K12,DAXX,TRAF2,GADD45A,DUSP10,GNA12,PIK3R5,KRAS,GNA13,GNG7,NFATC1
1D-myo-inositol Hexakisphosphate Biosynthesis II (Mammalian)	3.89E-01	1.58E-01	NA	ITPK1,ITPKB,INPP5B
Intrinsic Prothrombin Activation Pathway	3.81E-01	1.48E-01	NA	PROS1,F13A1,THBD,COL18A1
Superpathway of Cholesterol Biosynthesis	3.81E-01	1.48E-01	NA	ACAT1,TM7SF2,SC5D,LBR
Huntington's Disease Signaling	3.8E-01	1.27E-01	-2.000	PLCB2,REST,SGK1,CLTB,PIK3R5,Ubb,CASP4,HSPA5,GNG7,CDK5,IGF1,IGF1R,CASP1,DNAJB1,BET1L,GNG12,HDAC9,HDAC4,CASP3,PSME2,APAF1,HIP1,HDAC5,PSME1,PRKCH,NCOR2,UBC,CASP7

Coagulation System	3.77E-01	1.43E-01	-1.342	PROS1,F13A1,PLAU,THBD,SERPINE1
Role of Tissue Factor in Cancer	3.77E-01	1.31E-01	NA	SRC,CASP3,GNA12,ITGA6,PIK3R5,MMP13,KRAS,JAK2,FRK,VEGFA,CSF1,HCK,IL1B,GNA13
Melanocyte Development and Pigmentation Signaling	3.75E-01	1.33E-01	-1.265	PRKACB,SRC,PAX3,PIK3R5,PRKAR2A,PRKACA,PRKAG2,KRAS,ADCY7,MAP2K1,BCL2
CNTF Signaling	3.74E-01	1.37E-01	1.890	TYK2,PIK3R5,KRAS,STAT3,JAK2,STAT1,MAP2K1
Heparan Sulfate Biosynthesis	3.74E-01	1.37E-01	NA	HS6ST1,EXT2,XYLT2,UST,CHST10,CHST12,EXTL1
Sertoli Cell-Sertoli Cell Junction Signaling	3.73E-01	1.28E-01	NA	PRKACB,SPTBN2,MAP3K14,SRC,PRKAR2A,ITGA5,SPTA1,KRAS,BCAR1,MAP3K12,TJP3,PRKG1,TUBA1A,PRKACA,PRKAG2,MAP3K8,MAP2K3,PVRL1,NOS2,TNF,MAP2K1,PVRL2
Clathrin-mediated Endocytosis Signaling	3.62E-01	1.27E-01	NA	APOE,SRC,SH3BP4,CLTB,PIK3R5,ITGA5,Ubb,MDM2,PPP3CC,HIP1,ITGB7,VEGFA,MET,LYZ,RAB5A,LDLR,IGF1,DAB2,TFRC,FGF11,UBC,HGS
Glutaryl-CoA Degradation	3.61E-01	1.67E-01	NA	ACAT1,HADH
DNA Methylation and Transcriptional Repression Signaling	3.54E-01	1.5E-01	NA	MECP2,MTA1,SAP30
Serotonin Degradation	3.54E-01	1.35E-01	NA	EXT2,ADH7,UGT1A6,SMOX,ALDH1A2,ADHFE1,B4GAT1
Heparan Sulfate Biosynthesis (Late Stages)	3.53E-01	1.36E-01	NA	HS6ST1,EXT2,UST,CHST10,CHST12,EXTL1
Role of p14/p19ARF in Tumor Suppression	3.53E-01	1.43E-01	NA	NPM3,E2F1,PIK3R5,MDM2
autophagy	3.52E-01	1.39E-01	NA	ATG13,ATG12,SQSTM1,ATG16L1,BCL2
TGF-β Signaling	3.43E-01	1.29E-01	-1.265	MAP2K6,TGFBR2,IRF7,SMAD3,KRAS,MAP2K3,SERPINE1,MAP2K1,TGIF1,SMURF1,BCL2
Regulation of the Epithelial-Mesenchymal Transition Pathway	3.42E-01	1.26E-01	NA	MAP2K6,RELA,ADAM17,JAG2,SMAD3,TYK2,PIK3R5,KRAS,NFKB2,STAT3,ZEB1,JAK2,NFKB1,SMURF1,MET,TGFBR2,NOTCH2,FZD4,SMO,FZD5,FGF11,MAP2K3,MAP2K1

Calcium Signaling	3.38E-01	1.26E-01	-1.069	RAP1B,PRKACB,HDAC9,MYH10,CAMK1,HDAC4,LETM1,TNNI2,ITPR2,TNNC1,PRKAR2A,SLC8A2,PPP3CC,NFATC1,HDAC5,GRINA,Tpm4,PRKACA,PRKAG2,NFATC2,MEF2C
Rac Signaling	3.33E-01	1.27E-01	2.496	RELA,BAIAP2,NCF2,CYBB,PIK3R5,ITGA5,KRAS,NFKB2,PIP5K1B,NFKB1,MAP2K1,NOX1,IQGAP3
Cdc42 Signaling	3.29E-01	1.26E-01	2.121	B2M,SRC,FGD3,H2-T24,H2-T22,H2-T10,HLA-A,H2-K2/H2-Q9,ITGA5,CDC42EP2,Ppp1r12b,HLA-G,CDC42BPA,H2-M2,BAIAP2,HLA-E,IQGAP3
Notch Signaling	3.29E-01	1.35E-01	1.000	ADAM17,NOTCH2,JAG2,LFNG,DTX2
Neuregulin Signaling	3.28E-01	1.28E-01	-0.378	MYC,SRC,ADAM17,HSP90B1,CDK5,ITGA5,HSP90AA1,KRAS,PRKCH,CDKN1B,MAP2K1
Creatine-phosphate Biosynthesis	3.23E-01	2E-01	NA	CKB
Serine Biosynthesis	3.23E-01	2E-01	NA	PSAT1
Serotonin and Melatonin Biosynthesis	3.23E-01	2E-01	NA	TPH2
CMP-N-acetylneuraminate Biosynthesis I (Eukaryotes)	3.23E-01	2E-01	NA	GNE
Ceramide Degradation	3.23E-01	2E-01	NA	NAAA
2-oxobutanoate Degradation I	3.23E-01	2E-01	NA	PCCA
Citrulline-Nitric Oxide Cycle	3.23E-01	2E-01	NA	NOS2
Rapoport-Luebering Glycolytic Shunt	3.23E-01	2E-01	NA	PGAM1
Galactose Degradation I (Leloir Pathway)	3.23E-01	2E-01	NA	GALE
Glutamate Degradation III (via 4-aminobutyrate)	3.23E-01	2E-01	NA	SUCLG2
Gap Junction Signaling	3.22E-01	1.25E-01	NA	PRKACB,SRC,PLCB2,GUCY2C,ITPR2,PRKAR2A,PIK3R5,KRAS,PPP3CC,PLCD3,PRKG1,TUBA1A,ADRB1,PRKACA,PRKAG2,PRKCH,MAP2K1,ADCY7,Guc

				y2g
IL-3 Signaling	3.22E-01	1.29E-01	NA	RAPGEF1,PIK3R5,KRAS,PRKCH,PPP3CC,STAT3,JAK2,STAT1,MAP2K1
NAD biosynthesis II (from tryptophan)	3.2E-01	1.54E-01	NA	KMO,KYNU
tRNA Charging	3.07E-01	1.32E-01	NA	WARS,RARS2,MARS2,DARS2,VARS
Synaptic Long Term Depression	3.06E-01	1.24E-01	1.213	PLCB2,GUCY2C,ITPR2,GNA12,KRAS,PLA2G7,PLCD3,PRKG1,IGF1,PPP2R4,PLA2G5,IGF1R,PRKCH,GNA13,NOS2,MAP2K1,Gucy2g
NAD Salvage Pathway II	2.95E-01	1.36E-01	NA	NT5C3A,ACP6,Acp5
TCA Cycle II (Eukaryotic)	2.95E-01	1.36E-01	NA	IDH3A,MDH1,FH
NGF Signaling	2.94E-01	1.24E-01	1.387	RAP1B,MAP3K14,RELA,PIK3R5,IKBKE,KRAS,NFKB2,NFKB1,SMPD2,MAP3K12,IKBKG,MAP3K8,MAP2K1
RAN Signaling	2.85E-01	1.43E-01	NA	KPNA3,KPNA4
DNA Double-Strand Break Repair by Non-Homologous End Joining	2.85E-01	1.43E-01	NA	XRCC6,PARP1
Methylglyoxal Degradation III	2.85E-01	1.43E-01	NA	AKR7A2,AKR1B1
Vitamin-C Transport	2.85E-01	1.43E-01	NA	SLC2A1,TXNRD1
EGF Signaling	2.81E-01	1.25E-01	1.890	SRC,ITPR2,SRF,PIK3R5,STAT3,STAT1,MAP2K1
Neuropathic Pain Signaling In Dorsal Horn Neurons	2.77E-01	1.22E-01	-0.577	PRKACB,PLCD3,SRC,PLCB2,CAMK1,ITPR2,PIK3R5,PRKAR2A,PRKACA,PRKAG2,PRKCH,GRINA
Paxillin Signaling	2.77E-01	1.22E-01	0.000	DOCK1,SRC,TLN2,ITGAM,ARHGEF7,ITGA6,PIK3R5,ITGA5,KRAS,BCAR1,ITGB7,ITGAX
Chondroitin Sulfate Biosynthesis	2.75E-01	1.25E-01	NA	HS6ST1,XYLT2,UST,CHPF,CHST10,CHST12
FXR/RXR Activation	2.74E-01	1.22E-01	NA	APOE,IL36G,IL1A,C3,PCK2,SCARB1,CYP27A1,IL1RN,FASN,LPL,SERPINF1,IL1B,RXRA,TNF
14-3-3-mediated Signaling	2.74E-01	1.22E-01	1.265	SRC,PLCB2,STRADA,PIK3R5,VIM,KRAS,PLCD3,TRAF2,TUBA1A,PRKCH,CDKN1B,SFN,TNF,MAP2K1

Dopamine Degradation	2.69E-01	1.3E-01	NA	SMOX,COMT,ALDH1A2
Chondroitin and Dermatan Biosynthesis	2.69E-01	1.67E-01	NA	CHPF
Thioredoxin Pathway	2.69E-01	1.67E-01	NA	TXNRD1
Pentose Phosphate Pathway (Non-oxidative Branch)	2.69E-01	1.67E-01	NA	TALDO1
Glycerol Degradation I	2.69E-01	1.67E-01	NA	GK5
Acetyl-CoA Biosynthesis I (Pyruvate Dehydrogenase Complex)	2.69E-01	1.67E-01	NA	DLAT
Glycine Cleavage Complex	2.69E-01	1.67E-01	NA	GCSH
UDP-N-acetyl-D-glucosamine Biosynthesis II	2.69E-01	1.67E-01	NA	GFPT1
Salvage Pathways of Pyrimidine Deoxyribonucleotides	2.69E-01	1.67E-01	NA	TK1
Chondroitin Sulfate Biosynthesis (Late Stages)	2.67E-01	1.25E-01	NA	HS6ST1,UST,CHPF,CHST10,CHST12
Serotonin Receptor Signaling	2.67E-01	1.25E-01	NA	GCH1,SMOX,TPH2,SLC6A4,ADCY7
HIF1α Signaling	2.65E-01	1.21E-01	NA	VEGFA,EGLN1,SLC2A1,EDN1,PIK3R5,HSP90AA1,MMP13,MDM2,KRAS,NAA10,NOS2,APEX1
HER-2 Signaling in Breast Cancer	2.63E-01	1.22E-01	NA	CCNE1,CDKN1A,PIK3R5,MDM2,KRAS,PRKCH,CDKN1B,CCND1,ITGB7
G Protein Signaling Mediated by Tubby	2.58E-01	1.25E-01	NA	PLCB2,JAK2,GNG12,GNG7

Chondroitin Sulfate Degradation (Metazoa)	2.54E-01	1.33E-01	NA	GM2A,ARSB
FcγRIIB Signaling in B Lymphocytes	2.49E-01	1.22E-01	0.447	BLNK,BTK,SYK,PIK3R5,KRAS
Superpathway of D-myo-inositol (1,4,5)-trisphosphate Metabolism	2.45E-01	1.25E-01	NA	INPP1,ITPKB,INPP5B
CDP-diacylglycerol Biosynthesis I	2.27E-01	1.25E-01	NA	AGPAT4,TAMM41
Parkinson's Signaling	2.27E-01	1.25E-01	NA	UCHL1,CASP3
Phosphatidylcholine Biosynthesis I	2.26E-01	1.43E-01	NA	CEPT1
Glycoaminoglycan-protein Linkage Region Biosynthesis	2.26E-01	1.43E-01	NA	XYLT2
Aspartate Degradation II	2.26E-01	1.43E-01	NA	MDH1
Role of IL-17A in Psoriasis	1.91E-01	1.25E-01	NA	CXCL3
Histidine Degradation III	1.91E-01	1.25E-01	NA	MTHFD1
Sphingomyelin Metabolism	1.91E-01	1.25E-01	NA	SMPD2

Note: The data were analyzed using QIAGEN's Ingenuity® Pathway Analysis (IPA®, QIAGEN Redwood City, www.qiagen.com/ingenuity) for associated gene functions and pathways, as well as to predict gene interaction networks. (This note is also applied for Supplementary Table S5 and Supplementary Table S6).

Supplementary Table S5. Canonical pathways respond to AGE treatment (LPS+AGE vs. LPS).

Ingenuity Canonical Pathways	-log(p-value)	Ratio	z-score	Molecules
Granulocyte Adhesion and Diapedesis	7.27E00	9.6E-02	NA	VCAM1,IL1A,C5AR1,MMP13,SDC3,CSF3,IL18RAP,CXCL10,CXCL3,IL36G,ITGAM,CCL2,Ccl8,IL1B,Cxcl3,MMP12,MMP9
Communication	6.91E00	1.35E-01	NA	CXCL10,IL36G,IL1A,CD40,HLA-

between Innate and Adaptive Immune Cells				A,IL15,CD86,IL1B,TNFRSF13B,TLR3,CSF2,HLA-E
Role of Pattern Recognition Receptors in Recognition of Bacteria and Viruses	6.88E00	1.11E-01	-1.508	IFIH1,IRF7,IL1A,C5AR1,C3,CASP1,Oas1b,IL1B,CLEC6A,C1QC,TLR3,C1QB,CSF2,ATM
Interferon Signaling	5.35E00	1.94E-01	-2.449	IFIT3,IFIT1,IFITM3,STAT2,TAP1,IRF1,ISG15
Crosstalk between Dendritic Cells and Natural Killer Cells	5.12E00	1.12E-01	NA	IL15RA,CD40,HLA-A,CD69,IL15,CD86,TNFSF10,TLR3,CSF2,HLA-E
Dendritic Cell Maturation	5.1E00	7.91E-02	-1.387	IL36G,IL1A,CD40,FCGR2A,HLA-A,IL15,IL1B,CD86,STAT2,TLR3,CSF2,FCGR1A,FCGR3A/FCGR3B,ATM
LXR/RXR Activation	4.69E00	9.09E-02	-1.000	SCD,IL36G,IL1A,C3,CCL2,FASN,IL1B,TLR3,NOS2,MMP9,IL18RAP
Altered T Cell and B Cell Signaling in Rheumatoid Arthritis	4.34E00	1.02E-01	NA	IL36G,IL1A,CD40,IL15,CD86,IL1B,TNFRSF13B,TLR3,CSF2
Role of Macrophages, Fibroblasts and Endothelial Cells in Rheumatoid Arthritis	4.23E00	5.74E-02	NA	IL1A,VCAM1,C5AR1,IL15,MMP13,FCGR1A,IL18RAP,IL36G,CCL2,IL1B,TLR3,NOS2,CSF2,FCGR3A/FCGR3B,ATM,FZD7,ADAMTS4
Differential Regulation of Cytokine Production in Intestinal Epithelial Cells by IL-17A and IL-17F	4.21E00	2.17E-01	NA	IL1A,CCL2,IL1B,CSF3,CSF2
Agranulocyte Adhesion and Diapedesis	4.14E00	6.88E-02	NA	CXCL10,CXCL3,IL36G,VCAM1,IL1A,C5AR1,CCL2,Ccl8,IL1B,MMP13,Cxcl3,MMP12,MMP9
Role of Cytokines in Mediating Communication between Immune Cells	4.1E00	1.27E-01	NA	IL36G,IL1A,IL15,IL27,IL1B,CSF3,CSF2
TREM1 Signaling	4.05E00	1.07E-01	NA	CXCL3,CCL2,CD40,CASP1,CD86,IL1B,TLR3,CSF2
Role of Hypercytokinemia/hyperchem	3.75E00	1.36E-01	NA	CXCL10,IL36G,IL1A,CCL2,IL15,IL1B

okinemia in the Pathogenesis of Influenza				
Graft-versus-Host Disease Signaling	3.54E00	1.25E-01	NA	IL36G,IL1A,HLA-A,CD86,IL1B,HLA-E
Differential Regulation of Cytokine Production in Macrophages and T Helper Cells by IL-17A and IL-17F	3.49E00	2.22E-01	NA	CCL2,IL1B,CSF3,CSF2
Antigen Presentation Pathway	3.19E00	1.35E-01	NA	PSMB9,HLA-A,TAP1,TAPBP,HLA-E
Complement System	3.19E00	1.35E-01	-1.000	ITGAM,C3,C5AR1,C1QC,C1QB
Activation of IRF by Cytosolic Pattern Recognition Receptors	2.9E00	9.52E-02	-1.633	IFIH1,IRF7,CD40,STAT2,IFIT2,ISG15
Hematopoiesis from Multipotent Stem Cells	2.88E00	2.5E-01	NA	IL15,CSF3,CSF2
Role of IL-17F in Allergic Inflammatory Airway Diseases	2.84E00	1.14E-01	1.000	CXCL10,CCL2,MMP13,IL1B,CSF2
Aryl Hydrocarbon Receptor Signaling	2.83E00	6.43E-02	NA	CDKN2A,GSTA3,IL1A,MGST2,NQO1,CDKN1A,IL1B,DHFR,ATM
IL-15 Signaling	2.8E00	9.09E-02	NA	VCAM1,IL15RA,IL15,AXL,CSF2,ATM
Guanosine Nucleotides Degradation III	2.77E00	2.31E-01	NA	NT5C3A,PNP,NT5C
Urate Biosynthesis/Inosine 5'-phosphate Degradation	2.67E00	2.14E-01	NA	NT5C3A,PNP,NT5C
Atherosclerosis Signaling	2.58E00	6.45E-02	NA	IL36G,IL1A,VCAM1,CCL2,CD40,MMP13,IL1B,MMP9
Systemic Lupus Erythematosus Signaling	2.54E00	5.12E-02	NA	IL36G,IL1A,CD40,HLA-A,FCGR2A,CD86,IL1B,FCGR1A,FCGR3A/FCGR3B,HLA-E,ATM
Role of IL-17A in Arthritis	2.44E00	9.26E-02	NA	CXCL3,CCL2,MMP13,NOS2,ATM
Adenosine	2.42E00	1.76E-01	NA	NT5C3A,PNP,NT5C

Nucleotides Degradation II				
GADD45 Signaling	2.27E00	1.58E-01	NA	GADD45G,CDKN1A,ATM
Allograft Rejection Signaling	2.27E00	7.14E-02	NA	H2-T22,H2-T10,CD40,HLA-A,CD86,HLA-E
Purine Nucleotides Degradation II (Aerobic)	2.21E00	1.5E-01	NA	NT5C3A,PNP,NT5C
Bladder Cancer Signaling	2.19E00	6.9E-02	NA	CDKN2A,CDKN1A,MMP13,MMP12,MMP9,RASSF1
Glucocorticoid Receptor Signaling	2.17E00	4.36E-02	NA	CXCL3,VCAM1,CCL2,GTF2H4,CDKN1A,SLPI,IL1B,TAF7,CSF2,NOS2,FCGR1A,ATM
Hepatic Fibrosis / Hepatic Stellate Cell Activation	2.06E00	4.92E-02	NA	CXCL3,IL1A,VCAM1,CCL2,CD40,MMP13,IL1B,MMP9,IL18RAP
Airway Pathology in Chronic Obstructive Pulmonary Disease	2.03E00	2.5E-01	NA	CXCL3,MMP9
IL-10 Signaling	2.02E00	7.35E-02	NA	IL36G,IL1A,FCGR2A,IL1B,IL18RAP
p53 Signaling	1.95E00	6.12E-02	-1.342	CDKN2A,GADD45G,CDKN1A,PML,BIRC5,ATM
MSP-RON Signaling Pathway	1.95E00	8.7E-02	NA	ITGAM,CCL2,NOS2,ATM
Assembly of RNA Polymerase I Complex	1.92E00	2.22E-01	NA	TAF1A,POLR1B
Autoimmune Thyroid Disease Signaling	1.91E00	8.51E-02	NA	CD40,HLA-A,CD86,HLA-E
Cell Cycle: G2/M DNA Damage Checkpoint Regulation	1.85E00	8.16E-02	-1.000	CDKN2A,TOP2B,CDKN1A,ATM
Colorectal Cancer Metastasis Signaling	1.81E00	4.24E-02	0.632	MMP13,TLR3,MMP12,NOS2,ADCY7,BIRC5,MMP9,RALGDS,FZD7,ATM
Pancreatic Adenocarcinoma Signaling	1.8E00	5.66E-02	0.816	CDKN2A,CDKN1A,BIRC5,MMP9,RALGDS,ATM
Endothelin-1 Signaling	1.75E00	4.65E-02	-1.633	GNA15,PTGS1,CASP1,CASP4,ITPR1,NOS2,ADCY7,ATM
Type I Diabetes Mellitus	1.72E00	5.45E-02	NA	HLA-A,CD86,IL1B,NOS2,IRF1,HLA-E

Signaling				
Role of Tissue Factor in Cancer	1.72E00	5.45E-02	NA	GNA15,PLAUR,MMP13,IL1B,CSF2,ATM
Xanthine and Xanthosine Salvage	1.72E00	1E00	NA	PNP
Histamine Biosynthesis	1.72E00	1E00	NA	HDC
UDP-N-acetyl-D-galactosamine Biosynthesis I	1.72E00	1E00	NA	GALE
IL-6 Signaling	1.62E00	5.17E-02	0.816	ABCB1,IL36G,IL1A,IL1B,ATM,IL18RAP
Oleate Biosynthesis II (Animals)	1.61E00	1.54E-01	NA	SCD,FADS1
LPS/IL-1 Mediated Inhibition of RXR Function	1.6E00	4.11E-02	NA	GSTA3,ABCB1,IL36G,IL1A,CPT1A,MGST2,CHST3,IL1B,IL18RAP
Role of Osteoblasts, Osteoclasts and Chondrocytes in Rheumatoid Arthritis	1.6E00	4.11E-02	NA	IL36G,IL1A,MMP13,IL1B,CSF2,FZD7,ATM,IL18RAP,ADAMTS4
HMGB1 Signaling	1.56E00	5E-02	0.000	IL1A,VCAM1,CCL2,IL1B,CSF2,ATM
Retinoic acid Mediated Apoptosis Signaling	1.52E00	6.45E-02	-2.000	TNFSF10,PARP11,IRF1,PARP14
Death Receptor Signaling	1.5E00	5.43E-02	-2.236	DAXX,TNFSF10,PARP11,TNFSF15,PARP14
Fcγ Receptor-mediated Phagocytosis in Macrophages and Monocytes	1.49E00	5.38E-02	0.447	DOCK1,FCGR2A,CSF2,FCGR1A,FCGR3A/FCGR3B
Salvage Pathways of Pyrimidine Ribonucleotides	1.49E00	5.38E-02	NA	CMPK2,CDK5,UPP1,UCK2,UCKL1
Non-Small Cell Lung Cancer Signaling	1.45E00	6.15E-02	NA	CDKN2A,ITPR1,RASSF1,ATM
Xenobiotic Metabolism Signaling	1.45E00	3.69E-02	NA	GSTA3,ABCB1,IL1A,CAMK1,MGST2,NQO1,CHST3,IL1B,NOS2,ATM
Hepatic Cholestasis	1.44E00	4.32E-02	NA	ABCB1,IL36G,IL1A,IL1B,CSF2,ADCY7,IL18RAP
Guanine and Guanosine Salvage I	1.42E00	5E-01	NA	PNP

Palmitate Biosynthesis I (Animals)	1.42E00	5E-01	NA	FASN
Fatty Acid Biosynthesis Initiation II	1.42E00	5E-01	NA	FASN
Adenine and Adenosine Salvage I	1.42E00	5E-01	NA	PNP
Inhibition of Matrix Metalloprotease s	1.42E00	7.69E-02	NA	MMP13,MMP12,MMP9
Role of PKR in Interferon Induction and Antiviral Response	1.39E00	7.5E-02	NA	TLR3,FCGR1A,IRF1
Neuroprotective Role of THOP1 in Alzheimer's Disease	1.39E00	7.5E-02	NA	HLA-A,MMP9,HLA-E
Melanoma Signaling	1.34E00	7.14E-02	NA	CDKN2A,CDKN1A,ATM
HIF1α Signaling	1.34E00	4.9E-02	NA	MMP13,MMP12,NOS2,MMP9,ATM
Role of NFAT in Regulation of the Immune Response	1.34E00	4.09E-02	-1.633	GNA15,FCGR2A,CD86,ITPR1,FCGR1A,FCGR3A/FCGR3B,ATM
IL-17 Signaling	1.31E00	5.56E-02	NA	CXCL10,CCL2,NOS2,ATM
Toll-like Receptor Signaling	1.28E00	5.41E-02	1.000	IL36G,IL1A,IL1B,TLR3
Diphthamide Biosynthesis	1.25E00	3.33E-01	NA	DPH5
Methylglyoxal Degradation I	1.25E00	3.33E-01	NA	GLO1
Tyrosine Biosynthesis IV	1.25E00	3.33E-01	NA	PCBD2
Protein Ubiquitination Pathway	1.25E00	3.53E-02	NA	USP3,PSMB9,USP18,HSCB,PSMB10,HLA-A,DNAJB1,TAP1,HSPA4L
NRF2-mediated Oxidative Stress Response	1.24E00	3.89E-02	NA	GSTA3,MGST2,NQO1,DNAJB1,MAFK,EPHX1,ATM
phagosome formation	1.24E00	4.59E-02	NA	FCGR2A,TLR3,FCGR1A,FCGR3A/FCGR3B,ATM
Hematopoiesis from Pluripotent Stem Cells	1.22E00	6.38E-02	NA	IL1A,CSF3,CSF2
VDR/RXR Activation	1.21E00	5.13E-02	NA	CXCL10,MXD1,CDKN1A,CSF2
Primary	1.2E00	6.25E-02	NA	CD40,TNFRSF13B,TAP1

Immunodeficiency Signaling				
CXCR4 Signaling	1.15E00	3.95E-02	-1.342	DOCK1,GNA15,MYLPF,ITPR1,ADCY7,ATM
Huntington's Disease Signaling	1.14E00	3.49E-02	NA	CDK5,GNA15,CASP1,CASP4,DNAJB1,ITPR1,NAPB,ATM
p38 MAPK Signaling	1.13E00	4.27E-02	1.342	DAXX,IL36G,IL1A,IL1B,IL18RAP
Arsenate Detoxification I (Glutaredoxin)	1.13E00	2.5E-01	NA	PNP
Uracil Degradation II (Reductive)	1.13E00	2.5E-01	NA	DPYS
Heme Biosynthesis from Uroporphyrinogen-III I	1.13E00	2.5E-01	NA	CPOX
Thymine Degradation	1.13E00	2.5E-01	NA	DPYS
Phenylalanine Degradation I (Aerobic)	1.13E00	2.5E-01	NA	PCBD2
Gαi Signaling	1.1E00	4.17E-02	-1.000	CNR2,HCAR2,ADCY7,RALGDS,XCR1
NAD Salvage Pathway II	1.06E00	7.69E-02	NA	NT5C3A,NT5C
OX40 Signaling Pathway	1.04E00	4.49E-02	NA	H2-T22,H2-T10,HLA-A,HLA-E
Creatine-phosphate Biosynthesis	1.04E00	2E-01	NA	CKB
Citrulline-Nitric Oxide Cycle	1.04E00	2E-01	NA	NOS2
Galactose Degradation I (Leloir Pathway)	1.04E00	2E-01	NA	GALE
dTMP De Novo Biosynthesis	1.04E00	2E-01	NA	DHFR
IL-15 Production	1.03E00	7.41E-02	NA	IL15,IRF1
Glioma Invasiveness Signaling	1.02E00	5.26E-02	NA	PLAUR,MMP9,ATM
FXR/RXR Activation	1.02E00	3.94E-02	NA	IL36G,IL1A,C3,FASN,IL1B
Adipogenesis pathway	1.02E00	3.94E-02	NA	CDKN2A,NR1D2,CDK5,GTF2H4,FZD7
PI3K Signaling in B Lymphocytes	1.01E00	3.91E-02	NA	CD81,C3,CD40,PLEKHA4,ITPR1
ATM Signaling	9.89E-01	5.08E-02	NA	GADD45G,CDKN1A,ATM
PPAR	9.78E-01	4.26E-02	-2.000	IL36G,IL1A,IL1B,IL18RAP

Signaling				
Glutathione-mediated Detoxification	9.78E-01	6.9E-02	NA	GSTA3,MGST2
Ovarian Cancer Signaling	9.75E-01	3.82E-02	NA	CDKN2A,PTGS1,MMP9,FZD7,ATM
Glioma Signaling	9.66E-01	4.21E-02	0.000	CDKN2A,CAMK1,CDKN1A,ATM
Role of p14/p19ARF in Tumor Suppression	9.53E-01	6.67E-02	NA	CDKN2A,ATM
NF-κB Signaling	9.43E-01	3.47E-02	0.000	IL36G,IL1A,CD40,IL1B,TLR3,ATM
Relaxin Signaling	9.34E-01	3.7E-02	0.000	GNA15,NOS2,ADCY7,MMP9,ATM
Role of JAK1 and JAK3 in γc Cytokine Signaling	9.25E-01	4.76E-02	NA	IL15RA,IL15,ATM
Cell Cycle: G1/S Checkpoint Regulation	9.1E-01	4.69E-02	NA	CDKN2A,CDKN1A,ATM
Acetyl-CoA Biosynthesis I (Pyruvate Dehydrogenase Complex)	8.99E-01	1.43E-01	NA	DLAT
Adenine and Adenosine Salvage III	8.99E-01	1.43E-01	NA	PNP
Aspartate Degradation II	8.99E-01	1.43E-01	NA	MDH1
cAMP-mediated signaling	8.97E-01	3.2E-02	0.816	AKAP12,CAMK1,CNR2,ADORA2B,HCAR2,ADCY7,XCR1
CD40 Signaling	8.95E-01	4.62E-02	NA	CD40,PTGS1,ATM
Cholecystokinin/Gastrin-mediated Signaling	8.95E-01	3.96E-02	1.000	IL36G,IL1A,IL1B,ITPR1
B Cell Development	8.85E-01	6.06E-02	NA	CD40,CD86
PXR/RXR Activation	8.67E-01	4.48E-02	NA	ABCB1,SCD,CPT1A
Inhibition of Angiogenesis by TSP1	8.64E-01	5.88E-02	NA	CD47,MMP9
GDNF Family Ligand-Receptor Interactions	8.53E-01	4.41E-02	NA	DOK7,ITPR1,ATM
HGF Signaling	8.51E-01	3.81E-02	0.000	CDKN2A,DOCK1,CDKN1A,ATM
Superoxide	8.45E-01	1.25E-01	NA	NQO1

Radicals Degradation				
Purine Ribonucleosides Degradation to Ribose-1-phosphate	8.45E-01	1.25E-01	NA	PNP
Nucleotide Excision Repair Pathway	8.44E-01	5.71E-02	NA	ERCC1,GTF2H4
Glioblastoma Multiforme Signaling	8.32E-01	3.42E-02	-1.342	CDKN2A,CDKN1A,ITPR1,FZD7,ATM
Stearate Biosynthesis I (Animals)	8.24E-01	5.56E-02	NA	FASN,ELOVL6
iCOS-iCOSL Signaling in T Helper Cells	8.19E-01	3.7E-02	NA	CD40,PLEKHA4,ITPR1,ATM
GPCR-Mediated Integration of Enteroendocrine Signaling Exemplified by an L Cell	8.13E-01	4.23E-02	NA	GNA15,ITPR1,ADCY7
Renin-Angiotensin Signaling	8.09E-01	3.67E-02	NA	CCL2,ITPR1,ADCY7,ATM
Sphingosine-1-phosphate Signaling	8.09E-01	3.67E-02	0.000	CASP1,CASP4,ADCY7,ATM
JAK/Stat Signaling	8.01E-01	4.17E-02	NA	CDKN1A,STAT2,ATM
Thrombin Signaling	7.99E-01	3.14E-02	-1.342	CAMK1,GNA15,MYLPF,ITPR1,ADCY7,ATM
Pathogenesis of Multiple Sclerosis	7.98E-01	1.11E-01	NA	CXCL10
Prostanoid Biosynthesis	7.98E-01	1.11E-01	NA	PTGS1
Heme Biosynthesis II	7.98E-01	1.11E-01	NA	CPOX
UDP-N-acetyl-D-galactosamine Biosynthesis II	7.98E-01	1.11E-01	NA	GALE
Prolactin Signaling	7.88E-01	4.11E-02	NA	NMI,IRF1,ATM
Aldosterone Signaling in Epithelial Cells	7.81E-01	3.29E-02	NA	HSCB,DNAJB1,ITPR1,ATM,HSPA4L
Docosahexaenoic Acid (DHA) Signaling	7.69E-01	5.13E-02	NA	IL1B,ATM

Dolichyl-diphosphooligosaccharide Biosynthesis	7.57E-01	1E-01	NA	ALG5
Leukocyte Extravasation Signaling	7.49E-01	3.03E-02	0.447	VCAM1,ITGAM,MMP13,MMP12,MMP9,ATM
Tec Kinase Signaling	7.41E-01	3.18E-02	NA	GNA15,GTF2I,TNFSF10,STAT2,ATM
Role of BRCA1 in DNA Damage Response	7.29E-01	3.85E-02	NA	CDKN1A,FANCA,ATM
Cyclins and Cell Cycle Regulation	7.29E-01	3.85E-02	NA	CDKN2A,CDKN1A,ATM
Reelin Signaling in Neurons	7.18E-01	3.8E-02	NA	CDK5,CNR2,ATM
Role of RIG1-like Receptors in Antiviral Innate Immunity	6.89E-01	4.55E-02	NA	IFIH1,IRF7
iNOS Signaling	6.89E-01	4.55E-02	NA	NOS2,IRF1
Cdc42 Signaling	6.68E-01	2.99E-02	NA	H2-T22,H2-T10,HLA-A,MYLPF,HLA-E
GPCR-Mediated Nutrient Sensing in Enteroendocrine Cells	6.66E-01	3.57E-02	NA	GNA15,ITPR1,ADCY7
G-Protein Coupled Receptor Signaling	6.61E-01	2.73E-02	NA	GNA15,CNR2,ADORA2B,HCAR2,ADCY7,XCR1,ATM
Role of IL-17A in Psoriasis	6.55E-01	7.69E-02	NA	CXCL3
Ubiquinol-10 Biosynthesis (Eukaryotic)	6.55E-01	7.69E-02	NA	COQ3
Mevalonate Pathway I	6.55E-01	7.69E-02	NA	MVD
Hereditary Breast Cancer Signaling	6.34E-01	3.1E-02	NA	GADD45G,CDKN1A,FANCA,ATM
CTLA4 Signaling in Cytotoxic T Lymphocytes	6.27E-01	3.41E-02	NA	HLA-A,CD86,ATM
UVA-Induced MAPK Signaling	6.27E-01	3.41E-02	NA	PARP11,PARP14,ATM
DNA Double-	6.26E-01	7.14E-02	NA	ATM

Strand Break Repair by Homologous Recombination				
DNA Double-Strand Break Repair by Non-Homologous End Joining	6.26E-01	7.14E-02	NA	ATM
Superpathway of Citrulline Metabolism	6.26E-01	7.14E-02	NA	NOS2
Colanic Acid Building Blocks Biosynthesis	6.26E-01	7.14E-02	NA	GALE
Assembly of RNA Polymerase II Complex	6.07E-01	4E-02	NA	GTF2H4,TAF7
Chondroitin Sulfate Degradation (Metazoa)	6E-01	6.67E-02	NA	HYAL1
Leukotriene Biosynthesis	6E-01	6.67E-02	NA	MGST2
IL-1 Signaling	6E-01	3.3E-02	NA	IL1A,GNA15,ADCY7
IL-12 Signaling and Production in Macrophages	5.97E-01	2.99E-02	NA	CD40,NOS2,IRF1,ATM
PPARα/RXRα Activation	5.91E-01	2.79E-02	NA	GNA15,FASN,IL1B,ADCY7,IL18RAP
Chronic Myeloid Leukemia Signaling	5.83E-01	3.23E-02	NA	CDKN2A,CDKN1A,ATM
Molecular Mechanisms of Cancer	5.8E-01	2.47E-02	NA	CDKN2A,DAXX,CDK5,GNA15,CDKN1A,ADCY7,RALGDS,FZD7,ATM
Dermatan Sulfate Degradation (Metazoa)	5.76E-01	6.25E-02	NA	HYAL1
Lymphotoxin β Receptor Signaling	5.6E-01	3.7E-02	NA	VCAM1,ATM
γ-linolenate Biosynthesis II (Animals)	5.54E-01	5.88E-02	NA	FADS1
Mitochondrial L-carnitine Shuttle Pathway	5.54E-01	5.88E-02	NA	CPT1A
Superpathway of Geranylgeranyl diphosphate	5.54E-01	5.88E-02	NA	MVD

Biosynthesis I (via Mevalonate)				
ILK Signaling	5.5E-01	2.69E-02	NA	DOCK1,NOS2,MMP9,TESK1,ATM
Role of CHK Proteins in Cell Cycle Checkpoint Control	5.49E-01	3.64E-02	NA	CDKN1A,ATM
EGF Signaling	5.38E-01	3.57E-02	NA	ITPR1,ATM
D-myo-inositol (1,4,5)-trisphosphate Degradation	5.33E-01	5.56E-02	NA	INPP1
Neuropathic Pain Signaling In Dorsal Horn Neurons	5.26E-01	3E-02	NA	CAMK1,ITPR1,ATM
Breast Cancer Regulation by Stathmin1	5.22E-01	2.62E-02	NA	CAMK1,CDKN1A,ITPR1,ADCY7,ATM
Paxillin Signaling	5.19E-01	2.97E-02	NA	DOCK1,ITGAM,ATM
Myc Mediated Apoptosis Signaling	5.18E-01	3.45E-02	NA	CDKN2A,ATM
Glutathione Redox Reactions I	5.14E-01	5.26E-02	NA	MGST2
DNA damage-induced 14-3-3σ Signaling	5.14E-01	5.26E-02	NA	ATM
3-phosphoinositide Degradation	5.11E-01	2.72E-02	NA	INPP4B,PPFIA3,MDP1,NT5C
GM-CSF Signaling	4.79E-01	3.23E-02	NA	CSF2,ATM
Antiproliferative Role of Somatostatin Receptor 2	4.7E-01	3.17E-02	NA	CDKN1A,ATM
PCP pathway	4.7E-01	3.17E-02	NA	SDC3,FZD7
Gαs Signaling	4.62E-01	2.75E-02	NA	ADORA2B,HCAR2,ADCY7
Polyamine Regulation in Colon Cancer	4.62E-01	4.55E-02	NA	MXD1
Pyrimidine Deoxyribonucleotides De Novo Biosynthesis I	4.62E-01	4.55E-02	NA	CMPK2
IL-17A Signaling in Airway Cells	4.61E-01	3.12E-02	NA	CXCL3,ATM
Natural Killer Cell Signaling	4.56E-01	2.73E-02	NA	FCGR2A,FCGR3A/FCGR3B,ATM

Hypoxia Signaling in the Cardiovascular System	4.53E-01	3.08E-02	NA	NQO1,ATM
Corticotropin Releasing Hormone Signaling	4.5E-01	2.7E-02	NA	ITPR1,NOS2,ADCY7
Androgen Signaling	4.5E-01	2.7E-02	NA	GNA15,GTF2H4,DNAJB1
3-phosphoinositide Biosynthesis	4.49E-01	2.53E-02	NA	PPFIA3,MDP1,NT5C,ATM
Role of Lipids/Lipid Rafts in the Pathogenesis of Influenza	4.46E-01	4.35E-02	NA	RSAD2
TCA Cycle II (Eukaryotic)	4.46E-01	4.35E-02	NA	MDH1
Angiopoietin Signaling	4.44E-01	3.03E-02	NA	BIRC5,ATM
Mitotic Roles of Polo-Like Kinase	4.44E-01	3.03E-02	NA	PLK3,PTTG1
Tumoricidal Function of Hepatic Natural Killer Cells	4.32E-01	4.17E-02	NA	SERPINB9
Role of JAK1, JAK2 and TYK2 in Interferon Signaling	4.32E-01	4.17E-02	NA	STAT2
Estrogen-mediated S-phase Entry	4.32E-01	4.17E-02	NA	CDKN1A
Superpathway of D-myo-inositol (1,4,5)-trisphosphate Metabolism	4.32E-01	4.17E-02	NA	INPP1
Role of MAPK Signaling in the Pathogenesis of Influenza	4.2E-01	2.9E-02	NA	CXCL10,CCL2
IL-17A Signaling in Gastric Cells	4.18E-01	4E-02	NA	CXCL10
Gluconeogenesis I	4.18E-01	4E-02	NA	MDH1
CD28 Signaling in T Helper Cells	4.07E-01	2.54E-02	NA	CD86,ITPR1,ATM
Caveolar-	4.05E-01	2.82E-02	NA	ITGAM,HLA-A

mediated Endocytosis Signaling				
T Helper Cell Differentiation	4.05E-01	2.82E-02	NA	CD40,CD86
Chemokine Signaling	4.05E-01	2.82E-02	NA	CAMK1,CCL2
phagosome maturation	3.96E-01	2.5E-02	NA	HLA-A,TAP1,NAPB
Acute Phase Response Signaling	3.95E-01	2.37E-02	1.000	IL36G,IL1A,C3,IL1B
Cell Cycle Control of Chromosomal Replication	3.92E-01	3.7E-02	NA	CDK5
CREB Signaling in Neurons	3.85E-01	2.34E-02	NA	GNA15,ITPR1,ADCY7,ATM
FLT3 Signaling in Hematopoietic Progenitor Cells	3.83E-01	2.7E-02	NA	STAT2,ATM
Leptin Signaling in Obesity	3.83E-01	2.7E-02	NA	ADCY7,ATM
Superpathway of Cholesterol Biosynthesis	3.8E-01	3.57E-02	NA	MVD
HER-2 Signaling in Breast Cancer	3.7E-01	2.63E-02	NA	CDKN1A,ATM
Pyrimidine Ribonucleotides Interconversion	3.69E-01	3.45E-02	NA	CMPK2
Calcium Signaling	3.55E-01	2.25E-02	NA	LETM1,CAMK1,TRPC4,ITPR1
AMPK Signaling	3.51E-01	2.23E-02	NA	CPT1A,FASN,CDKN1A,ATM
Role of NFAT in Cardiac Hypertrophy	3.51E-01	2.23E-02	0.000	CAMK1,ITPR1,ADCY7,ATM
Acute Myeloid Leukemia Signaling	3.5E-01	2.53E-02	NA	PML,ATM
Axonal Guidance Signaling	3.5E-01	2.07E-02	NA	DOCK1,CDK5,GNA15,MYLPF,MMP13,MMP9,FZD7,ATM,ADAMTS4
Cellular Effects of Sildenafil (Viagra)	3.49E-01	2.33E-02	NA	MYLPF,ITPR1,ADCY7
GNRH Signaling	3.49E-01	2.33E-02	NA	GNA15,ITPR1,ADCY7
Pyrimidine Ribonucleotides	3.47E-01	3.23E-02	NA	CMPK2

De Novo Biosynthesis				
D-myo-inositol (1,4,5,6)-Tetrakisphosphate Biosynthesis	3.45E-01	2.31E-02	NA	PPFIA3,MDP1,NT5C
D-myo-inositol (3,4,5,6)-tetrakisphosphate Biosynthesis	3.45E-01	2.31E-02	NA	PPFIA3,MDP1,NT5C
Ceramide Signaling	3.44E-01	2.5E-02	NA	ATM,KSR1
Cytotoxic T Lymphocyte-mediated Apoptosis of Target Cells	3.37E-01	3.12E-02	NA	HLA-A
Prostate Cancer Signaling	3.32E-01	2.44E-02	NA	CDKN1A,ATM
IL-8 Signaling	3.31E-01	2.17E-02	0.000	VCAM1,ITGAM,MMP9,ATM
Phospholipase C Signaling	3.25E-01	2.11E-02	NA	FCGR2A,MYLPF,ITPR1,ADCY7,RALGDS
Melanocyte Development and Pigmentation Signaling	3.21E-01	2.38E-02	NA	ADCY7,ATM
ERK/MAPK Signaling	3.19E-01	2.14E-02	1.000	DOCK1,VRK2,ATM,KSR1
IL-9 Signaling	3.18E-01	2.94E-02	NA	ATM
Oncostatin M Signaling	3.18E-01	2.94E-02	NA	MMP13
TR/RXR Activation	3.15E-01	2.35E-02	NA	FASN,ATM
FGF Signaling	3.15E-01	2.35E-02	NA	ITPR1,ATM
HIPPO signaling	3.1E-01	2.33E-02	NA	DLG3,RASSF1
Coagulation System	3.09E-01	2.86E-02	NA	PLAUR
IL-17A Signaling in Fibroblasts	3.09E-01	2.86E-02	NA	CCL2
Triacylglycerol Biosynthesis	3.09E-01	2.86E-02	NA	ELOVL6
α-Adrenergic Signaling	3.04E-01	2.3E-02	NA	ITPR1,ADCY7
FAK Signaling	3.04E-01	2.3E-02	NA	DOCK1,ATM
Virus Entry via Endocytic Pathways	2.94E-01	2.25E-02	NA	HLA-A,ATM
PAK Signaling	2.94E-01	2.25E-02	NA	MYLPF,ATM
Synaptic Long Term Depression	2.92E-01	2.11E-02	NA	GNA15,ITPR1,NOS2
eNOS Signaling	2.92E-01	2.11E-02	NA	ITPR1,ADCY7,ATM

Superpathway of Inositol Phosphate Compounds	2.91E-01	2.05E-02	NA	PPFIA3,MDP1,NT5C,ATM
April Mediated Signaling	2.85E-01	2.63E-02	NA	TNFRSF13B
D-myo-inositol-5-phosphate Metabolism	2.76E-01	2.05E-02	NA	PPFIA3,MDP1,NT5C
Gαq Signaling	2.73E-01	2.04E-02	NA	GNA15,ITPR1,ATM
Integrin Signaling	2.71E-01	1.99E-02	NA	DOCK1,ITGAM,TSPAN4,ATM
B Cell Activating Factor Signaling	2.7E-01	2.5E-02	NA	TNFRSF13B
Thyroid Cancer Signaling	2.7E-01	2.5E-02	NA	CXCL10
SAPK/JNK Signaling	2.69E-01	2.13E-02	NA	DAXX,ATM
Mouse Embryonic Stem Cell Pluripotency	2.65E-01	2.11E-02	NA	FZD7,ATM
MIF Regulation of Innate Immunity	2.63E-01	2.44E-02	NA	NOS2
FcγRIIB Signaling in B Lymphocytes	2.63E-01	2.44E-02	NA	ATM
Amyotrophic Lateral Sclerosis Signaling	2.52E-01	2.04E-02	NA	CASP1,ATM
CDK5 Signaling	2.47E-01	2.02E-02	NA	CDK5,ADCY7
Telomerase Signaling	2.47E-01	2.02E-02	NA	CDKN1A,ATM
Gap Junction Signaling	2.44E-01	1.94E-02	NA	ITPR1,ADCY7,ATM
Nitric Oxide Signaling in the Cardiovascular System	2.43E-01	2E-02	NA	ITPR1,ATM
Dermatan Sulfate Biosynthesis (Late Stages)	2.43E-01	2.27E-02	NA	CHST3
Serotonin Receptor Signaling	2.43E-01	2.27E-02	NA	ADCY7
Chondroitin Sulfate Biosynthesis (Late Stages)	2.31E-01	2.17E-02	NA	CHST3

Ephrin A Signaling	2.19E-01	2.08E-02	NA	ATM
Heparan Sulfate Biosynthesis (Late Stages)	2.09E-01	2E-02	NA	CHST3
Amyloid Processing	2.04E-01	1.96E-02	NA	CDK5
CNTF Signaling	1.99E-01	1.92E-02	NA	ATM
Endometrial Cancer Signaling	1.99E-01	1.92E-02	NA	ATM

Supplementary Table S6. Canonical pathways respond to FruArg treatment (LPS+FruArg vs. LPS).

Ingenuity Canonical Pathways	-log(p-value)	Ratio	z-score	Molecules
Aryl Hydrocarbon Receptor Signaling	6.14E00	6.43E-02	NA	CDKN2A,GSTA3,IL1A,MGST2,GSTM5,IL1B,DHFR,GSTP1,ATM
TREM1 Signaling	5.97E00	9.33E-02	NA	CXCL3,CCL2,CD40,NLRC3,CASP1,CD86,IL1B
Systemic Lupus Erythematosus Signaling	5.48E00	4.65E-02	NA	RNU1-4,IL1A,CD40,FCGR2A,CD86,IL1B,FCGR1A,FCGR3A/FCGR3B,PRPF38A,ATM
Xenobiotic Metabolism Signaling	5.4E00	4.06E-02	NA	GSTA3,HMOX1,ABCB1,IL1A,FTL,MGST2,GSTM5,IL1B,NOS2,GSTP1,ATM
NRF2-mediated Oxidative Stress Response	5.23E00	5E-02	2.000	GSTA3,HMOX1,FTL,SCARB1,MGST2,GSTM5,GSTP1,EPHX1,ATM
Differential Regulation of Cytokine Production in Intestinal Epithelial Cells by IL-17A and IL-17F	4.71E00	1.74E-01	NA	IL1A,CCL2,IL1B,CSF3
LPS/IL-1 Mediated Inhibition of RXR Function	4.55E00	4.11E-02	NA	GSTA3,ABCB1,IL1A,SCARB1,MGST2,GSTM5,XPO1,IL1B,GSTP1
Dendritic Cell Maturation	4.39E00	4.52E-02	-0.707	IL1A,CD40,FCGR2A,CD86,IL1B,FCGR1A,FCGR3A/FCGR3B,ATM
Glutathione-mediated Detoxification	4.29E00	1.38E-01	NA	GSTA3,MGST2,GSTM5,GSTP1
IL-10 Signaling	3.91E00	7.35E-02	NA	HMOX1,SOCS3,IL1A,FCGR2A,IL1B
Role of NFAT in Regulation of the Immune	3.64E00	4.09E-02	-1.342	FCGR2A,CD86,XPO1,FCGR1A,FCGR3A/FCGR3B,ORAI1,ATM

Response				
Differential Regulation of Cytokine Production in Macrophages and T Helper Cells by IL-17A and IL-17F	3.57E00	1.67E-01	NA	CCL2,IL1B,CSF3
Role of Hypercytokinemia/hyperchemokinemia in the Pathogenesis of Influenza	3.57E00	9.09E-02	NA	CXCL10,IL1A,CCL2,IL1B
Role of Macrophages, Fibroblasts and Endothelial Cells in Rheumatoid Arthritis	3.56E00	3.04E-02	NA	SOCS3,IL1A,CCL2,IL1B,NOS2,FCGR1A,FCGR3A/FCGR3B,PDGFB,ATM
Granulocyte Adhesion and Diapedesis	3.55E00	3.95E-02	NA	CXCL10,CXCL3,IL1A,CCL2,IL1B,MMP12,CSF3
Communication between Innate and Adaptive Immune Cells	3.36E00	5.62E-02	NA	CXCL10,IL1A,CD40,CD86,IL1B
Role of IL-17A in Arthritis	3.23E00	7.41E-02	NA	CXCL3,CCL2,NOS2,ATM
Sphingosine-1-phosphate Signaling	2.96E00	4.59E-02	0.447	CASP1,CASP4,ADCY7,PDGFB,ATM
Acute Phase Response Signaling	2.87E00	3.55E-02	1.342	HMOX1,SOCS3,IL1A,HP,FTL,IL1B
IL-6 Signaling	2.84E00	4.31E-02	-0.447	ABCB1,SOCS3,IL1A,IL1B,ATM
Endothelin-1 Signaling	2.83E00	3.49E-02	-1.000	HMOX1,CASP1,CASP4,NOS2,ADCY7,ATM
HMGB1 Signaling	2.77E00	4.17E-02	-0.447	IL1A,CCL2,HAT1,IL1B,ATM
IL-17 Signaling	2.76E00	5.56E-02	NA	CXCL10,CCL2,NOS2,ATM
Atherosclerosis Signaling	2.71E00	4.03E-02	NA	IL1A,CCL2,CD40,IL1B,PDGFB
Hepatic Fibrosis / Hepatic Stellate Cell Activation	2.7E00	3.28E-02	NA	CXCL3,IL1A,CCL2,CD40,IL1B,PDGFB
Role of Pattern Recognition Receptors in Recognition of Bacteria and Viruses	2.68E00	3.97E-02	0.000	IL1A,CASP1,IL1B,CLEC6A,ATM

Agranulocyte Adhesion and Diapedesis	2.63E00	3.17E-02	NA	CXCL10,CXCL3,IL1A,CCL2,IL1B,MMP12
Glucocorticoid Receptor Signaling	2.44E00	2.55E-02	NA	CXCL3,CCL2,SLPI,IL1B,NOS2,FCGR1A,ATM
Altered T Cell and B Cell Signaling in Rheumatoid Arthritis	2.44E00	4.55E-02	NA	IL1A,CD40,CD86,IL1B
Crosstalk between Dendritic Cells and Natural Killer Cells	2.42E00	4.49E-02	NA	CD40,CD69,CD86,TNFSF10
Role of IL-17F in Allergic Inflammatory Airway Diseases	2.42E00	6.82E-02	NA	CXCL10,CCL2,IL1B
MSP-RON Signaling Pathway	2.37E00	6.52E-02	NA	CCL2,NOS2,ATM
Fcγ Receptor-mediated Phagocytosis in Macrophages and Monocytes	2.36E00	4.3E-02	0.000	HMOX1,FCGR2A,FCGR1A,FCGR3A/FCGR3B
Graft-versus-Host Disease Signaling	2.32E00	6.25E-02	NA	IL1A,CD86,IL1B
Pancreatic Adenocarcinom a Signaling	2.16E00	3.77E-02	1.000	CDKN2A,HMOX1,RALGDS,ATM
Role of Cytokines in Mediating Communication between Immune Cells	2.15E00	5.45E-02	NA	IL1A,IL1B,CSF3
Histamine Biosynthesis	2.15E00	1E00	NA	HDC
phagosome formation	2.11E00	3.67E-02	NA	FCGR2A,FCGR1A,FCGR3A/FCGR3B,ATM
Type I Diabetes Mellitus Signaling	2.1E00	3.64E-02	NA	SOCS3,CD86,IL1B,NOS2
EIF2 Signaling	1.98E00	2.7E-02	0.000	EIF5,RPS8,RPL41,ATM,RPL38
LXR/RXR Activation	1.96E00	3.31E-02	0.000	IL1A,CCL2,IL1B,NOS2
Macropinocytos is Signaling	1.9E00	4.41E-02	NA	CSF1R,PDGFB,ATM
Glycolysis I	1.87E00	8E-02	NA	GAPDH,ALDOC
Gluconeogenesi	1.87E00	8E-02	NA	GAPDH,ALDOC

s I				
Prolactin Signaling	1.82E00	4.11E-02	NA	SOCS3,NMI,ATM
Leptin Signaling in Obesity	1.8E00	4.05E-02	NA	SOCS3,ADCY7,ATM
Role of p14/p19ARF in Tumor Suppression	1.72E00	6.67E-02	NA	CDKN2A,ATM
NADH Repair	1.67E00	3.33E-01	NA	GAPDH
TR/RXR Activation	1.64E00	3.53E-02	NA	HP,SCARB1,ATM
B Cell Development	1.64E00	6.06E-02	NA	CD40,CD86
IL-9 Signaling	1.61E00	5.88E-02	NA	SOCS3,ATM
Colorectal Cancer Metastasis Signaling	1.57E00	2.12E-02	0.447	MMP12,NOS2,ADCY7,RALGDS,ATM
Death Receptor Signaling	1.55E00	3.26E-02	NA	TNFSF10,PARP11,TNFSF15
Heme Degradation	1.55E00	2.5E-01	NA	HMOX1
Hepatic Cholestasis	1.54E00	2.47E-02	NA	ABCB1,IL1A,IL1B,ADCY7
PPAR Signaling	1.53E00	3.19E-02	NA	IL1A,IL1B,PDGFB
Glioma Signaling	1.52E00	3.16E-02	NA	CDKN2A,PDGFB,ATM
Docosahexaenoic Acid (DHA) Signaling	1.5E00	5.13E-02	NA	IL1B,ATM
Citrulline-Nitric Oxide Cycle	1.45E00	2E-01	NA	NOS2
Galactose Degradation I (Leloir Pathway)	1.45E00	2E-01	NA	GALK1
dTMP De Novo Biosynthesis	1.45E00	2E-01	NA	DHFR
NF-κB Signaling	1.45E00	2.31E-02	0.000	IL1A,CD40,IL1B,ATM
Melanoma Signaling	1.44E00	4.76E-02	NA	CDKN2A,ATM
HIF1α Signaling	1.44E00	2.94E-02	NA	MMP12,NOS2,ATM
Glycerol Degradation I	1.38E00	1.67E-01	NA	Gyk
Renin-Angiotensin Signaling	1.37E00	2.75E-02	NA	CCL2,ADCY7,ATM
Natural Killer Cell Signaling	1.36E00	2.73E-02	NA	FCGR2A,FCGR3A/FCGR3B,ATM
Role of Tissue	1.36E00	2.73E-02	NA	PLAUR,IL1B,ATM

Factor in Cancer				
Autoimmune Thyroid Disease Signaling	1.35E00	4.26E-02	NA	CD40,CD86
Hematopoiesis from Pluripotent Stem Cells	1.35E00	4.26E-02	NA	IL1A,CSF3
Molecular Mechanisms of Cancer	1.33E00	1.64E-02	NA	CDKN2A,CDK5,HAT1,ADCY7,RALGDS,ATM
Cell Cycle: G2/M DNA Damage Checkpoint Regulation	1.32E00	4.08E-02	NA	CDKN2A,ATM
p38 MAPK Signaling	1.29E00	2.56E-02	NA	IL1A,DDIT3,IL1B
Semaphorin Signaling in Neurons	1.26E00	3.77E-02	NA	PLXNA1,CDK5
Airway Pathology in Chronic Obstructive Pulmonary Disease	1.26E00	1.25E-01	NA	CXCL3
Pathogenesis of Multiple Sclerosis	1.21E00	1.11E-01	NA	CXCL10
Sucrose Degradation V (Mammalian)	1.21E00	1.11E-01	NA	ALDOC
Glioma Invasiveness Signaling	1.21E00	3.51E-02	NA	PLAUR,ATM
FXR/RXR Activation	1.21E00	2.36E-02	NA	IL1A,SCARB1,IL1B
Adipogenesis pathway	1.21E00	2.36E-02	NA	CDKN2A,DDIT3,CDK5
Myc Mediated Apoptosis Signaling	1.19E00	3.45E-02	NA	CDKN2A,ATM
Phospholipases	1.19E00	3.45E-02	NA	HMOX1,LIPG
IL-12 Signaling and Production in Macrophages	1.15E00	2.24E-02	NA	CD40,NOS2,ATM
Role of Osteoblasts, Osteoclasts and Chondrocytes in Rheumatoid Arthritis	1.15E00	1.83E-02	NA	IL1A,IL1B,CSF1R,ATM

Relaxin Signaling	1.14E00	2.22E-02	NA	NOS2,ADCY7,ATM
Retinoic acid Mediated Apoptosis Signaling	1.14E00	3.23E-02	NA	TNFSF10,PARP11
Role of JAK1 and JAK3 in γc Cytokine Signaling	1.13E00	3.17E-02	NA	SOCS3,ATM
IL-17A Signaling in Airway Cells	1.12E00	3.12E-02	NA	CXCL3,ATM
Cell Cycle: G1/S Checkpoint Regulation	1.12E00	3.12E-02	NA	CDKN2A,ATM
CD40 Signaling	1.11E00	3.08E-02	NA	CD40,ATM
Non-Small Cell Lung Cancer Signaling	1.11E00	3.08E-02	NA	CDKN2A,ATM
eNOS Signaling	1.09E00	2.11E-02	NA	PRKG1,ADCY7,ATM
Huntington's Disease Signaling	1.09E00	1.75E-02	NA	CDK5,CASP1,CASP4,ATM
Hematopoiesis from Multipotent Stem Cells	1.09E00	8.33E-02	NA	CSF3
Erythropoietin Signaling	1.08E00	2.99E-02	NA	SOCS3,ATM
GDNF Family Ligand-Receptor Interactions	1.07E00	2.94E-02	NA	DOK7,ATM
Glioblastoma Multiforme Signaling	1.06E00	2.05E-02	NA	CDKN2A,PDGFB,ATM
Growth Hormone Signaling	1.06E00	2.9E-02	NA	SOCS3,ATM
Role of MAPK Signaling in the Pathogenesis of Influenza	1.06E00	2.9E-02	NA	CXCL10,CCL2
Role of IL-17A in Psoriasis	1.05E00	7.69E-02	NA	CXCL3
Choline Biosynthesis III	1.05E00	7.69E-02	NA	HMOX1
Guanosine Nucleotides Degradation III	1.05E00	7.69E-02	NA	NT5C3A
Phospholipase C Signaling	1.05E00	1.69E-02	NA	HMOX1,FCGR2A,ADCY7,RALGDS
T Helper Cell	1.04E00	2.82E-02	NA	CD40,CD86

Differentiation				
Renal Cell Carcinoma Signaling	1.04E00	2.82E-02	NA	PDGFB,ATM
GPCR-Mediated Integration of Enteroendocrine Signaling Exemplified by an L Cell	1.04E00	2.82E-02	NA	SSTR5,ADCY7
JAK/Stat Signaling	1.03E00	2.78E-02	NA	SOCS3,ATM
DNA Double-Strand Break Repair by Homologous Recombination	1.02E00	7.14E-02	NA	ATM
DNA Double-Strand Break Repair by Non-Homologous End Joining	1.02E00	7.14E-02	NA	ATM
Superpathway of Citrulline Metabolism	1.02E00	7.14E-02	NA	NOS2
Urate Biosynthesis/Inosine 5'-phosphate Degradation	1.02E00	7.14E-02	NA	NT5C3A
Colanic Acid Building Blocks Biosynthesis	1.02E00	7.14E-02	NA	GALK1
Toll-like Receptor Signaling	1.01E00	2.7E-02	NA	IL1A,IL1B
Gap Junction Signaling	1E00	1.94E-02	NA	PRKG1,ADCY7,ATM
Leukotriene Biosynthesis	9.93E-01	6.67E-02	NA	MGST2
PDGF Signaling	9.81E-01	2.6E-02	NA	PDGFB,ATM
VDR/RXR Activation	9.72E-01	2.56E-02	NA	CXCL10,MXD1
Cyclins and Cell Cycle Regulation	9.72E-01	2.56E-02	NA	CDKN2A,ATM
Dopamine-DARPP32 Feedback in cAMP Signaling	9.68E-01	1.86E-02	NA	PRKG1,CDK5,ADCY7
Reelin Signaling in	9.63E-01	2.53E-02	NA	CDK5,ATM

Neurons				
Acute Myeloid Leukemia Signaling	9.63E-01	2.53E-02	NA	CSF1R,ATM
RAN Signaling	9.41E-01	5.88E-02	NA	XPO1
Adenosine Nucleotides Degradation II	9.41E-01	5.88E-02	NA	NT5C3A
Prostate Cancer Signaling	9.36E-01	2.44E-02	NA	GSTP1,ATM
Melanocyte Development and Pigmentation Signaling	9.19E-01	2.38E-02	NA	ADCY7,ATM
Allograft Rejection Signaling	9.19E-01	2.38E-02	NA	CD40,CD86
GADD45 Signaling	8.96E-01	5.26E-02	NA	ATM
Glutathione Redox Reactions I	8.96E-01	5.26E-02	NA	MGST2
DNA damage-induced 14-3-3σ Signaling	8.96E-01	5.26E-02	NA	ATM
Bladder Cancer Signaling	8.94E-01	2.3E-02	NA	CDKN2A,MMP12
CTLA4 Signaling in Cytotoxic T Lymphocytes	8.86E-01	2.27E-02	NA	CD86,ATM
UVA-Induced MAPK Signaling	8.86E-01	2.27E-02	NA	PARP11,ATM
PAK Signaling	8.78E-01	2.25E-02	NA	PDGFB,ATM
Purine Nucleotides Degradation II (Aerobic)	8.75E-01	5E-02	NA	NT5C3A
Sertoli Cell-Sertoli Cell Junction Signaling	8.73E-01	1.69E-02	NA	SPTBN2,PRKG1,NOS2
IL-1 Signaling	8.63E-01	2.2E-02	NA	IL1A,ADCY7
Endoplasmic Reticulum Stress Pathway	8.56E-01	4.76E-02	NA	DDIT3
Chronic Myeloid Leukemia Signaling	8.47E-01	2.15E-02	NA	CDKN2A,ATM
Maturity Onset Diabetes of Young	8.37E-01	4.55E-02	NA	GAPDH

(MODY) Signaling				
Polyamine Regulation in Colon Cancer	8.37E-01	4.55E-02	NA	MXD1
mTOR Signaling	8.22E-01	1.6E-02	NA	HMOX1,RPS8,ATM
Role of Lipids/Lipid Rafts in the Pathogenesis of Influenza	8.19E-01	4.35E-02	NA	RSAD2
IGF-1 Signaling	8.18E-01	2.06E-02	NA	SOCS3,ATM
Amyotrophic Lateral Sclerosis Signaling	8.11E-01	2.04E-02	NA	CASP1,ATM
p53 Signaling	8.11E-01	2.04E-02	NA	CDKN2A,ATM
CDK5 Signaling	8.04E-01	2.02E-02	NA	CDK5,ADCY7
IL-22 Signaling	8.02E-01	4.17E-02	NA	SOCS3
Tumoricidal Function of Hepatic Natural Killer Cells	8.02E-01	4.17E-02	NA	SERPINB9
Triacylglycerol Degradation	8.02E-01	4.17E-02	NA	LIPG
Nitric Oxide Signaling in the Cardiovascular System	7.98E-01	2E-02	NA	PRKG1,ATM
Cholecystokinin/Gastrin-mediated Signaling	7.91E-01	1.98E-02	NA	IL1A,IL1B
IL-17A Signaling in Gastric Cells	7.86E-01	4E-02	NA	CXCL10
Role of JAK family kinases in IL-6-type Cytokine Signaling	7.86E-01	4E-02	NA	SOCS3
NAD Salvage Pathway II	7.7E-01	3.85E-02	NA	NT5C3A
HGF Signaling	7.65E-01	1.9E-02	NA	CDKN2A,ATM
Cell Cycle Control of Chromosomal Replication	7.56E-01	3.7E-02	NA	CDK5
iCOS-iCOSL Signaling in T Helper Cells	7.46E-01	1.85E-02	NA	CD40,ATM
Corticotropin Releasing	7.28E-01	1.8E-02	NA	NOS2,ADCY7

Hormone Signaling				
Axonal Guidance Signaling	7.04E-01	1.15E-02	NA	PLXNA1,ADAMTS6,CDK5,PDGFB,ATM
Type II Diabetes Mellitus Signaling	6.93E-01	1.71E-02	NA	SOCS3,ATM
CD28 Signaling in T Helper Cells	6.88E-01	1.69E-02	NA	CD86,ATM
PKCθ Signaling in T Lymphocytes	6.88E-01	1.69E-02	NA	CD86,ATM
P2Y Purigenic Receptor Signaling Pathway	6.82E-01	1.68E-02	NA	ADCY7,ATM
Retinol Biosynthesis	6.77E-01	3.03E-02	NA	LIPG
Gαi Signaling	6.77E-01	1.67E-02	NA	ADCY7,RALGDS
Coagulation System	6.55E-01	2.86E-02	NA	PLAUR
IL-17A Signaling in Fibroblasts	6.55E-01	2.86E-02	NA	CCL2
Role of JAK2 in Hormone-like Cytokine Signaling	6.55E-01	2.86E-02	NA	SOCS3
PI3K Signaling in B Lymphocytes	6.36E-01	1.56E-02	NA	CD81,CD40
Cellular Effects of Sildenafil (Viagra)	6.31E-01	1.55E-02	NA	PRKG1,ADCY7
Ovarian Cancer Signaling	6.21E-01	1.53E-02	NA	CDKN2A,ATM
Netrin Signaling	6.14E-01	2.56E-02	NA	PRKG1
Inhibition of Matrix Metalloproteases	6.14E-01	2.56E-02	NA	MMP12
Cardiac β-adrenergic Signaling	6.12E-01	1.5E-02	NA	AKAP12,ADCY7
Human Embryonic Stem Cell Pluripotency	6.07E-01	1.49E-02	NA	PDGFB,ATM
Insulin Receptor Signaling	6.07E-01	1.49E-02	NA	SOCS3,ATM

Role of PKR in Interferon Induction and Antiviral Response	6.04E-01	2.5E-02	NA	FCGR1A
Thyroid Cancer Signaling	6.04E-01	2.5E-02	NA	CXCL10
MIF Regulation of Innate Immunity	5.95E-01	2.44E-02	NA	NOS2
FcγRIIB Signaling in B Lymphocytes	5.95E-01	2.44E-02	NA	ATM
Mechanisms of Viral Exit from Host Cells	5.95E-01	2.44E-02	NA	XPO1
Synaptic Long Term Depression	5.71E-01	1.41E-02	NA	PRKG1,NOS2
iNOS Signaling	5.69E-01	2.27E-02	NA	NOS2
Serotonin Receptor Signaling	5.69E-01	2.27E-02	NA	ADCY7
Regulation of eIF4 and p70S6K Signaling	5.55E-01	1.37E-02	NA	RPS8,ATM
Gαq Signaling	5.5E-01	1.36E-02	NA	HMOX1,ATM
Primary Immunodeficiency Signaling	5.37E-01	2.08E-02	NA	CD40
Ephrin A Signaling	5.37E-01	2.08E-02	NA	ATM
CXCR4 Signaling	5.31E-01	1.32E-02	NA	ADCY7,ATM
Aldosterone Signaling in Epithelial Cells	5.31E-01	1.32E-02	NA	ATM,HSPA4L
Amyloid Processing	5.15E-01	1.96E-02	NA	CDK5
Tec Kinase Signaling	5.12E-01	1.27E-02	NA	TNFSF10,ATM
3-phosphoinositide Biosynthesis	5.08E-01	1.27E-02	NA	SOCS3,ATM
CNTF Signaling	5.08E-01	1.92E-02	NA	ATM
Endometrial Cancer Signaling	5.08E-01	1.92E-02	NA	ATM
UVB-Induced MAPK Signaling	5.01E-01	1.89E-02	NA	ATM
IL-2 Signaling	5.01E-01	1.89E-02	NA	ATM
Lymphotoxin β	4.94E-01	1.85E-02	NA	ATM

Receptor Signaling				
Unfolded protein response	4.94E-01	1.85E-02	NA	DDIT3
Thrombopoietin Signaling	4.88E-01	1.82E-02	NA	ATM
Role of CHK Proteins in Cell Cycle Checkpoint Control	4.88E-01	1.82E-02	NA	ATM
EGF Signaling	4.81E-01	1.79E-02	NA	ATM
Nur77 Signaling in T Lymphocytes	4.75E-01	1.75E-02	NA	CD86
ErbB2-ErbB3 Signaling	4.75E-01	1.75E-02	NA	ATM
CREB Signaling in Neurons	4.63E-01	1.17E-02	NA	ADCY7,ATM
ATM Signaling	4.63E-01	1.69E-02	NA	ATM
ErbB4 Signaling	4.57E-01	1.67E-02	NA	ATM
B Cell Receptor Signaling	4.53E-01	1.15E-02	NA	FCGR2A,ATM
GM-CSF Signaling	4.46E-01	1.61E-02	NA	ATM
Activation of IRF by Cytosolic Pattern Recognition Receptors	4.4E-01	1.59E-02	NA	CD40
Antiproliferative Role of Somatostatin Receptor 2	4.4E-01	1.59E-02	NA	ATM
Estrogen-Dependent Breast Cancer Signaling	4.4E-01	1.59E-02	NA	ATM
PPARα/RXRα Activation	4.38E-01	1.12E-02	NA	IL1B,ADCY7
Role of NFAT in Cardiac Hypertrophy	4.38E-01	1.12E-02	NA	ADCY7,ATM
Production of Nitric Oxide and Reactive Oxygen Species in Macrophages	4.35E-01	1.11E-02	NA	NOS2,ATM
Calcium-induced T Lymphocyte	4.35E-01	1.56E-02	NA	ORAI1

Apoptosis				
Role of PI3K/AKT Signaling in the Pathogenesis of Influenza	4.35E-01	1.56E-02	NA	ATM
Pyridoxal 5'-phosphate Salvage Pathway	4.35E-01	1.56E-02	NA	CDK5
Eicosanoid Signaling	4.35E-01	1.56E-02	NA	ALOX5AP
Hypoxia Signaling in the Cardiovascular System	4.29E-01	1.54E-02	NA	ATM
IL-15 Signaling	4.24E-01	1.52E-02	NA	ATM
Angiopoietin Signaling	4.24E-01	1.52E-02	NA	ATM
IL-8 Signaling	4.23E-01	1.09E-02	NA	HMOX1,ATM
Clathrin-mediated Endocytosis Signaling	4.2E-01	1.08E-02	NA	PDGFB,ATM
PXR/RXR Activation	4.19E-01	1.49E-02	NA	ABCB1
GABA Receptor Signaling	4.19E-01	1.49E-02	NA	ADCY7
Neurotrophin/TRK Signaling	4.19E-01	1.49E-02	NA	ATM
ILK Signaling	4.17E-01	1.08E-02	NA	NOS2,ATM
Thrombin Signaling	4.03E-01	1.05E-02	NA	ADCY7,ATM
Breast Cancer Regulation by Stathmin1	4.03E-01	1.05E-02	NA	ADCY7,ATM
IL-3 Signaling	4E-01	1.41E-02	NA	ATM
Small Cell Lung Cancer Signaling	4E-01	1.41E-02	NA	ATM
PEDF Signaling	4E-01	1.41E-02	NA	ATM
Chemokine Signaling	4E-01	1.41E-02	NA	CCL2
Superpathway of Inositol Phosphate Compounds	3.92E-01	1.03E-02	NA	SOCS3,ATM
LPS-stimulated MAPK Signaling	3.9E-01	1.37E-02	NA	ATM
NF-κB Activation by Viruses	3.9E-01	1.37E-02	NA	ATM
STAT3	3.9E-01	1.37E-02	NA	SOCS3

Pathway				
FLT3 Signaling in Hematopoietic Progenitor Cells	3.86E-01	1.35E-02	NA	ATM
Leukocyte Extravasation Signaling	3.84E-01	1.01E-02	NA	MMP12,ATM
IL-4 Signaling	3.81E-01	1.33E-02	NA	ATM
HER-2 Signaling in Breast Cancer	3.77E-01	1.32E-02	NA	ATM
VEGF Family Ligand-Receptor Interactions	3.77E-01	1.32E-02	NA	ATM
Integrin Signaling	3.77E-01	9.95E-03	NA	PDGFB,ATM
Role of BRCA1 in DNA Damage Response	3.69E-01	1.28E-02	NA	ATM
Dopamine Receptor Signaling	3.69E-01	1.28E-02	NA	ADCY7
Ceramide Signaling	3.6E-01	1.25E-02	NA	ATM
GPCR-Mediated Nutrient Sensing in Enteroendocrine Cells	3.45E-01	1.19E-02	NA	ADCY7
FGF Signaling	3.41E-01	1.18E-02	NA	ATM
Actin Cytoskeleton Signaling	3.38E-01	9.22E-03	NA	PDGFB,ATM
ErbB Signaling	3.37E-01	1.16E-02	NA	ATM
cAMP-mediated signaling	3.34E-01	9.13E-03	NA	AKAP12,ADCY7
α-Adrenergic Signaling	3.34E-01	1.15E-02	NA	ADCY7
FAK Signaling	3.34E-01	1.15E-02	NA	ATM
Neuregulin Signaling	3.3E-01	1.14E-02	NA	CDK5
RANK Signaling in Osteoclasts	3.3E-01	1.14E-02	NA	ATM
Virus Entry via Endocytic Pathways	3.27E-01	1.12E-02	NA	ATM
Cardiac Hypertrophy Signaling	3.25E-01	8.97E-03	NA	ADCY7,ATM

VEGF Signaling	3.16E-01	1.09E-02	NA	ATM
Salvage Pathways of Pyrimidine Ribonucleotides	3.13E-01	1.08E-02	NA	CDK5
SAPK/JNK Signaling	3.1E-01	1.06E-02	NA	ATM
Mouse Embryonic Stem Cell Pluripotency	3.07E-01	1.05E-02	NA	ATM
T Cell Receptor Signaling	3E-01	1.03E-02	NA	ATM
Telomerase Signaling	2.94E-01	1.01E-02	NA	ATM
Antioxidant Action of Vitamin C	2.94E-01	1.01E-02	NA	HMOX1
Neuropathic Pain Signaling In Dorsal Horn Neurons	2.91E-01	1E-02	NA	ATM
Paxillin Signaling	2.88E-01	9.9E-03	NA	ATM
Rac Signaling	2.8E-01	9.62E-03	NA	ATM
NGF Signaling	2.71E-01	9.35E-03	NA	ATM
Fc Epsilon RI Signaling	2.69E-01	9.26E-03	NA	ATM
fMLP Signaling in Neutrophils	2.69E-01	9.26E-03	NA	ATM
Gαs Signaling	2.66E-01	9.17E-03	NA	ADCY7
G-Protein Coupled Receptor Signaling	2.63E-01	7.81E-03	NA	ADCY7,ATM
Role of NANOG in Mammalian Embryonic Stem Cell Pluripotency	2.61E-01	9.01E-03	NA	ATM
14-3-3-mediated Signaling	2.46E-01	8.55E-03	NA	ATM
CCR3 Signaling in Eosinophils	2.46E-01	8.55E-03	NA	ATM
Gα12/13 Signaling	2.46E-01	8.55E-03	NA	ATM
p70S6K Signaling	2.41E-01	8.4E-03	NA	ATM
Sperm Motility	2.37E-01	8.26E-03	NA	PRKG1
Gustation Pathway	2.37E-01	8.26E-03	NA	ADCY7

RhoA Signaling	2.34E-01	8.2E-03	NA	PLXNA1
PI3K/AKT Signaling	2.32E-01	8.13E-03	NA	GDF15
GNRH Signaling	2.19E-01	7.75E-03	NA	ADCY7
Hereditary Breast Cancer Signaling	2.19E-01	7.75E-03	NA	ATM
D-myo-inositol (1,4,5,6)-Tetrakisphosphate Biosynthesis	2.17E-01	7.69E-03	NA	SOCS3
D-myo-inositol (3,4,5,6)-tetrakisphosphate Biosynthesis	2.17E-01	7.69E-03	NA	SOCS3
